# Synthesis of chiral *anti*-1,2-diamine derivatives through copper(I)-catalyzed asymmetric α-addition of ketimines to aldimines

**DOI:** 10.1038/s41467-020-18235-9

**Published:** 2020-09-08

**Authors:** Xu-Cheng Gan, Cheng-Yuan Zhang, Feng Zhong, Ping Tian, Liang Yin

**Affiliations:** 1 The Research Center of Chiral Drugs, Innovation Research Institute of Traditional Chinese Medicine and China-Thailand Joint Research Institute of Natural Medicine, Shanghai University of Traditional Chinese Medicine, 201203 Shanghai, China; 2 CAS Key Laboratory of Synthetic Chemistry of Natural Substances, Center for Excellence in Molecular Synthesis, Shanghai Institute of Organic Chemistry, University of Chinese Academy of Sciences, Chinese Academy of Sciences, 200032 Shanghai, China

**Keywords:** Asymmetric catalysis, Asymmetric synthesis, Green chemistry, Synthetic chemistry methodology

## Abstract

Chiral 1,2-diamines serve as not only common structure units in bioactive molecules but also useful ligands for a range of catalytic asymmetric reactions. Here, we report a method to access *anti*-1,2-diamine derivatives. By means of the electron-withdrawing nature of 2- or 4-nitro-phenyl group, a copper(I)-catalyzed asymmetric α-addition of ketimines derived from trifluoroacetophenone and 2- or 4-NO_2_-benzylamines to aldimines is achieved, which affords a series of chiral *anti*-1,2-diamine derivatives in moderate to high yields with moderate to high diastereoselectivity and high to excellent enantioselectivity. Aromatic aldimines, heteroaromatic aldimines, and aliphatic aldimines serve as suitable substrates. The nitro group is demonstrated as a synthetical handle by several transformations, including a particularly interesting Fe(acac)_3_-catalyzed radical hydroamination with a trisubstituted olefin. Moreover, the aryl amine moiety obtained by the reduction of the nitro group serves as a synthetically versatile group, which leads to the generation of several functional groups by the powerful Sandmeyer reaction, such as -OH, -Br, -CF_3_, and -BPin.

## Abstract (web summary)

Chiral 1,2-diamines are useful chemicals used as ligands in asymmetric catalysis. Here, the authors report a copper(I)-catalyzed asymmetric α-addition of ketimines derived from trifluoroacetophenone and 2- or 4-NO2-benzylamines to aldimines, affording a series of chiral anti-1,2-diamine derivatives.

## Introduction

**Fig. 1:**
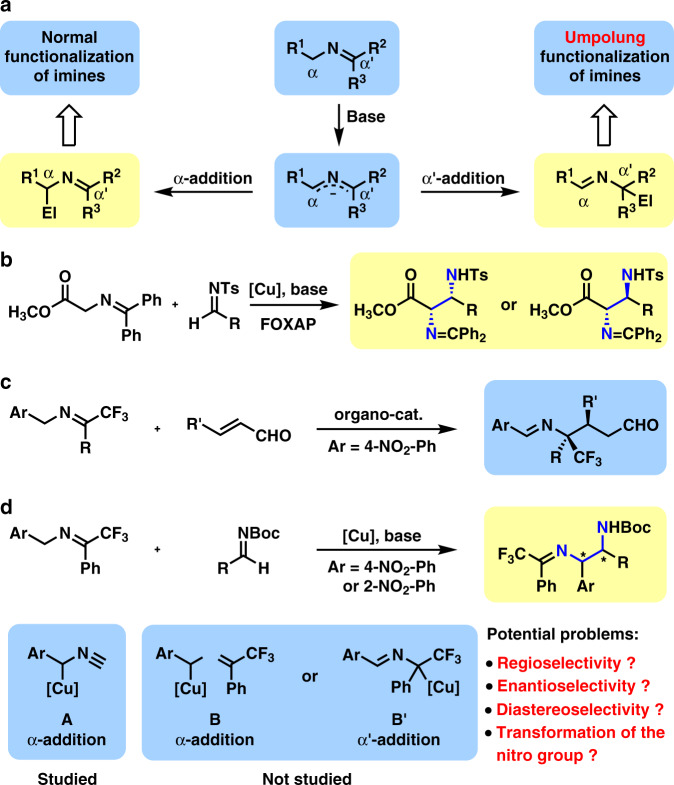
Prior arts and our work in the catalytic asymmetric reactions with 2-azaallyl anions. **a** Regioselectivity in the nucleophilic addition of 2-azaallyl anions. **b** Reported catalytic asymmetric α-addition by Hou and Wu. **c** Reported catalytic asymmetric α′-addition (umpolung addition) by Deng. **d** Our work: synthesis of 1,2-diamines through copper(I)-catalyzed α-addition.

Chiral 1,2-diamines have been identified as one of the most important structural motifs in bioactive natural products and pharmaceutical compounds^1–4^. Moreover, they served as both powerful ligands in transition metal-catalyzed asymmetric reaction and efficient chiral organocatalysts^5–8^. Therefore, various methods have been developed for their asymmetric synthesis^1–4^. Reported synthetic methods could be divided into three types, transformations with preformed diamine skeleton, transformations with carbon–nitrogen bond formation, and transformations with carbon–carbon bond formation. In the third type, a carbon atom containing an *N*-based functional group, such as nitro^9^, isocyanide^10–13^, isothiocyanato^14,15^, or ketimine^16^ was usually employed as the pronucleophile in addition to aldimines. In the case of ketimine, a 2-azaallyl anion^16^ was generated upon the deprotonation with a base, which would exhibit two pathways toward electrophiles, α-addition and α′-addition (Fig. 1a).

Usually, a second functional group such as ester^17–21^, nitrile^22,23^, or phosphate^18,24^ was required to stabilize the 2-azaallyl anion (R^1^ = CO_2_R, CN, or P(O)(OR)_2_). Furthermore, the introduction of a strong electron-withdrawing group on the α-position of ketimine would lower p*K*_a_ value of the α-protons and thus enable a facile proton-transfer α-addition to aldimines, which would produce chiral 1,2-diamine derivatives in the presence of a chiral catalyst. In 2008, Hou, Wu and co-workers disclosed an interesting catalytic asymmetric Mannich reaction of *N*-(diphenylmethylene)glycine methyl ester with high enantioselectivity^17^ (Fig. 1b). By changing the electronic property on the ligand (FOXAP), both *syn*- and *anti*-1,2-diamine derivatives were synthesized in high diastereoselectivity. Moreover, Zhao group uncovered a powerful biomimetic asymmetric Mannich reaction of *tert*-butyl glycine hydrochloride via carbonyl catalysis, which afforded chiral *anti*-1,2-diamine derivatives in excellent diastereo- and enantioselectivity^21^.

Except for stabilized 2-azaallyl anion, *semi*-stabilized 2-azaallyl anion (R^1^ = aryl) and nonstabilized 2-azaallyl anion (R^1^ = H or alkyl) were also employed as nucleophiles in the α-addition to aldimines^16^. As a breakthrough in this field, Kobayashi group disclosed a KO^*t*^Bu-18-crown-6-catalyzed Mannich-type reaction of (9-fluorenylidene)aminoalkanes and Dpp-aldimines^25^. However, a catalytic asymmetric version afforded a chiral *syn*-1,2-diamine derivative in 74% ee. Although Reddy group developed a diastereoselective version by using Davis–Ellman’s imines in 2018 (ref. ^26^), a catalytic asymmetric reaction has rarely been achieved. In the same year, Malcolmson’s group reported a powerful reductive coupling of azadienes with aldimines and ketimines, which afforded *anti*-1,2-diamine derivatives in excellent diastereo- and enantioselectivities^27^. In this beautiful reaction, a catalytic amount of nonstabilized 2-azaallyl anions were generated by the reduction of azadienes with (Ph-BPE)CuH.

The α′-addition of 2-azaallyl anion is called as a umpolung addition of imine as imine usually serves as electrophile toward various nucleophiles^28^, which provides accesses to new structures and new substitution patterns^29–39^. In 2015, Deng group uncovered an admiring catalytic asymmetric umpolung reaction of ketimines derived from trifluoroacetophenone and 4-NO_2_-benzylamines and α,β-unsaturated aldehydes^29^ (Fig. 1c). In the presence of a powerful organocatalyst derived from cinchona alkaloid, the generated 2-azaallyl anions from ketimines via deprotonation favored α′-addition to α,β-unsaturated aldehydes (1,4-conjugate addition). However, α-addition of such 2-azaallyl anions has been much less achieved in asymmetric catalysis. In 2015, Shibasaki and Kumagai proposed a benzyl copper(I) species A, which reacted with aldimines to afford chiral 1,2-diarylethylenediamine derivatives through α-addtion^13^. Therefore, it is envisioned that a benzyl copper(I) species B will be generated through the deprotonation of the ketimine with a base in the presence of a copper(I) complex, which may form an equilibrium with another benzyl copper(I) species B′ (Fig. 1d). Species B will lead to α-addition, while species B′ will lead to α′-addition. Significant steric hindrances from both the bulky copper(I)–bisphosphine complex and the congested α′-position bearing Ph and CF_3_ exist in α′-addition, and thus α′-addition is disfavored. Obviously, α-addition of the benzyl copper(I) species to aldimines will produce chiral 1,2-diamine derivatives. Moreover, it is well known that the nitro group serves as a versatile functional group in organic synthesis^40^. By reducing the nitro group to amino group, Sandmeyer reaction^41^ will allow facile preparation of more chiral 1,2-diamines containing a variety of other functional groups.

Here, we show the catalytic asymmetric α-addition of ketimines derived from trifluoroacetophenone and 2- or 4-NO_2_-benzylamines to aldimines, which furnishes a series of *anti*-1,2-diamine derivatives in moderate-to-high yields with moderate-to-high diastereoselectivity and high-to-excellent enantioselectivity. The nitro group in the products is demonstrated as a synthetic handle to afford amine and nitrone. Moreover, the produced arylamine serves as an excellent substrate for synthetically powerful Sandmeyer reaction to afford arene, phenol, arylbromide, trifluoroarene, or arylboronate in good yield.

## Results

### Condition optimization

First of all, the reaction between ketimine 1a and *N*-Boc-aldimine 2a was performed in the presence of 5 mol % Cu(CH_3_CN)_4_PF_6_, 5 mol % (*R*)-BINAP, and 5 mol % Barton’s Base (Table 1, entry 1). The addition proceeded smoothly to afford product 3aa in 48% yield with 17% ee. By screening of commercially available bisphosphine ligands, (*R*,*R*_*P*_)-TANIAPHOS was found to be the best in term of enantioselectivity (entries 2–8). Lowering the reaction temperature to –30 °C resulted in 96% ee (entry 9). Other organic bases, such as TMG and Et_3_N, also worked very well to catalyze the reaction with higher yields and maintained enantioselectivity (entry 10-11). Mesitylcopper^42^-(*R*,*R*_*P*_)-TANIAPHOS was used to replace the combination of Cu(CH_3_CN)_4_PF_6_-(*R*,*R*_*P*_)-TANIAPHOS-organic base, which resulted in the same reaction efficiency (entry 12). Changing the solvent from THF to DME led to a slightly increased yield (entry 13). Other solvents (including ^*n*^octane, toluene, mesitylene, PhCF_3_, and DCM) were also tested without any better results observed (for the details, see Supplementary Table 1). The conditions shown in entry 13 were selected as the optimized reaction conditions due to the easy manipulation with mesitylcopper and the highest yield. Without mesitylcopper complex, the reaction did not proceed at all at room temperature (entry 14). Except for *N*-Boc aldimine 2a, other aldimines derivated from benzaldehyde, such as *N*-Cbz aldimine, *N*-Ts aldimine, *N-*2-thiophenesulfonyl aldimine, and *N*-P(S)Ph_2_ aldimine were also evaluated under the reaction conditions showed in entry 13. However, no superior results were obtained (for the details, see Supplementary Table 2). Both *N*-Ts aldimine and *N-*2-thiophenesulfonyl aldimine afforded the corresponding products without asymmetric induction. The reaction with *N*-Cbz aldimine and *N*-P(S)Ph_2_ aldimine led to much worse yields and enantioselectivity.

**Table 1:** Optimization of reaction conditions^a^.


Entry	Ligand	Base	*T*	Yield^b^	ee (%)^c^
1	(*R*)-BINAP	Barton’s base	RT	48	−17
2	(*R*)-Tol-BINAP	Barton’s base	RT	49	−52
3	(*R*)-SEGPHOS	Barton’s base	RT	50	−20
4	(*R*)-DTBM-SEGPHOS	Barton’s base	RT	43	−66
5	(*R*,*R*)-Ph-BPE	Barton’s base	RT	38	−21
6	(*R*,*R*)-QUINOXP*	Barton’s base	RT	55	−31
7	(*R*)-(*S*)-JOSIPHOS	Barton’s base	RT	32	0
8	(*R*,*Rp*)-TANIAPHOS	Barton’s base	RT	57	86
9	(*R*,*Rp*)-TANIAPHOS	Barton’s base	−30	61	96
10	(*R*,*Rp*)-TANIAPHOS	TMG	−30	65	96
11	(*R*,*Rp*)-TANIAPHOS	Et_3_N	−30	75	98
12^d^	(*R*,*Rp*)-TANIAPHOS	–	−30	77	97
13^d,e^	(*R*,*Rp*)-TANIAPHOS	–	−30	82	97
14^f^	(*R*,*Rp*)-TANIAPHOS	–	RT	–	–

*DME* 1,2-dimethoxyethane, *RT* room temperature.

^a^**1a**: 0.1 mmol, **2a**: 0.15 mmol.

^b^Determined by ^1^H NMR analysis of reaction crude mixture using CH_3_NO_2_ as an internal standard.

^c^Determined by chiral-stationary-phase HPLC analysis.

^d^In total, 5 mol % mesitylcopper and 5 mol % ligand were used.

^e^DME was employed instead of THF.

^f^The reaction was performed without mesitylcopper and ligand.

### Substrate scope

**Fig. 2:**
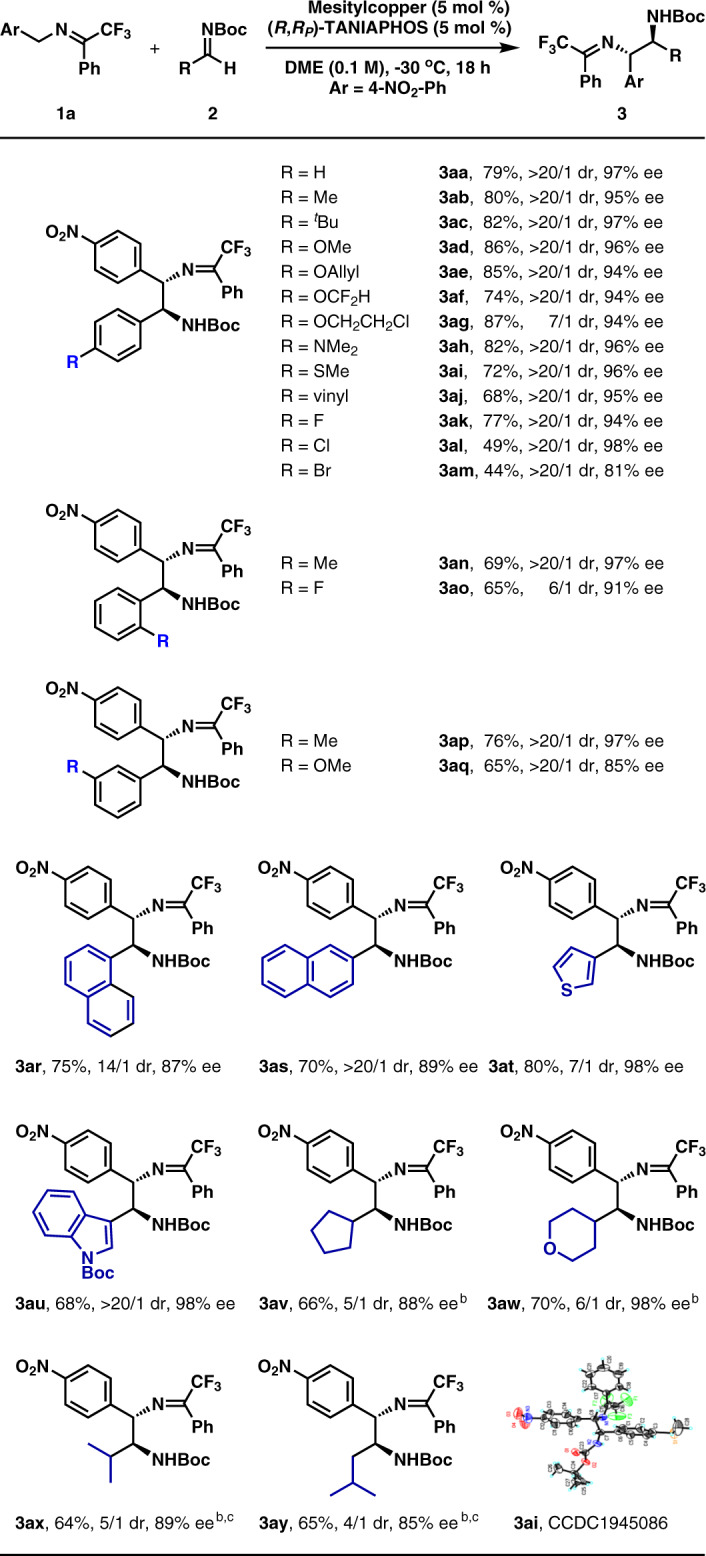
Substrate scope of aldimines 2^a^. Both aromatic aldimines and aliphatic aldimines are employed. ^a^**1a**: 0.2 mmol, **2**: 0.3 mmol. Isolated yield. Dr determined by ^1^H NMR analysis of the crude reaction mixture. Enantioselectivity determined by chiral-stationary-phase HPLC analysis. ^b^4 equiv **2**. ^c^The reaction is performed with 10 mol % mesitylcopper and 10 mol % (*R*,*R*_*P*_)-TANIAPHOS in THF at RT for 8 h.

Under the optimized reaction conditions, the substrate scope of aldimines was investigated (Fig. 2). As for the aromatic aldimines containing a *para*-substituent, both electron-donating groups and electro-withdrawing groups were well tolerated with the corresponding products generated in 7/1 to >20/1 dr and 81% to 98% ee (3aa–3am). However, aromatic aldimines with electron-withdrawing groups afforded lower yields. Both the aromatic aldimines containing an *ortho*-substituent and the aromatic aldimines containing a *meta*-substituent were competent substrates too (3an–3aq). Moreover, both 1-naphthyl aldimine and 2-naphthyl aldimine were well applicable to the present reaction protocol (3ar–3as). The reactions of two heteroaromatic aldimines, containing 2-thienyl and *N*-Boc-3-indolyl, also worked very well (3at–3au). It should be mentioned that the diastereoselectivity was moderate in several cases (3ag, 3ao, and 3at).

Then, aliphatic aldimines were examined. Aldimines with a cycloalkyl substituent were found as satisfactory substrates too (3av–3aw). In the cases of α-branched aldimine (2x) and β-branched aldimine (2y), the reactions were performed in THF at room temperature in the presence of 10 mol % mesitylcopper and 10 mol % (*R*,*R*_*P*_)-TANIAPHOS. Products (3ax–3ay) were obtained in moderate yields with moderate diastereo- and high enantioselectivities. The diastereoselectivity in these cases was moderate possibly due to the smaller differences of both steric hindrance and electronic nature between an alkyl and the hydrogen atom. The absolute configurations of the two stereogenic carbon centers in 3ai were determined by X-ray analysis of its single crystals. The absolute configurations of other products were deduced by analogy.

**Fig. 3:**
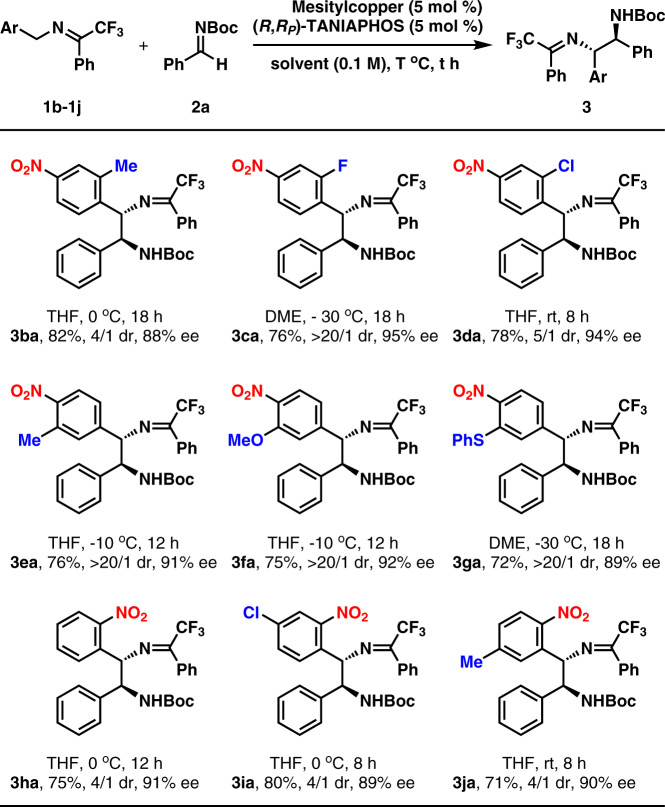
Substrate scope of ketimines 1^a^. Ketimines derived from both 4-nitro-benzylamines and 2-nitro-benzylamines are employed. ^a^1b-1j: 0.2 mmol, 2a: 0.3 mmol. Isolated yield. Dr determined by ^1^H NMR analysis of the crude reaction mixture. Enantioselectivity determined by chiral-stationary-phase HPLC analysis.

A simple study of ketimines was performed, as shown in Fig. 3. In order to get better results, the reaction conditions (including reaction temperature, and solvent) were slightly changed. Generally speaking, lower reaction temperature favors higher enantioselectivity, while higher reaction temperature favors higher yield. In many cases, THF was a better solvent than DME, which led to higher enantioselectivity at higher reaction temperature than −30 °C. As shown in Fig. 3, as for 4-nitrobenzyl imines, a substituent, such as methyl, fluorine, and chlorine was successfully tolerated at the 2-positon (3ba, 3ca, and 3da). However, with the increasing of steric hindrance of the substituent, the diastereoselectivity decreased. Furthermore, a substituent, such as methyl, methoxyl, and thiophenyl, could be introduced at the 3-position with high diastereo- and enantioselectivities observed (3ea, 3fa, and 3ga). At last, 2-nitrobenzyl imine 1 h was investigated, which was transformed to product 3 ha in 75% yield with 4/1 dr and 91% ee. Similar reaction efficiency was observed in the reactions of imines 3i and 3j. It should be pointed out that the reactions of 2-nitrobenzyl imines proceeded in moderate diastereoselectivity. The absolute configurations of 3ba–3ja were assumed according to the stereo-structure of 3ai. Except for the nitro group, other electron-withdrawing groups, such as 4-COOMe, 4-CF_3_, 4-P(O)Ph_2_, 4-CN, and 4-SO_2_CF_3_ were also introduced into the ketimines. However, even at room temperature, the reaction with 4-COOMe-Ph, 4-CF_3_-Ph, 4-P(O)Ph_2_-Ph, and 4-CN-Ph proceeded in unsatisfactory results, indicating less efficient deprotonation process. The ketimine-bearing 4-SO_2_CF_3_ was a suitable substrate, which reacted with aldimine 2a to give the corresponding product in 53% yield with 91% ee (for the details, see Supplementary Table 3).

### Gram-scale reaction and transformation

**Fig. 4:**
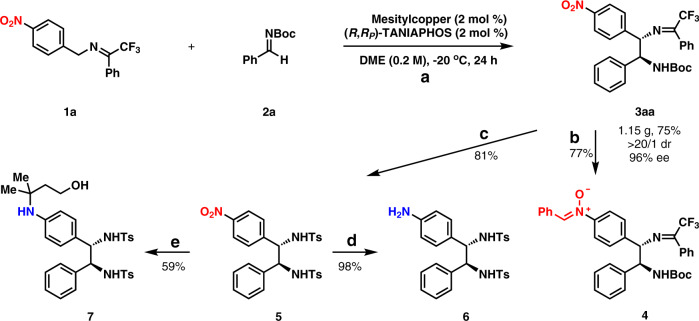
Gram-scale reaction and transformations of the nitro group. **a** Gram-scale reaction. **b** Transformation of the nitro group to the nitrone group. Zn (6.0 equiv), HOAc (12.0 equiv), EtOH, 0 °C to RT. **c** Removal of the ketimine moiety and protection of the newly generated free amine moiety. (1) 12 M HCl, MeOH, RT; (2) TsCl, Et_3_N, DMAP, DCM, RT. **d** Reduction of the nitro group to the amine group. Pd/C, H_2_ (1 atm), MeOH, RT. **e** Radical hydroamination. (1) 3-methylbut-3-en-1-ol (3.0 equiv), Fe(acac)_3_ (30 mol %), PhSiH_3_, EtOH, 60 °C; (2) Zn, HCl, 60 °C.

A gram-scale preparation of 3aa was achieved successfully with 2 mol % mesitylcopper and 2 mol % (*R*,*R*_*P*_)-TANIAPHOS at −20 °C as shown in Fig. 4, which afforded 1.15 gram 3aa in 75% yield with >20/1 dr and 96% ee. The transformation of the nitro group to the nitrone group was easily achieved without touching both the imine moiety and the *N*-Boc moiety. Nitrone 4 was generated in 77% yield by following a reported reaction procedure^43^, which potentially might serve as a versatile intermediate in organic synthesis. The cleavage of both C = N moiety and *N*-Boc moiety was carried out in 12 M HCl in MeOH to give the desired *anti*-1,2-diamine, which was protected with TsCl to deliver 5 in 81% yield for two steps. The nitro group in 5 was easily reduced to an amine group (6) in 98% yield in the presence of Pd/C under 1 atm H_2_. Interestingly, 5 underwent a Fe(acac)_3_-catalyzed radical hydroamination with 3-methylbut-3-en-1-ol to furnish α-tertiary amine 7 in 59% yield^44^.

**Fig. 5:**
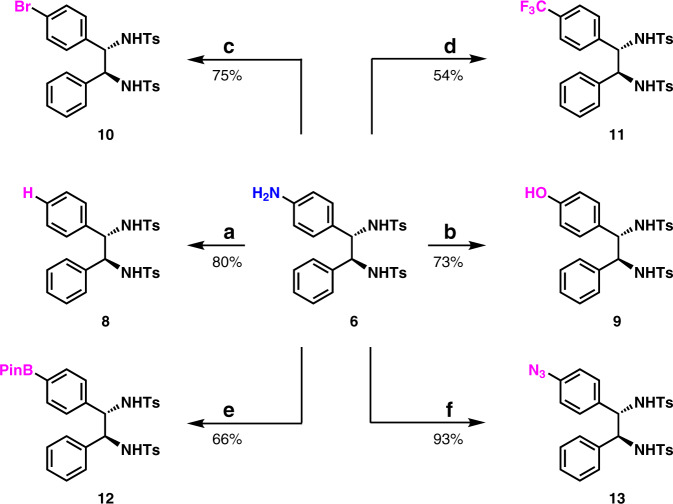
Synthetic applications. **a** Removal of the amine moiety. NaNO_2_, HCl, H_3_PO_2_, H_2_O, 0 °C. **b** transformation of the arylamine to phenol. NaNO_2_, H_2_SO_4_, H_2_O, 0 to 100 °C. **c** transformation of the arylamine to arylbromide. CuBr_2_ (1 mol %), ^*t*^BuONO, TsOH, TBAB, MeCN, RT. **d** transformation of the arylamine to trifluoromethyl arene. ^*t*^BuONO, HCl, AgCF_3_, MeCN, −40 °C. **e** Transformation of the arylamine to arylBPin. ^*t*^BuONO, B_2_Pin_2_, MeCN, 80 °C. **f** Transformation of the arylamine to aryl azide. KHCO_3_, FSO_2_N_3_, MTBE/DMF/H_2_O, RT.

Transformations of aromatic amine 6 were performed by means of synthetically powerful Sandmeyer reaction^41^ (Fig. 5). The removal of the NH_2_ group was carried out by means of the traditional Sandmeyer reaction conditions, which delivered 1,2-diaryldiamine 8 in 80% yield. The conversion of arylamine 6 to phenol 9 was achieved in 73% yield. The preparation of arylbromide 10 was also accomplished in 75% yield. The deaminative trifluoromethylation was carried out to give 11 in 54% yield by following a newly reported procedure^45^. The conversion of arylamine 6 to arylboronate 12 was achieved in 66% yield^46^. More interestingly, by following a powerful reaction^47^, aryl azide 13 was synthesized from 6 in 93% yield. Evidently, compounds 9, 10, 12, and 13 would allow further easy structure elaboration.

## Discussion

In conclusion, we developed a copper(I)-catalyzed asymmetric α-addition of ketimines derived from trifluoroacetophenone and 2- or 4-NO_2_-benzylamines to *N*-Boc-aldimines. A series of chiral *anti*-1,2-diamine derivatives were generated in moderate-to-high yields with moderate-to-high diastereoselectivity and high-to-excellent enantioselectivity. The reaction enjoyed a broad substrate scope of aldimines. The nitro group in the products served as a synthetic handle to facilitate further structure elaboration. Furthermore, the arylamine generated by the reduction of the nitro group was an excellent substrate for the synthetically powerful Sandmeyer reaction, which was transformed to arene, phenol, arylbromide, trifluoroarene, and arylboronate. More interestingly, the preparation of aryl azide was efficiently accomplished by means of a powerful reaction protocol. The capture of the benzyl copper(I) species and its further application in asymmetric catalysis are currently ongoing in our laboratory.

## Methods

### A general procedure for aromatic aldimines

A dried 25-mL schlenk tube equipped with a magnetic stirring bar was charged with mesitylcopper (1.8 mg, 0.01 mmol, 5.0 mol %) and (*R*,*R*p)-TANIAPHOS (6.8 mg, 0.01 mmol, 5.0 mol %) in a glove box under Ar atmosphere. Anhydrous DME (2 mL) was added via a syringe. The mixture was stirred for 20 min at room temperature to give a yellow solution. The reaction mixture was cooled to −30 °C, and benzyl imine (0. 2 mmol, 1.0 equiv) and *N*-Boc-aldimine (0.3 mmol, 1.5 equiv) were added. The resulting reaction mixture was stirred at −30 °C for 18 h. Then, the reaction mixture was purified by silica gel column chromatography (petroleum ether/ethyl acetate = 12/1) to give the desired product.

### A general procedure for aliphatic aldimines

A dried 25-mL schlenk tube equipped with a magnetic stirring bar was charged with mesitylcopper (3.6 mg, 0.02 mmol, 10 mol %) and (*R*,*R*p)-TANIAPHOS (13.6 mg, 0.02 mmol, 10 mol %) in a glove box under Ar atmosphere. Anhydrous THF (2 mL) was added via a syringe. The mixture was stirred for 20 min at room temperature to give a yellow solution. Then benzyl imine (0.2 mmol, 1.0 equiv) and *N*-Boc-aldimine (0.8 mmol, 4.0 equiv) were added. The resulting reaction mixture was stirred at room temperature for 8 h. Then, the reaction mixture was purified by silica gel column chromatography (petroleum ether/ethyl acetate = 12/1) to give the desired product.

## Supplementary information

Supplementary Information

## Data Availability

The X-ray crystallographic coordinates for structures reported in this study have been deposited at the Cambridge Crystallographic Data Centre (CCDC), under deposition number CCDC 1945086 (3ai) [www.ccdc.cam.ac.uk/data_request/cif]. The data supporting the findings of this study are available within the article and its Supplementary Information file. Any further relevant data are available from the authors on request.
