# Supplementary material for: Synthesis of chiral anti-1,2-diamine derivatives through copper(I)-catalyzed asymmetric α-addition of ketimines to aldimines
Source: Nat Commun. 2020 Sep 8;11:4473. doi: 10.1038/s41467-020-18235-9 (PMC7479099; doi:10.1038/s41467-020-18235-9)
Supplement: Supplementary file 1 — Supplementary Information [file 41467_2020_18235_MOESM1_ESM.pdf]

Supplementary Information

Synthesis of Chiral *anti*-1,2-Diamine Derivatives through Copper(I)-Catalyzed Asymmetric α-Addition of ketimines to Aldimines

Gan et al.

Supplementary Methods

General Information

All reagents were obtained commercially unless otherwise noted. Anhydrous tetrahydrofuran (Energy Chemical Co., Ltd.) was used without further purification. Nuclear Magnetic Resonance (NMR) spectra were acquired on an Bruker 400 (500) instrument operating at 400 (500) MHz, 100 (125) MHz and 376 (471) MHz for ^{1}H, ^{13}C and ^{19}F, respectively. For ^{1}H NMR, chemical shifts were reported in δ ppm referenced to an internal $SiMe_4$ standard. For ^{13}C NMR, chemical shifts were reported in the scale relative to NMR solvent ($CDCl_3$: δ 77.0 ppm) as an internal reference. For ^{19}F NMR, chemical shifts were reported in the scale relative to TFA as an external reference (δ = -76.5 ppm). Multiplicities are reported using the following abbreviations: s = singlet, d = doublet, t = triplet, q = quartet, m = multiplet, br = broad signal. High-resolution mass spectra (ESI) were measured on Thermo Scientific LTQ FT Ultra FT-MS. Infrared (IR) spectra were recorded on Thermo Scientific Nicolet iS5 FT-IR. Optical rotation was measured using a 1 mL cell with a 1.0 dm path length on a JASCO P-1030 polarimeter. HPLC analysis was conducted on a Shimadzu HPLC system equipped with Daicel chiral-stationary-phase columns (ϕ4.6 mm × 250 mm).

Preparation of ketimines 1b-1j

According to a reported procedure[1], to the mixture of benzyl amine (5.0 mmol, 1.0 equiv) and acetic acid (5.0 mmol, 1.0 equiv) in 5 mL $CHCl_3$ was added the solution of trifluoromethyl phenyl ketone (5.0 mmol, 1.0 equiv) in 1 mL $CHCl_3$ in one portion. The resulting mixture was refluxed until all the ketone was consumed (indicated by TLC or by the disappearance of the insoluble solid). After cooling down to room temperature, 20 mL CH_2Cl_2 was added and the mixture was washed with saturated aqueous 10 mL $NaHCO_3$. The aqueous layer was extracted with CH_2Cl_2 (20 mL × 2). The organic phases were combined, washed with brine, dried over anhydrous Na_2SO_4 and concentrated. The yellow residue was applied to silica gel chromatography (PE/EA = 50/1) to afford the benzyl imine.

(*E*)-2,2,2-trifluoro-*N*-(2-methyl-4-nitrobenzyl)-1-phenylethan-1-imine (**1b**)

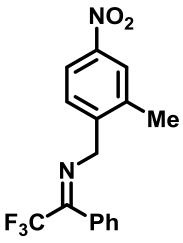

1b

white solid, 638 mg (39% yield);
^{1}H NMR (400 MHz, $CDCl_3$) δ 8.05-7.98 (m, 2H), 7.56-7.49 (m, 3H), 7.44 (d, *J* = 8.2 Hz, 1H), 7.32-7.27 (m, 2H), 4.62 (s, 2H), 2.29 (s, 3H); **^{13}C NMR (125 MHz, $CDCl_3$)** δ 160.2 (q, *J* = 37 Hz), 146.9, 143.7, 137.5, 130.6, 129.8, 129.1, 128.3, 127.3, 124.8, 121.2, 119.5 (q, *J* = 278 Hz), 54.16, 19.11; **^{19}F NMR (471 MHz, $CDCl_3$)** δ -70.82; **HRMS (ESI) m/z $[M-H]^-$:** calcd. 321.0856, found. 321.0851; **IR (film):** ν_{max} (cm^{-1}) 1670, 1590, 1520, 1348, 1197, 1132, 944, 815, 771, 740, 703;

(*E*)-2,2,2-trifluoro-N-(2-fluoro-4-nitrobenzyl)-1-phenylethan-1-imine (**1c**)

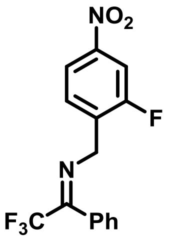

1c

white solid, 625 mg (40% yield);
^{1}H NMR (400 MHz, $CDCl_3$) δ 8.07 (d, *J* = 8.5 Hz, 1H), 7.93-7.88 (m, 1H), 7.69 (t, *J* = 8.0 Hz, 1H), 7.57-7.50 (m, 3H), 7.33-7.28 (m, 2H), 4.68 (s, 2H); **^{13}C NMR (125 MHz, $CDCl_3$)** δ 161.2 (q, *J* = 34 Hz), 159.6 (d, *J* = 250 Hz), 147.9 (d, *J* = 8.8 Hz), 133.0 (d, *J* = 13.8 Hz), 130.7, 130.0 (d, *J* = 5.0 Hz), 129.7, 129.2, 127.3, 119.50 (d, *J* = 3.8 Hz), 119.47 (q, *J* = 278 Hz), 111.0 (d, *J* = 25 Hz), 49.9 (d, *J* = 2.9 Hz); **^{19}F NMR (471 MHz, $CDCl_3$)** δ -70.97, -114.14; **HRMS (ESI) m/z $[M+H]^+$:** calcd. 327.0751, found. 327.0752; **IR (film):** ν_{max} (cm^{-1}) 1671, 1530, 1425, 1352, 1275, 1198, 1133, 1071, 941, 917, 844, 750, 702;

(*E*)-*N*-(2-chloro-4-nitrobenzyl)-2,2,2-trifluoro-1-phenylethan-1-imine (**1d**)

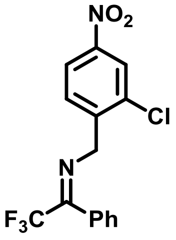

1d

white solid, 737 mg (43% yield);
^{1}H NMR (400 MHz, $CDCl_3$) δ 8.23-8.19 (m, 1H), 8.18-8.12 (m, 1H), 7.75 (d, *J* = 8.5 Hz, 1H), 7.58-7.49 (m, 3H), 7.35-7.28 (m, 2H), 4.71 (s, 2H); **^{13}C NMR (125 MHz, $CDCl_3$)** δ161.0 (q, *J* = 34 Hz), 147.3, 143.2, 133.6, 130.7, 129.6, 129.5, 129.1, 127.3, 124.3, 121.9, 119.5 (q, *J* = 276 Hz), 53.6.; **^{19}F NMR (471 MHz, $CDCl_3$)** δ -70.79; **HRMS (ESI) m/z $[M+H]^+$:** calcd. 343.0456, found. 343.0453; **IR (film):** ν_{max} (cm^{-1}) 1672, 1592, 1525, 1347, 1262, 1197, 943, 893, 809, 738, 701;

(*E*)-2,2,2-trifluoro-*N*-(3-methyl-4-nitrobenzyl)-1-phenylethan-1-imine (**1e**)

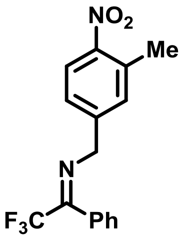

1e

white solid, 709 mg (44% yield);

^{1}H NMR (400 MHz, $CDCl_3$) δ 7.99-7.93(m, 1H), 7.58-7.48 (m, 3H), 7.31-7.22 (m, 4H), 4.63 (s, 2H), 2.60 (s, 3H); **^{13}C NMR (100 MHz, $CDCl_3$)** δ 160.0 (q, *J* = 33 Hz), 134.1, 131.6, 130.5, 129.8, 129.1, 127.4, 125.8, 125.1, 119.5 (q, *J* = 277 Hz), 55.8, 20.6; **^{19}F NMR (376 MHz, $CDCl_3$)** δ -70.95; **HRMS (ESI) m/z $[M-H]^-$:** calcd. 321.0856, found. 321.0851; **IR (film):** ν_{max} (cm^{-1}) 1670, 1612, 1589, 1445, 1522, 1343, 1197, 1132, 1039, 946, 836, 747, 703;

(*E*)-2,2,2-trifluoro-N-(3-methoxy-4-nitrobenzyl)-1-phenylethan-1-imine (**1f**)

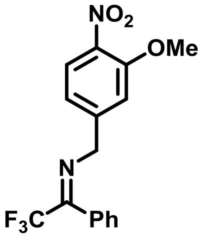

1f

white solid, 778 mg (46% yield);

^{1}H NMR (400 MHz, $CDCl_3$) δ 7.84 (d, *J* = 8.3 Hz, 1H), 7.58-7.48 (m, 3H), 7.29-7.27 (m, 2H), 7.11 (s, 1H), 6.90 (d, *J* = 8.3 Hz, 1H), 4.63 (s, 2H), 3.96 (s, 3H); **^{13}C NMR (125 MHz, $CDCl_3$)** δ160.2 (q, *J* = 34 Hz), 153.4, 145.4, 138.4, 130.6, 129.9, 129.1, 127.4, 126.1, 119.5 (q, *J* = 278 Hz), 118.9, 112.4, 56.5, 55.9; **^{19}F NMR (471 MHz, $CDCl_3$)** δ -70.96; **HRMS (ESI) m/z $[M+H]^+$:** calcd. 339.0951, found. 339.0951; **IR (film):** ν_{max} (cm^{-1}) 1669, 1609, 1520, 1457, 1331, 1266, 1196, 1131, 944, 840, 702, 682;

(*E*)-2,2,2-trifluoro-N-(4-nitro-3-(phenylthio)benzyl)-1-phenylethan-1-imine (**1g**)

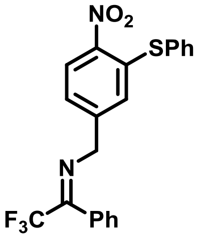

1g

yellow solid, 874 mg (42% yield);

^{1}H NMR (400 MHz, $CDCl_3$) δ 8.20 (d, *J* = 8.5 Hz, 1H), 7.62-7.56 (m, 2H), 7.54-7.41 (m, 6H), 7.10-7.03 (m, 3H), 6.77 (s, 1H), 4.44 (s, 2H); **^{13}C NMR (125 MHz, $CDCl_3$)** δ 160.2 (q, *J* = 34 Hz), 144.2, 143.7, 140.3, 135.9, 130.6, 130.5, 130.2, 130.1, 129.6, 129.0, 127.3, 126.6, 126.0, 123.9, 119.3 (q, *J* = 276 Hz), 55.50; **^{19}F NMR (471 MHz, $CDCl_3$)** δ -70.96; **HRMS (ESI) m/z $[M+H]^+$:** calcd. 471.0879, found. 471.0880; **IR (film):** ν_{max} (cm^{-1}) 1669, 1595, 1575, 1506, 1334, 1195, 1131, 948, 837, 825, 771, 750, 705;

(*E*)-*N*-(4-chloro-2-nitrobenzyl)-2,2,2-trifluoro-1-phenylethan-1-imine (**1i**)

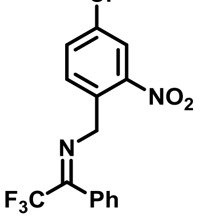

1i

yellow solid, 684 mg (40% yield);

^{1}H NMR (400 MHz, $CDCl_3$) δ 8.03 (d, *J* = 2.1 Hz, 1H), 7.71 (d, *J* = 8.4 Hz, 1H), 7.62 (dd, *J* = 8.4, 2.1 Hz, 1H), 7.56-7.48 (m, 3H), 7.35-7.30 (m, 2H), 4.88 (s, 2H); **^{13}C NMR (125 MHz, $CDCl_3$)** δ 160.47 (q, *J* = 35 Hz), 148.0, 133.9, 133.7, 132.6, 131.4, 130.6, 129.7, 129.1, 127.3, 125.0, 119.5 (q, *J* = 276 Hz), 53.2; **^{19}F NMR (471 MHz, $CDCl_3$)** δ -70.76; **HRMS (ESI) m/z $[M+H]^+$:** calcd. 343.0456, found. 343.0454; **IR (film):** ν_{max} (cm^{-1}) 1672, 1537, 1351, 1259, 1200, 1131, 964, 887, 813, 702;

(*E*)-2,2,2-trifluoro-*N*-(5-methyl-2-nitrobenzyl)-1-phenylethan-1-imine (**1j**)

yellow solid, 676 mg (42% yield);

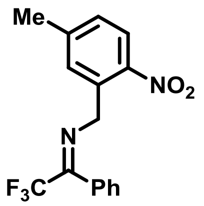

1j

^{1}H NMR (400 MHz, CDCl$_3$) δ 7.97 (d, *J* = 8.3 Hz, 1H), 7.53-7.49 (m, 3H), 7.47 (s, 1H), 7.37-7.31 (m, 2H), 7.22 (d, *J* = 8.3 Hz, 1H), 4.91 (s, 2H), 2.46 (s, 3H); **^{13}C NMR (125 MHz, CDCl$_3$)** δ 160.0 (q, *J* = 34 Hz), 145.5, 145.1, 134.0, 130.6, 130.5, 129.9, 129.0, 128.6, 127.4, 125.2, 119.6 (q, *J* = 277 Hz), 54.0, 21.6; **^{19}F NMR (471 MHz, CDCl$_3$)** δ -70.69; **HRMS (ESI) m/z [M+H]$^+$:** calcd. 323.1002, found. 323.0997; **IR (film):** ν_{max} (cm^{-1}) 1669, 1612, 1589, 1520, 1344, 1197, 1132, 1022, 969, 836, 776, 748;

Additional optimization of the reaction conditions

The non-coordinating solvents in the reaction of ketimine **1a** with aldimine **2a** was investigated (**Supplementary Table 1**).

Supplementary Table 1. Optimization of non-coordinating solvents.

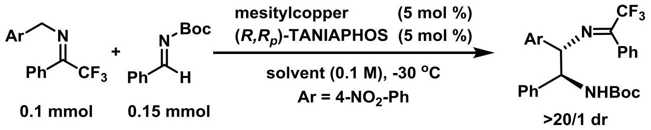

entry	solvent	yield (%)^a	ee (%)^b
1	toluene	45	76
2	*n*-octane	36	64
3	DCM	57	98
4	mesitylene	39	74
5	$PhCF_3$	75	97

^{a}Determined by ^{1}H NMR analysis of reaction crude mixture. ^{b}Determined by chiral-stationary-phase HPLC analysis.

Except for *N*-Boc aldimine **2a**, other aldimines derivated from benzaldehyde, such as *N*-Cbz aldimine, *N*-Ts aldimine, *N*-2-thiophenesulfonyl aldimine, and *N*-P(S)Ph$_2$ aldimine were also investigated (**Supplementary Table 2**).

Supplementary Table 2. Optimization of other aldimines derivated from benzaldehyde.

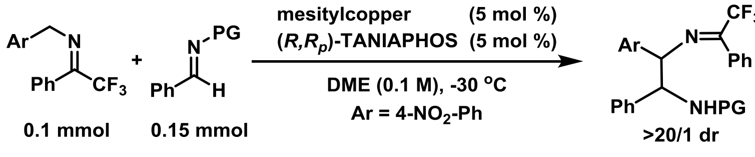

entry	PG	yield (%)^a	ee (%)^b
1	Cbz	45	78
2	Ts	49	0
3	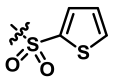	91	0
4	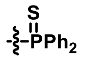	12	27

^{a}Determined by ^{1}H NMR analysis of reaction crude mixture. ^{b}Determined by chiral-stationary-phase HPLC analysis.

General procedure for copper(I)-catalyzed asymmetric α-Addition of ketimines to Aldimines

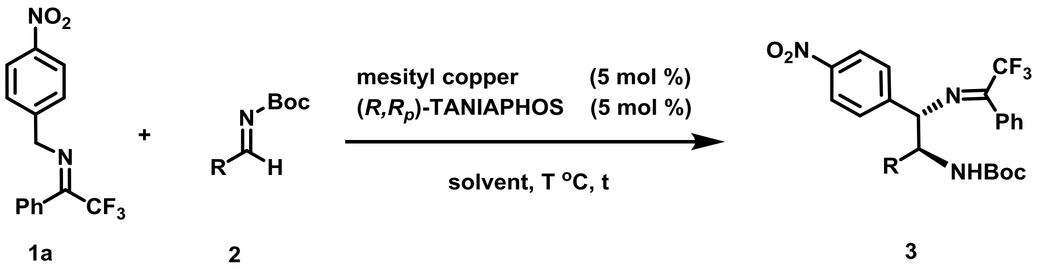

Procedure A

A dried 25 mL schlenk tube equipped with a magnetic stirring bar was charged with mesityl copper (1.8 mg, 0.01 mmol, 5.0 mol %) and (R,R_p)-TANIAPHOS (6.8 mg, 0.01 mmol, 5.0 mol %) in a glove box under Ar atmosphere. Anhydrous DME (2 mL) was added via a syringe. The mixture was stirred for 20 minutes to give a yellow solution. The reaction mixture was cooled to -30 ℃, and then benzyl imine (0. 2 mmol, 1.0 equiv) and *N*-Boc-aldimine (0.3 mmol, 1.5 equiv) were added. The resulting reaction mixture was stirred at -30 ℃ for 18 hours. Then, the reaction mixture was purified by silica gel column chromatography (petroleum ether/ethyl acetate = 12/1) to give the product.

Procedure B

A dried 25 mL schlenk tube equipped with a magnetic stirring bar was charged with mesityl copper (3.6 mg, 0.02 mmol, 10 mol %) and (R,R_p)-TANIAPHOS (13.6 mg, 0.02 mmol, 10 mol %) in a glove box under Ar atmosphere. Anhydrous THF (2 mL) was added via a syringe. The mixture was stirred for 20 minutes to give a yellow solution. Then benzyl imine (0.2 mmol, 1.0 equiv) and *N*-Boc-aldimine (0.8 mmol, 4.0 equiv) were added. The resulting reaction mixture was stirred at room temperature for 8 hours. Then, the reaction mixture was purified by silica gel column chromatography (petroleum ether/ethyl acetate = 12/1) to give the product.

***tert*-butyl ((1*S*,2*S*)-2-(4-nitrophenyl)-1-phenyl-2-(((*E*)-2,2,2-trifluoro-1-phenylethylidene)amino)ethyl)carbamate** (3aa)

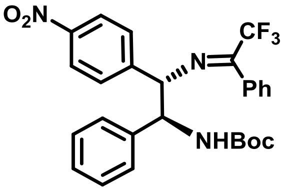

3aa

white solid, 81.1 mg (79% yield); **^{1}H NMR (400 MHz, $CDCl_3$)** δ 8.22 (d, *J* = 8.7 Hz, 2H), 7.52 (d, *J* = 8.6 Hz, 2H), 7.39-7.30 (m, 4H), 7.20 (t, *J* = 7.8 Hz, 2H), 7.13-7.06 (m, 2H), 6.31 (d, *J* = 7.5 Hz, 2H), 5.84 (d, *J* = 8.8 Hz, 1H), 5.17 (d, *J* = 8.8 Hz, 1H), 4.71 (s, 1H), 1.30 (s, 9H); **^{13}C NMR (100 MHz, $CDCl_3$)** δ 161.4 (q, *J* = 34 Hz), 155.0, 147.4, 146.6, 139.4, 130.3, 129.1, 128.6, 128.5, 127.9, 127.7, 126.7, 126.6, 123.8, 119.2 (q, *J* = 277 Hz), 80.0, 69.8, 59.6, 28.2; **^{19}F NMR (376 MHz, $CDCl_3$)** δ -71.15;

HRMS (ESI) m/z [M+H]$^+$: calcd. 514.1948, found. 514.1948;

IR (film): ν_{max} (cm^{-1}) 3438, 2968, 2925, 1712, 1601, 1524, 1494, 1347, 1199, 1047, 897, 700;

Optical Rotation: $[\alpha]_D^{25}$ = +27.3 (*c* = 1.00, $CHCl_3$, 97% ee); **HPLC:** DAICEL CHIRALPAK ID, hexane/*i*-PrOH = 95/5, flow rate: 1.0 mL/min, λ = 254 nm, t_R(minor) = 10.0 min, t_R(major) = 23.6 min, ee = 97%.

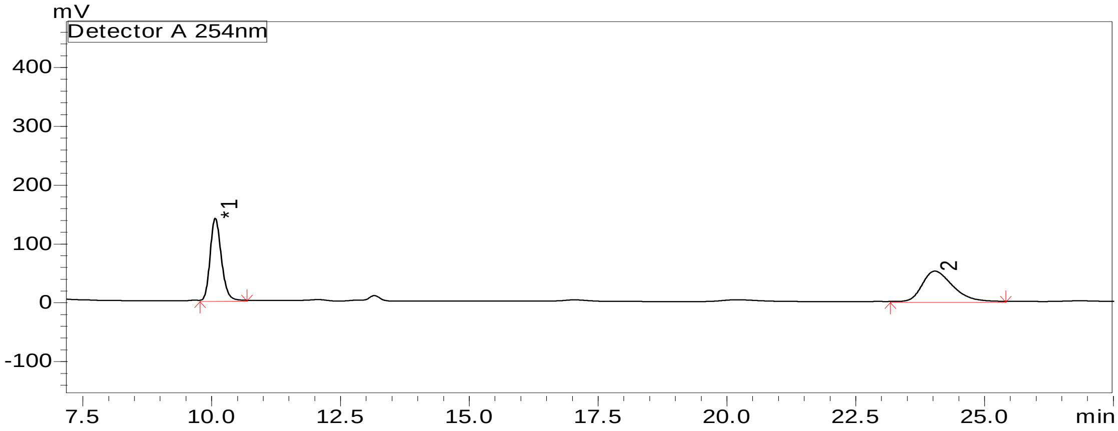

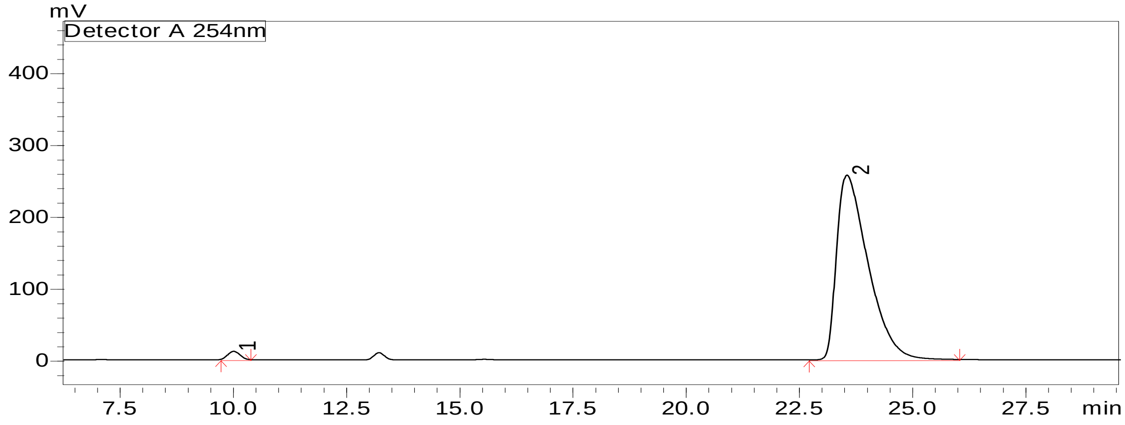

Peak#	Ret. Time	Area%
1	10.095	50.047
2	24.061	49.953

Peak#	Ret. Time	Area%
1	10.036	1.674
2	23.578	98.326

Supplementary Figure 1. HPLC chromatogram for compound **3aa**

***tert*-butyl ((1*S*,2*S*)-2-(4-nitrophenyl)-1-(*p*-tolyl)-2-(((*E*)-2,2,2-trifluoro-1-phenylethylidene)amino)ethyl)carbamate** (**3ab**)

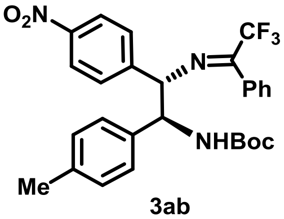

3ab

white solid, 84.4 mg (80% yield);

^{1}H NMR (400 MHz, $CDCl_3$) δ 8.21 (d, *J* = 8.5 Hz, 2H), 7.51 (d, *J* = 8.5 Hz, 2H), 7.37 (t, *J* = 7.6 Hz, 1H), 7.21 (t, *J* = 7.8 Hz, 2H), 7.13 (d, *J* = 7.8 Hz, 2H), 6.97 (d, *J* = 7.8 Hz, 2H), 6.35 (d, *J* = 7.6 Hz, 2H), 5.79 (d, *J* = 8.0 Hz, 1H), 5.12 (d, J = 8.0 Hz, 1H), 4.70 (s, 1H), 2.38 (s, 3H), 1.30 (s, 9H); **^{13}C NMR (125 MHz, $CDCl_3$)** δ 161.2 (q, *J* = 35 Hz), 155.0, 147.4, 146.8, 137.4, 136.4, 130.3, 129.2, 128.5, 127.9, 126.8, 126.5, 123.7, 119.2 (q, *J* = 276 Hz), 79.9, 69.9, 59.4, 28.2, 21.1; **^{19}F NMR (471MHz, $CDCl_3$)** δ -71.04;

HRMS (ESI) m/z $[M+H]^+$: calcd. 528.2105, found. 528.2100;

IR (film): ν_{max} (cm^{-1}) 3443, 2956, 2925, 1712, 1601, 1525, 1494, 1346, 1199, 1166, 1139, 1020, 975, 847, 704;

Optical Rotation: $[\alpha]_D^{25}$ = +37.3 (*c* = 1.00, $CHCl_3$, 95% ee); **HPLC:** DAICEL CHIRALPAK ID, hexane/*i*-PrOH = 95/5, flow rate: 1.0 mL/min, λ = 254 nm, t_R(minor) = 9.1 min, t_R(major) = 24.1 min, ee = 95%.

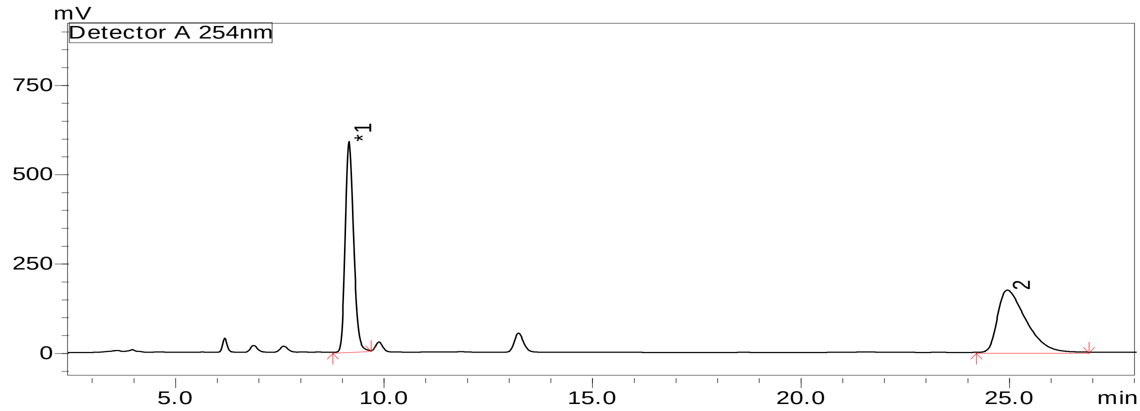

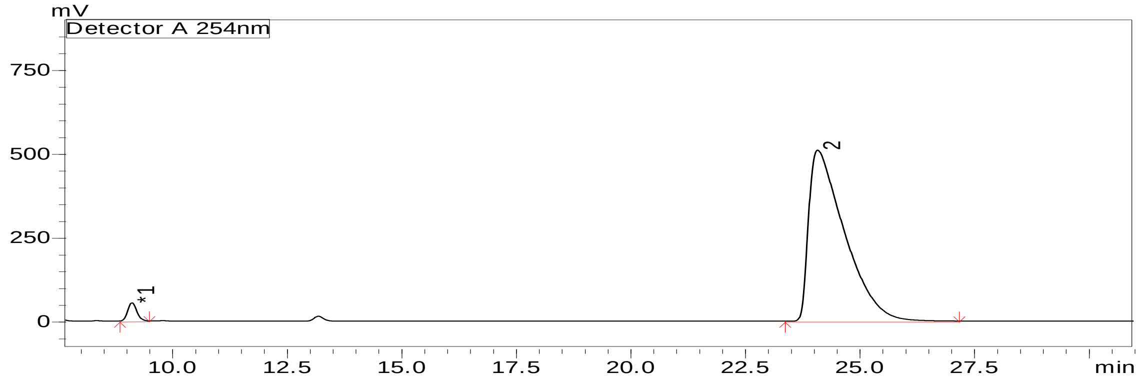

Peak#	Ret. Time	Area%
1	9.191	50.068
2	24.984	49.932

Peak#	Ret. Time	Area%
1	9.140	2.598
2	24.104	97.402

Supplementary Figure 2. HPLC chromatogram for compound **3ab**

***tert*-butyl ((1*S*,2*S*)-1-(tert-butyl)phenyl)-2-(4-nitrophenyl)-2-(((*E*)-2,2,2-trifluoro-1-phenylethylidene)amino)ethyl)carbamate** (**3ac**)

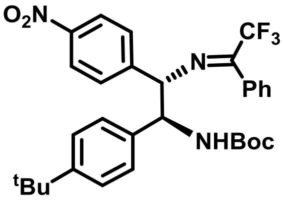

3ac

white solid, 93.4 mg (82% yield);
^{1}H NMR (400 MHz, $CDCl_3$) δ 8.22 (d, J = 8.0 Hz, 2H), 7.55 (d, J = 8.0 Hz, 2H), 7.39-7.32 (m, 3H), 7.17 (t, J = 7.5 Hz, 2H), 7.02 (d, J = 8.0 Hz, 2H), 6.23 (d, J = 7.5 Hz, 2H), 5.85 (d, J = 8.8 Hz, 1H), 5.16 (d, J = 8.8 Hz, 1H), 4.67 (s, 1H), 1.37 (s, 9H), 1.30 (s, 9H); **^{13}C NMR (125 MHz, $CDCl_3$)** δ 161.1 (q, J = 34 Hz), 155.0, 150.9, 147.4, 146.6, 136.4, 130.2, 129.1, 128.4, 127.9, 126.7, 126.3, 125.4, 123.7, 119.2 (q, J = 277 Hz), 79.9, 70.0, 59.3, 34.5, 31.4, 28.2; **^{19}F NMR (471MHz, $CDCl_3$)** δ -71.11;
HRMS (ESI) m/z [M+Na]$^+$: calcd. 592.2394, found. 592.2390;
IR (film): ν_{max} (cm^{-1}) 3447, 2962, 2928, 1716, 1600, 1525, 1495, 1347, 1224, 1199, 1168, 1140, 1017, 975, 850, 702;
Optical Rotation: $[\alpha]_D^{25}$ = +22.7 (c = 1.00, $CHCl_3$, 97% ee); **HPLC:** DAICEL CHIRALPAK ID, hexane/*i*-PrOH = 95/5, flow rate: 1.0 mL/min, λ = 254 nm, t_R(minor) = 7.9 min, t_R(major) = 9.3min, ee = 97%.

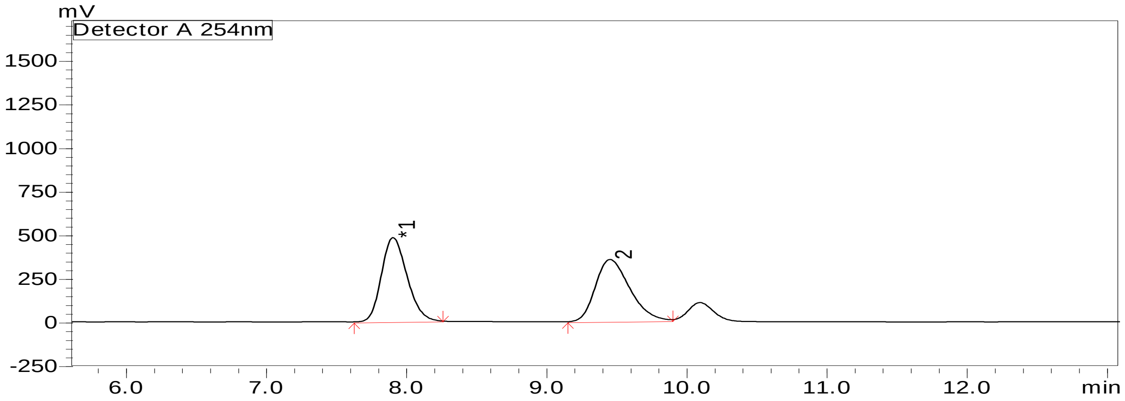

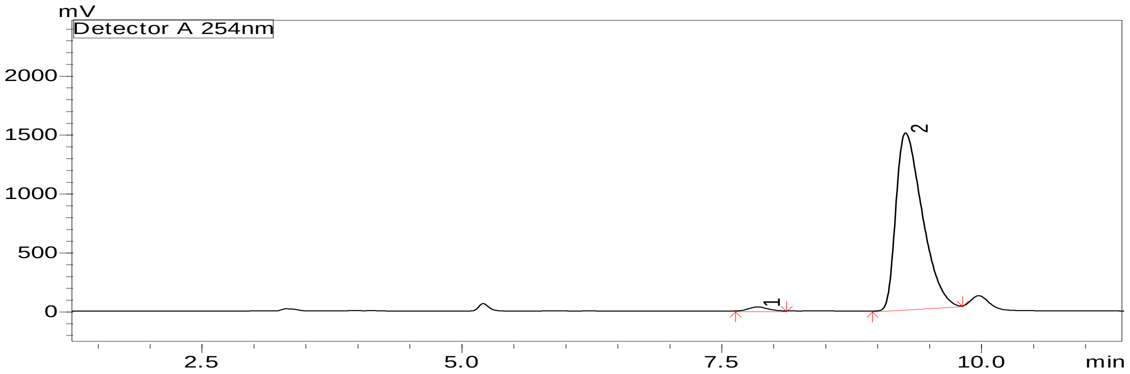

Peak#	Ret. Time	Area%
1	7.912	50.215
2	9.460	49.785

Peak#	Ret. Time	Area%
1	7.859	1.538
2	9.281	98.462

Supplementary Figure 3. HPLC chromatogram for compound **3ac**

***tert*-butyl ((1*S*,2*S*)-1-(4-methoxyphenyl)-2-(4-nitrophenyl)-2-(((*E*)-2,2,2-trifluoro-1-phenylethylidene)amino)ethyl)carbamate** (3ad)

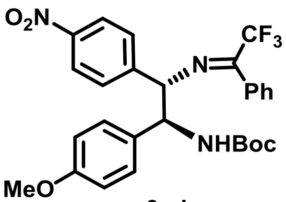

3ad

white solid, 93.4 mg (86% yield);
^{1}H NMR (400 MHz, $CDCl_3$) δ 8.21 (d, *J* = 8.7 Hz, 2H), 7.50 (d, *J* = 8.7 Hz, 2H), 7.38 (t, *J* = 7.5 Hz, 1H), 7.23 (t, *J* = 7.5 Hz, 2H), 7.00 (d, *J* = 8.6 Hz, 2H), 6.86 (d, *J* = 8.6 Hz, 2H), 6.40 (d, *J* = 7.5 Hz, 2H), 5.75 (d, *J* = 7.3 Hz, 1H), 5.11 (d, *J* = 7.3 Hz, 1H), 4.68 (s, 1H), 3.83 (s, 3H), 1.29 (s, 9H); **^{13}C NMR (125 MHz, $CDCl_3$)** δ 161.3 (q, *J* = 35 Hz), 159.1, 155.0, 147.4, 146.7, 131.6, 130.3, 129.2, 128.6, 127.9, 127.7, 126.8, 123.8, 119.5 (q, *J* = 277 Hz), 113.9, 79.9, 70.0, 59.1, 55.4, 28.2; **^{19}F NMR (376 MHz, $CDCl_3$)** δ -71.07;

HRMS (ESI) m/z $[M+H]^+$: calcd. 544.2054, found. 544.2054;

IR (film): ν_{max} (cm^{-1}) 3438, 2958, 2926, 1712, 1610, 1514, 1494, 1367, 1250, 1199, 1041, 848, 704;

Optical Rotation: $[\alpha]_D^{25}$ = +18.7 (*c* = 1.00, $CHCl_3$, 96% ee); **HPLC:** DAICEL CHIRALPAK ID, hexane/*i*-PrOH = 90/10, flow rate: 1.0 mL/min, λ = 254 nm, t_R(minor) = 9.0 min, t_R(major) = 23.6 min, ee = 96%.

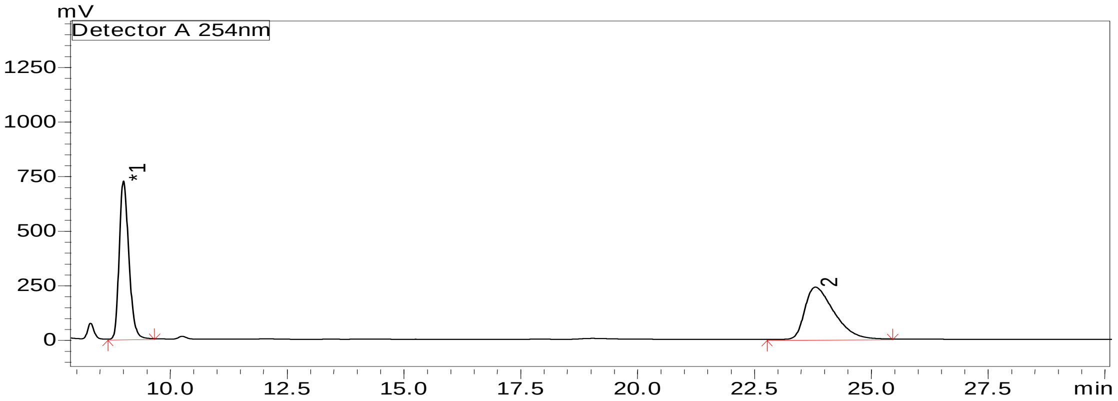

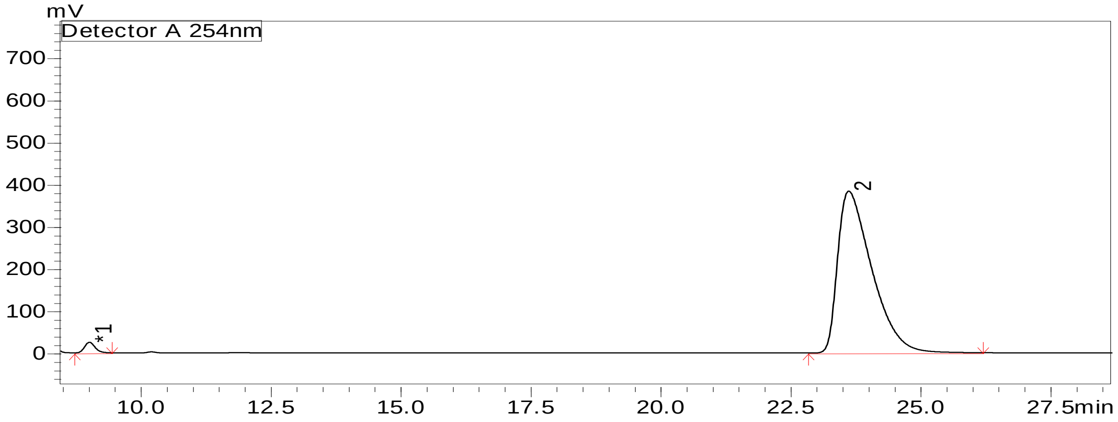

Peak#	Ret. Time	Area%
1	9.025	50.176
2	23.830	49.824

Peak#	Ret. Time	Area%
1	9.030	1.958
2	23.636	98.042

Supplementary Figure 4. HPLC chromatogram for compound **3ad**

***tert*-butyl ((1*S*,2*S*)-1-(4-(allyloxy)phenyl)-2-(4-nitrophenyl)-2-(((*E*)-2,2,2-trifluoro-1-phenylethylidene)amino)ethyl)carbamate** (**3ae**)

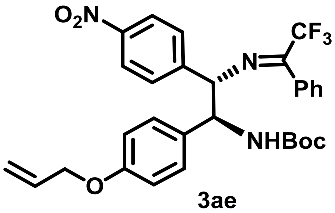

3ae

white solid, 96.8 mg (85% yield); **^{1}H NMR (400 MHz, CDCl$_3$)** δ 8.21 (d, *J* = 8.7 Hz, 2H), 7.50 (d, *J* = 8.7 Hz, 2H), 7.37 (t, *J* = 7.5 Hz, 1H), 7.23 (t, J = 7.5 Hz, 2H), 7.00 (d, *J* = 8.6 Hz, 2H), 6.88 (d, *J* = 8.6 Hz, 2H), 6.40 (d, *J* = 7.6 Hz, 2H), 6.14-6.01 (m, 1H), 5.77 (d, *J* = 8.6 Hz, 1H), 5.44 (dd, *J* = 16.0, 1.4 Hz, 1H), 5.32 (dd, *J* = 10.5, 1.4 Hz, 1H), 5.11 (d, *J* = 8.0 Hz, 1H), 4.69 (s, 1H), 4.62-4.52 (m, 2H), 1.26 (m, 9H); **^{13}C NMR (125 MHz, CDCl$_3$)** δ 161.3 (q, *J* = 34 Hz), 158.1, 155.0, 147.4, 146.7, 133.0, 130.3, 129.2, 128.5, 127.9, 127.7, 126.8, 123.7, 119.2 (q, *J* = 277 Hz), 117.7, 114.8, 79.9, 70.0, 68.8, 59.0, 28.1; **^{19}F NMR (471 MHz, CDCl$_3$)** δ -71.03;

HRMS (ESI) m/z [M+H]$^+$: calcd. 570.2210, found. 570.2211; **IR (film):** ν_{max} (cm^{-1}) 3447, 2960, 2926, 1716, 1609, 1522, 1492, 1347, 1198, 1138, 1025, 848, 705;

Optical Rotation: $[\alpha]_D^{25}$ = +20.6 (*c* = 1.00, CHCl$_3$, 94% ee); **HPLC:** DAICEL CHIRALPAK ID, hexane/*i*-PrOH = 95/5, flow rate: 1.0 mL/min, λ = 254 nm, t_R(minor) = 10.7 min, t_R(major) = 25.5 min, ee = 94%.

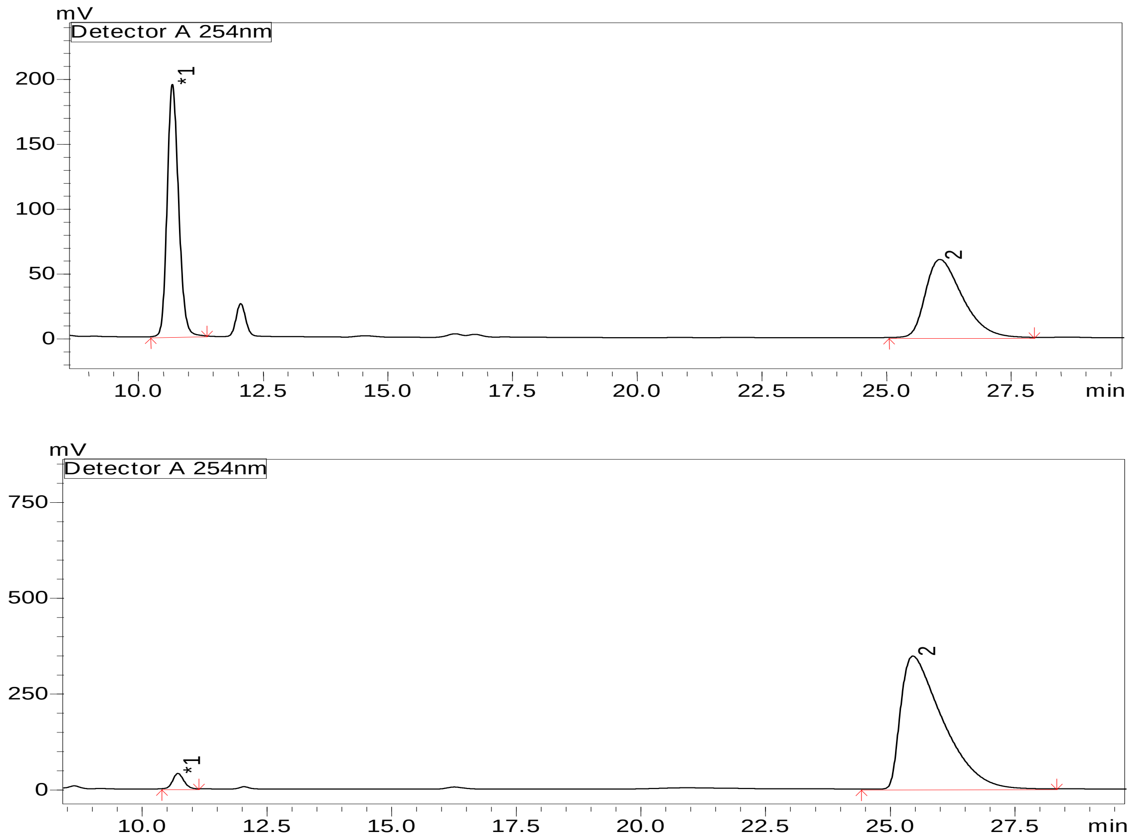

Peak#	Ret. Time	Area%
1	10.706	49.932
2	26.096	50.068

Peak#	Ret. Time	Area%
1	10.741	2.915
2	25.480	97.085

Supplementary Figure 5. HPLC chromatogram for compound **3ae**

***tert*-butyl ((1*S*,2*S*)-1-(4-(difluoromethoxy)phenyl)-2-(4-nitrophenyl)-2-(((*E*)-2,2,2-trifluoro-1-phenylethylidene)amino)ethyl)carbamate** (**3af**)

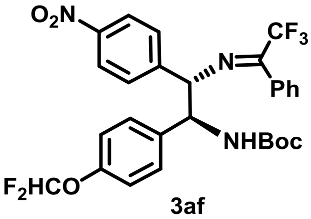

3af

white solid, 85.7 mg (74% yield);

^{1}H NMR (400 MHz, $CDCl_3$) δ 8.24 (d, *J* = 8.7 Hz, 2H), 7.53 (d, *J* = 8.7 Hz, 2H), 7.38 (t, *J* = 7.6 Hz, 1H), 7.24 (t, *J* = 7.6 Hz, 2H), 7.10 (s, 4H), 6.55 (t, *J* = 18.0 Hz, 1H), 6.38 (d, *J* = 8.0 Hz, 2H), 5.84 (d, *J* = 8.0 Hz, 1H), 5.16 (d, *J* = 8.0 Hz, 1H), 4.72 (s, 1H), 1.30 (s, 9H).; **^{13}C NMR (125 MHz, $CDCl_3$)** δ 161.7 (q, *J* = 34 Hz), 155.0, 150.3, 147.5, 146.3, 136.9, 130.5, 128.9, 128.6, 128.0, 127.9, 126.6, 123.8, 120.1, 119.1 (q, *J* = 277 Hz), 115.6 (t, *J* = 259 Hz), 80.2, 69.6, 59.1, 28.1; **^{19}F NMR (471 MHz, $CDCl_3$)** δ -70.93, -81.19;

HRMS (ESI) m/z [M+H]$^+$: calcd. 580.1865, found. 580.1866;

IR (film): ν_{max} (cm^{-1}) 3441, 2958, 2925, 1710, 1607, 1524, 1495, 1347, 1199, 1165, 1134, 1046, 976, 855, 704;

Optical Rotation: $[\alpha]_D^{25}$ = +36.9 (*c* = 1.00, $CHCl_3$, 94% ee); **HPLC:** DAICEL CHIRALPAK ID, hexane/*i*-PrOH = 95/5, flow rate: 1.0 mL/min, λ = 254 nm, t_R(minor) = 9.5 min, t_R(major) = 14.9 min, ee = 94%.

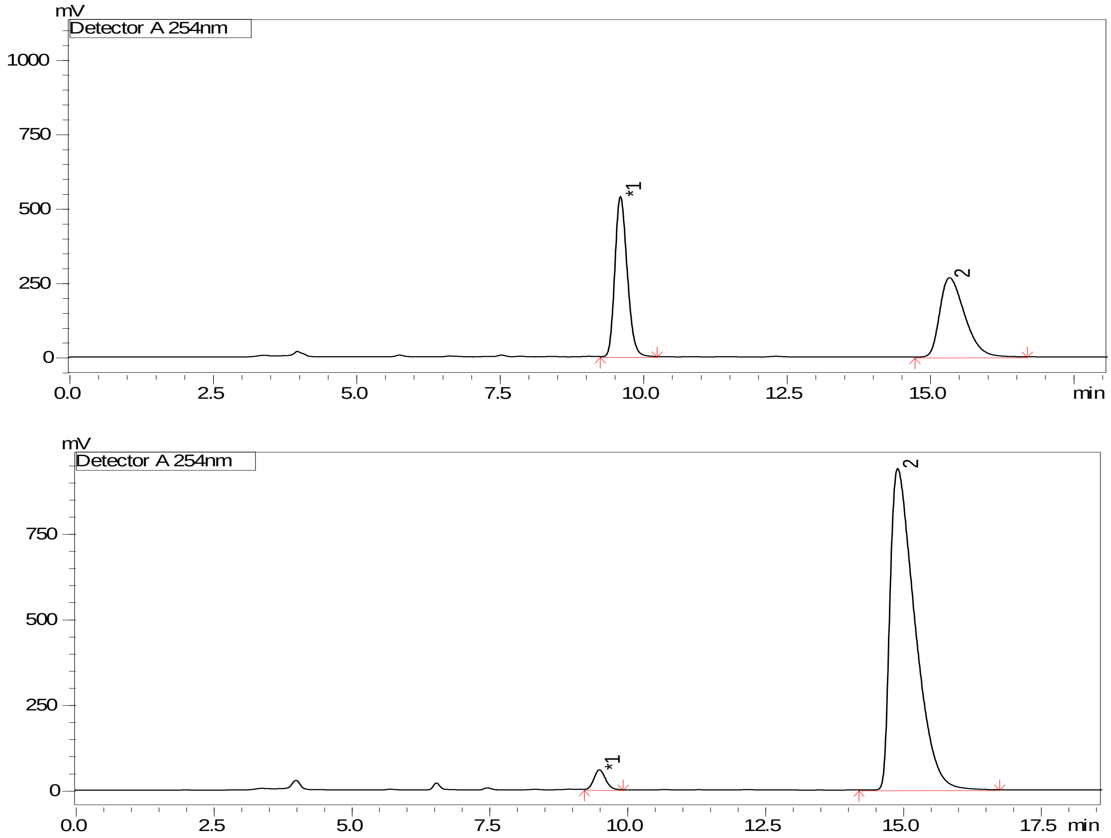

Peak#	Ret. Time	Area%
1	9.616	49.955
2	15.352	50.045

Peak#	Ret. Time	Area%
1	9.509	2.774
2	14.918	97.226

Supplementary Figure 6. HPLC chromatogram for compound **3af**

***tert*-butyl ((1*S*,2*S*)-1-(4-(2-chloroethoxy)phenyl)-2-(4-nitrophenyl)-2-(((*E*)-2,2,2-trifluoro-1-phenylethylidene)amino)ethyl)carbamate** (**3ag**)

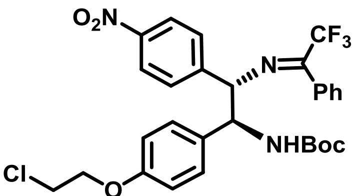

3ag

white solid, 103.0 mg (87% yield); **^{1}H NMR (400 MHz, CDCl$_3$)** δ 8.21 (d, *J* = 8.7 Hz, 2H), 7.50 (d, *J* = 8.7 Hz, 2H), 7.43-7.34 (m, 1H), 7.27-7.20 (m, 2H), 7.05-7.00 (m, 2H), 6.91-6.85 (m, 2H), 6.42 (d, *J* = 7.5 Hz, 2H), 5.77 (d, *J* = 8.0 Hz, 1H), 5.11 (d, *J* = 8.0 Hz, 1H), 4.70 (s, 1H), 4.30-4.19 (m, 2H), 3.87-3.80 (m, 2H), 1.29 (s, 9H); **^{13}C NMR (125 MHz, CDCl$_3$)** δ 161.3 (q, *J* = 34 Hz), 157.8, 155.0, 147.4, 146.7, 130.4, 129.1, 128.6, 127.9, 127.8, 126.8, 123.7, 119.2 (q, *J* = 279 Hz), 114.8, 80.0, 69.9, 68.2, 59.0, 41.9, 28.1; **^{19}F NMR (471 MHz, CDCl$_3$)** δ -70.96;

HRMS (ESI) m/z [M+H]$^+$: calcd. 592.1821, found. 592.1820;

IR (film): ν_{max} (cm^{-1}) 3434, 2958, 2926, 1712, 1609, 1514, 1494, 1347, 1245, 1199, 1139, 1043, 848, 705;

Optical Rotation: $[\alpha]_D^{25}$ = +10.4 (*c* = 1.10, CHCl$_3$, 94% ee); **HPLC:** DAICEL CHIRALPAK IF-3, hexane/*i*-PrOH = 90/10, flow rate: 1.0 mL/min, λ = 254 nm, t_R(minor) = 8.9 min, t_R(major) = 24.2 min, ee = 94%.

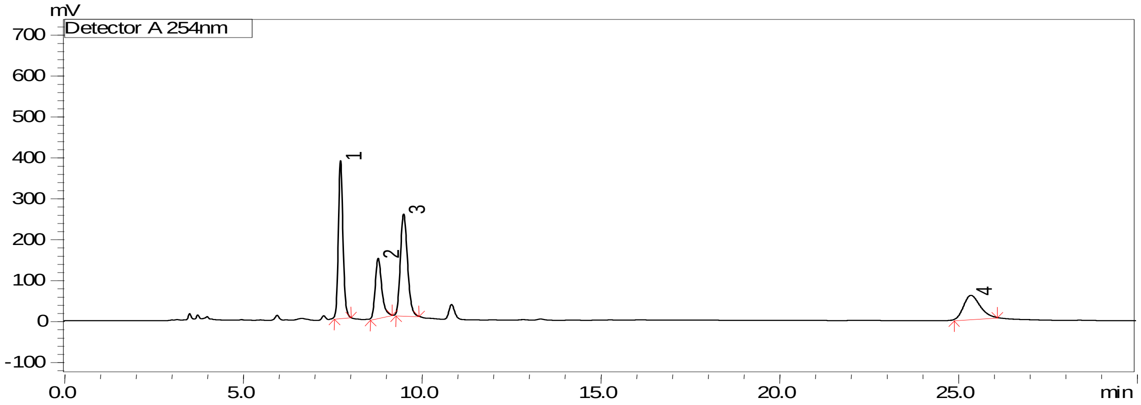

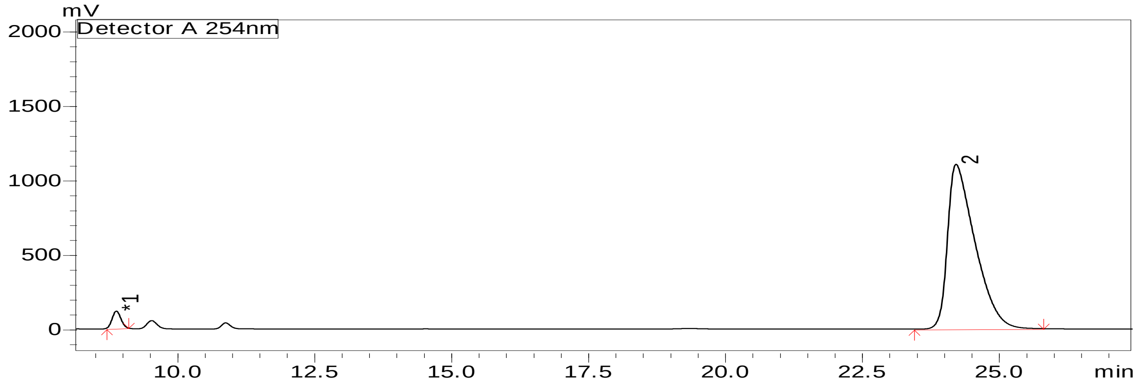

Peak#	Ret. Time	Area%
1	7.745	32.839
2	8.798	17.206
3	9.510	32.713
4	25.383	17.242

Peak#	Ret. Time	Area%
1	8.902	3.132
2	24.233	96.868

Supplementary Figure 7. HPLC chromatogram for compound **3ag**

***tert*-butyl ((1*S*,2*S*)-1-(4-(dimethylamino)phenyl)-2-(4-nitrophenyl)-2-(((*E*)-2,2,2-trifluoro-1-phenylethylidene)amino)ethyl)carbamate** (**3ah**)

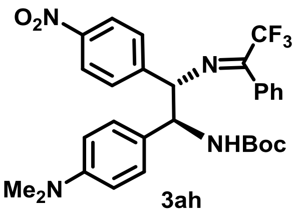

3ah

yellow solid, 91.2 mg (82% yield);
^{1}H NMR (400 MHz, $CDCl_3$) δ 8.19 (d, *J* = 8.4 Hz, 2H), 7.49 (d, *J* = 8.4 Hz, 2H), 7.40-7.34 (m, 1H), 7.25-7.18 (m, 2H), 6.92 (d, *J* = 8.4 Hz, 2H), 6.67 (d, *J* = 8.4 Hz, 2H), 6.42 (d, *J* = 8.4 Hz, 2H), 5.68 (d, *J* = 8.0 Hz, 1H), 5.06 (d, *J* = 8.0 Hz, 1H), 4.67 (s, 1H), 2.96 (s, 6H), 1.29 (s, 9H); **^{13}C NMR (125 MHz, $CDCl_3$)** δ 160.9 (q, *J* = 34 Hz), 155.0, 150.3, 147.3, 130.2, 129.4, 128.5, 128.0, 127.4, 127.0, 123.6, 119.3 (q, *J* = 277 Hz), 112.6, 79.7, 70.3, 59.2, 40.7, 28.2; **^{19}F NMR (471 MHz, $CDCl_3$)** δ -71.06;
HRMS (ESI) m/z $[M+H]^+$: calcd. 557.2370, found. 557.2369;
IR (film): ν_{max} (cm^{-1}) 3443, 2958, 2926, 1714, 1599, 1524, 1494, 1346, 1198, 1165, 1137, 1044, 844, 704;
Optical Rotation: $[\alpha]_D^{25}$ = +1.4 (*c* = 1.20, $CHCl_3$, 96% ee); **HPLC:** DAICEL CHIRALPAK ID, hexane/*i*-PrOH = 90/10, flow rate: 1.0 mL/min, λ = 254 nm, t_R(minor) = 9.7 min, t_R(major) = 15.3 min, ee = 96%.

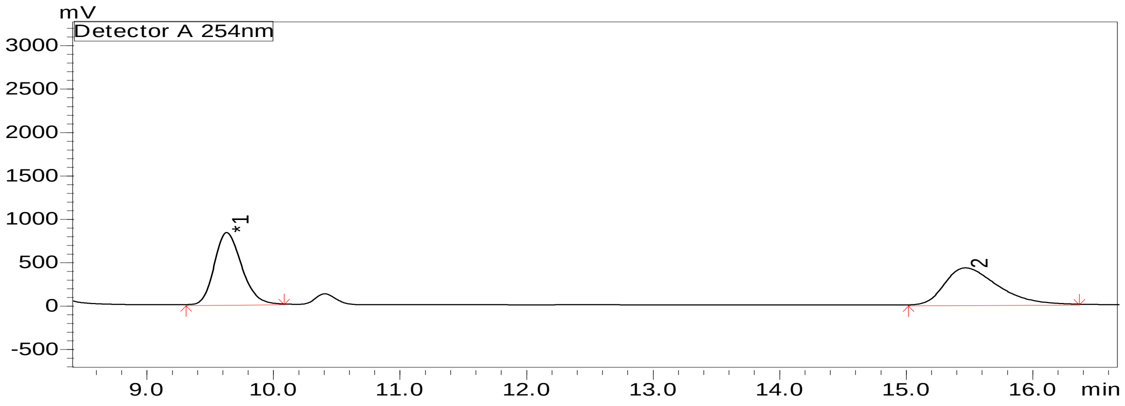

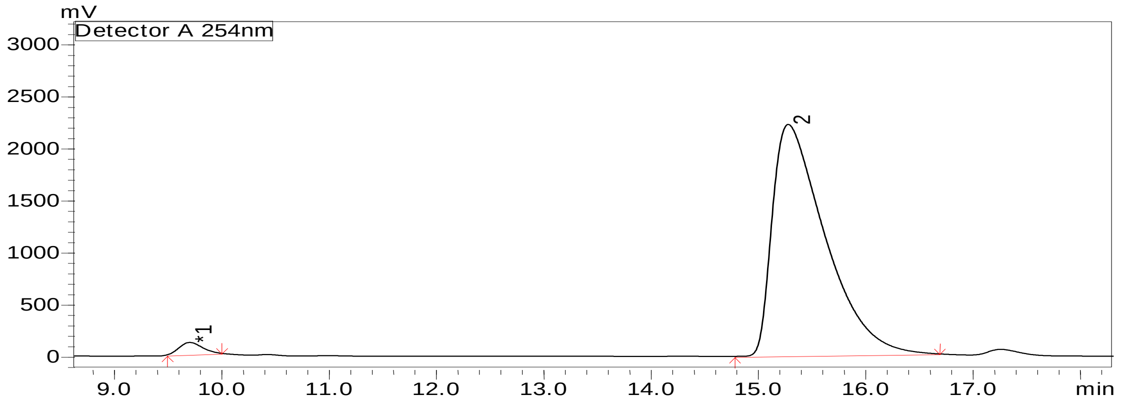

Peak#	Ret. Time	Area%
1	9.641	50.219
2	15.478	49.781

Peak#	Ret. Time	Area%
1	9.713	2.161
2	15.290	97.839

Supplementary Figure 8. HPLC chromatogram for compound **3ah**

***tert*-butyl ((1*S*,2*S*)-1-(methylthio)phenyl)-2-(4-nitrophenyl)-2-(((*E*)-2,2,2-trifluoro-1-phenylethylidene)amino)ethyl)carbamate** (**3ai**)

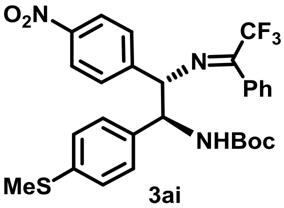

3ai

white solid, 80.5 mg (72% yield);

^{1}H NMR (400 MHz, $CDCl_3$) δ 8.21 (d, *J* = 8.7 Hz, 2H), 7.51 (d, *J* = 8.7 Hz, 2H), 7.40-7.34 (m, 1H), 7.26-7.17 (m, 4H), 7.03-7.68 (m, 2H), 6.39 (d, *J* = 7.5 Hz, 2H), 5.80 (d, *J* = 8.0 Hz, 1H), 5.11 (d, *J* = 8.0 Hz, 1H), 4.71 (s, 1H), 2.50 (s, 3H), 1.29 (s, 9H); **^{13}C NMR (125 MHz, $CDCl_3$)** δ 161.4 (q, *J* = 34 Hz), 155.0, 147.4, 146.5, 138.1, 136.3, 130.4, 129.0, 128.6, 127.9, 127.0, 126.7, 126.6, 123.7, 120.1 (q, *J* = 277 Hz), 80.0, 69.7, 59.2, 28.1, 15.9; **^{19}F NMR (471 MHz, $CDCl_3$)** δ -70.92;

HRMS (ESI) m/z [M+H]$^+$: calcd. 560.1825, found. 560.1824;

IR (film): ν_{max} (cm^{-1}) 3438, 2959, 2925, 1712, 1600, 1524, 1493, 1347, 1228, 1139, 1045, 848, 702;

Optical Rotation: $[\alpha]_D^{25}$ = -13.9 (*c* = 1.00, $CHCl_3$, 96% ee); **HPLC:** DAICEL CHIRALPAK ID, hexane/*i*-PrOH = 90/10, flow rate: 1.0 mL/min, λ = 254 nm, t_R(minor) = 8.6 min, t_R(major) = 16.0 min, ee = 96%.

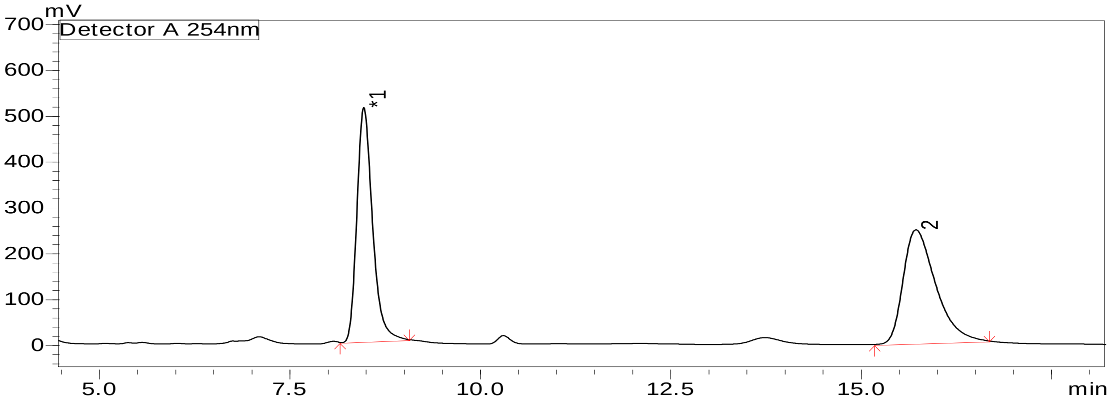

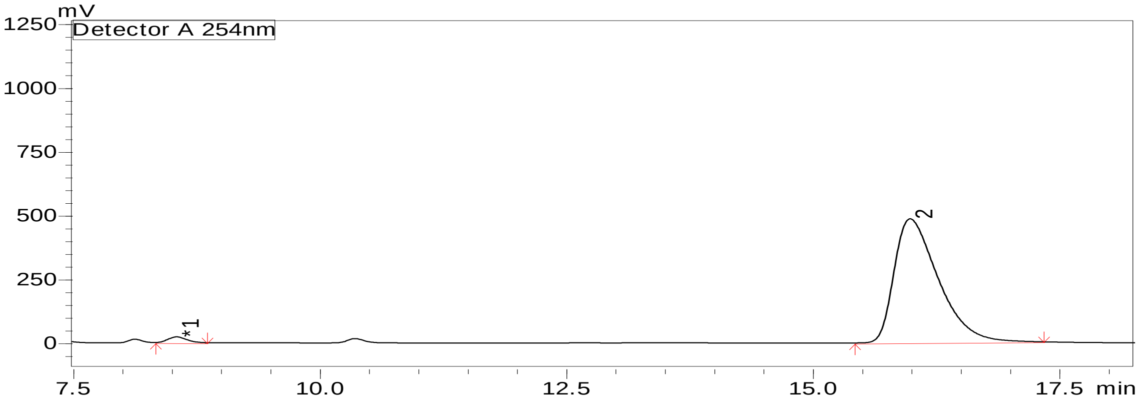

Peak#	Ret. Time	Area%
1	8.486	49.848
2	15.736	50.152

Peak#	Ret. Time	Area%
1	8.557	1.893
2	15.996	98.107

Supplementary Figure 9. HPLC chromatogram for compound **3ai**

***tert*-butyl ((1*S*,2*S*)-2-(4-nitrophenyl)-2-(((*E*)-2,2,2-trifluoro-1-phenylethylidene)amino)-1-(4-vinylphenyl)ethyl)carbamate** (**3aj**)

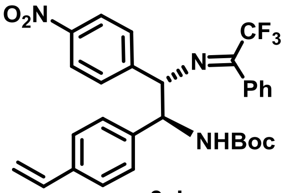

3aj

white solid, 73.4 mg (68% yield);
^{1}H NMR (400 MHz, $CDCl_3$) δ 8.22 (d, *J* = 8.7 Hz, 2H), 7.50 (d, *J* = 8.7 Hz, 2H), 7.38-7.34 (m, 3H), 7.23-7.17 (m, 2H), 7.05-7.02 (m, 2H), 6.74 (dd, *J* = 17.6, 11.0 Hz, 1H), 6.36 (d, *J* = 7.5 Hz, 2H), 5.83 (br, 1H), 5.78 (d, *J* = 17.6 Hz, 1H), 5.29 (d, *J* = 11.0 Hz, 1H), 5.15 (d, *J* = 8.0 Hz, 1H), 4.72 (br, 1H), 1.30 (s, 9H); **^{13}C NMR (125 MHz, $CDCl_3$)** δ 161.4 (q, *J* = 34 Hz), 155.0, 147.4, 146.6, 139.0, 137.2, 136.2, 130.3, 129.1, 128.5, 127.9, 126.8, 126.3, 123.8, 119.2 (q, *J* = 277 Hz), 114.3, 80.0, 69.7, 59.4, 28.2; **^{19}F NMR (471 MHz, $CDCl_3$)** δ -70.97;

HRMS (ESI) m/z [M+Na]$^{+}$: calcd. 562.1924, found. 562.1921;

IR (film): ν_{max} (cm^{-1}) 3445, 2969, 2922, 1712, 1524, 1493, 1346, 1199, 1050, 904, 853, 705;

Optical Rotation: $[\alpha]_D^{25}$ = -6.0 (*c* = 1.00, $CHCl_3$, 95% ee); **HPLC:** DAICEL CHIRALPAK ID, hexane/*i*-PrOH = 95/5, flow rate: 1.0 mL/min, λ = 254 nm, t_R(minor) = 8.8 min, t_R(major) = 19.3 min, ee = 95%.

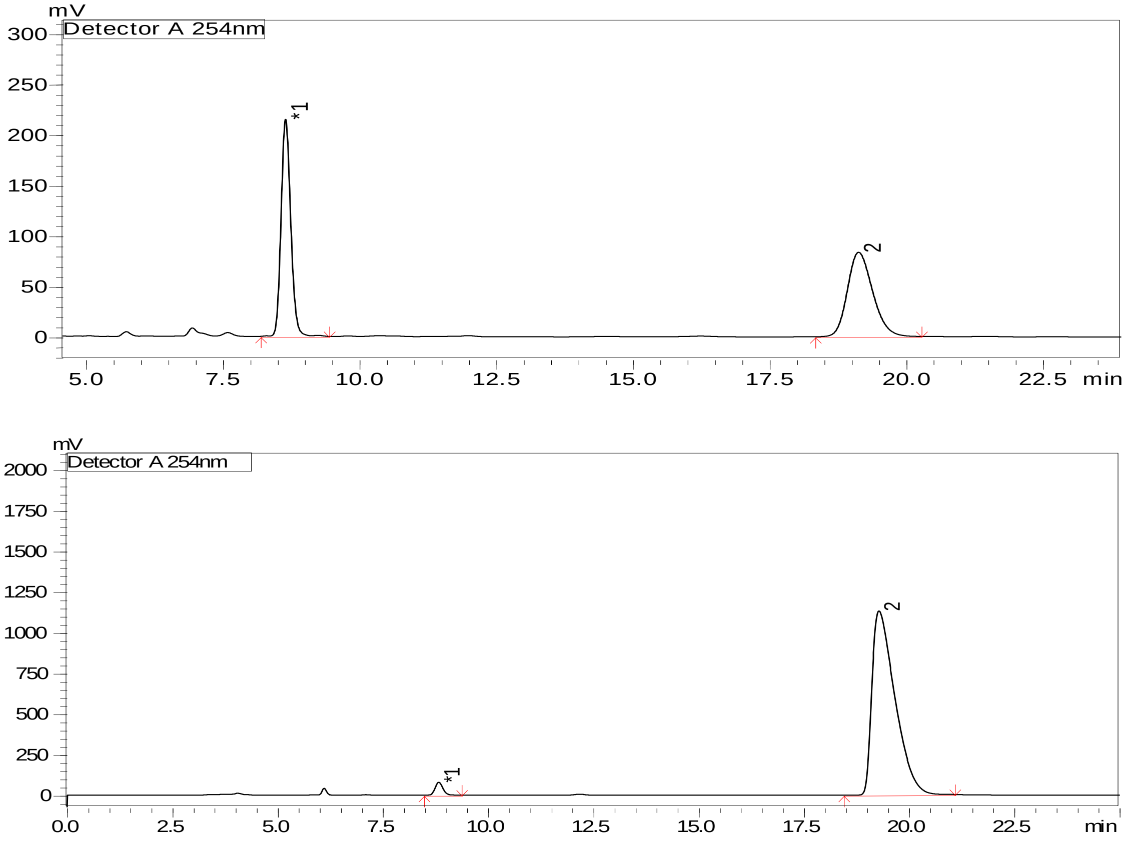

Peak#	Ret. Time	Area%
1	8.661	49.799
2	19.144	50.201

Peak#	Ret. Time	Area%
1	8.848	2.264
2	19.306	97.736

Supplementary Figure 10. HPLC chromatogram for compound **3aj**

***tert*-butyl ((1*S*,2*S*)-1-(4-fluorophenyl)-2-(4-nitrophenyl)-2-(((*E*)-2,2,2-trifluoro-1-phenylethylidene)amino)ethyl)carbamate (3ak)**

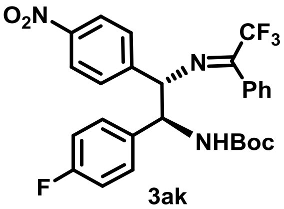

3ak

white solid, 81.8 mg (77% yield);

^{1}H NMR (400 MHz, $CDCl_3$) δ 8.23 (d, *J* = 8.7 Hz, 2H), 7.52 (d, *J* = 8.6 Hz, 2H), 7.39 (t, *J* = 7.5 Hz, 1H), 7.24 (t, *J* = 7.5 Hz, 2H), 7.15-6.97 (m, 4H), 6.41 (d, *J* = 7.5 Hz, 2H), 5.82 (d, *J* = 7.9 Hz, 1H), 5.15 (d, *J* = 7.9 Hz, 1H), 4.71 (s, 1H), 1.29 (s, 9H); **^{13}C NMR (125 MHz, $CDCl_3$)** δ 162.2 (d, *J* = 120 Hz), 161.6 (q, *J* = 34 Hz), 155.0, 147.5, 146.4, 135.4, 130.5, 129.0, 128.6, 128.2 (d, *J* = 7.5 Hz), 127.8, 126.6, 123.8, 119.1 (q, *J* = 277 Hz), 115.41 (d, *J* = 21 Hz), 80.1, 69.7, 59.0, 28.1; **^{19}F NMR (471MHz, $CDCl_3$)** δ -70.94, -114.43;

HRMS (ESI) m/z $[M+Na]^+$: calcd. 554.1673, found. 554.1672;

IR (film): ν_{max} (cm^{-1}) 3441, 2957, 2926, 1712, 1606, 1524, 1495, 1367, 1224, 1199, 1044, 851, 704;

Optical Rotation: $[\alpha]_D^{25}$ = +71.2 (*c* = 1.00, $CHCl_3$, 94% ee); **HPLC:** DAICEL CHIRALPAK ID, hexane/*i*-PrOH = 96/4, flow rate: 1.0 mL/min, λ = 254 nm, t_R(minor) = 10.2 min, t_R(major) = 24.0 min, ee = 94%.

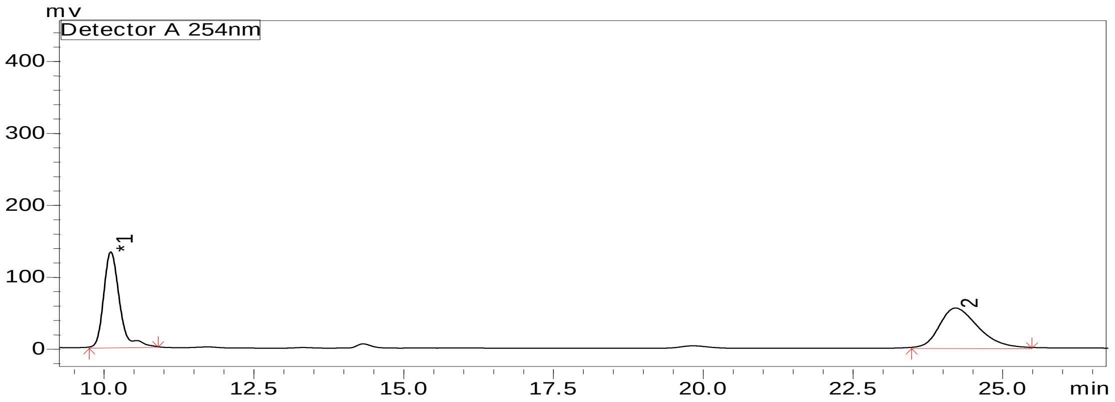

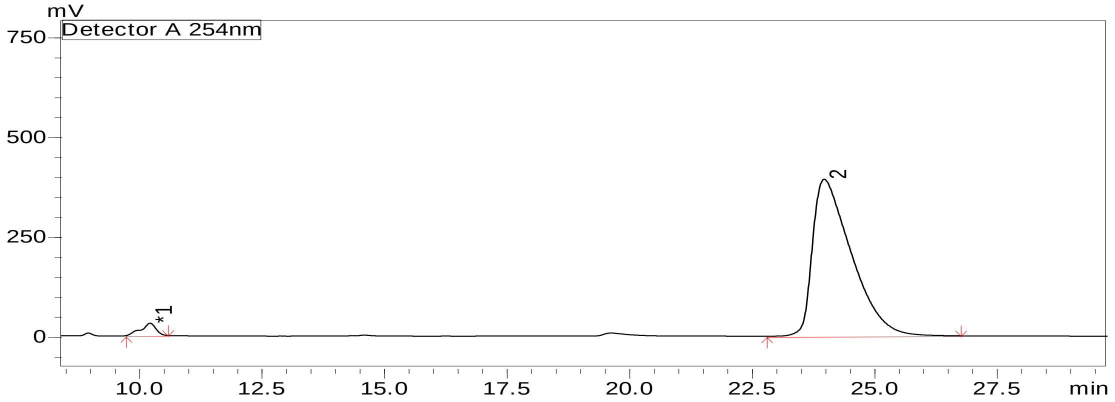

Peak#	Ret. Time	Area%
1	10.132	49.917
2	24.233	50.083

Peak#	Ret. Time	Area%
1	10.241	2.932
2	23.996	97.068

Supplementary Figure 11. HPLC chromatogram for compound **3ak**

tert-butyl ((1_S_,2_S_)-1-(4-chlorophenyl)-2-(4-nitrophenyl)-2-(((_E_)-2,2,2-trifluoro-1-phenylethylidene)amino)ethyl)carbamate (3al)

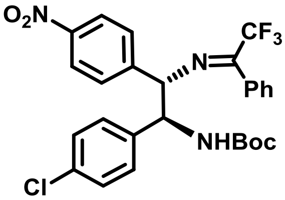

3al

white solid, 54.1 mg (49% yield);

^{1}H NMR (400 MHz, $CDCl_3$) δ 8.23 (d, J = 8.7 Hz, 2H), 7.51 (d, J = 8.7 Hz, 2H), 7.39 (t, J = 7.5 Hz, 1H), 7.31 (d, J = 8.4 Hz, 2H), 7.25 (t, J = 7.7 Hz, 2H), 7.04 (d, J = 8.4 Hz, 2H), 6.41 (d, J = 7.5 Hz, 2H), 5.81 (d, J = 8.4 Hz, 1H), 5.13 (d, J = 8.4 Hz, 1H), 4.72 (s, 1H), 1.30 (s, 9H); **^{13}C NMR (100 MHz, $CDCl_3$)** δ 161.7 (q, J = 34 Hz), 154.9, 147.5, 146.3, 138.1, 133.6, 130.5, 128.9, 128.7, 127.9, 127.8, 126.6, 123.9, 119.1 (q, J = 279 Hz), 80.2, 69.5, 59.1, 28.1; **^{19}F NMR (376MHz, $CDCl_3$)** δ -70.91;

HRMS (ESI) m/z $[M+H]^+$: calcd. 548.1558, found. 548.1559;

IR (film): ν_{max} (cm^{-1}) 3422, 2978, 2931, 1708, 1600, 1525, 1492, 1347, 1270, 1200, 1045, 850, 702;

Optical Rotation: $[\alpha]_D^{25}$ = +15.5 (c = 1.13, $CHCl_3$, 98% ee); **HPLC:** DAICEL CHIRALPAK ID, hexane/i-PrOH = 95/5, flow rate: 1.0 mL/min, λ = 254 nm, t_R(minor) = 8.4 min, t_R(major) = 18.7 min, ee = 98%.

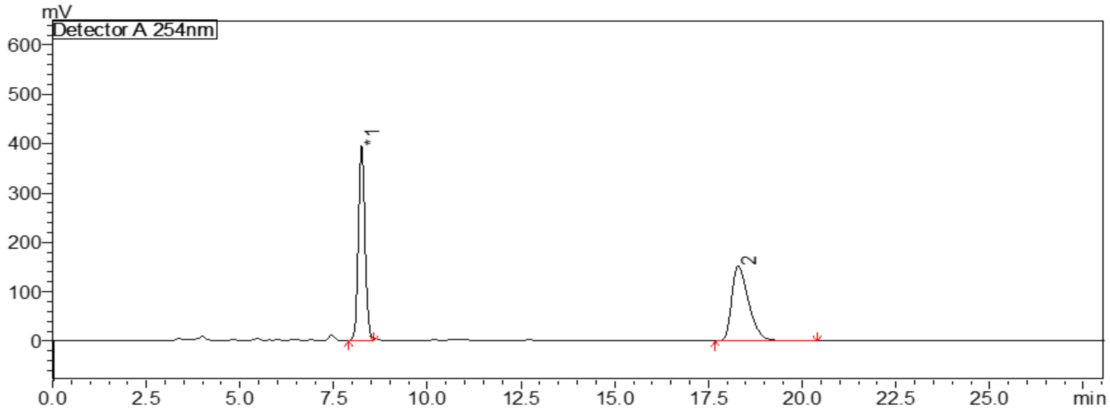

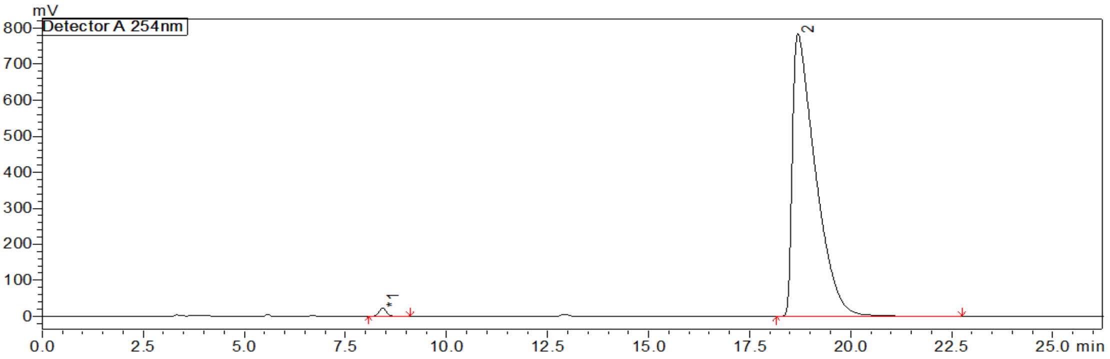

Peak#	Ret. Time	Area%
1	8.240	50.413
2	18.292	49.587

Peak#	Ret. Time	Area%
1	8.422	1.022
2	18.695	98.998

Supplementary Figure 12. HPLC chromatogram for compound **3al**

***tert*-butyl ((1*S*,2*S*)-1-(4-bromophenyl)-2-(4-nitrophenyl)-2-(((*E*)-2,2,2-trifluoro-1-phenylethylidene)amino)ethyl)carbamate (3am)**

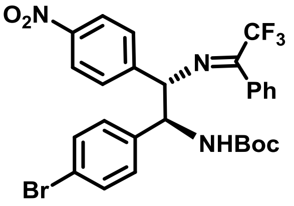

3am

white solid, 51.8 mg (44% yield);

^{1}H NMR (400 MHz, $CDCl_3$) δ 8.23 (d, *J* = 8.7 Hz, 2H), 7.51 (d, *J* = 8.7 Hz, 2H), 7.46 (d, *J* = 8.4 Hz, 2H), 7.39 (t, *J* = 7.6 Hz, 1H), 7.25 (t, *J* = 7.7 Hz, 2H), 6.98 (d, *J* = 8.4 Hz, 2H), 6.41 (d, *J* = 7.5 Hz, 2H), 5.81 (d, *J* = 8.6 Hz, 1H), 5.11 (d, *J* = 8.6 Hz, 1H), 4.72 (s, 1H), 1.30 (s, 9H); **^{13}C NMR (100 MHz, $CDCl_3$)** δ 161.7 (q, *J* = 34 Hz), 155.0, 147.5, 146.3, 138.6, 131.6, 130.5, 128.9, 128.7, 128.3, 127.8, 126.6, 123.9, 121.6, 119.1 (q, *J* = 279 Hz), 80.3, 69.4, 59.2, 28.1; **^{19}F NMR (376MHz, $CDCl_3$)** δ -70.88;

HRMS (ESI) m/z $[M+H]^+$: calcd. 592.1053, found. 592.1053;

IR (film): ν_{max} (cm^{-1}) 3409, 2979, 2931, 1708, 1599, 1524, 1489, 1346, 1230, 1199, 1045, 849, 702;

Optical Rotation: $[\alpha]_D^{25}$ = +0.6 (*c* = 0.96, $CHCl_3$, 85% ee of another sample); **HPLC:** DAICEL CHIRALPAK ID, hexane/*i*-PrOH = 95/5, flow rate: 1.0 mL/min, λ = 254 nm, t_R(minor) = 8.5 min, t_R(major) = 17.8 min, ee = 81%.

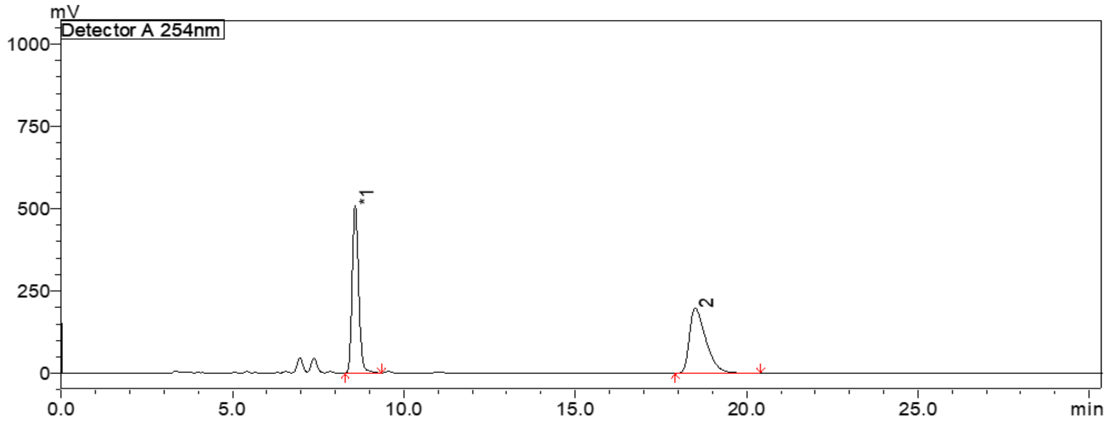

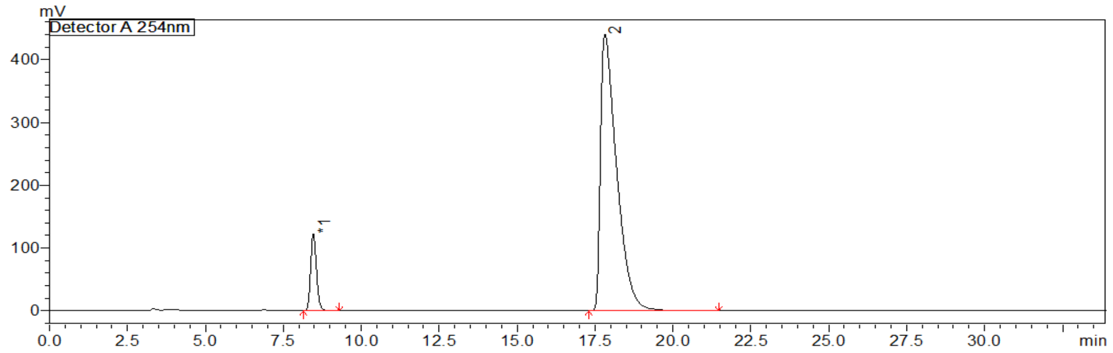

Peak#	Ret. Time	Area%
1	8.578	50.441
2	18.503	49.559

Peak#	Ret. Time	Area%
1	8.464	9.255
2	17.822	90.745

Supplementary Figure 13. HPLC chromatogram for compound **3am**

***tert*-butyl ((1*S*,2*S*)-2-(4-nitrophenyl)-1-(*o*-tolyl)-2-(((*E*)-2,2,2-trifluoro-1-phenylethylidene)amino)ethyl)carbamate** (**3an**)

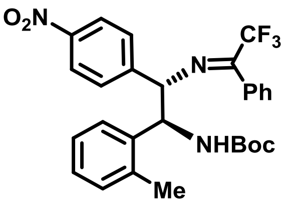

3an

white solid, 72.8 mg (69% yield);

^{1}H NMR (400 MHz, $CDCl_3$) δ 8.23 (d, *J* = 8.8 Hz, 2H), 7.53 (d, *J* = 8.6 Hz, 2H), 7.39-7.32 (m 1H), 7.30-7.22 (m, 2H), 7.21-7.15 (m, 2H), 7.14-7.06 (m, 2H), 6.21 (d, *J* = 7.5 Hz, 2H), 5.89 (d, *J* = 8.0 Hz, 1H), 5.39 (d, *J* = 8.0 Hz, 1H), 4.66 (s, 1H), 2.12 (s, 3H), 1.29 (s, 9H); **^{13}C NMR (125 MHz, $CDCl_3$)** δ 161.6 (q, *J* = 34 Hz), 155.1, 147.4, 146.7, 137.4, 134.6, 130.7, 130.2, 129.1, 128.5, 127.7, 127.6, 126.6, 126.3, 126.1, 123.8, 119.2 (q, *J* = 276 Hz), 80.0, 67.1, 56.0, 28.2, 18.8; **^{19}F NMR (471MHz, $CDCl_3$)** δ -71.14;

HRMS (ESI) m/z [M+Na]$^{+}$: calcd. 550.1924, found. 550.1921;

IR (film): ν_{max} (cm^{-1}) 3445, 2966, 2926, 1716, 1606, 1525, 1494, 1346, 1199, 1139, 1045, 976, 736, 706;

Optical Rotation: $[\alpha]_D^{25}$ = +59.3 (*c* = 1.00, $CHCl_3$, 97% ee); **HPLC:** DAICEL CHIRALPAK ID, hexane/*i*-PrOH = 95/5, flow rate: 1.0 mL/min, λ = 254 nm, t_R(minor) = 6.9 min, t_R(major) = 7.3min, ee = 97%.

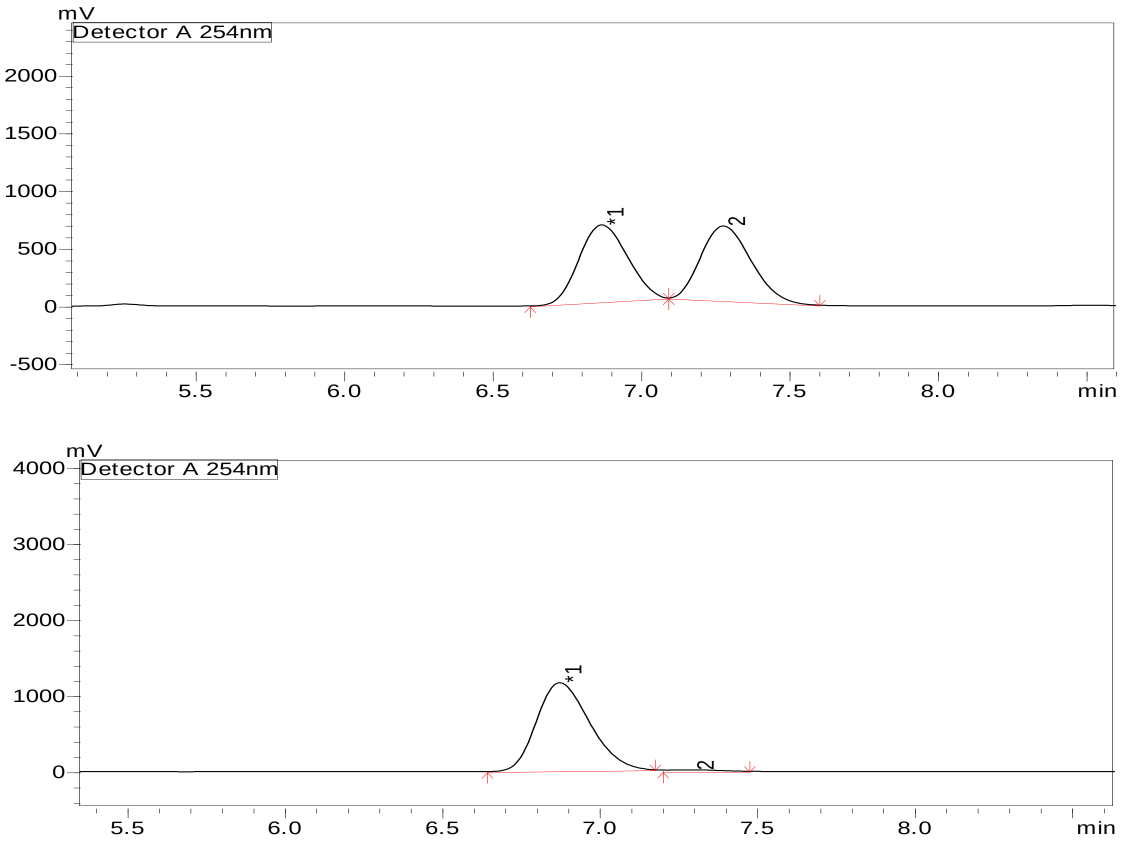

Peak#	Ret. Time	Area%
1	6.870	49.871
2	7.280	50.129

Peak#	Ret. Time	Area%
1	6.875	98.546
2	7.295	1.454

Supplementary Figure 14. HPLC chromatogram for compound **3an**

***tert*-butyl ((1*S*,2*S*)-1-(2-fluorophenyl)-2-(4-nitrophenyl)-2-(((*E*)-2,2,2-trifluoro-1-phenylethylidene)amino)ethyl)carbamate** (**3ao**)

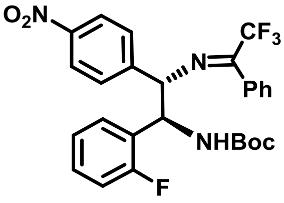

3ao

white solid, 69.1 mg (65% yield);
^{1}H NMR (400 MHz, $CDCl_3$) δ 8.22 (d, *J* = 8.6 Hz, 2H), 7.54 (d, *J* = 8.6 Hz, 2H), 7.43-7.36 (m, 1H), 7.35-7.28 (m, 1H), 7.27-7.21 (m, 2H), 7.20-7.12 (m, 2H), 6.99-6.91 (m, 1H), 6.38 (d, *J* = 7.6 Hz, 2H), 5.81 (d, *J* = 9.0 Hz, 1H), 5.48 (d, *J* = 9.0 Hz, 1H), 4.82 (s, 1H), 1.31 (s, 9H); **^{13}C NMR (125 MHz, $CDCl_3$)** δ161.8 (q, *J* = 34 Hz), 159.9 (d, *J* = 245 Hz), 154.9, 147.5, 146.4, 130.4, 129.4 (d, *J* = 8.7 Hz), 129.0, 128.7, 128.0 (d, *J* = 3.8 Hz), 127.9, 126.6, 124.2, 123.8, 119.2 (q, *J* = 277 Hz), 115.5 (d, *J* = 23 Hz), 80.2, 68.0, 54.5, 28.1; **^{19}F NMR (471MHz, $CDCl_3$)** δ -71.05, -118.75;
HRMS (ESI) m/z $[M+H]^+$: calcd. 532.1854, found. 532.1852;
IR (film): ν_{max} (cm^{-1}) 3439, 2979, 2930, 1715, 1603, 1525, 1494, 1347, 1199, 1046, 976, 838, 759, 738, 705;
Optical Rotation: $[\alpha]_D^{25}$ = +39.4 (*c* = 1.10, $CHCl_3$, 91% ee); **HPLC:** DAICEL CHIRALPAK ID, hexane/*i*-PrOH = 19/1, flow rate: 0.5 mL/min, λ = 254 nm, t_R(minor) = 14.7 min, t_R(major) = 15.6 min, ee = 91%.

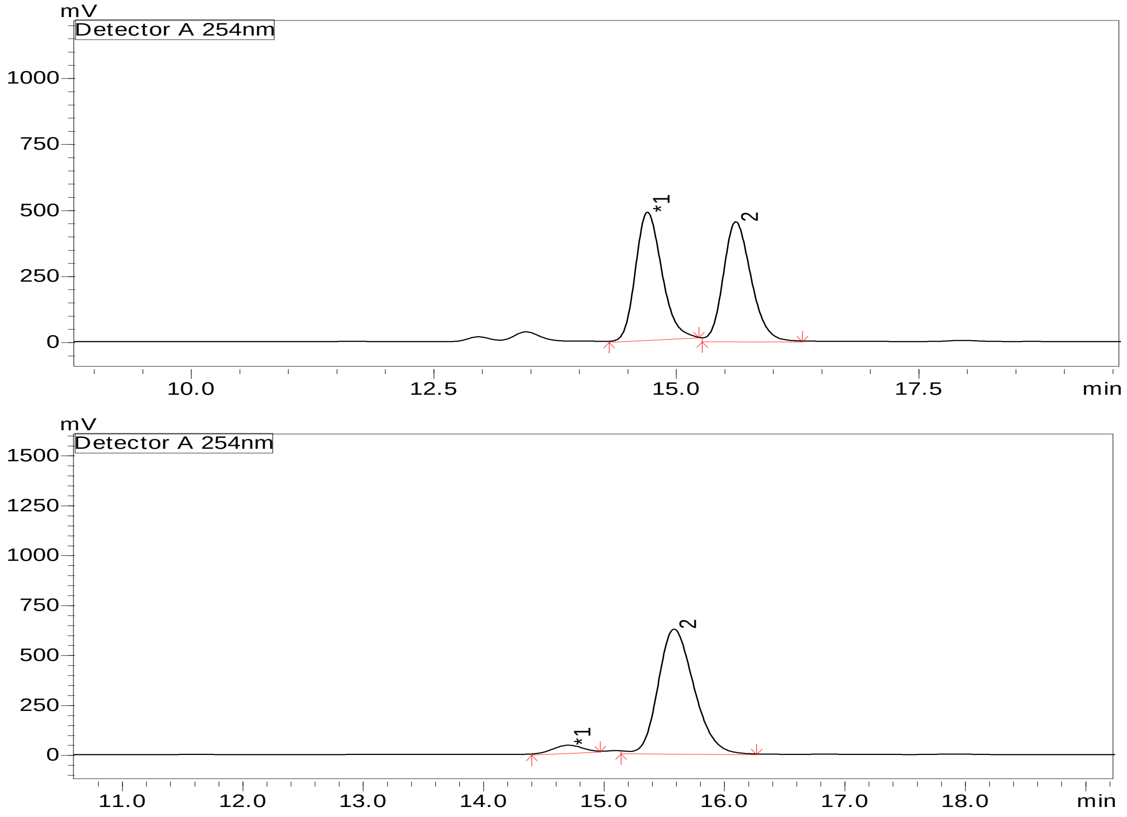

Peak#	Ret. Time	Area%
1	14.716	50.258
2	15.627	49.742

Peak#	Ret. Time	Area%
1	14.714	4.391
2	15.592	95.609

Supplementary Figure 15. HPLC chromatogram for compound **3ao**

***tert*-butyl ((1*S*,2*S*)-2-(4-nitrophenyl)-1-(*m*-tolyl)-2-(((*E*)-2,2,2-trifluoro-1-phenylethylidene)amino)ethyl)carbamate** (**3ap**)

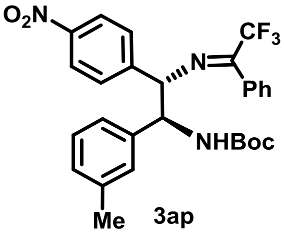

3ap

white solid, 80.0 mg (76% yield);
^{1}H NMR (400 MHz, $CDCl_3$) δ 8.22 (d, *J* = 8.7 Hz, 2H), 7.54 (d, *J* = 8.7 Hz, 2H), 7.39-7.33 (m, 1H), 7.25-7.17 (m, 3H), 7.16-7.11 (m, 1H), 6.94-6.85 (m, 2H), 6.28 (d, *J* = 7.5 Hz, 2H), 5.82 (d, *J* = 12 Hz, 1H), 5.13 (d, *J* = 12 Hz, 1H), 4.69 (s, 1H), 2.33 (s, 3H), 1.29 (s, 9H); **^{13}C NMR (125 MHz, $CDCl_3$)** δ 161.2 (q, *J* = 34 Hz), 155.0, 147.4, 146.6, 139.3, 138.3, 130.2, 129.1, 128.5, 128.4, 127.9, 127.4, 126.8, 123.7, 123.6, 119.2 (q, *J* = 277 Hz), 79.9, 69.9, 59.5, 28.2, 21.3; **^{19}F NMR (471MHz, $CDCl_3$)** δ -71.09;
HRMS (ESI) m/z [M+Na]$^+$: calcd. 550.1924, found. 550.1920;
IR (film): ν_{max} (cm^{-1}) 3447, 2968, 2925, 1716, 1607, 1525, 1491, 1347, 1199, 1139, 1046, 975, 776, 704;
Optical Rotation: $[\alpha]_D^{25}$ = +62.0 (*c* = 1.00, $CHCl_3$, 97% ee); **HPLC:** DAICEL CHIRALPAK ID, hexane/*i*-PrOH = 95/5, flow rate: 1.0 mL/min, λ = 254 nm, t_R(minor) = 8.5 min, t_R(major) = 19.8 min, ee = 97%.

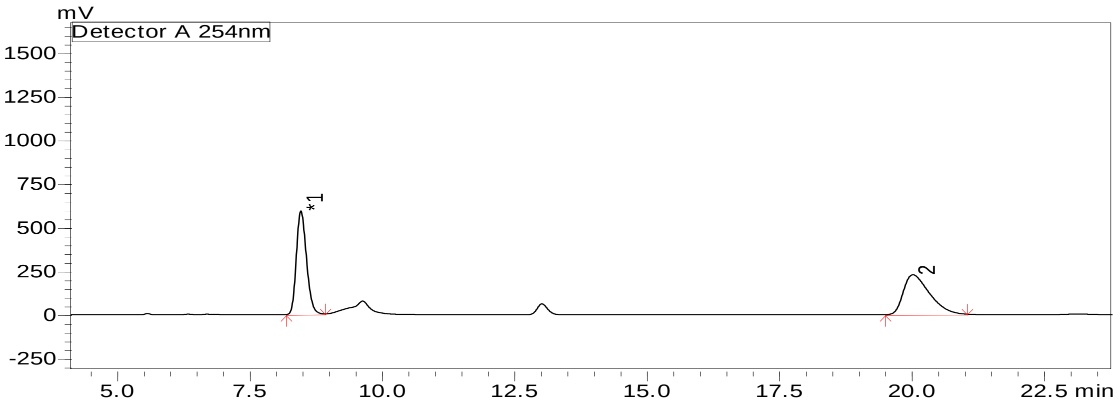

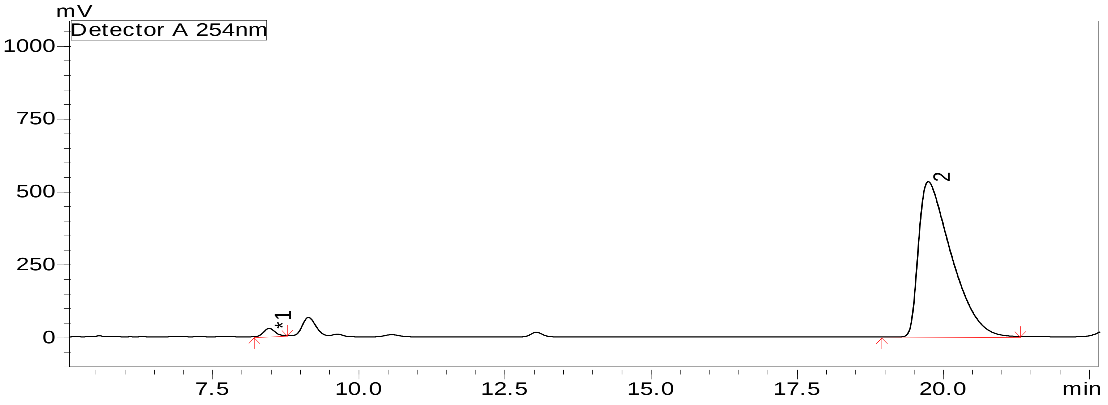

Peak#	Ret. Time	Area%
1	8.484	50.273
2	20.038	49.727

Peak#	Ret. Time	Area%
1	8.487	1.574
2	19.764	98.426

Supplementary Figure 16. HPLC chromatogram for compound **3ap**

***tert*-butyl ((1*S*,2*S*)-1-(3-methoxyphenyl)-2-(4-nitrophenyl)-2-(((*E*)-2,2,2-trifluoro-1-phenylethylidene)amino)ethyl)carbamate** (**3aq**)

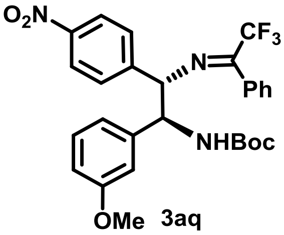

3aq

white solid, 70.6 mg (65% yield);
^{1}H NMR (400 MHz, $CDCl_3$) δ 8.21 (d, *J* = 8.7 Hz, 2H), 7.53 (d, *J* = 8.7 Hz, 2H), 7.40-7.33 (m, 1H), 7.27-7.18 (m, 3H), 6.86 (dd, *J* = 8.0, 2.0 Hz, 1H), 6.69 (d, *J* = 7.6 Hz, 1H), 6.62-6.59 (m, 1H), 6.36 (d, *J* = 7.5 Hz, 2H), 5.82 (d, *J* = 9.0 Hz, 1H), 5.14 (d, *J* = 9.0 Hz, 1H), 4.73 (s, 1H), 3.76 (s, 3H), 1.30 (s, 9H); **^{13}C NMR (125 MHz, $CDCl_3$)** δ 161.3 (q, *J* = 34 Hz), 159.8, 155.0, 147.4, 146.6, 141.0, 130.3, 129.6, 129.1, 128.5, 127.9, 126.8, 123.7, 119.2 (q, *J* = 277 Hz), 118.8, 113.5, 111.9, 80.0, 69.7, 59.6, 55.2, 28.1;
^{19}F NMR (471MHz, $CDCl_3$) δ -71.01;
HRMS (ESI) m/z $[M+Na]^+$: calcd. 566.1873, found. 566.1872;
IR (film): ν_{max} (cm^{-1}) 3443, 2965, 2926, 1718, 1601, 1525, 1494, 1347, 1199, 1139, 1045, 861, 778, 735, 702;
Optical Rotation: $[\alpha]_D^{25}$ = +62.5 (*c* = 1.00, $CHCl_3$, 85% ee); **HPLC:** DAICEL CHIRALPAK ID, hexane/*i*-PrOH = 95/5, flow rate: 1.0 mL/min, λ = 254 nm, t_R(minor) = 12.3 min, t_R(major) = 40.0 min, ee = 85%.

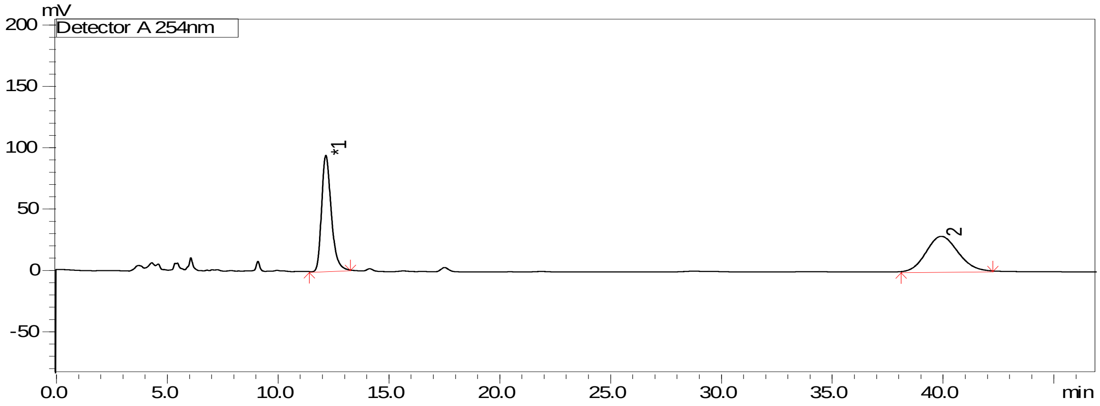

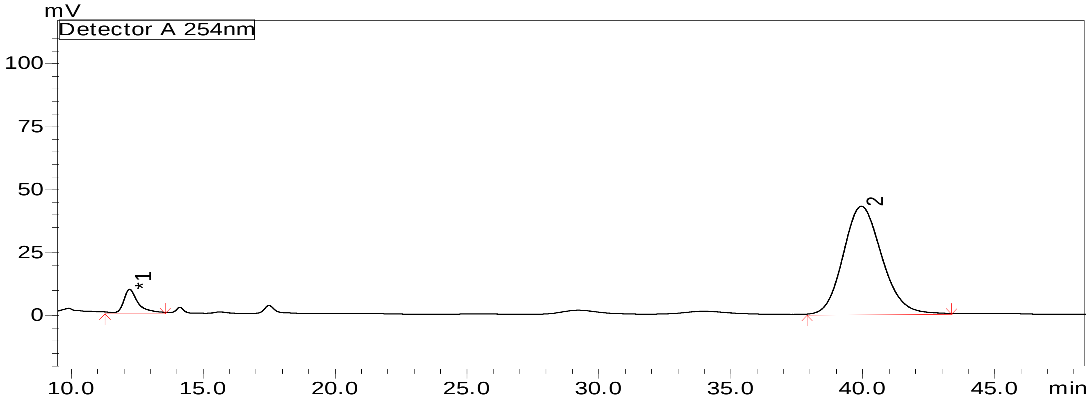

Peak#	Ret. Time	Area%
1	12.201	50.004
2	39.970	49.996

Peak#	Ret. Time	Area%
1	12.272	7.477
2	39.993	92.523

Supplementary Figure 17. HPLC chromatogram for compound **3aq**

***tert*-butyl ((1*S*,2*S*)-1-(naphthalen-1-yl)-2-(4-nitrophenyl)-2-(((*E*)-2,2,2-trifluoro-1-phenylethylidene)amino)ethyl)carbamate (3ar)**

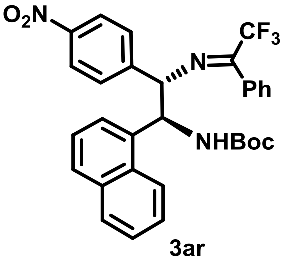

3ar

white solid, 84.5 mg (75% yield);
^{1}H NMR (400 MHz, $CDCl_3$) δ 8.28 (d, *J* = 8.7 Hz, 2H), 7.89 (t, *J* = 8.9 Hz, 2H), 7.82-7.77 (m, 1H), 7.67 (d, *J* = 8.6 Hz, 2H), 7.55 (t, *J* = 7.7 Hz, 1H), 7.49-7.43(m, 1H), 7.42-7.34 (m, 2H), 7.05 (t, *J* = 7.6 Hz, 1H), 6.76 (t, *J* = 7.7 Hz, 2H), 6.12-5.99 (m, 2H), 5.84 (d, *J* = 7.4 Hz, 2H), 4.90 (s, 1H), 1.33 (s, 9H); **^{13}C NMR (125 MHz, $CDCl_3$)** δ 161.7 (q, *J* = 34 Hz), 155.1, 147.5, 146.7, 134.5, 133.9, 130.0, 129.7, 129.1, 128.5, 127.9, 127.8, 126.5, 126.0, 125.7, 125.1, 123.8, 121.3, 119.1 (q, *J* = 277 Hz), 80.1, 67.3, 55.6, 28.2; **^{19}F NMR (471MHz, $CDCl_3$)** δ -71.13;

HRMS (ESI) m/z $[M+Na]^+$: calcd. 586.1924, found. 586.1920;

IR (film): ν_{max} (cm^{-1}) 3441, 2964, 2927, 1715, 1600, 1525, 1494, 1346, 1199, 1139, 1043, 975, 836, 776, 735, 705;

Optical Rotation: $[\alpha]_D^{25}$ = +37.5 (*c* = 1.00, $CHCl_3$, 87% ee); **HPLC:** DAICEL CHIRALPAK IC-3, hexane/*i*-PrOH = 95/5, flow rate: 1.0 mL/min, λ = 254 nm, t_R(minor) = 15.4 min, t_R(major) = 17.7 min, ee = 87%.

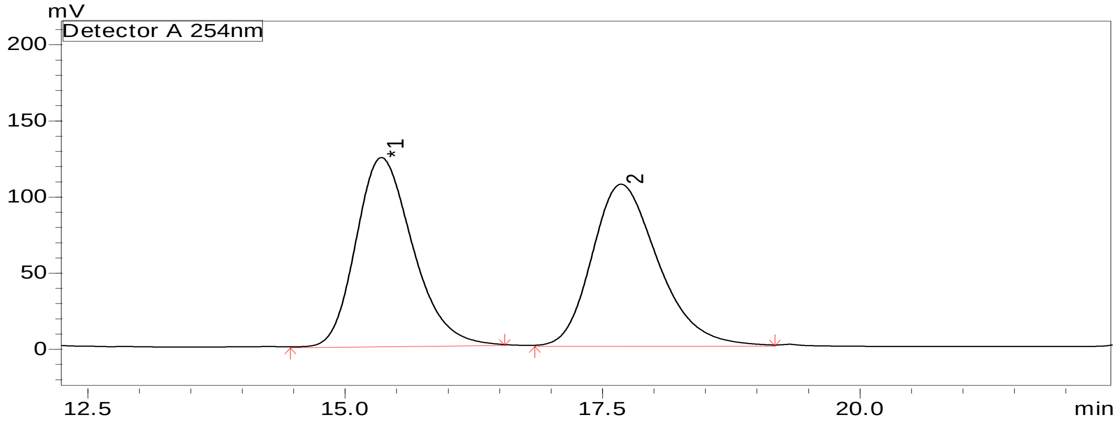

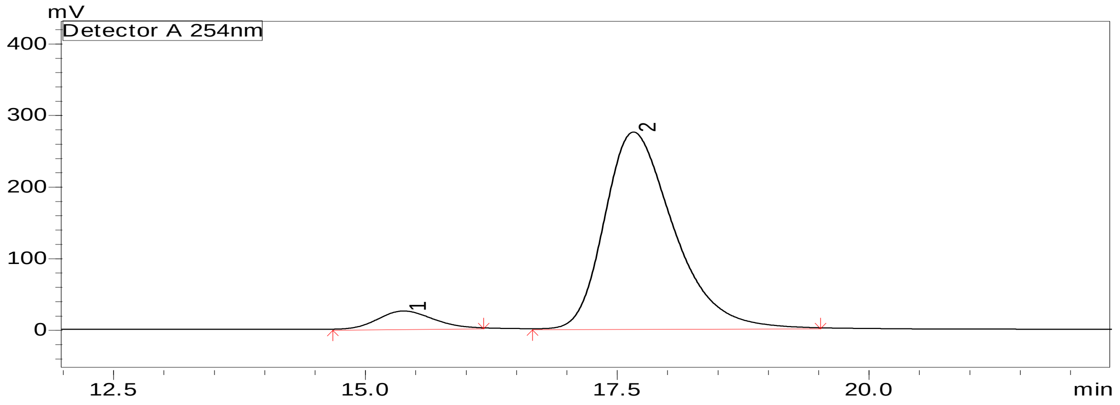

Peak#	Ret. Time	Area%
1	15.364	50.141
2	17.692	49.859

Peak#	Ret. Time	Area%
1	15.394	6.501
2	17.674	93.499

Supplementary Figure 18. HPLC chromatogram for compound **3ar**

***tert*-butyl ((1*S*,2*S*)-1-(naphthalen-2-yl)-2-(4-nitrophenyl)-2-(((*E*)-2,2,2-trifluoro-1-phenylethylidene)amino)ethyl)carbamate** (**3as**)

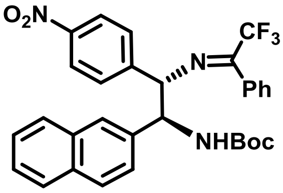

3as

white solid, 78.9 mg (70% yield);

^{1}H NMR (400 MHz, CDCl$_3$) δ 8.25 (d, J = 8.7 Hz, 2H), 7.90- 7.83 (m, 1H), 7.82-7.76 (m, 2H), 7.62-7.55 (m, 3H), 7.55-7.49 (m, 2H), 7.32-7.25 (m, 1H), 7.23-7.17 (m, 1H), 7.03 (t, J = 7.8 Hz, 2H), 6.19-6.13 (m, 2H), 6.02-5.91 (m, 1H), 5.40-5.28 (m, 1H), 4.86 (s, 1H) 1.32 (s, 9H); **^{13}C NMR (125 MHz, CDCl$_3$)** δ 161.5 (q, J = 35 Hz), 155.1, 147.5, 146.7, 136.8, 133.2, 132.8, 130.2, 128.9, 128.4, 128.3, 127.9, 127.8, 127.6, 126.6, 126.5, 126.2, 125.6, 124.4, 123.8, 119.1 (q, J = 277 Hz), 80.1, 69.7, 59.8, 28.2; **^{19}F NMR (471MHz, CDCl$_3$)** δ -70.96;

HRMS (ESI) m/z [M+Na]$^+$: calcd. 586.1924, found. 586.1921;

IR (film): ν_{max} (cm^{-1}) 3441, 2971, 2925, 1713, 1600, 1525, 1494, 1346, 1199, 1139, 1047, 976, 838, 734, 705;

Optical Rotation: $[\alpha]_D^{25}$ = -21.1 (c = 1.00, CHCl$_3$, 89% ee); **HPLC:** DAICEL CHIRALPAK ID, hexane/*i*-PrOH = 95/5, flow rate: 1.0 mL/min, λ = 254 nm, t_R(minor) = 10.6 min, t_R(major) = 22.8 min, ee = 89%.

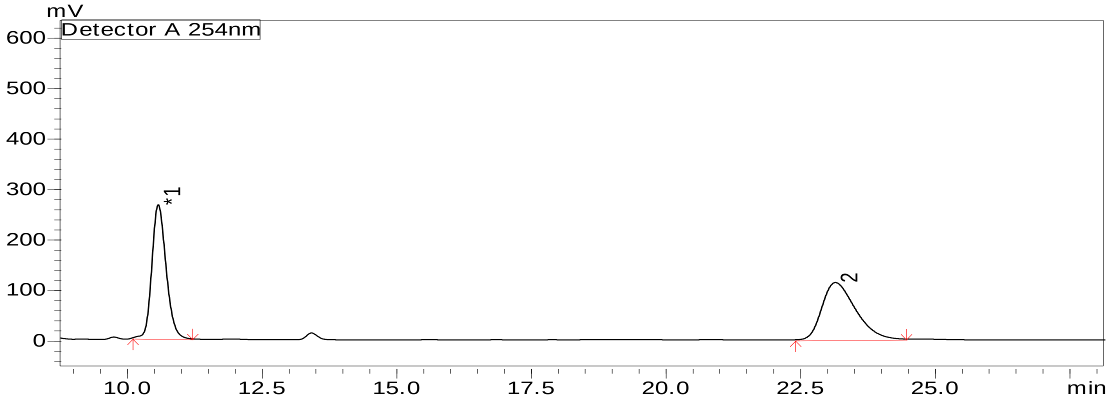

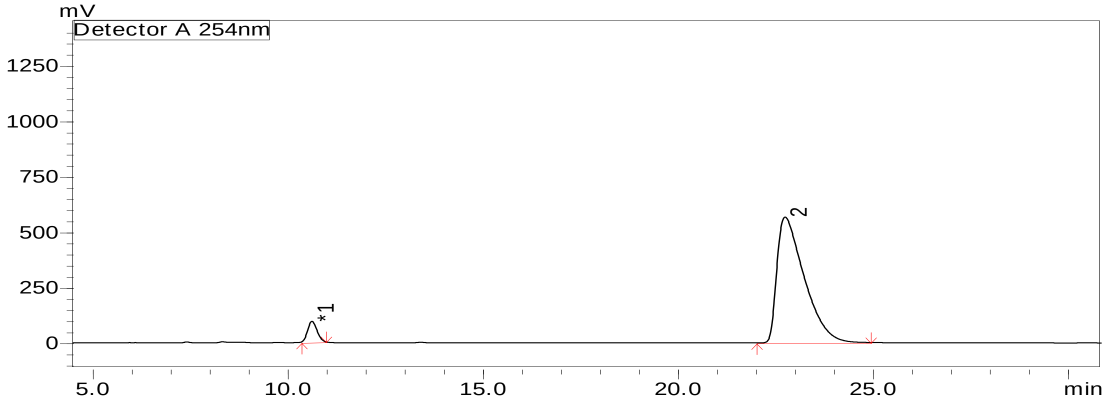

Peak#	Ret. Time	Area%
1	10.595	50.064
2	23.170	49.936

Peak#	Ret. Time	Area%
1	10.643	5.288
2	22.770	94.712

Supplementary Figure 19. HPLC chromatogram for compound **3as**

***tert*-butyl ((1*S*,2*S*)-2-(4-nitrophenyl)-1-(thiophen-3-yl)-2-(((*E*)-2,2,2-trifluoro-1-phenylethylidene)amino)ethyl)carbamate** (**3at**)

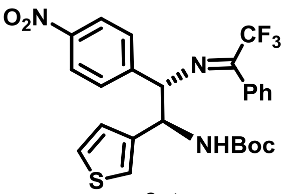

3at

white solid, 83.2 mg (80% yield);
^{1}H NMR (400 MHz, $CDCl_3$) δ 8.21 (d, *J* = 8.7 Hz, 2H), 7.49 (d, *J* = 8.7 Hz, 2H), 7.44-7.36 (m, 1H), 7.32-7.26 (m, 3H), 7.04-6.98 (m, 1H), 6.80 (dd, *J* = 5.0, 1.2 Hz, 1H), 6.54 (d, *J* = 7.5 Hz, 2H), 5.71 (d, *J* = 9.0 Hz, 1H), 5.25 (d, *J* = 9.0 Hz, 1H), 4.75 (s, 1H), 1.29 (s, 9H); **^{13}C NMR (125 MHz, $CDCl_3$)** δ 161.4 (q, *J* = 35 Hz), 155.0, 147.4, 146.5, 140.8, 130.4, 129.2, 128.7, 128.0, 126.8, 126.3, 126.0, 123.7, 121.6, 119.2 (q, *J* = 277 Hz), 80.0, 69.5, 56.0, 28.1; **^{19}F NMR (471MHz, $CDCl_3$)** δ -70.96;
HRMS (ESI) m/z [M+H]$^+$: calcd. 520.1512, found. 520.1511;
IR (film): ν_{max} (cm^{-1}) 3436, 2957, 2926, 1712, 1600, 1524, 1494, 1347, 1199, 1139, 1045, 833, 776, 703;
Optical Rotation: $[\alpha]_D^{25}$ = +73.1 (*c* = 1.10, $CHCl_3$, 98% ee); **HPLC:** DAICEL CHIRALPAK ID, hexane/*i*-PrOH = 90/10, flow rate: 1.0 mL/min, λ = 254 nm, t_R(minor) = 7.4 min, t_R(major) = 20.2 min, ee = 98%.

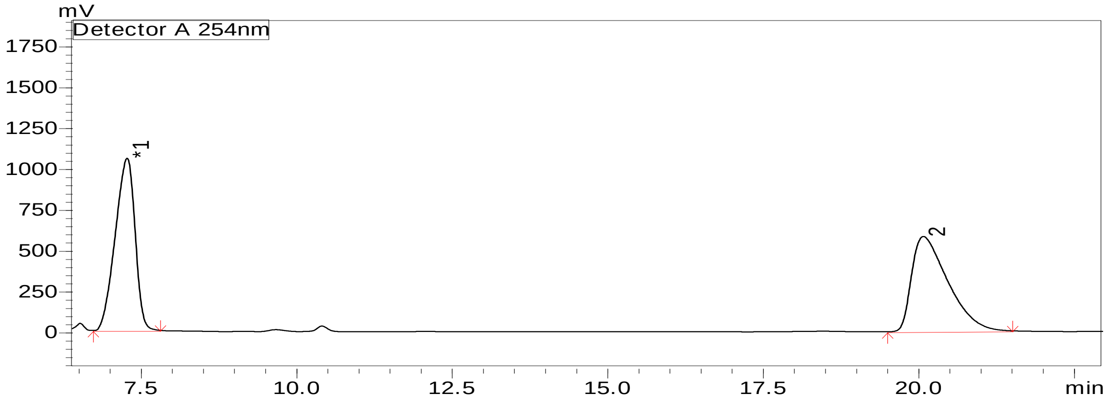

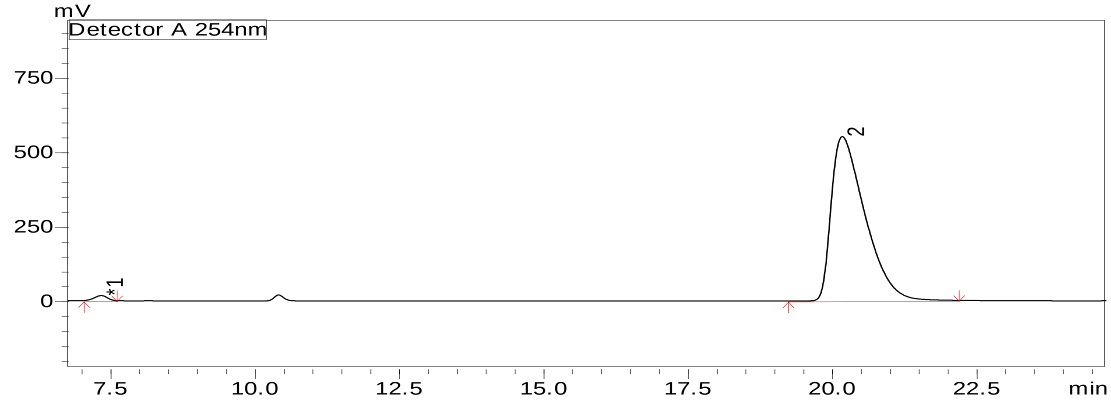

Peak#	Ret. Time	Area%
1	7.294	49.797
2	20.094	50.203

Peak#	Ret. Time	Area%
1	7.355	1.155
2	20.190	98.845

Supplementary Figure 20. HPLC chromatogram for compound **3at**

tert-butyl 3-((1_S_,2_S_)-1-((_tert_-butoxycarbonyl)amino)-2-(4-nitrophenyl)-2-(((_E_)-2,2,2-trifluoro -1-phenylethylidene)amino)ethyl)-1H-indole-1-carboxylate (**3au**)

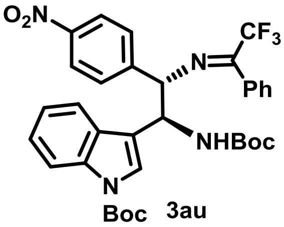

white solid, 88.7 mg (68% yield);
^{1}H NMR (400 MHz, $CDCl_3$) δ 8.12 (d, J = 8.5 Hz, 1H), 8.08 (d, J = 8.5 Hz, 2H), 7.68 (s, 1H), 7.51-7.45 (m, 1H), 7.44-7.34 (m, 4H), 7.31-7.27 (m, 1H), 7.14-7.06 (m, 2H), 6.99-6.91 (m, 2H), 5.40-5.28 (m, 1H), 5.16-5.08 (m, 1H), 4.85 (d, J = 8.0 Hz, 1H), 1.68 (s, 9H), 1.36 (s, 9H); **^{13}C NMR (125 MHz, $CDCl_3$)** δ 160.9 (q, J = 34 Hz), 154.7, 149.4, 147.3, 146.3, 135.0, 130.4, 129.6, 128.9, 128.4, 127.3, 125.2, 124.7, 123.5, 122.7, 120.6, 119.5 (q, J = 277 Hz), 118.6, 116.6, 115.3, 84.0, 80.1, 68.3, 52.9, 28.2, 28.1; **^{19}F NMR (471MHz, $CDCl_3$)** δ -71.05;

HRMS (ESI) m/z [M+H]$^+$: calcd. 653.2581, found. 653.2581;

IR (film): ν_{max} (cm^{-1}) 3400, 2956, 2927, 1737, 1699, 1607, 1526, 1454, 1370, 1347, 1257, 1199, 1158, 1088, 854, 704;

Optical Rotation: $[\alpha]_D^{25}$ = -10.2 (c = 1.00, $CHCl_3$, 98% ee); **HPLC:** DAICEL CHIRALPAK IF-3, hexane/i-PrOH = 95/5 flow rate: 1.0 mL/min, λ = 254 nm, t_R(minor) = 7.6 min, t_R(major) = 9.7 min, ee = 98%.

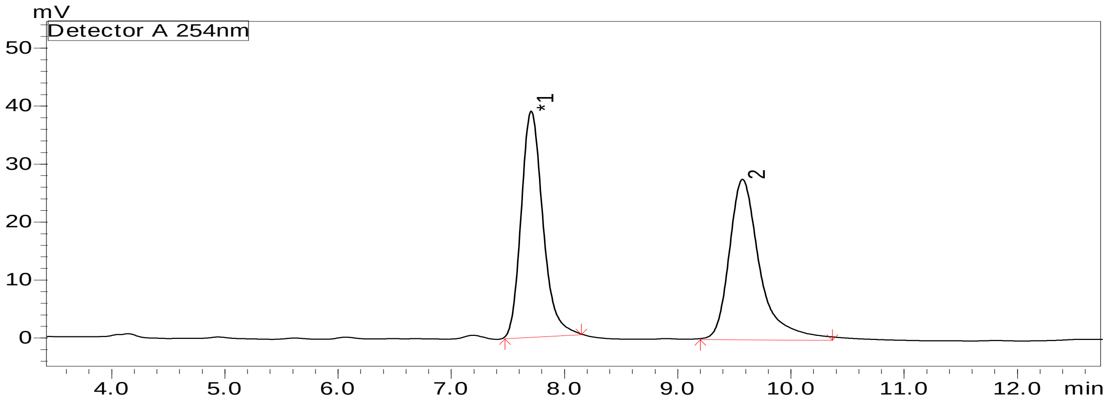

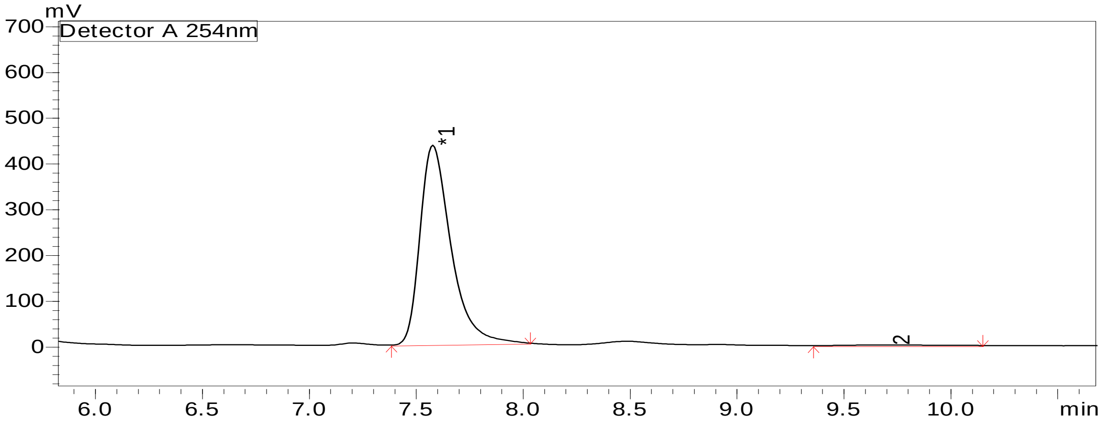

Peak#	Ret. Time	Area%
1	7.717	50.045
2	9.584	49.955

Peak#	Ret. Time	Area%
1	7.583	99.147
2	9.710	0.853

Supplementary Figure 21. HPLC chromatogram for compound **3au**

***tert*-butyl ((1*S*,2*S*)-4-methyl-1-(4-nitrophenyl)-1-(((*E*)-2,2,2-trifluoro-1-phenylethylidene)amino)pentan-2-yl)carbamate** (**3av**)

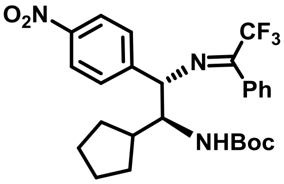

3av

white solid, 66.7 mg (66% yield);
^{1}H NMR (400 MHz, $CDCl_3$) δ 8.17 (d, J = 8.7 Hz, 2H), 7.51-7.44 (m, 1H), 7.42-7.33 (m, 4H), 7.00-6.94 (m, 2H), 5.20 (d, J = 10.4 Hz, 1H), 4.63 (s, 1H), 3.75-3.66 (m, 1H), 1.76-1.65 (m, 2H), 1.61 -1.54 (m, 2H), 1.51-1.42 (m, 3H), 1.36-1.30 (m, 1H), 1.25 (s, 9H), 1.05-0.94 (m, 1H); **^{13}C NMR (125 MHz, $CDCl_3$)** δ 160.8 (q, J = 34 Hz), 155.4, 148.4, 147.1, 130.7, 129.6, 128.8, 127.8, 127.1, 123.6, 119.4 (q, J = 277 Hz), 79.2, 67.3, 60.3, 42.9, 29.7, 29.6, 28.2, 25.4, 25.0; **^{19}F NMR (471MHz, $CDCl_3$)** δ -70.40;
HRMS (ESI) m/z [M+Na]$^+$: calcd. 528.2081, found. 528.2082;
IR (film): ν_{max} (cm^{-1}) 3448, 2956, 2927, 1715, 1600, 1526, 1496, 1347, 1199, 1138, 855, 702;
Optical Rotation: $[\alpha]_D^{25}$ = +143.0 (c = 1.10, $CHCl_3$, 88% ee); **HPLC:** DAICEL CHIRALPAK ID, hexane/*i*-PrOH = 95/5, flow rate: 1.0 mL/min, λ = 254 nm, t_R(minor) = 7.2 min, t_R(major) = 8.4 min, ee = 88%.

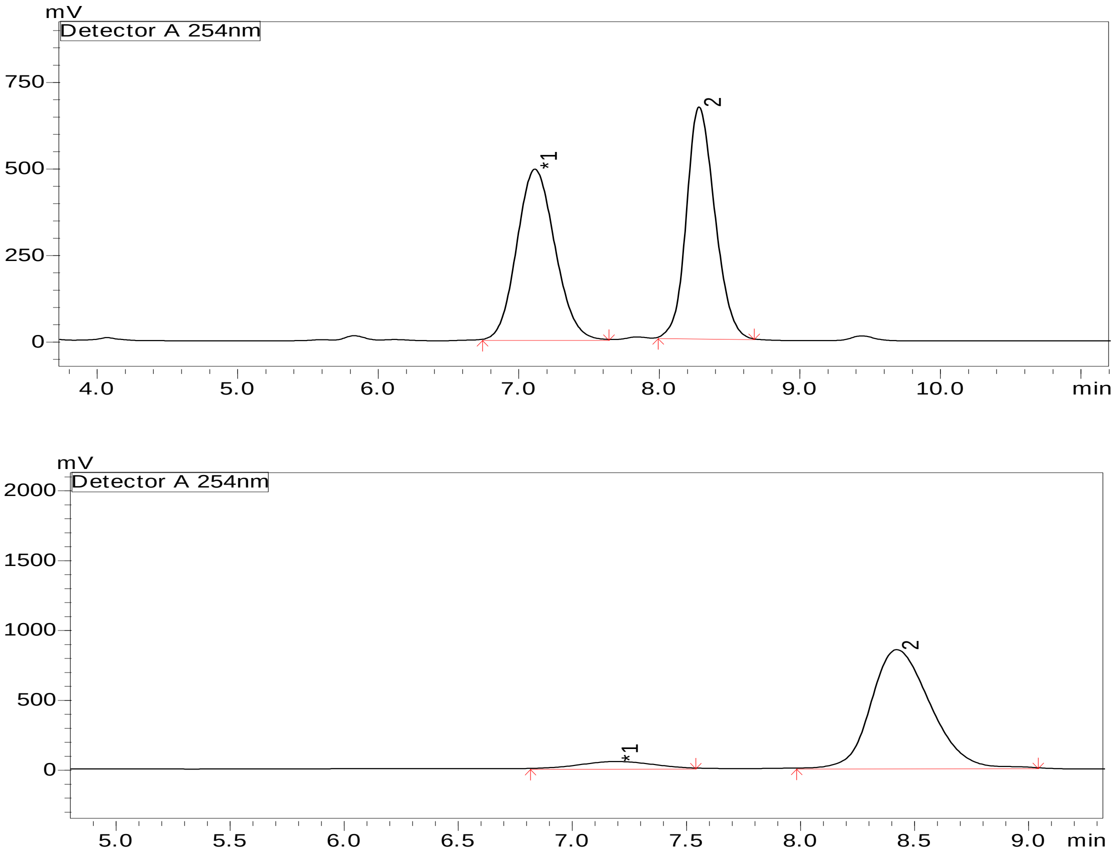

Peak#	Ret. Time	Area%
1	7.124	49.615
2	8.292	50.385

Peak#	Ret. Time	Area%
1	7.197	6.249
2	8.428	93.751

Supplementary Figure 22. HPLC chromatogram for compound **3av**

***tert*-butyl ((1*S*,2*S*)-2-(4-nitrophenyl)-1-(tetrahydro-2H-pyran-4-yl)-2-(((*E*)-2,2,2-trifluoro-1-phenylethylidene)amino)ethyl)carbamate** (**3aw**)

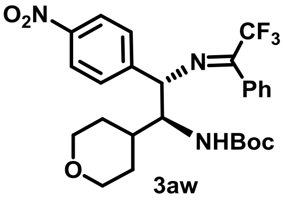

white solid, 73.0 mg (70% yield);
^{1}H NMR (400 MHz, CDCl$_3$) δ 8.22-8.15 (m, 2H), 7.49-7.45 (m, 1H), 7.42-7.34 (m, 4H), 6.95 (d, *J* = 7.4 Hz, 2H), 5.16 (d, *J* = 10.4 Hz, 1H), 4.72 (s, 1H), 3.99-3.92 (m, 1H), 3.89-3.82 (m, 1H), 3.71-3.63 (m, 1H), 3.35-3.17 (m, 2H), 1.68-1.60 (m, 1H), 1.40-1.28 (m, 2H), 1.23 (s, 9H), 1.11-1.05 (m, 2H); **^{13}C NMR (125 MHz, CDCl$_3$)** δ 161.1 (q, *J* = 34 Hz), 155.4, 148.1, 147.2, 131.0, 129.4, 128.9, 127.8, 126.9, 123.7, 119.3 (q, *J* = 277 Hz), 79.4, 67.7, 67.0, 64.7, 60.0, 37.7, 29.8, 29.4, 28.1; **^{19}F NMR (471MHz, CDCl$_3$)** δ -70.22;
HRMS (ESI) m/z [M+H]$^+$: calcd. 522.2210, found. 522.2211;
IR (film): ν_{max} (cm^{-1}) 23442, 956, 2928, 1713, 1600, 1525, 1495, 1346, 1199, 1137, 855, 735, 703;
Optical Rotation: $[\alpha]_D^{25}$ = +138.8 (*c* = 1.20, CHCl$_3$, 98% ee); **HPLC:** DAICEL CHIRALPAK ID, hexane/*i*-PrOH = 90/10, flow rate: 1.0 mL/min, λ = 254 nm, t_R(minor) = 10.6 min, t_R(major) = 18.4 min, ee = 98%.

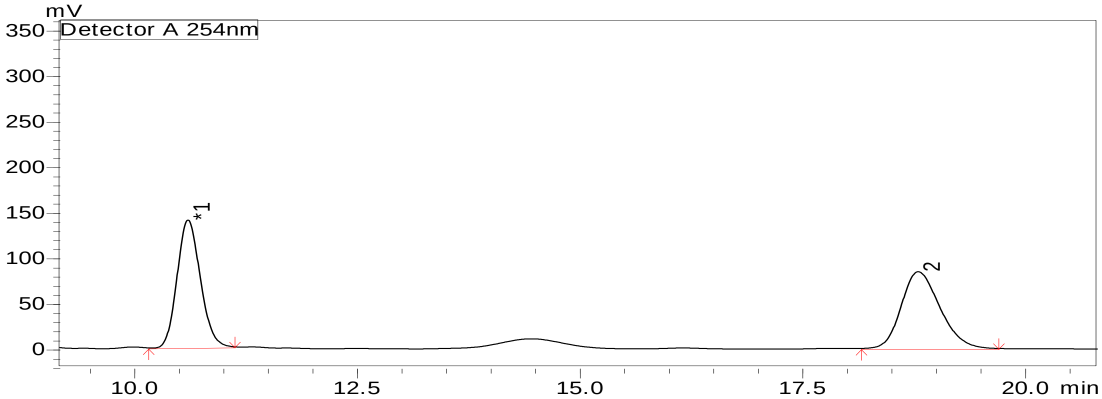

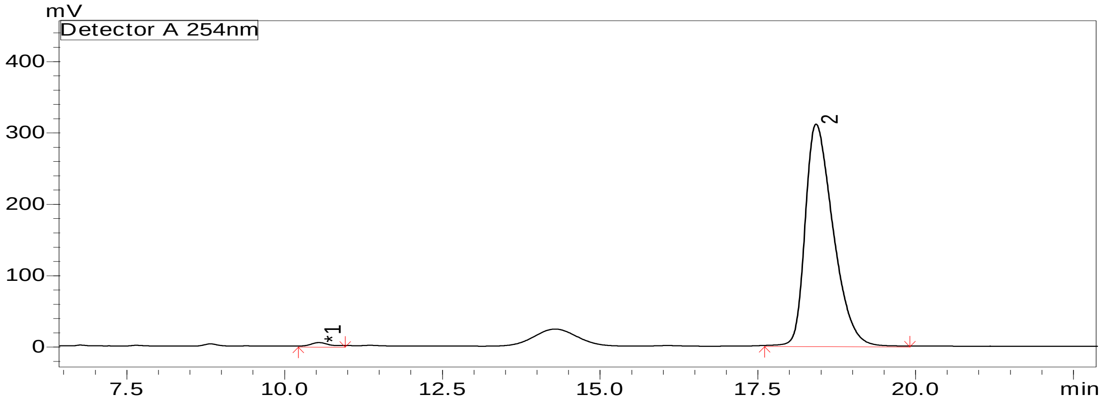

Peak#	Ret. Time	Area%
1	10.612	49.974
2	18.810	50.026

Peak#	Ret. Time	Area%
1	10.565	0.993
2	18.443	99.007

Supplementary Figure 23. HPLC chromatogram for compound **3aw**

***tert*-butyl ((1*S*,2*S*)-3-methyl-1-(4-nitrophenyl)-1-(((*E*)-2,2,2-trifluoro-1-phenylethylidene)amino)butan-2-yl)carbamate** (**3ax**)

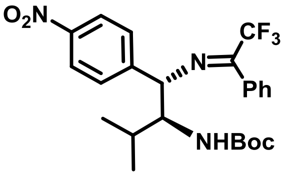

3ax

colorless oil, 61.3 mg (64% yield);

^{1}H NMR (400 MHz, $CDCl_3$) δ 8.18 (d, J = 8.8 Hz, 2H), 7.51-7.45 (m, 1H), 7.43-7.33 (m, 4H), 7.00-6.96 (m, 2H), 5.14 (d, J = 10 Hz, 1H), 4.71 (d, J = 1.8 Hz, 1H), 3.65-3.54 (m, 1H), 1.51-1.40 (m, 1H), 1.26 (s, 9H), 0.89 (d, J = 6.7 Hz, 3H), 0.72 (d, J = 6.7 Hz, 3H); **^{13}C NMR (125 MHz, $CDCl_3$)** δ 160.7 (q, J = 34 Hz), 155.5, 148.6, 147.1, 130.8, 129.6, 128.9, 127.9, 127.1, 123.6, 119.4 (q, J = 277 Hz), 79.2, 65.9, 61.4, 30.7, 28.2, 19.4, 19.2; **^{19}F NMR (471MHz, $CDCl_3$)** δ -70.43;

HRMS (ESI) m/z [M+H]$^+$: calcd. 480.2105, found. 480.2103;

IR (film): ν_{max} (cm^{-1}) 3448, 2961, 2926, 1715, 1601, 1526, 1494, 1370, 1347, 1226, 1198, 1139, 977, 701;

Optical Rotation: $[\alpha]_D^{25}$ = +165.4 (c = 1.50, $CHCl_3$, 89% ee); **HPLC:** DAICEL CHIRALPAK ID, hexane/*i*-PrOH = 19/1, flow rate: 0.5 mL/min, λ = 254 nm, t_R(minor) = 12.8 min, t_R(major) = 13.7 min, ee = 89%.

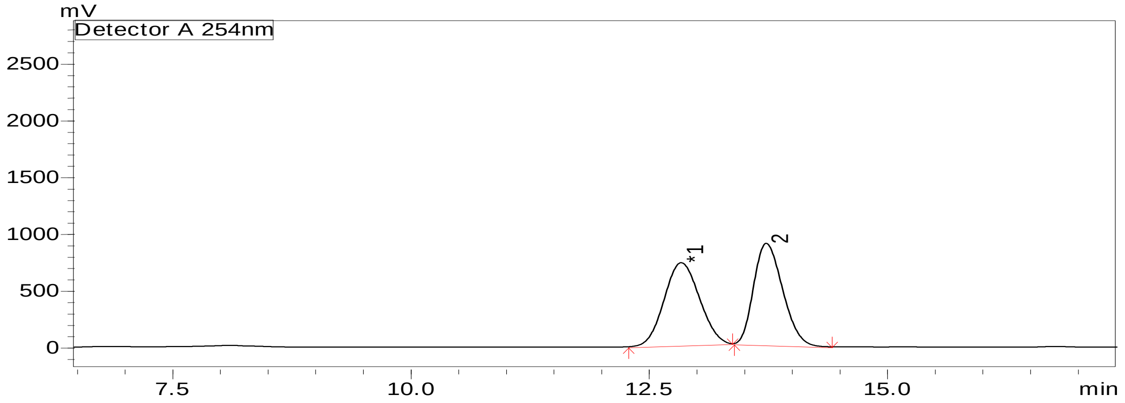

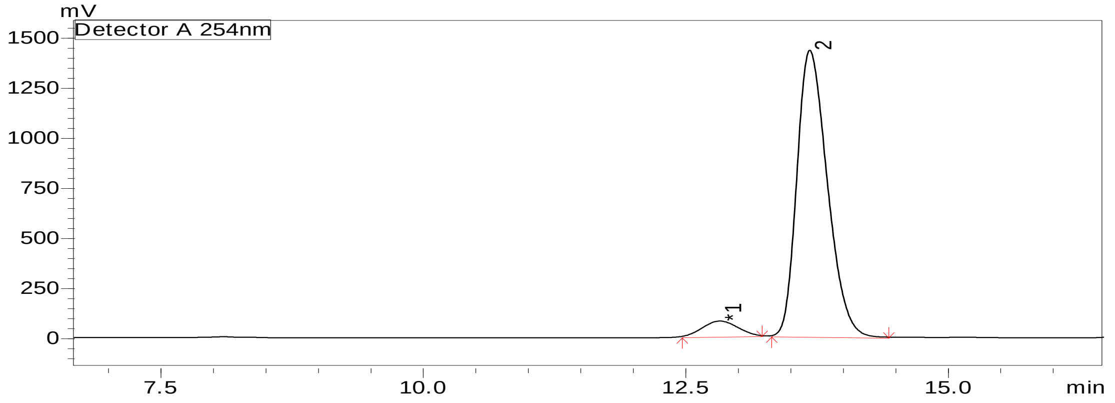

Peak#	Ret. Time	Area%
1	12.848	49.821
2	13.740	50.179

Peak#	Ret. Time	Area%
1	12.836	5.580
2	13.693	94.420

Supplementary Figure 24. HPLC chromatogram for compound **3ax**

***tert*-butyl ((1*S*,2*S*)-4-methyl-1-(4-nitrophenyl)-1-(((*E*)-2,2,2-trifluoro-1-phenylethylidene)amino)pentan-2-yl)carbamate (3ay)**

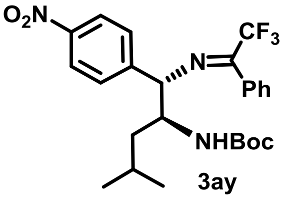

3ay

colorless oil, 64.1 mg (65% yield);

^{1}H NMR (400 MHz, $CDCl_3$) δ 8.16 (d, J = 8.7 Hz, 2H), 7.51-7.45 (m, 1H), 7.43-7.37 (m, 4H), 7.00 (d, J = 7.4 Hz, 2H), 4.92 (d, J = 10 Hz, 1H), 4.49 (d, J = 2.4 Hz, 1H), 4.08-3.96 (m, 1H), 1.57-1.46 (m, 1H), 1.27 (s, 9H), 1.02-0.89 (m, 2H), 0.88-0.80 (m, 6H); **^{13}C NMR (125 MHz, $CDCl_3$)** δ 160.7 (q, J = 34 Hz), 155.1, 147.8, 147.2, 130.6, 129.7 128.9, 128.0, 127.1, 123.6, 119.4 (q, J = 277 Hz), 79.3, 68.6, 53.6, 42.2, 28.1, 24.6, 23.1, 21.8; **^{19}F NMR (471MHz, $CDCl_3$)** δ -70.60;

HRMS (ESI) m/z [M+H]$^+$: calcd. 494.2261, found. 494.2260;

IR (film): ν_{max} (cm^{-1}) 3443, 2958, 2927, 1713, 1600, 1526, 1498, 1346, 1199, 1138, 855, 702;

Optical Rotation: $[\alpha]_D^{25}$ = +118.9 (c = 1.00, $CHCl_3$, 85% ee); **HPLC:** DAICEL CHIRALPAK IF-3, hexane/i-PrOH = 19/1, flow rate: 0.5 mL/min, λ = 254 nm, t_R(minor) = 10.7 min, t_R(major) = 11.8 min, ee = 85%.

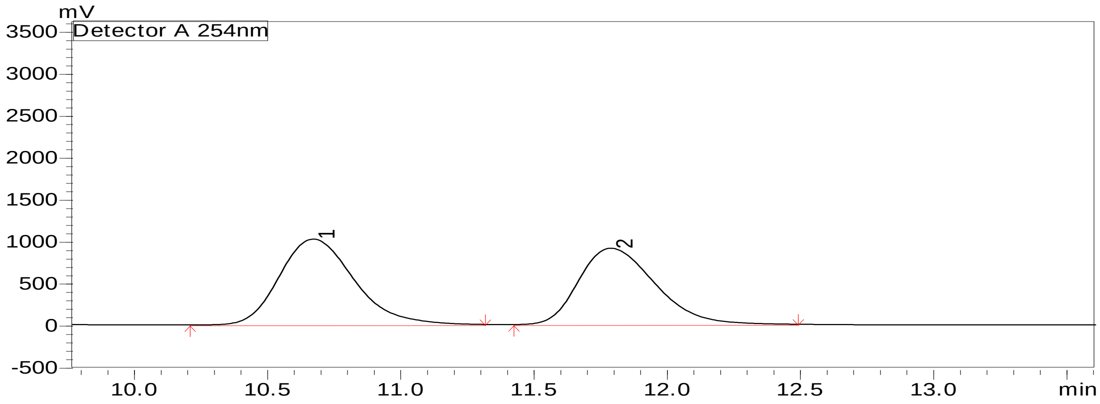

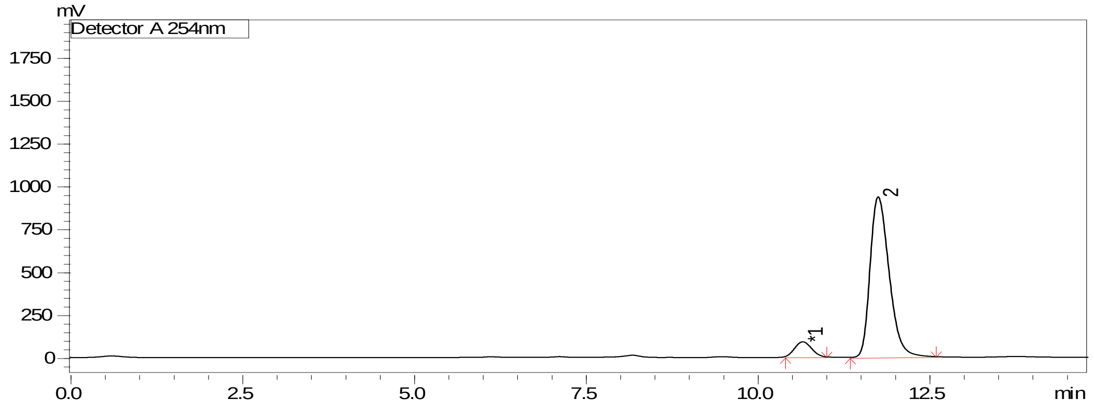

Peak#	Ret. Time	Area%
1	10.676	50.360
2	11.794	49.640

Peak#	Ret. Time	Area%
1	10.668	7.522
2	11.766	92.478

Supplementary Figure 25. HPLC chromatogram for compound **3ay**

***tert*-butyl ((1*S*,2*S*)-2-(2-methyl-4-nitrophenyl)-1-phenyl-2-(((*E*)-2,2,2-trifluoro-1-phenylethylidene)amino)ethyl)carbamate (3ba)**

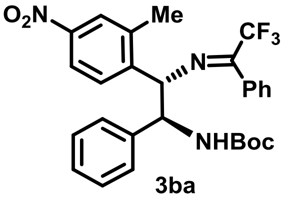

3ba

white solid, 86.5 mg (82% yield);
^{1}H NMR (400 MHz, CDCl$_3$) δ 8.14-8.06 (m, 1H), 8.03-8.00 (m, 1H), 7.80-7.71 (m, 1H), 7.39-7.32 (m, 4H), 7.24-7.19 (m, 2H), 7.12-7.06 (m, 2H), 6.32 (d, J = 7.5 Hz, 2H), 5.95 (d, J = 8.0 Hz, 1H), 5.04 (d, J = 8.0 Hz, 1H), 4.83 (s, 1H), 2.17 (s, 3H), 1.30 (s, 9H); **^{13}C NMR (125 MHz, CDCl$_3$)** δ 161.1 (q, J = 34 Hz), 155.2, 146.9, 145.1, 139.7, 136.2, 130.3, 129.7, 128.8, 128.6, 128.2, 127.7, 127.1, 126.6, 126.3, 119.2 (q, J = 277 Hz), 80.0, 66.6, 57.1, 28.2, 19.1; **^{19}F NMR (471MHz, CDCl$_3$)** δ -71.14;
HRMS (ESI) m/z [M+H]$^+$: calcd. 528.2105, found. 528.2106;
IR (film): ν_{max} (cm^{-1}) 3442, 2977, 2928, 1716, 1611, 1533, 1494, 1353, 1199, 1139, 977, 884, 700;
Optical Rotation: $[\alpha]_D^{25}$ = +5.9 (c = 1.30, CHCl$_3$, 88% ee); **HPLC:** DAICEL CHIRALPAK IF-3, hexane/*i*-PrOH = 19/1, flow rate: 0.5 mL/min, λ = 254 nm, t_R(minor) = 19.7 min, t_R(major) = 22.3 min, ee = 88%.

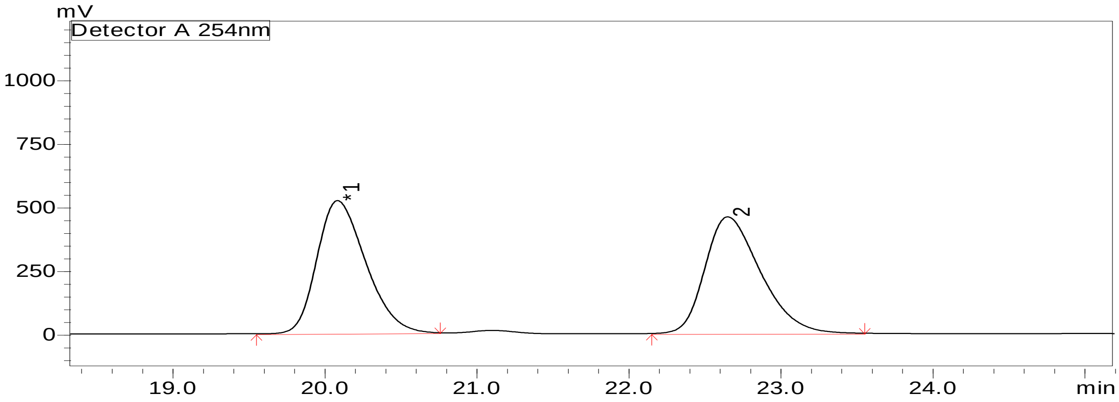

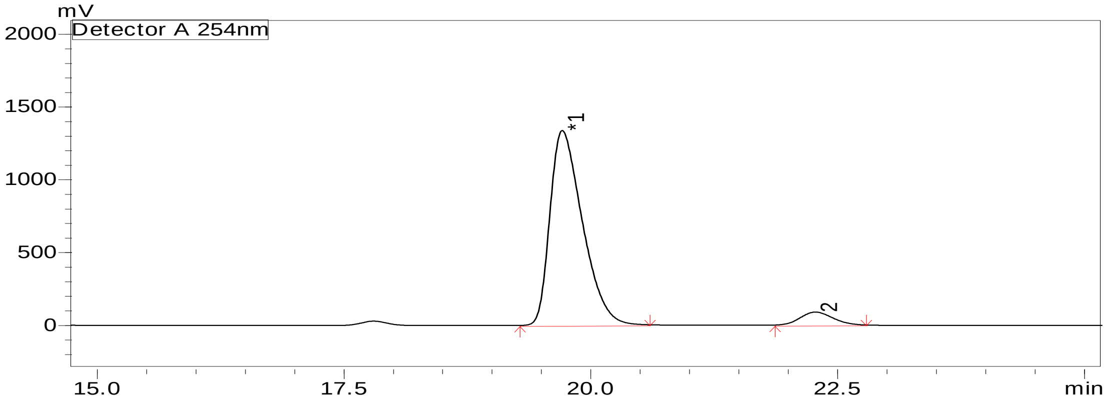

Peak#	Ret. Time	Area%
1	20.091	49.968
2	22.658	50.032

Peak#	Ret. Time	Area%
1	19.724	93.803
2	22.287	6.197

Supplementary Figure 26. HPLC chromatogram for compound **3ba**

***tert*-butyl ((1*S*,2*S*)-2-(2-fluoro-4-nitrophenyl)-1-phenyl-2-(((*E*)-2,2,2-trifluoro-1-phenylethylidene)amino)ethyl)carbamate** (**3ca**)

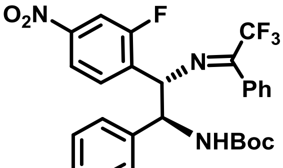

3ca

white solid, 80.7 mg (76% yield);

^{1}H NMR (400 MHz, $CDCl_3$) δ 8.08 (d, J = 8.0 Hz, 1H), 7.94 (d, J = 9.6 Hz, 1H), 7.78 (t, J = 7.5 Hz, 1H), 7.40-7.29 (m, 4H), 7.20 (t, J = 7.7 Hz, 2H), 7.14 -7.06 (m, 2H), 6.29 (d, J = 7.5 Hz, 2H), 5.84 (d, J = 10.0 Hz, 1H), 5.23 (d, J = 10.0 Hz, 1H), 5.01 (s, 1H), 1.29 (s, 9H); **^{13}C NMR (125 MHz, $CDCl_3$)** δ 162.2 (q, J = 34 Hz), 159.0 (d, J = 250 Hz), 155.1, 148.00 (d, J = 8.8 Hz), 139.1, 134.4 (d, J = 12.5 Hz), 130.4, 129.4, 128.9, 128.6, 127.8, 126.6, 126.5, 119.1 (q, J = 278 Hz), 119.2, 111.4 (d, J = 2.8 Hz), 80.0, 63.7, 57.8, 28.2; **^{19}F NMR (471MHz, $CDCl_3$)** δ -71.06, -113.98;

HRMS (ESI) m/z [M+H]$^{+}$: calcd. 532.1854, found. 532.1854;

IR (film): ν_{max} (cm^{-1}) 3438, 2978, 2928, 1718, 1532, 1492, 1352, 1200, 1166, 1140, 976, 811, 741, 701;

Optical Rotation: $[\alpha]_D^{25}$ = +52.1 (c = 1.00, $CHCl_3$, 95% ee); **HPLC:** DAICEL CHIRALPAK ID, hexane/*i*-PrOH = 19/1, flow rate: 0.5 mL/min, λ = 254 nm, t_R(minor) = 14.5 min, t_R(major) = 15.2 min, ee = 95%.

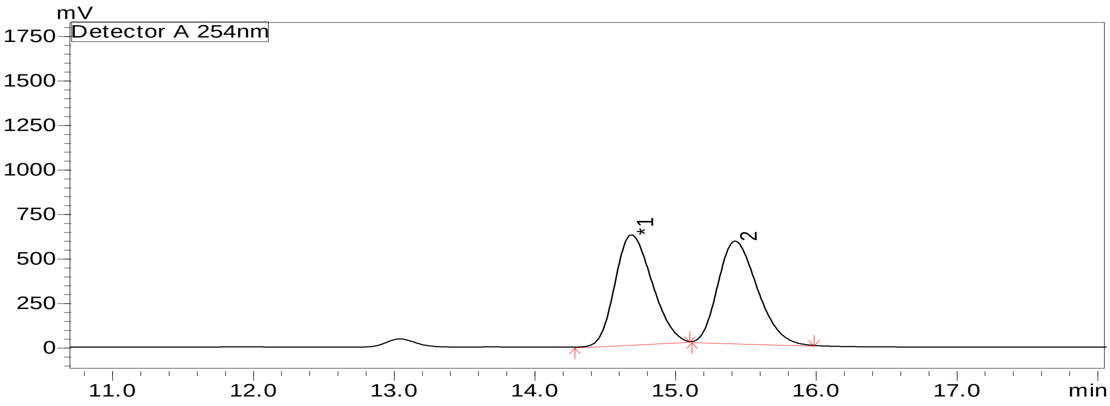

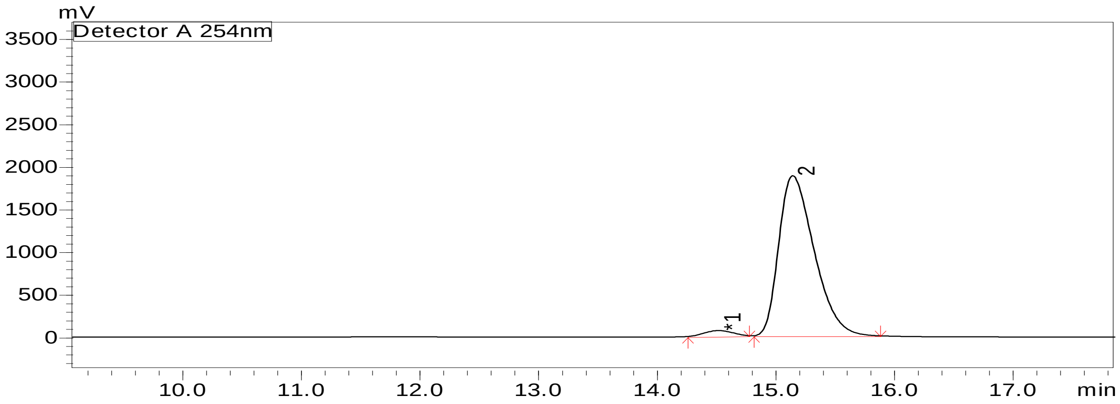

Peak#	Ret. Time	Area%
1	14.696	50.232
2	15.432	49.768

Peak#	Ret. Time	Area%
1	14.527	2.597
2	15.152	97.403

Supplementary Figure 27. HPLC chromatogram for compound **3ca**

***tert*-butyl ((1*S*,2*S*)-2-(2-chloro-4-nitrophenyl)-1-phenyl-2-(((*E*)-2,2,2-trifluoro-1-phenylethylidene)amino)ethyl)carbamate (3da)**

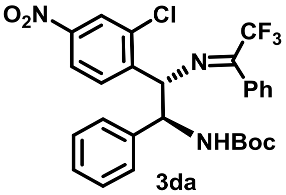

3da

white solid, 85.5 mg (78% yield);
^{1}H NMR (400 MHz, $CDCl_3$) δ 8.30-8.25 (m, 1H), 8.20-8.14 (m, 1H), 7.90-7.82 (m, 1H), 7.39-7.30 (m, 4H), 7.20 (t, J = 7.8 Hz, 2H), 7.15-7.10 (m, 2H), 6.29 (d, J = 7.6 Hz, 2H), 5.88 (d, J = 10.0 Hz, 1H), 5.26 (d, J = 10.0 Hz, 1H), 5.15 (s, 1H), 1.29 (s, 9H); **^{13}C NMR (125 MHz, $CDCl_3$)** δ 161.1 (q, J = 34 Hz), 155.0, 147.4, 144.0, 139.3, 133.2, 130.4, 129.8, 129.1, 128.6, 128.5, 127.7, 126.6 126.3 124.9, 121.6, 119.1 (q, J = 277 Hz), 80.0, 66.9, 56.7, 28.2; **^{19}F NMR (471MHz, $CDCl_3$)** δ -70.96;
HRMS (ESI) m/z [M+Na]$^+$: calcd. 570.1378, found. 570.1374;
IR (film): ν_{max} (cm^{-1}) 3439, 2977, 2929, 1717, 1525, 1493, 1350, 1199, 1166, 1140, 977, 740, 700;
Optical Rotation: $[\alpha]_D^{25}$ = +8.7 (c = 1.30, $CHCl_3$, 94% ee); **HPLC:** DAICEL CHIRALPAK IF-3, hexane/*i*-PrOH = 19/1, flow rate: 0.5 mL/min, λ = 254 nm, t_R(minor) = 13.8 min, t_R(major) = 15.3 min, ee = 94%.

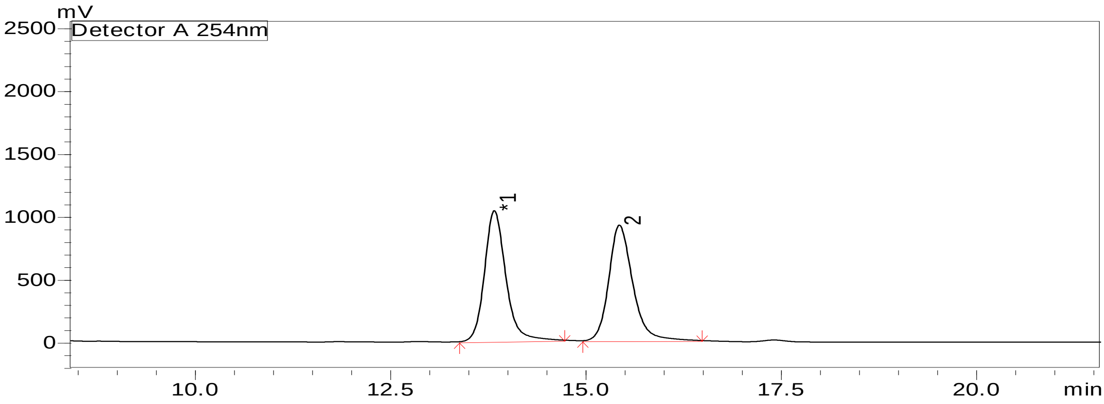

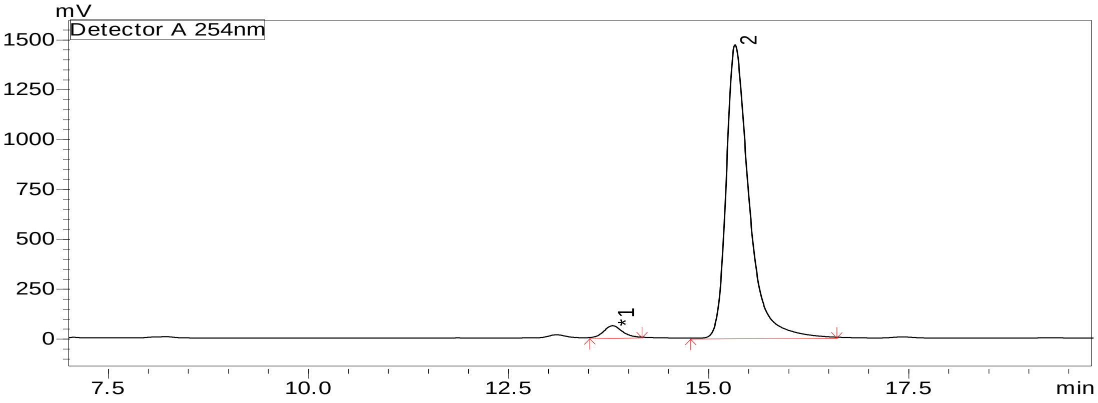

Peak#	Ret. Time	Area%
1	13.842	50.228
2	15.442	49.772

Peak#	Ret. Time	Area%
1	13.815	3.029
2	15.347	96.971

Supplementary Figure 28. HPLC chromatogram for compound **3da**

tert-butyl ((1_S_,2_S_)-2-(3-methyl-4-nitrophenyl)-1-phenyl-2-(((_E_)-2,2,2-trifluoro-1-phenylethyl idene)amino)ethyl)carbamate (**3ea**)

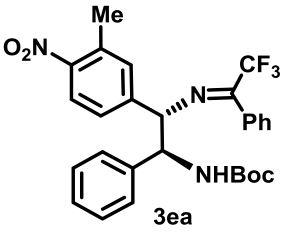

white solid, 80.2 mg (76% yield);
^{1}H NMR (400 MHz, $CDCl_3$) δ 7.98 (d, J = 9.0 Hz, 1H), 7.39-7.29 (m, 6H), 7.20 (t, J = 7.8 Hz, 2H), 7.12-7.07 (m, 2H), 6.31 (d, J = 7.5 Hz, 2H), 5.86 (d, J = 8.6 Hz, 1H), 5.15 (d, J = 8.6 Hz, 1H), 4.65 (s, 1H), 2.63 (s, 3H), 1.31 (s, 9H); **^{13}C NMR (125 MHz, $CDCl_3$)** δ 161.2 (q, J = 34 Hz), 155.1, 148.4, 144.8, 139.6, 134.0, 131.1, 130.2, 129.1, 128.5, 127.6, 126.7, 126.6, 125.4, 125.0, 119.2 (q, J = 277 Hz), 79.9, 69.7, 59.6, 28.1, 20.7; **^{19}F NMR (471MHz, $CDCl_3$)** δ -71.05;
HRMS (ESI) m/z [M+Na]$^+$: calcd. 550.1924, found. 550.1920;
IR (film): ν_{max} (cm^{-1}) 3442, 2971, 2901, 1715, 1611, 1521, 1494, 1393, 1348, 1199, 1050, 838, 702;
Optical Rotation: $[\alpha]_D^{25}$ = +63.2 (c = 1.50, $CHCl_3$, 91% ee); **HPLC:** DAICEL CHIRALPAK ID, hexane/i-PrOH = 95/5, flow rate: 1.0 mL/min, λ = 254 nm, t_R(minor) = 7.6 min, t_R(major) = 16.9 min, ee = 91%.

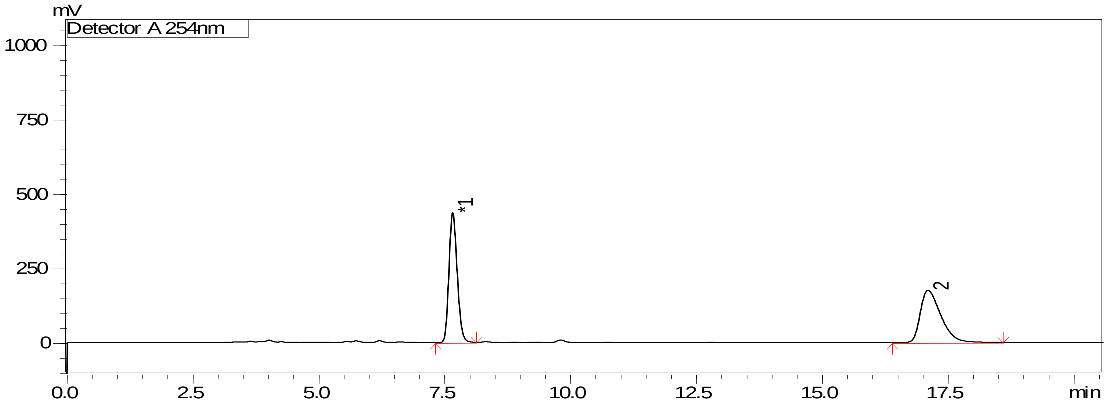

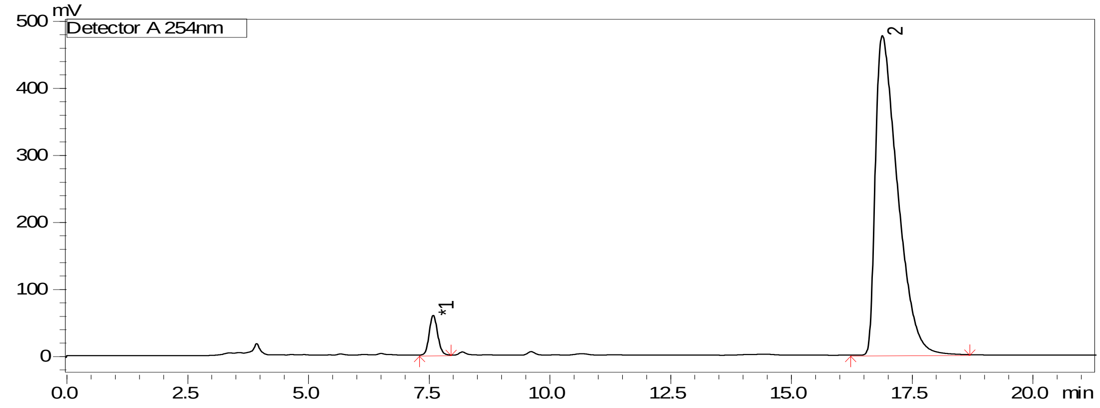

Peak#	Ret. Time	Area%
1	7.682	50.154
2	17.123	49.846

Peak#	Ret. Time	Area%
1	7.609	4.468
2	16.909	95.532

Supplementary Figure 29. HPLC chromatogram for compound **3ea**

***tert*-butyl((1*S*,2*S*)-2-(3-methoxy--4-nitrophenyl)-1-phenyl-2-(((*E*)-2,2,2-trifluoro-1-phenylethylidene)amino)ethyl)carbamate** (**3fa**)

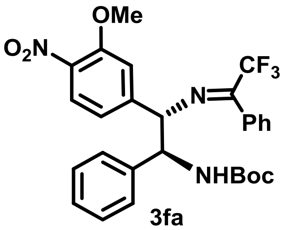

3fa

white solid, 81.4 mg (75% yield);

^{1}H NMR (400 MHz, CDCl$_3$) δ 7.84 (d, *J* = 8.4 Hz, 1H), 7.40-7.33 (m, 4H), 7.22 (t, *J* = 7.8 Hz, 2H), 7.12-7.06 (m, 3H), 6.95 (d, *J* = 8.0 Hz, 1H), 6.35 (d, *J* = 7.5 Hz, 2H), 5.79 (d, *J* = 8.0 Hz, 1H), 5.18 (d, *J* = 8.0 Hz, 1H), 4.64 (s, 1H), 3.98 (s, 3H), 1.32 (s, 9H); **^{13}C NMR (125 MHz, CDCl$_3$)** δ 161.3 (q, *J* = 34 Hz), 155.1, 153.1, 146.3, 139.4, 138.8, 130.3, 128.6, 128.5, 127.7, 126.8, 126.7, 126.0, 119.2 (q, *J* = 278 Hz), 118.8, 112.1, 80.1, 69.8, 59.4, 56.5, 28.2; **^{19}F NMR (471MHz, CDCl$_3$)** δ -71.03;

HRMS (ESI) m/z [M+H]$^+$: calcd. 544.2054, found. 544.2056;

IR (film): ν_{max} (cm^{-1}) 3407, 2978, 2932, 1704, 1609, 1591, 1520, 1495, 1366, 1199, 1138, 1027, 977, 841, 744, 702;

Optical Rotation: $[\alpha]_D^{25}$ = +40.2 (*c* = 1.10, CHCl$_3$, 92% ee); **HPLC:** DAICEL CHIRALPAK ID, hexane/*i*-PrOH = 90/10, flow rate: 1.0 mL/min, λ = 254 nm, t_R(minor) = 7.3 min, t_R(major) = 11.6 min, ee = 92%.

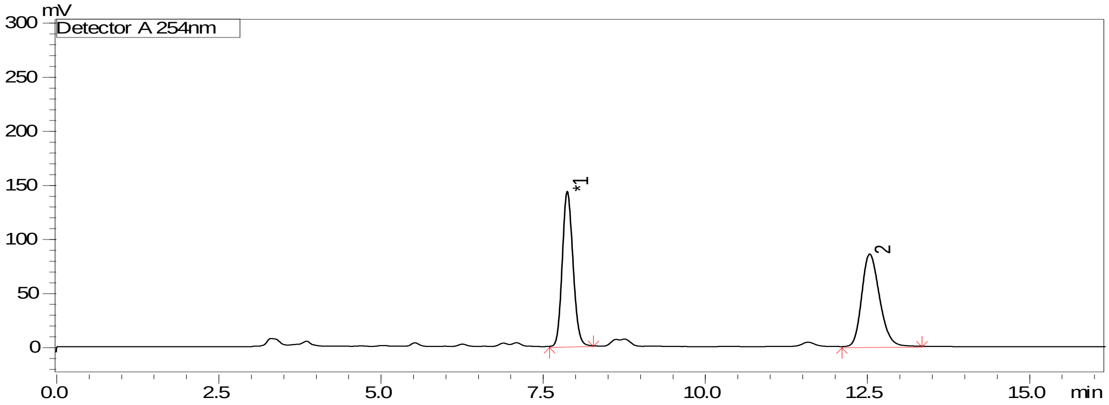

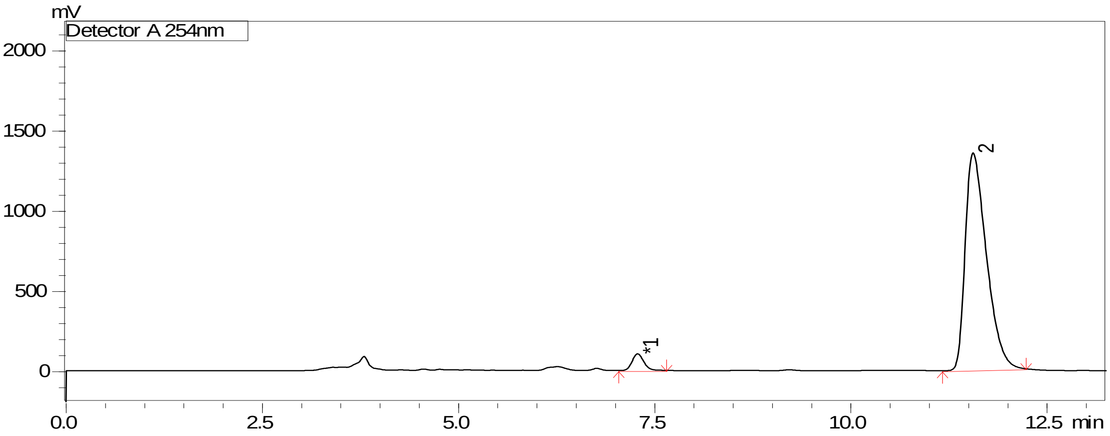

Peak#	Ret. Time	Area%
1	7.892	50.177
2	12.553	49.823

Peak#	Ret. Time	Area%
1	7.298	4.007
2	11.573	95.993

Supplementary Figure 30. HPLC chromatogram for compound **3fa**

***tert*-butyl((1*S*,2*S*)-2-(4-nitro-3-(phenylthio)phenyl)-1-phenyl-2-(((*E*)-2,2,2-trifluoro-1-phenylethylidene)amino)ethyl)carbamate** (**3ga**)

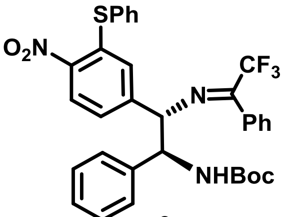

3ga

yellow solid, 89.4 mg (72% yield);
^{1}H NMR (400 MHz, $CDCl_3$) δ 8.23 (d, *J* = 8.5 Hz, 1H), 7.63-7.58 (m, 2H), 7.55-7.50 (m, 3H), 7.32 (t, *J* = 7.6 Hz, 1H), 7.29-7.27 (m, 3H), 7.14 (t, *J* = 7.8 Hz, 3H), 6.99-6.92 (m, 2H), 6.81 (d, *J* = 1.0 Hz, 1H), 6.15 (d, *J* = 8.0 Hz, 2H), 5.58 (d, *J* = 8.0 Hz, 1H), 5.03 (d, *J* = 8.0 Hz, 1H), 4.44 (s, 1H), 1.35 (s, 9H); **^{13}C NMR (125 MHz, $CDCl_3$)** δ 161.1 (q, *J* = 34 Hz), 155.0, 145.2, 144.0, 140.1, 139.4, 135.7, 130.3, 130.2, 128.9, 128.5, 128.4, 127.6, 126.7, 126.6, 126.5, 126.0, 123.3, 118.9 (q, *J* = 278 Hz), 80.0, 69.2, 59.2, 28.2; **^{19}F NMR (471MHz, $CDCl_3$)** δ -71.24;
HRMS (ESI) m/z $[M+H]^+$: calcd. 622.1982, found. 622.1983;
IR (film): ν_{max} (cm^{-1}) 3440, 2961, 2927, 1715, 1594, 1574, 1494, 1337, 1199, 1165, 1138, 1023, 836, 751, 700;
Optical Rotation: $[\alpha]_D^{25}$ = +54.8 (*c* = 1.10, $CHCl_3$, 89% ee); **HPLC:** DAICEL CHIRALPAK ID, hexane/*i*-PrOH = 90/10, flow rate: 1.0 mL/min, λ = 254 nm, t_R(minor) = 11.1 min, t_R(major) = 19.5 min, ee = 89%.

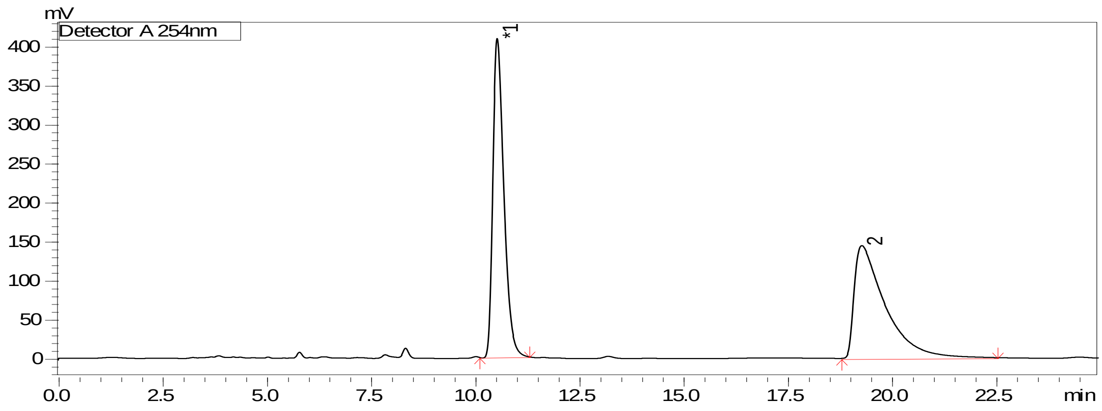

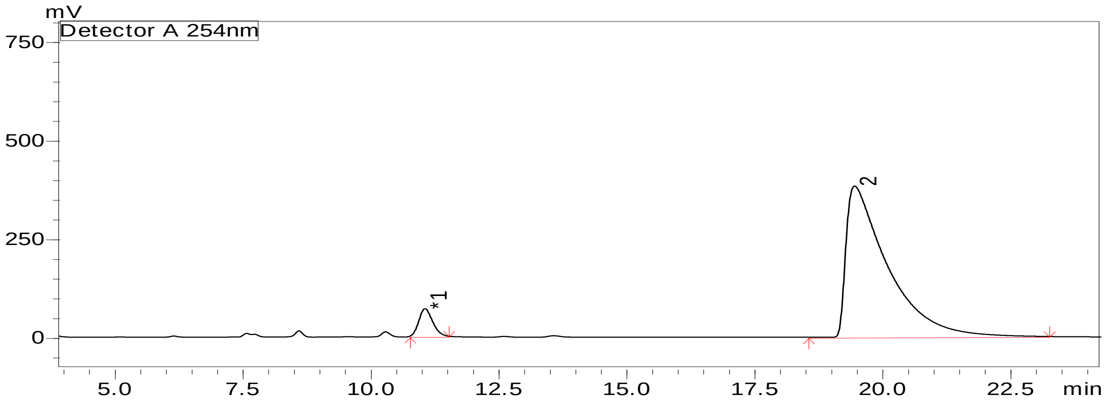

Peak#	Ret. Time	Area%
1	10.545	50.366
2	19.292	49.634

Peak#	Ret. Time	Area%
1	11.079	5.661
2	19.472	94.339

Supplementary Figure 31. HPLC chromatogram for compound **3ga**

tert-butyl ((1_S_,2_S_)-2-(2-nitrophenyl)-1-phenyl-2-(((_E_)-2,2,2-trifluoro-1-phenylethylidene)amino)ethyl)carbamate (**3ha**)

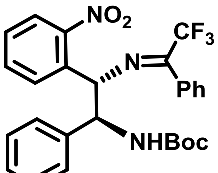

3ha

white solid, 76.9 mg (75% yield);

^{1}H NMR (400 MHz, $CDCl_3$) δ 8.00-7.91 (m, 2H), 7.69-7.62 (m, 1H), 7.50-7.43 (m, 1H), 7.36-7.28 (m, 4H), 7.24-7.19 (m, 2H), 7.18-7.12 (m, 2H), 6.24 (d, J = 7.5 Hz, 2H), 5.98 (d, J = 10.0 Hz, 1H), 5.44 (d, J = 10.0 Hz, 1H), 5.24 (s, 1H), 1.32 (s, 9H); **^{13}C NMR (125 MHz, $CDCl_3$)** δ 161.5 (q, J = 34 Hz), 155.1, 147.8, 139.7, 134.6, 133.1, 130.3, 129.8, 129.2, 128.5, 128.4, 127.4, 126.5, 126.4, 125.0, 119.2 (q, J = 277 Hz), 79.6, 65.4, 57.7, 28.2; **^{19}F NMR (471MHz, $CDCl_3$)** δ -71.05;

HRMS (ESI) m/z $[M+Na]^+$: calcd. 536.1768, found. 536.1765;

IR (film): ν_{max} (cm^{-1}) 3444, 2977, 2928, 1716, 1530, 1494, 1366, 1199, 1138, 1020, 855, 702;

Optical Rotation: $[\alpha]_D^{25}$ = -105.1 (c = 1.20, $CHCl_3$, 91% ee); **HPLC:** DAICEL CHIRALPAK ID, hexane/i-PrOH = 95/5, flow rate: 1.0 mL/min, λ = 254 nm, t_R(minor) = 8.5 min, t_R(major) = 9.9 min, ee = 91%.

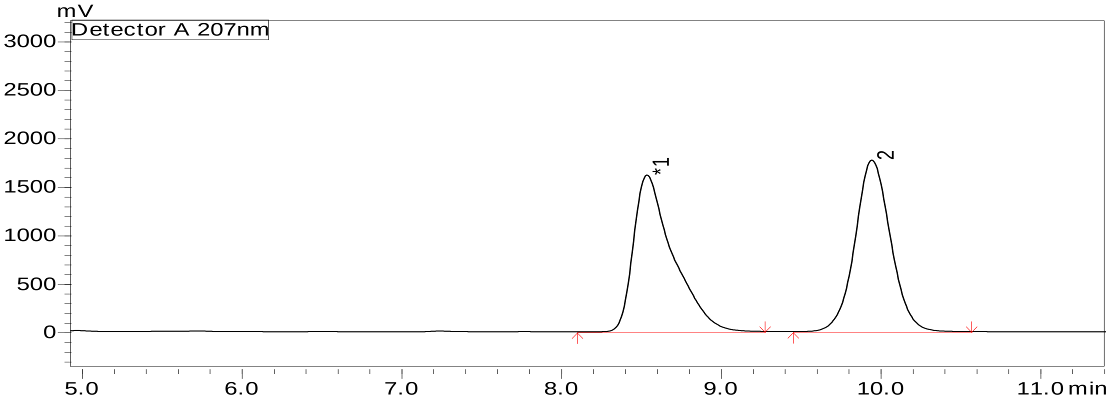

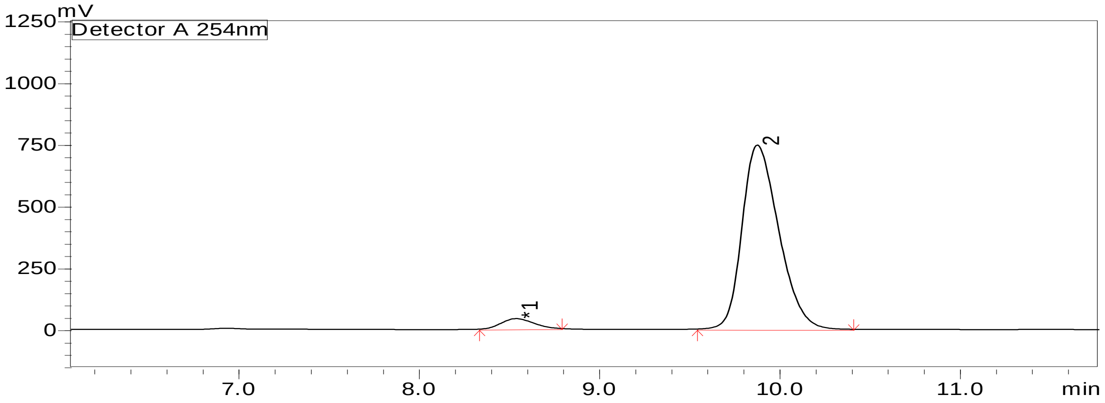

Peak#	Ret. Time	Area%
1	8.543	50.214
2	9.951	49.786

Peak#	Ret. Time	Area%
1	8.544	4.634
2	9.881	95.366

Supplementary Figure 32. HPLC chromatogram for compound **3ha**

tert-butyl ((1S,2S)-2-(4-chloro-2-nitrophenyl)-1-phenyl-2-(((E)-2,2,2-trifluoro-1-phenylethylidene)amino)ethyl)carbamate (3ia)

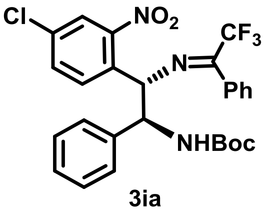

3ia

white solid, 87.6 mg (80% yield);

^{1}H NMR (400 MHz, $CDCl_3$) δ 7.97 (d, *J* = 2.2 Hz, 1H), 7.91 (d, *J* = 8.5 Hz, 1H), 7.70-7.60 (m, 1H), 7.39-7.29 (m, 4H), 7.22-7.14 (m, 4H), 6.24 (d, *J* = 7.5 Hz, 2H), 5.90 (d, J = 10.0 Hz, 1H), 5.40 (d, *J* = 10.0 Hz, 1H), 5.21 (s, 1H), 1.33 (s, 9H); **^{13}C NMR (125 MHz, $CDCl_3$)** δ 161.9 (q, *J* = 34 Hz), 155.1, 148.1, 139.3, 134.4, 133.2, 133.1, 131.1, 130.4, 129.1, 128.6, 128.5, 127.6, 126.5, 126.4, 125.1, 119.1 (q, *J* = 277 Hz), 79.8, 65.1, 57.6, 28.2; **^{19}F NMR (471MHz, $CDCl_3$)** δ -71.08;

HRMS (ESI) m/z [M+H]$^+$: calcd. 548.1558, found. 548.1553;

IR (film): ν_{max} (cm^{-1}) 3437, 2961, 2928, 1699, 1560, 1525, 1491, 1348, 1199, 1167, 806, 701;

Optical Rotation: $[\alpha]_D^{25}$ = -65.6 (*c* = 1.50, $CHCl_3$, 89% ee); **HPLC:** DAICEL CHIRALPAK IC-3, hexane/*i*-PrOH = 24/1, flow rate: 0.5 mL/min, λ = 254 nm, t_R(minor) = 11.3 min, t_R(major) = 23.1 min, ee = 89%.

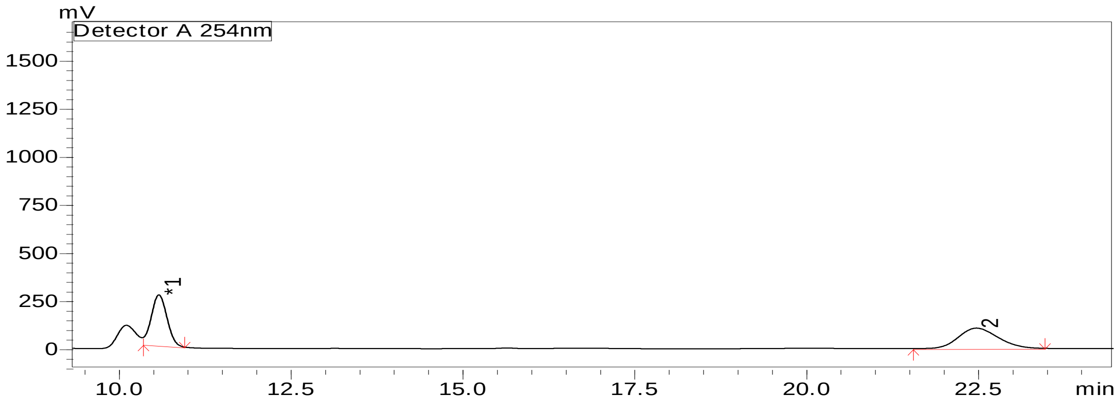

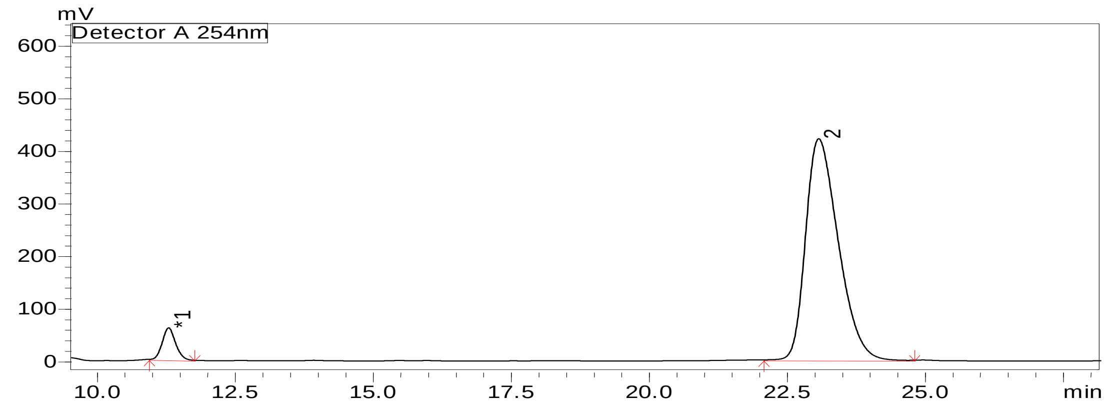

Peak#	Ret. Time	Area%
1	10.595	49.920
2	22.486	50.080

Peak#	Ret. Time	Area%
1	11.314	5.512
2	23.091	94.488

Supplementary Figure 33. HPLC chromatogram for compound **3ia**

***tert*-butyl((1*S*,2*S*)-2-(5-methyl-2-nitrophenyl)-1-phenyl-2-(((*E*)-2,2,2-trifluoro-1-phenylethylidene)amino)ethyl)carbamate (3ja)**

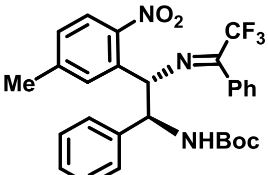

3ja

white solid, 74.9 mg (71% yield); **^{1}H NMR (400 MHz, $CDCl_3$)** δ 7.90 (d, J = 8.0 Hz, 1H), 7.70 (s, 1H), 7.34-7.29 (m, 4H), 7.25-7.11 (m, 5H), 6.24 (d, J = 7.6 Hz, 2H), 5.94 (d, J = 10.0 Hz, 1H), 5.42 (d, J = 10.0 Hz, 1H), 5.27 (s, 1H), 2.50 (s, 3H), 1.32 (s, 9H); **^{13}C NMR (125 MHz, $CDCl_3$)** δ 161.3 (q, J = 34 Hz), 155.1, 145.6, 144.3, 139.8, 134.8, 130.3, 130.2, 129.3, 129.0, 128.5, 128.4, 127.4, 126.6, 126.5, 125.3, 119.2 (q, J = 277 Hz), 79.5, 65.4, 57.6, 28.2, 21.8; **^{19}F NMR (471MHz, $CDCl_3$)** δ -70.96;

HRMS (ESI) m/z [M+H]$^+$: calcd. 528.2105, found. 528.2102;

IR (film): ν_{max} (cm^{-1}) 3442, 2975, 2926, 1721, 1522, 1493, 1339, 1198, 1138, 839, 701;

Optical Rotation: $[\alpha]_D^{25}$ = -130.6 (c = 1.00, $CHCl_3$, 90% ee); **HPLC:** DAICEL CHIRALPAK IF-3, hexane/*i*-PrOH = 95/5, flow rate: 1.0 mL/min, λ = 254 nm, t_R(minor) = 7.7 min, t_R(major) = 11.1 min, ee = 90%.

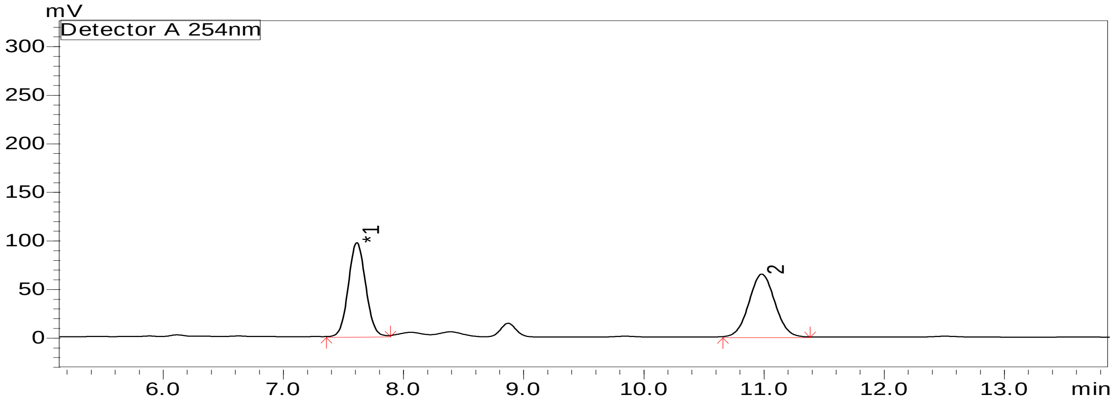

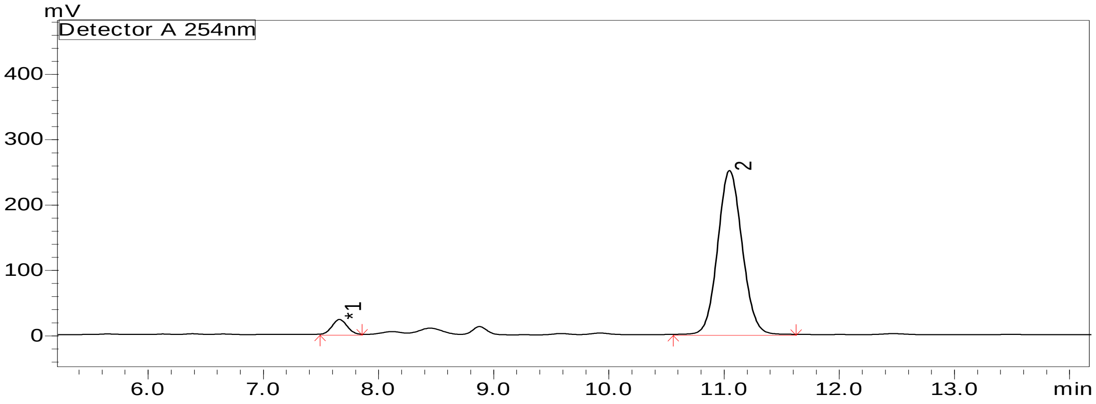

Peak#	Ret. Time	Area%
1	7.621	49.996
2	10.992	50.004

Peak#	Ret. Time	Area%
1	7.672	5.188
2	11.058	94.812

Supplementary Figure 34. HPLC chromatogram for compound **3ja**

The reaction of aldimine 2a with ketimines derivated from other benzylamines

Except for the nitro group, other electron-withdrawing groups, such as 4-COOMe, 4-CF_3, 4-P(O)Ph_2, 4-CN, and 4-SO_2CF_3 were investigated (**Supplementary Table 3** and **Supplementary Table 4**).

Supplementary Table 3. The reaction of aldimine **2a** with ketimines derivated from other benzylamines (mesitylcopper-(*R*,*Rp*)-TANIAPHOS as the catalyst).

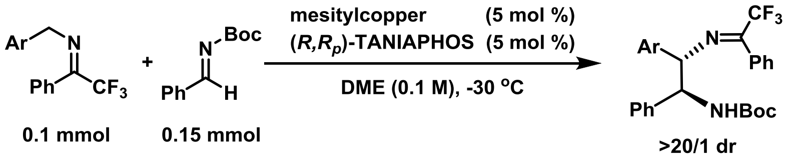

entry	Ar	yield (%)[a]	ee (%)[b]
1	4-COOMe-Ph	trace[c]	-
2	4-CF_3-Ph	trace[c]	-
3	4-P(O)Ph_2-Ph	trace[c]	-
4	4-SO_2CF_3-Ph	53[d]	91
5	4-CN-Ph	15	69

[a]Determined by ^{1}H NMR analysis of reaction crude mixture. [b]Determined by chiral-stationary-phase HPLC analysis. [c]At room temperature. [d]Isolated yield

Supplementary Table 4. The reaction of aldimine **2a** with ketimines derivated from other benzylamines (Cu(CH_3CN)$_4$$PF_6$-(*R*,*Rp*)-TANIAPHOS-Barton's Base as the catalyst).

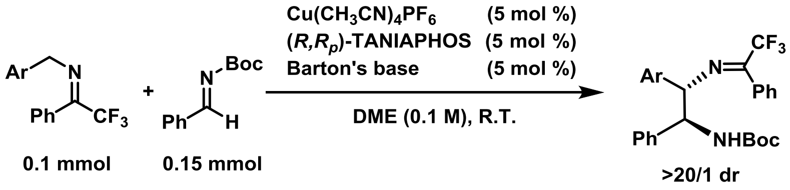

entry	Ar	yield (%)[a]	ee (%)[b]
1	4-COOMe-Ph	21	54
2	4-CF_3-Ph	16	41
3	4-P(O)Ph_2-Ph	10	-
4	4-SO_2CF_3-Ph	51	61
5	4-CN-Ph	39	66

[a]Determined by ^{1}H NMR analysis of reaction crude mixture. [b]Determined by chiral-stationary-phase HPLC analysis.

***tert*-butyl((*1S,2S*)-1-phenyl-2-(((*E*)-2,2,2-trifluoro-1-phenylethylidene)amino)-2-(4-((trifluoro methyl)sulfonyl)phenyl)ethyl)carbamate** (**3ka**)

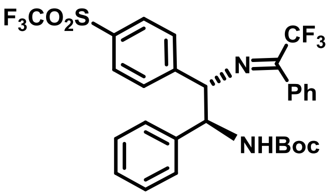

3ka

white solid, 63.3 mg (53% yield);
^{1}H NMR (400 MHz, CDCl$_3$) δ 8.03 (d, *J* = 8.3 Hz, 2H), 7.67(d, *J* = 8.3 Hz, 2H), 7.41-7.31 (m, 4H), 7.20 (t, *J* = 7.7 Hz, 2H), 7.11-7.07 (m, 2H), 6.29 (d, *J* = 7.5 Hz, 2H), 5.83 (d, *J* = 8.8 Hz, 1H), 5.21 (d, *J* = 8.8 Hz, 1H), 4.74 (s, 1H), 1.28 (s, 9H); **^{13}C NMR (100 MHz, CDCl$_3$)** δ 161.8 (q, *J* = 34 Hz), 154.9, 148.9, 139.1, 131.0, 130.6, 130.4, 129.0, 128.63, 128.56, 128.5, 127.8, 126.7, 126.6, 119.7 (q, *J* = 326 Hz), 119.1 (q, *J* = 279 Hz), 80.1, 70.0, 59.5, 28.1; **^{19}F NMR (376MHz, CDCl$_3$)** δ -71.15, -78.34;
HRMS (ESI) m/z [M+H]$^+$: calcd. 601.1590, found. 601.1590;
IR (film): ν_{max} (cm^{-1}) 3444, 3065, 2978, 2932, 1713, 1596, 1494, 1332, 1219, 1200, 1141, 1075, 975, 700, 624;
Optical Rotation: $[\alpha]_D^{25}$ = +52.0 (*c* = 1.22, CHCl$_3$, 91% ee); **HPLC:** DAICEL CHIRALPAK ID, hexane/*i*-PrOH = 90/10, flow rate: 1.0 mL/min, λ = 254 nm, t_R(minor) = 5.0 min, t_R(major) = 8.6 min, ee = 91%.

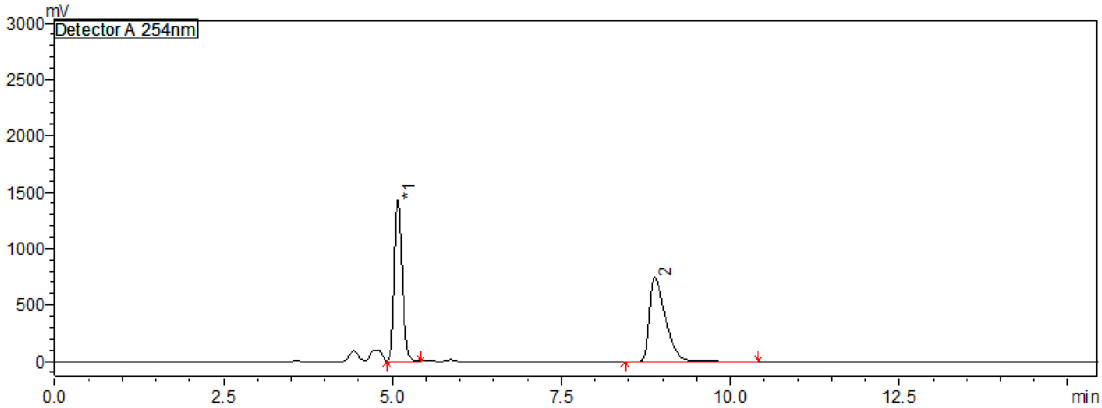

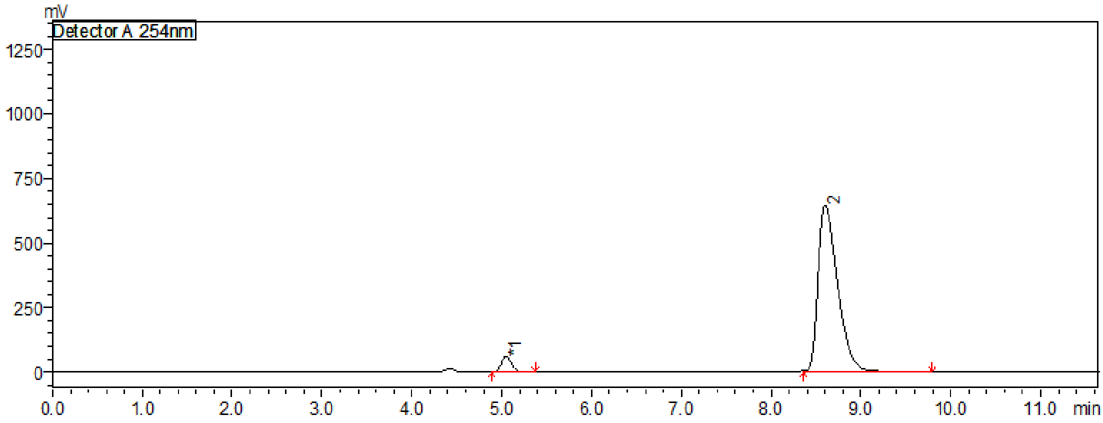

Peak#	Ret. Time	Area%
1	5.079	48.887
2	8.883	51.113

Peak#	Ret. Time	Area%
1	5.048	4.669
2	8.601	95.331

Supplementary Figure 35. HPLC chromatogram for compound **3ka**

Gram-Scale Reaction and Transformations of the Products

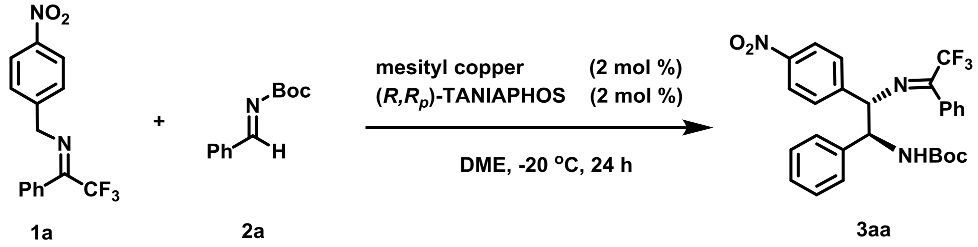

A dried 100 mL schlenk flask equipped with a magnetic stirring bar was charged with mesityl copper (11.0 mg, 0.06 mmol, 2.0 mol %) and (R,R_p)-TANIAPHOS (41.0 mg, 0.06 mmol, 2.0 mol %) in a glove box under Ar atmosphere. Anhydrous DME (30 mL) was added via a syringe. The mixture was stirred for 20 minutes to give a yellow solution. The reaction mixture was cooled down to -20 ℃. Then benzyl imine **1a** (925 mg, 3.0 mmol, 1.0 equiv) and *N*-Boc-aldimine **2a** (924 mg, 4.5 mmol, 1.5 equiv) were added. The resulting reaction mixture was stirred at -20 ℃ for 24 hours. Then the reaction mixture was evaporated to remove volatiles and purified by silica gel column chromatography (petroleum ether/ethyl acetate = 20/1) to afford **3aa** as white solids (1.15 g, 75% yield, >20:1 dr, 96% ee).

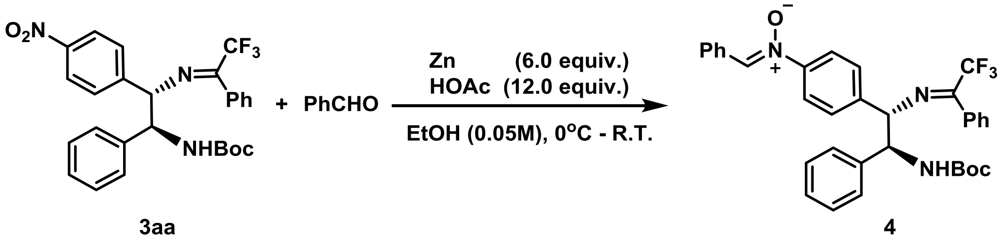

The compounds **4** were prepared using a procedure described in the literature[2] with slight modifications. The diamine (103 mg, 0.2 mmol, 1.0 equiv) and benzaldehyde (41 μl, 0.4 mmol, 2.0 equiv) were dissolved in EtOH (4mL) in a round-bottom 25ml flask. Then, zinc (78 mg, 1.2 mmol, 6.0 equiv) was added. Next, this suspension was cooled to 0 ℃ and glacial acetic acid (137 μl, 2.4 mmol, 12.0 equiv) was added slowly with mechanical stirring. The solution was allowed to reach room temperature and was stirred for an additional 5 h. The precipitate was filtered and washed with DCM. The solvent was evaporated and the residue was purified by flash chromatography (PE/EA= 4/1) to give the product **4** as a yellow solid(90 mg, 77%).

(*Z*)-*N*-(4-((1*S*,2*S*)-2-((*tert*-butoxycarbonyl)amino)-2-phenyl-1-(((*E*)-2,2,2-trifluoro-1-phenylethylidene)amino)ethyl)phenyl)-1-phenylmethanimine oxide (4)

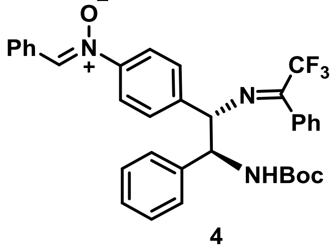

^{1}H NMR (400 MHz, CD_2Cl_2) δ 8.42-8.38 (m, 2H), 7.94 (s, 1H), 7.78 (d, J = 8.5 Hz, 2H), 7.51-7.43 (m, 5H), 7.41-7.31 (m, 4H), 7.23 (t, J = 7.8 Hz, 2H), 7.14-7.11 (m, 2H), 6.39 (d, J = 7.5 Hz, 2H), 5.92 (d, J = 8.7 Hz, 1H), 5.13 (d, J = 8.7 Hz, 1H), 4.70 (s, 1H), 1.32 (s, 9H); **^{13}C NMR (100 MHz, $CDCl_3$)** δ 160.85 (q, J = 34 Hz), 155.2, 148.4, 141.5, 139.9, 134.8, 131.1, 130.5, 130.2, 129.3 129.1, 128.7, 128.5, 128.4, 127.7, 127.6, 126.8, 126.6, 122.0, 119.2 (q, J = 279 Hz), 79.7, 69.7, 59.6, 28.3; **^{19}F NMR (376 MHz, CD_2Cl_2)** δ -72.39; **HRMS (ESI) m/z [M+H]$^+$:** calcd. 588.2469, found. 588.2469; **IR (film):** ν_{max}

(cm^{-1}) 3442, 3062, 2977, 2938, 1712, 1521, 1493, 1427, 1392, 1367, 1330, 1198, 1137, 1019, 976, 893, 702, 618, 513; **Optical Rotation:** $[\alpha]_D^{25}$ = 106.7 (*c* = 0.97, $CHCl_3$).

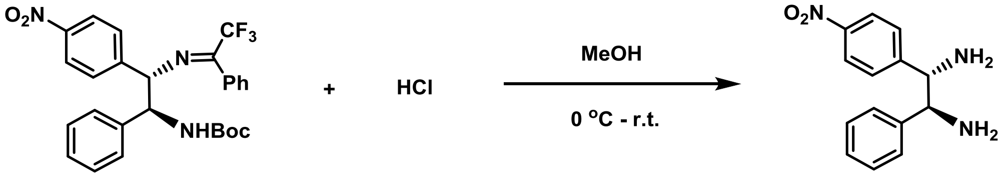

3aa

In a 25 mL schlenk tube, **3aa** (103 mg, 0.2 mmol) was dissolved in dry MeOH (2.0 mL), which was treated with 0.1 mL 12 M HCl at 0 °C. Then the mixture was allowed to stir at room temperature for 3 h. Saturated aqueous $NaHCO_3$ was added dropwise to the aqueous layer to basify the solution untill pH around 9. The aqueous layer was extracted with dichloromethane (3 x 5 mL). The combined organic layers were dried over anhydrous sodium sulfate and concentrated. The resulting residue was purified by flash chromatography (dichloromethane/methanol = 20/1) to provide diamine (48.8 mg, 95%) as brown solids.

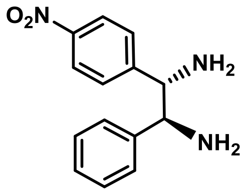

(1*S*,2*S*)-1-(4-nitrophenyl)-2-phenylethane-1,2-diamine

^{1}H NMR (400 MHz, $CDCl_3$) δ 8.10 (d, *J* = 8.8 Hz, 2H), 7.41 (d, *J* = 8.8 Hz, 2H), 7.31-7.25 (m, 2H), 7.25-7.17 (m, 3H), 4.18 (d, *J* = 6.0 Hz, 1H), 4.06 (d, *J* = 6.0 Hz, 1H); **^{13}C NMR (125 MHz, $CDCl_3$)** δ 151.1, 146.9, 142.6, 128.4, 127.9, 127.5, 126.7, 123.3, 62.1, 61.9; **HRMS (ESI) m/z [M+H]$^+$:** calcd. 258.1237, found. 258.1237; **IR (film):** ν_{max} (cm^{-1}) 3373, 2957, 1599, 1519, 1454, 1346, 1075, 854, 756, 700; **Optical Rotation:** $[\alpha]_D^{25}$ = -68.4 (*c* = 1.00, $CHCl_3$).

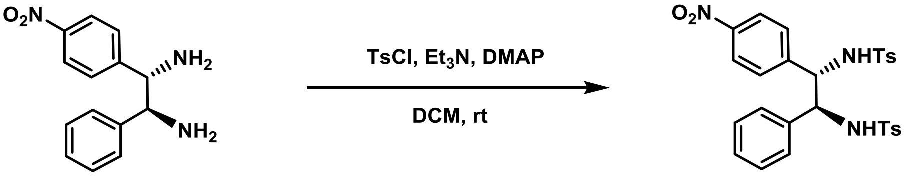

5

The diamine (257 mg, 1.0 mmol, 1.0 equiv) and DMAP (6.1 mg, 0.05 mmol, 0.05 equiv) were dissolved in CH_2Cl_2 (10 mL). The reaction mixture was cooled down to 0 °C. After adding Et_3N (544 µL, 4.0 mmol, 4.0 equiv), the reaction mixture was stirred for further 10 min at 0 °C. Then TsCl (457 mg, 2.4 mmol, 2.4 equiv) was added. The reaction mixture was warmed to room temperature and stirred for 8 h. The reaction was quenched by addition of 1 M HCl. The phases were separated and the aqueous phase was extracted with CH_2Cl_2. The combined organic phases were washed with brine and dried over anhydrous Na_2SO_4. The solvent was evaporated and the residue was purified by flash chromatography (PE/EA= 3/1) to give product **5** (481 mg, 85%) as white solids.

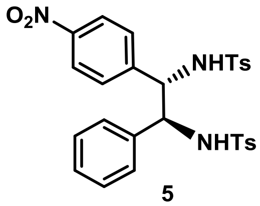

5

***N*,*N*'-((1*S*,2*S*)-1-(4-nitrophenyl)-2-phenylethane-1,2-diyl)bis(4-methyl benzenesulfonamide) (5)**

^{1}H NMR (400 MHz, $CDCl_3$) δ 7.77 (d, *J* = 8.7 Hz, 2H), 7.53-7.43 (m, 4H), 7.12-7.07 (m, 5H), 6.99-6.93 (m, 4H), 6.63 (d, *J* = 7.4 Hz, 2H), 6.22 (d, *J* = 5.9 Hz, 1H), 5.68 (d, *J* = 7.7 Hz, 1H), 4.72-4.67 (m, 1H),

4.43-4.35 (m, 1H), 2.33 (s, 3H), 2.31 (s, 3H); **^{13}C NMR (125 MHz, $CDCl_3$)** δ 147.0, 144.2, 143.7, 143.5, 136.8, 136.6, 135.5, 129.4, 128.7, 128.5 128.2, 127.2, 127.1, 127.0, 123.0, 62.3, 61.8, 21.4; **HRMS (ESI) m/z [M+ NH_4]$^+$:** calcd. 583.1680, found. 583.1681; **IR (film):** ν_{max} (cm^{-1}) 3280, 2955, 2925, 1599, 1521, 1457, 1160, 1088, 928, 814, 701, 668; **Optical Rotation:** $[\alpha]_D^{25}$ = -137.2 (c = 1.00, $CHCl_3$).

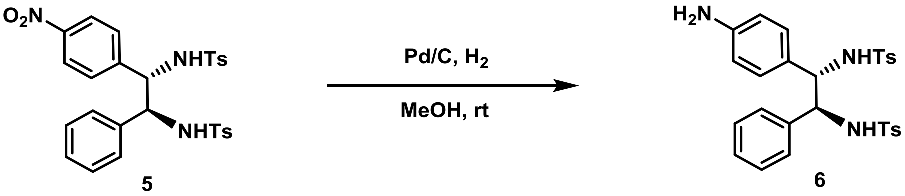

To a round-bottom flask was charged with compound **5** (566 mg, 1.0 mmol), MeOH (10 mL) and Pd/C (57 mg, 10 wt %). The flask was evacuated and backfilled with H_2 for three times. The reaction mixture was allowed to stir at room temperature overnight under H_2 (1 atm). Then the reaction mixture was filtered through a short plug of celite and concentrated under reduced pressure. The crude reaction mixture was purified by flash column chromatography (PE/EA = 2/1) to afford product **6** (525 mg, 98%) as white solids.

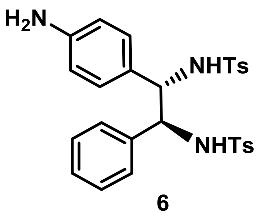

***N,N'*-((1*S*,2*S*)-1-(4-aminophenyl)-2-phenylethane-1,2-diyl)bis(4-methylbenzenesulfonamide) (6)**

^{1}H NMR (400 MHz, $CDCl_3$) δ 7.52 (t, J = 7.4 Hz, 4H), 7.17-7.08 (m 4H), 7.07-7.03 (m, 1H), 7.02-6.95 (m, 2H), 6.70 (d, J = 7.6 Hz, 2H), 6.40 (d, J = 8.0 Hz, 2H), 6.30 (d, J = 8.0 Hz, 2H), 5.38 (d, J = 6.1 Hz, 1H), 5.10 (d, J = 6.1 Hz, 1H), 4.45 (t, J = 7.6 Hz, 1H), 4.28 (t, J = 7.7 Hz, 1H), 2.36 (s, 3H), 2.34 (s, 3H); **^{13}C NMR (125 MHz, $CDCl_3$)** δ 146.0, 143.3, 143.2, 136.9, 136.8, 136.6, 129.4, 129.3, 128.5, 128.0, 127.7, 127.6, 127.2, 125.9, 114.6, 61.9, 61.6, 21.5, 21.4; **HRMS (ESI) m/z [M+ NH_4]$^+$:** calcd. 553.1938, found. 553.1938; **IR (film):** ν_{max} (cm^{-1}) 3315, 2953, 2922, 1519, 1455, 1317, 1154, 1093, 1076, 1056, 932, 809, 696; **Optical Rotation:** $[\alpha]_D^{25}$ = -57.7 (c = 1.00, $CHCl_3$).

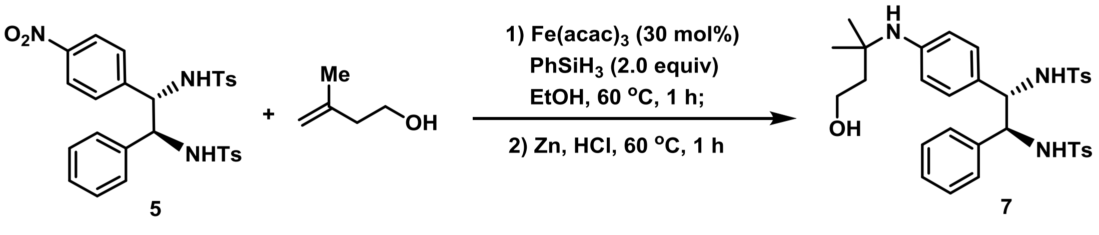

According to a reported procedure[3], to a solution of compound **5** (0.1 mmol, 56.6 mg, 1.0 equiv) and $Fe(acac)_3$ (10.6 mg, 0.03 mmol, 30 mol%) in EtOH (0.50 mL, 0.2 M) were added olefin (0.3 mmol, 30 μL, 3.0 equiv) and $PhSiH_3$ (24.6 μL, 0.2 mmol, 2.0 equiv). The resulting mixture was heated in an oil bath preheated to 60 ℃ with stirring for 1 h. The reaction mixture was then cooled down to room temperature and Zn (130 mg, 2 mmol, 20 equiv) and 2N HCl (1 mL) were added to the reaction mixture. After stirring at 60 ℃ for another 1 h, the reaction mixture was cooled down to room temperature, filtered through Celite and washed with EtOAc. The filtrate was neutralized with sat. $NaHCO_3$ (aq) and extracted with EtOAc three times. The

combined organic layers were washed with brine, dried over anhydrous Na_2SO_4, filtered and concentrated under reduced pressure. The resulting crude product was purified by flash chromatography (DCM/MeOH = 40/1) to furnish **7** (38.4 mg, 59%) as white solids.

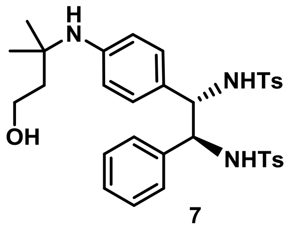

7

***N,N'*-((1*S*,2*S*)-1-(4-((4-hydroxy-2-methylbutan-2-yl)amino)phenyl)-2-phenylethane-1,2-diyl)bis(4-methylbenzenesulfonamide) (7)**

^{1}H NMR (400 MHz, $CDCl_3$) δ 7.50 (dd, *J* = 8.1, 5.4 Hz, 4H), 7.08 (t, *J* = 8.1 Hz, 4H), 7.05-7.00 (m, 1H), 6.94 (t, *J* = 7.5 Hz, 2H), 6.67 (d, *J* = 7.3 Hz, 2H), 6.48 (s, 4H), 5.57 (d, *J* = 6.1 Hz, 1H), 5.47 (br, 1H), 4.48-4.41 (m, 1H), 4.40-4.34 (m, 1H), 3.84 (t, *J* = 5.9 Hz, 2H), 2.32 (s, 6H), 1.83-1.77 (m, 2H), 1.19 (s, 3H), 1.18 (s, 3H); **^{13}C NMR (125 MHz, $CDCl_3$)** δ 143.0, 137.1, 137.0, 136.4, 129.2, 128.1, 127.9, 127.7, 127.5, 127.1, 119.5, 62.1, 61.7, 59.9, 42.5, 29.6, 27.9, 27.8, 21.4; **HRMS (ESI) m/z [M+H]$^+$:** calcd. 622.2404, found. 622.2404; **IR (film):** ν_{max} (cm^{-1}) 3284, 2956, 2925, 1599, 1517, 1457, 1324, 1158, 1092, 1065, 932, 813, 700, 667; **Optical Rotation:** $[\alpha]_D^{25}$ = -11.0 (*c* = 0.90, $CHCl_3$).

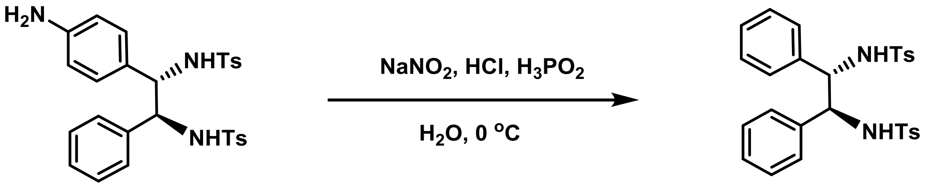

6 8

$NaNO_2$ (17.3 mg, 0.25 mmol, 2.5 equiv) was added to a suspension of **6** (53.6 mg, 0.1 mmol, 1.0 equiv) in 0.25 M HCl (2 mL) at 0 ℃. The reaction mixture was stirred for 10 min at 0 ℃ and then 50 wt % H_3PO_2 (110 μL, 1.0 mmol, 10 equiv) was introduced. The reaction mixture was stirred at 0 ℃ for 24 h. The aqueous layer was extracted with DCM (3 x 5 mL). The combined organic layers were dried with anhydrous Na_2SO_4 and concentrated. The residue was purified by flash column chromatography (PE/EA = 4/1) to afford **8** (41.6 mg, 80%) as white solids. The spectral data was consistent with literature[4].

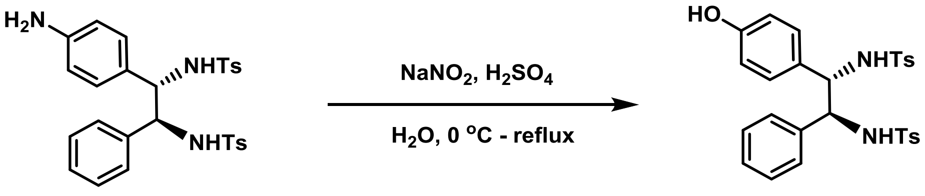

6 9

$NaNO_2$ (10.4 mg, 0.15 mmol, 1.5 equiv) was added to a suspension of **6** (53.6 mg, 0.1 mmol, 1.0 equiv) in 0.25 M H_2SO_4 (2 mL) at 0 ℃. After stirred for 1 h at 0 ℃, the reaction mixture was subsequently stirred at reflux for another 30 min. The reaction mixture was then cooled down to room temperature and extracted with DCM (3 x 5 mL). The combined organic layers were dried with anhydrous Na_2SO_4 and concentrated. The residue was purified by flash column chromatography (PE/EA = 4/1) to afford **9** (39.2 mg, 73%) as white solids.

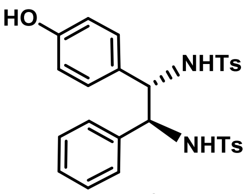

9

***N,N'*-((1*S*,2*S*)-1-(4-hydroxyphenyl)-2-phenylethane-1,2-diyl)bis(4-methylbenzenesulfonamide) (9)**

^{1}H NMR (400 MHz, CDCl$_3$) δ 7.54-7.46 (m, 4H), 7.15-7.02 (m, 5H), 7.00-6.94 (m, 2H), 6.66 (d, *J* = 7.3 Hz, 2H), 6.53 (d, *J* = 8.6 Hz, 2H), 6.43 (d, *J* = 8.6 Hz, 2H), 5.40 (d, *J* = 6.0 Hz, 1H), 5.37 (d, *J* = 6.0 Hz, 1H), 4.93 (br, 1H), 4.44-4.35 (m, 2H), 2.35 (s, 3H), 2.33 (s, 3H); **^{13}C NMR (125 MHz, CDCl$_3$)** δ 155.2, 143.4, 136.7, 136.3, 129.4, 129.3, 128.9, 128.5, 128.2, 127.8, 127.5, 127.2, 115.0, 62.1, 61.5, 21.5, 21.4; **HRMS (ESI) m/z [M+ NH$_4$]$^+$:** calcd. 554.1778, found. 554.1778; **IR (film):** ν_{max} (cm^{-1}) 3281, 2956, 2925, 1599, 1518, 1456, 1322, 1157, 1092, 1076, 929, 812, 701, 667; **Optical Rotation:** $[\alpha]_D^{25}$ = -61.6 (*c* = 1.10, CHCl$_3$).

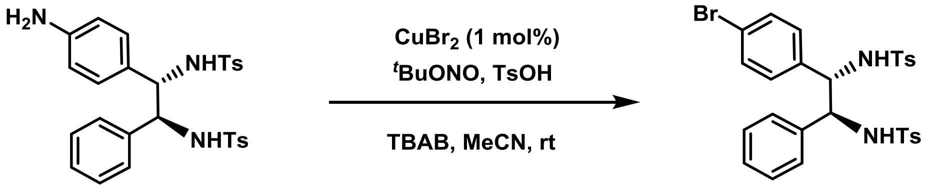

6 10

A dried 25 mL schlenk tube equipped with a magnetic stirring bar was charged with **6** (107 mg, 0.2 mmol, 1.0 equiv), TBAB (129 mg, 0.4 mmol, 2.0 equiv), TsOH (41.3 mg, 0.24 mmol, 1.2 equiv), $CuBr_2$ (0.5 mg, 0.002 mmol, 1.0 mol%) and MeCN (2 mL). Then ^{t}BuONO (29 μL, 0.24 mmol, 1.2 equiv) was added. The resulting reaction mixture was stirred at rt for 12 h. The reaction mixture was then concentrated under reduced pressure and the crude residue was purified by flash chromatography (PE/EA = 4/1) to afford product **10** (89.6 mg, 75%) as white solids.

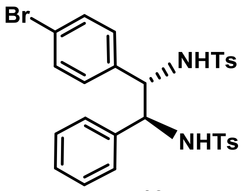

10

***N,N'*-((1*S*,2*S*)-1-(4-bromophenyl)-2-phenylethane-1,2-diyl)bis(4-methylbenzenesulfonamide) (10)**

^{1}H NMR (400 MHz, CDCl$_3$) δ 7.45 (dd, *J* = 8.3, 2.5 Hz, 4H), 7.10-7.00 (m, 7H), 6.96 (t, *J* = 7.4 Hz, 2H), 6.66 (d, *J* = 7.3 Hz, 2H), 6.58 (d, *J* = 8.4 Hz, 2H), 5.85 (d, *J* = 6.6 Hz, 1H), 5.63 (d, *J* = 7.3 Hz, 1H), 4.55-4.48 (m, 1H), 4.43-4.37 (m, 1H), 2.36 (s, 3H), 2.32 (s, 3H); **^{13}C NMR (125 MHz, CDCl$_3$)** δ 143.4, 136.8, 136.6, 135.9, 135.5, 131.1, 129.4, 129.3, 128.3, 128.0, 127.4, 127.1, 121.7, 62.1, 61.7, 21.5, 21.4; **HRMS (ESI) m/z [M+NH$_4$]$^+$:** calcd. 616.0934, found. 616.0933; **IR (film):** ν_{max} (cm^{-1}) 3280, 2955, 2924, 1598, 1489, 1456, 1328, 1162, 1092, 1074, 930, 812, 701, 668; **Optical Rotation:** $[\alpha]_D^{25}$ = -59.0 (*c* = 1.50, CHCl$_3$).

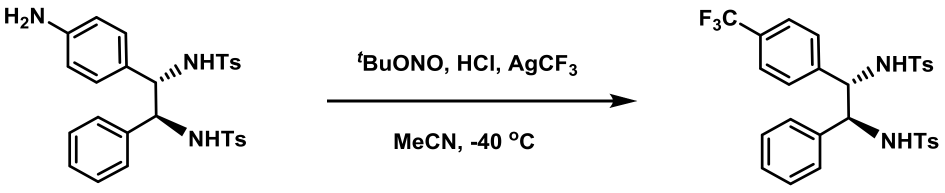

6 11

According to a reported procedure[5], an oven-dried Schlenk tube (A) equipped with a magnetic stir bar was charged with AgF (101.5 mg, 0.8 mmol, 4.0 equiv) and sealed with a septum. The tube was evacuated and backfilled with N_2 for three times before freshly distilled MeCN (2 mL) was added. To the resulting suspension, which was precooled to -78 ^{o}C, was added $TMSCF_3$

(113.8 mg, 0.8 mmol, 4.0 equiv) by a syringe. The mixture was allowed to warm up to room temperature and stirring was continued for an additional 15 min. In due course, AgF solid dissolved and gray dark solution ([$AgCF_3$]) was formed.

Another Schlenk tube (B) equipped with a magnetic stir bar was charged with **6** (107 mg, 0.2 mmol, 1.0 equiv) in freshly distilled MeCN (1.0 mL). To the resulting solution, which was precooled to 0 °C, aq. HCl (12 M, 33 μL, 0.40 mmol, 2.0 equiv) was added and precipitates were formed immediately. After 5 min stirring, ^{t}BuONO (29 μL, 0.22 mmol. 1.1 equiv) was added by a syringe. The mixture was allowed to stir at 0 °C for 15 min. The gray dark solution ([$AgCF_3$]) in Schlenk tube (A), which was precooled to -78 °C, was added over a period of 1 h to Schlenk tube (B) by a syringe at -78 °C. After the addition was complete, the reaction mixture was stirred for 3 h at -78 °C, then was allowed to warm up to room temperature. The stirring was continued at room temperature for an additional 1 h. The reaction mixture was diluted with EtOAc (3 mL) and filtrated through celite. The solvent was removed under reduced pressure, and the residue was purified by flash column chromatography (PE/EA = 4/1) to afford product **11** (63.5 mg, 54%) as white solids.

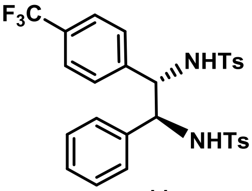

11

***N,N'*-((1*S*,2*S*)-1-phenyl-2-(4-(trifluoromethyl)phenyl)ethane-1,2-diyl) bis(4-methylbenzenesulfonamide) (11)**

^{1}H NMR (400 MHz, $CDCl_3$) δ 7.48-7.39 (m, 4H), 7.13 (d, *J* = 8.1 Hz, 2H), 7.07-6.99 (m, 5H), 6.97-6.92 (m, 2H), 6.83 (d, *J* = 8.1 Hz, 2H), 6.66 (d, *J* = 7.4 Hz, 2H), 6.12 (d, *J* = 6.5 Hz, 1H), 5.77 (d, *J* = 7.6 Hz, 1H), 4.66 (dd, *J* = 9.0, 6.7 Hz, 1H), 4.48-4.42 (m, 1H), 2.30 (s, 6H); **^{13}C NMR (125 MHz, $CDCl_3$)** δ 143.4, 140.5, 136.8, 136.6, 135.7, 129.6 (q, *J* = 33 Hz), 129.3, 129.2, 128.4, 128.2, 128.1, 127.3, 127.1, 127.0, 124.8 (q, *J* = 4 Hz), 62.2, 61.9, 21.4, 21.3; **^{19}F NMR (471MHz, $CDCl_3$)** δ -62.73; **HRMS (ESI) m/z [M+ NH_4]$^+$:** calcd. 606.1703, found. 606.1703; **IR (film):** ν_{max} (cm^{-1}) 3278, 1599, 1456, 1325, 1162, 1124, 1068, 931, 813, 700; **Optical Rotation:** $[\alpha]_D^{25}$ = -59.2 (*c* = 1.10, $CHCl_3$).

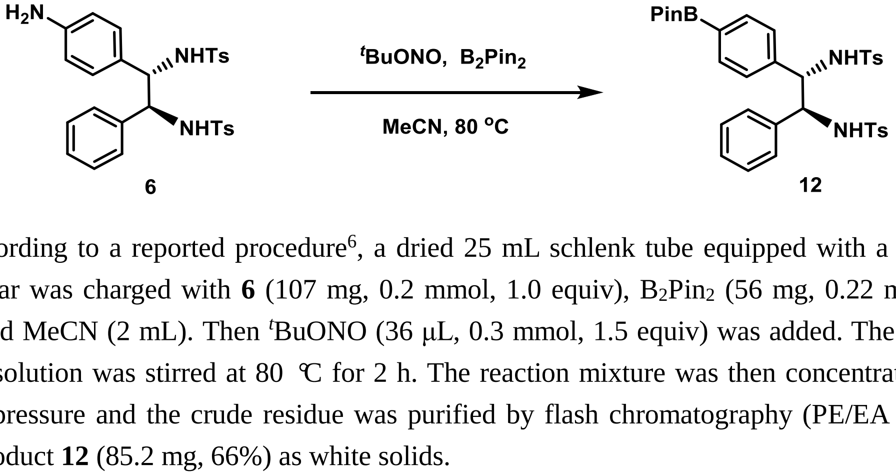

According to a reported procedure[6], a dried 25 mL schlenk tube equipped with a magnetic stirring bar was charged with **6** (107 mg, 0.2 mmol, 1.0 equiv), B_2Pin_2 (56 mg, 0.22 mmol, 1.1 equiv) and MeCN (2 mL). Then ^{t}BuONO (36 μL, 0.3 mmol, 1.5 equiv) was added. The resulting reaction solution was stirred at 80 ℃ for 2 h. The reaction mixture was then concentrated under reduced pressure and the crude residue was purified by flash chromatography (PE/EA = 4/1) to afford product **12** (85.2 mg, 66%) as white solids.

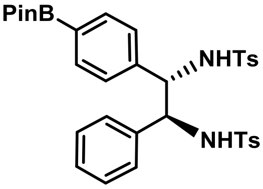

12

***N,N'*-((1*S*,2*S*)-1-phenyl-2-(4-(4,4,5,5-tetramethyl-1,3,2-dioxaborolan-2-yl)phenyl)ethane-1,2-diyl)bis(4-methylbenzenesulfonamide) (12)**

^{1}H NMR (400 MHz, $CDCl_3$) δ 7.53-7.46 (m, 4H), 7.12-7.01 (m, 6H), 6.99-6.93 (m, 3H), 6.68-6.62 (m, 4H), 5.46-5.36 (m, 2H), 4.48-4.42 (m, 2H), 2.33 (s, 6H), 1.25 (s, 12H); **^{13}C NMR (125 MHz, $CDCl_3$)** δ 143.1,

139.2, 137.0, 136.8, 136.3, 136.2, 134.5, 129.3, 128.1, 128.0, 127.7, 127.6, 127.2, 127.0, 83.8, 62.3, 24.9, 24.8, 21.44; **HRMS (ESI) m/z [M+ NH_4]$^+$:** calcd. 663.2717, found. 663.2717; **IR (film):** ν_{max} (cm^{-1}) 3280, 2925, 1600, 1495, 1456, 1400, 1360, 1326, 1161, 1090, 1065, 932, 812, 700; **Optical Rotation:** $[\alpha]_D^{25}$ = -33.7 (*c* = 0.80, $CHCl_3$).

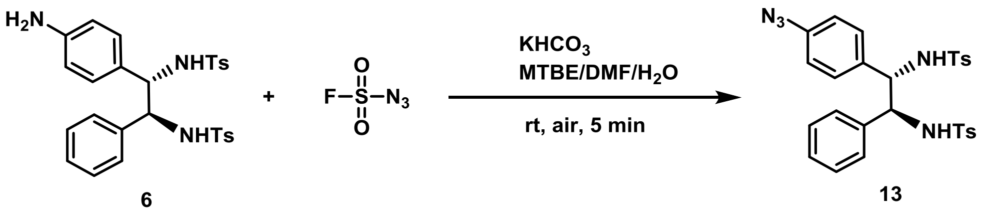

According to a reported procedure[7], to a 25 mL schlenk tube was added sequentially **6** (53.6 mg, 1.0 mmol, 1.0 equiv), 0.25 mL DMF, FSO_2N_3 solution in MTBE (0.4 M, 0.25 ml, containing 0.1 mmol FSO_2N_3) and aqueous $KHCO_3$ solution (3.0 M, 0.133 ml, containing 4.0 mmol $KHCO_3$). The reaction mixture was stirred for 5 min at room temperature and then 3 mL water was added. The aqueous layer was extracted with dichloromethane (3 x 5 mL). The combined organic layers were dried over anhydrous sodium sulfate and concentrated. The resulting residue was purified by flash chromatography (PE/EA = 3/1) to provide product **13** (52.0 mg, 93%) as brown solids.

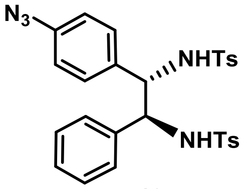

13

***N,N'*-((1*S*,2*S*)-1-(4-azidophenyl)-2-phenylethane-1,2-diyl)bis(4-methyl benzenesulfonamide) (13)**

^{1}H NMR (400 MHz, $CDCl_3$) δ 7.52-7.44 (m, 4H), 7.13-7.02 (m, 5H), 6.97 (t, *J* = 7.5 Hz, 2H), 6.71-6.63 (m, 4H), 6.59 (d, *J* = 8.5 Hz, 2H), 5.71 (d, *J* = 6.4 Hz, 1H), 5.52 (d, *J* = 7.2 Hz, 1H), 4.53-4.47 (m, 1H), 4.43-4.36 (m, 1H), 2.35 (s, 3H), 2.32 (s, 3H); **^{13}C NMR (125 MHz, $CDCl_3$)** δ 143.4, 143.3, 139.5, 136.9, 136.7, 136.1, 133.2, 129.4, 129.1, 128.3, 127.9, 127.4, 127.2, 127.1, 118.6, 62.2, 61.6, 21.44; **HRMS (ESI) m/z [M+NH_4]$^+$:** calcd. 579.1843, found. 579.1843; **IR (film):** ν_{max} (cm^{-1}) 3279, 2956, 2925, 2115, 1606, 1507, 1457, 1324, 1290, 1161, 927, 812; **Optical Rotation:** $[\alpha]_D^{25}$ = -48.9 (*c* = 1.00, $CHCl_3$).

Determination of the Absolute Configurations of the Products

***tert*-butyl((1*S*,2*S*)-1-(4-(methylthio)phenyl)-2-(4-nitrophenyl)-2-(((*E*)-2,2,2-trifluoro-1-phenyl ethylidene)amino)ethyl)carbamate (3ai)**

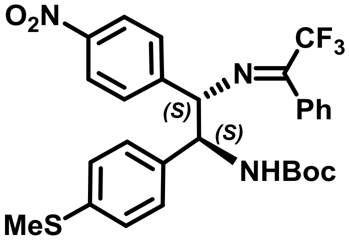

A solution of **3ai** in hexane/ethyl acetate in NMR tube was left at room temperature to grow single crystals. The absolute configuration of **3ai** was determined by X-ray analysis of its single crystal. CCDC 1948086 contains the supplementary crystallographic data for **3ai**. The absolute configurations of other products were deduced by analogy.

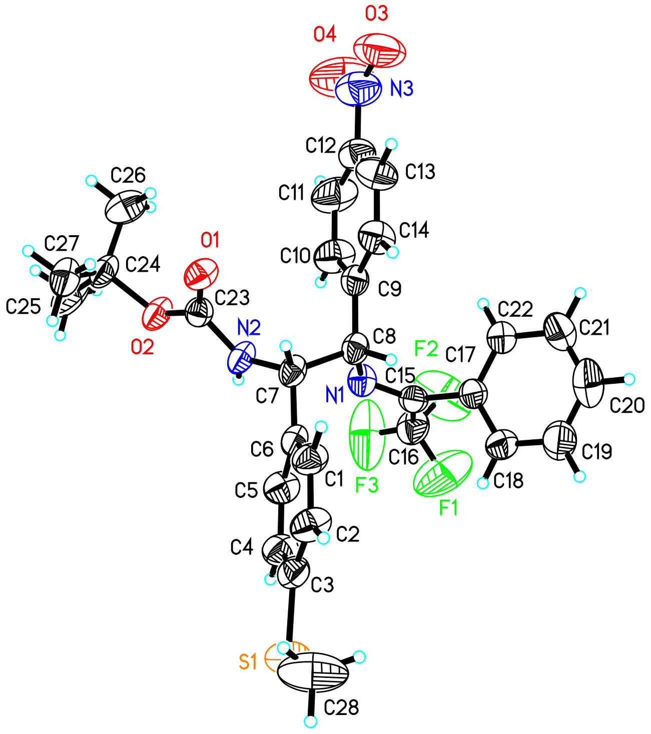

Supplementary Figure 36. The full numbering scheme of CCDC: 194808

Supplementary Table 5. Crystal data and structure refinement for mo_d8v19781_0m.

Identification code	mo_d8v19781_0m	
Empirical formula	C28 H28 F3 N3 O4 S	
Formula weight	559.59	
Temperature	293(2) K	
Wavelength	0.71073 Å	
Crystal system	Monoclinic	
Space group	P 21	
Unit cell dimensions	a = 11.2185(12) Å	a= 90°.
	b = 14.7974(14) Å	b= 96.448(3)°.
	c = 17.6386(16) Å	g = 90°.
Volume	2909.6(5) $Å^3$	
Z	4	
Density (calculated)	1.277 Mg/m^3	
Absorption coefficient	0.167 mm^{-1}	
F(000)	1168	
Crystal size	0.170 x 0.150 x 0.100 mm^3	
Theta range for data collection	2.324 to 25.498°.	
Index ranges	-13<=h<=13, -17<=k<=17, -21<=l<=21	
Reflections collected	37089	
Independent reflections	10436 [R(int) = 0.0725]	
Completeness to theta = 25.242°	99.7 %	
Absorption correction	Semi-empirical from equivalents	
Max. and min. transmission	0.7456 and 0.5928	
Refinement method	Full-matrix least-squares on F^2	
Data / restraints / parameters	10436 / 1 / 712	
Goodness-of-fit on F^2	1.006	
Final R indices [I>2sigma(I)]	R1 = 0.0613, wR2 = 0.1475	
R indices (all data)	R1 = 0.1247, wR2 = 0.1904	
Absolute structure parameter	-0.02(5)	
Extinction coefficient	0.021(3)	
Largest diff. peak and hole	0.255 and -0.190 e.$Å^{-3}$	

Supplementary Figures of NMR Spectra

Note: The major diastereoisomer was inseparable from the minor diastereoisomer by flash column chromatography. Thus the signals of minor diastereoisomer appeared in the NMR spectra, which were not from the impurities.

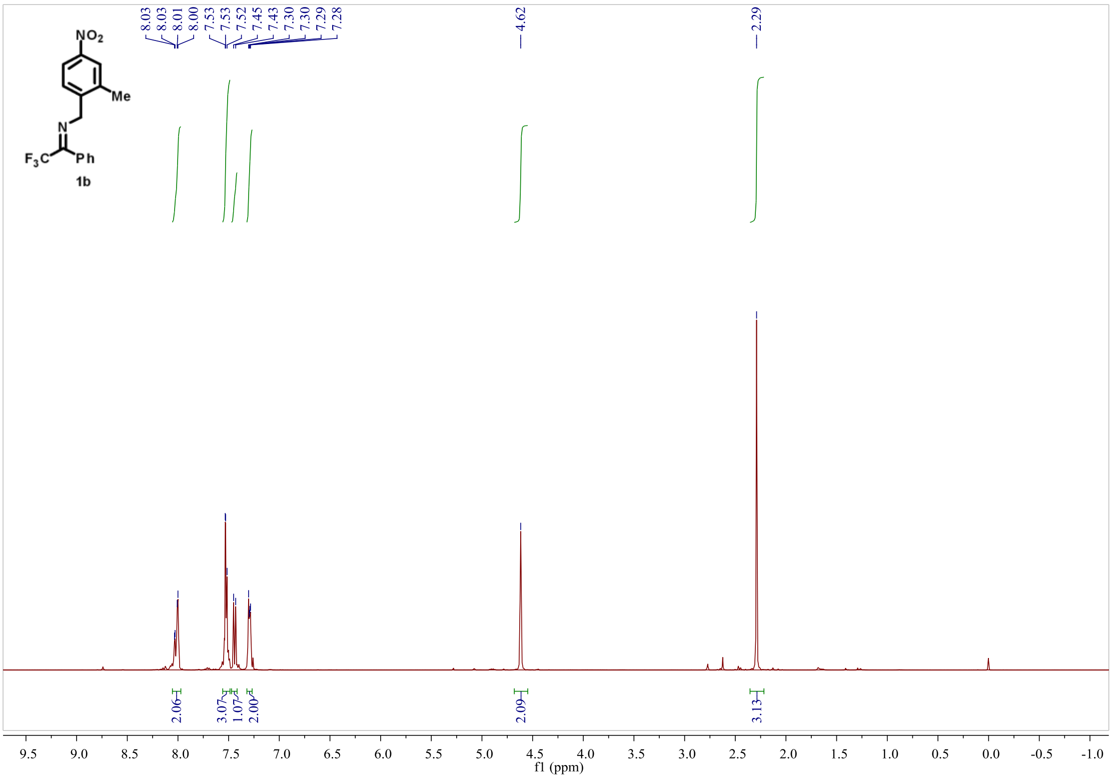

Supplementary Figure 37. ^{1}H NMR spectrum for compound **1b**

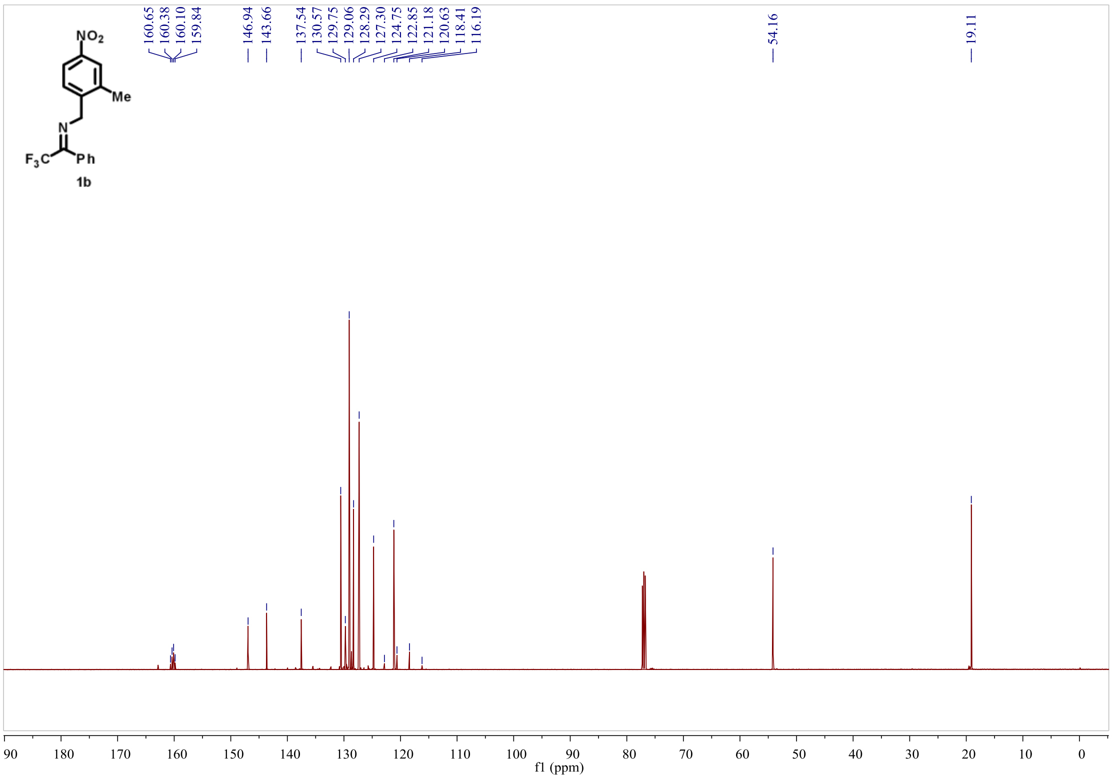

Supplementary Figure 38. ^{13}C NMR spectrum for compound **1b**

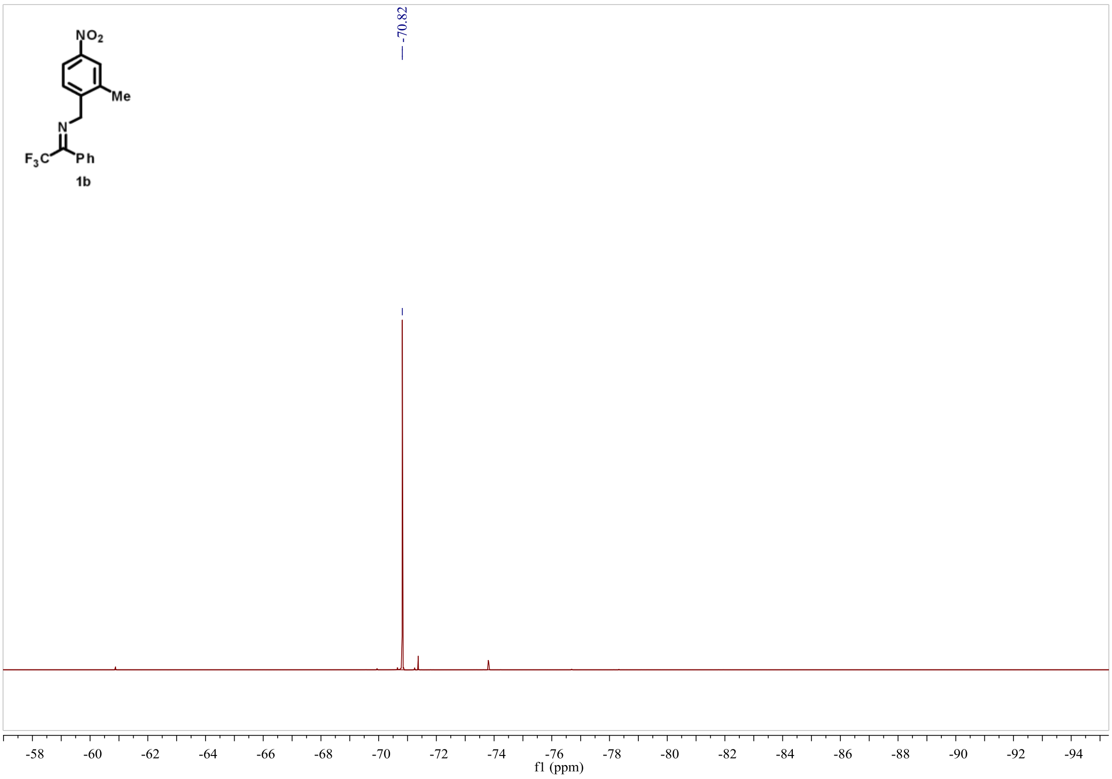

Supplementary Figure 39. ^{19}F NMR spectrum for compound **1b**

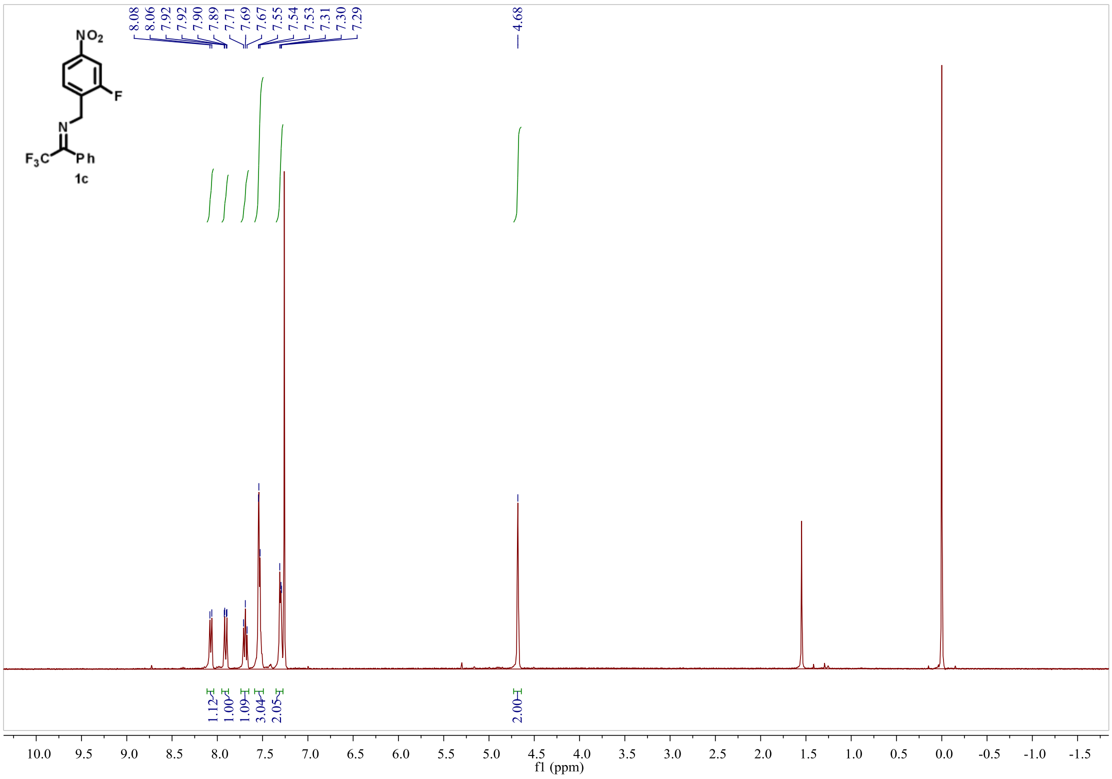

Supplementary Figure 40. ^{1}H NMR spectrum for compound **1c**

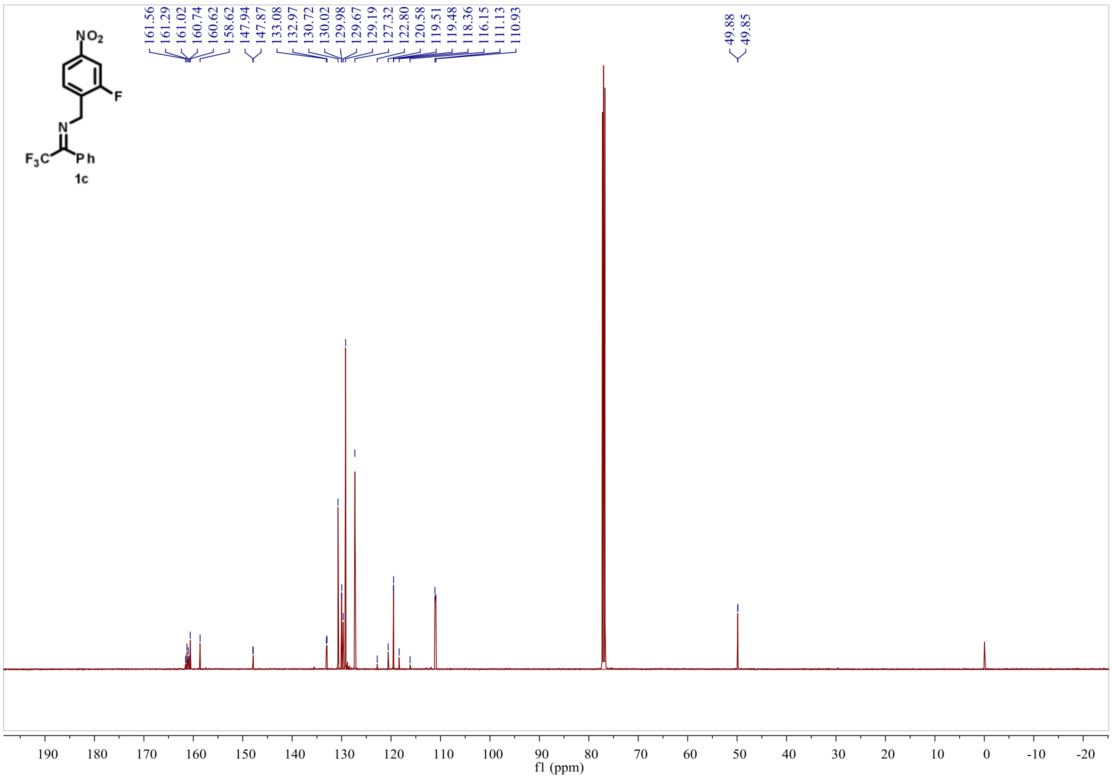

Supplementary Figure 41. ^{13}C NMR spectrum for compound **1c**

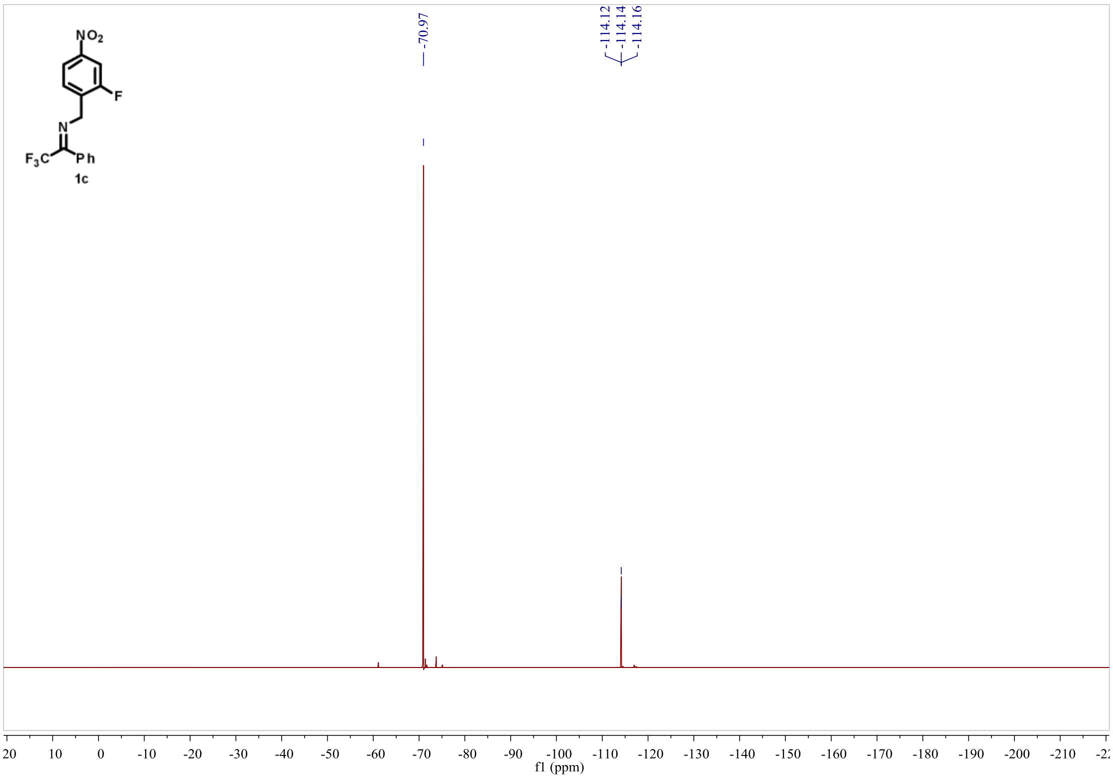

Supplementary Figure 42. ^{19}F NMR spectrum for compound **1c**

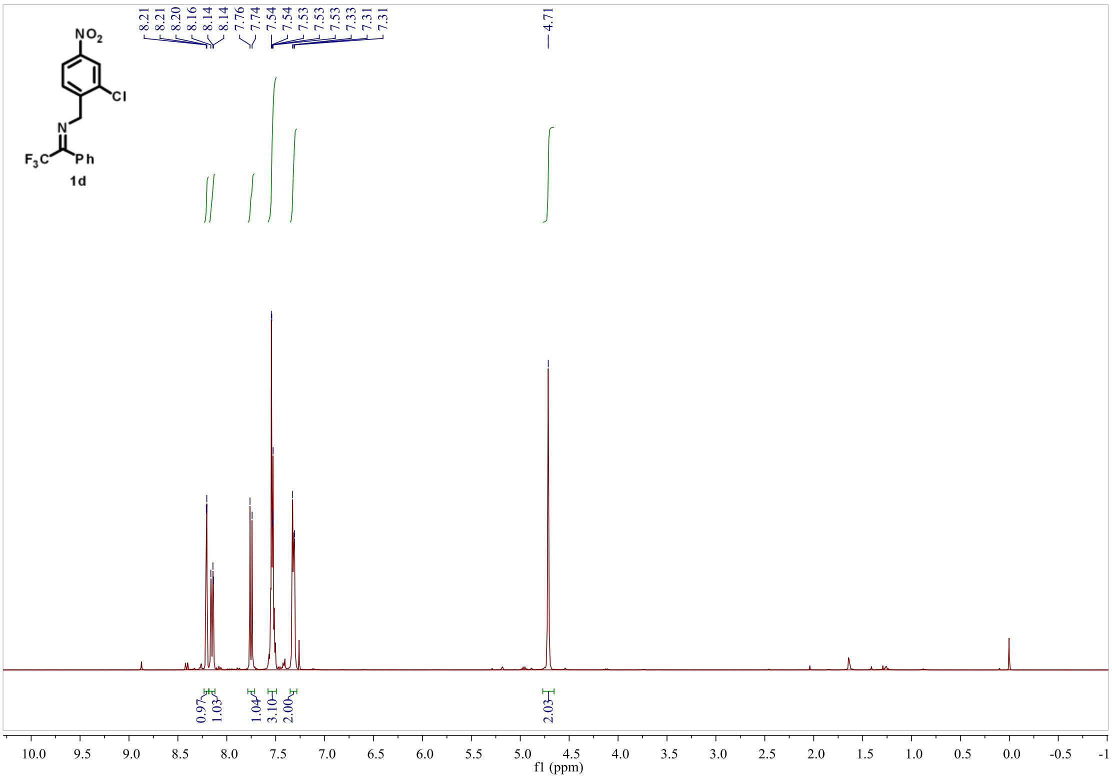

Supplementary Figure 43. ^{1}H NMR spectrum for compound **1d**

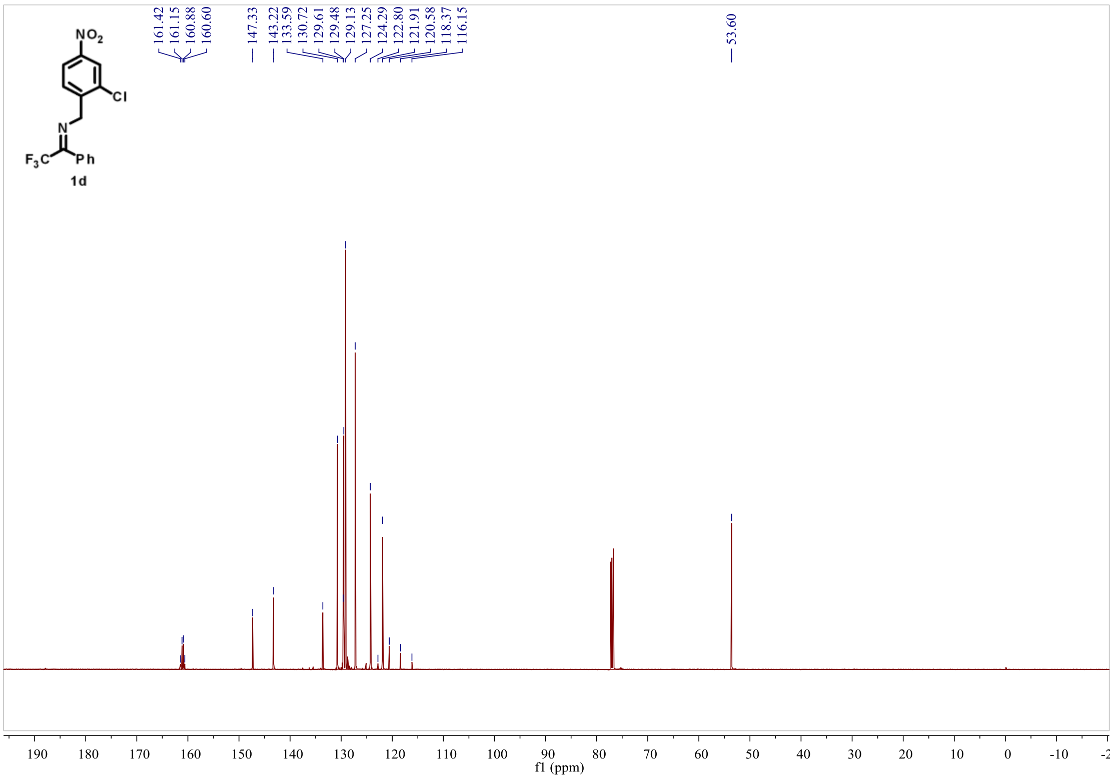

Supplementary Figure 44. ^{13}C NMR spectrum for compound **1d**

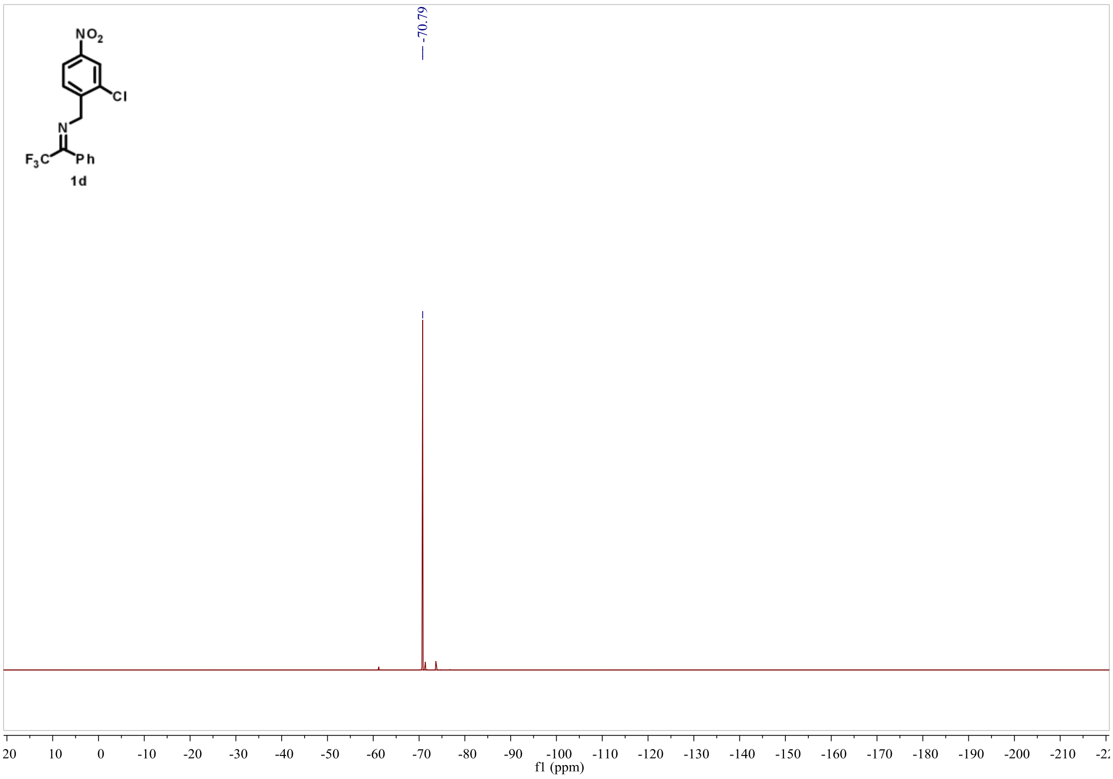

Supplementary Figure 45. ^{19}F NMR spectrum for compound **1d**

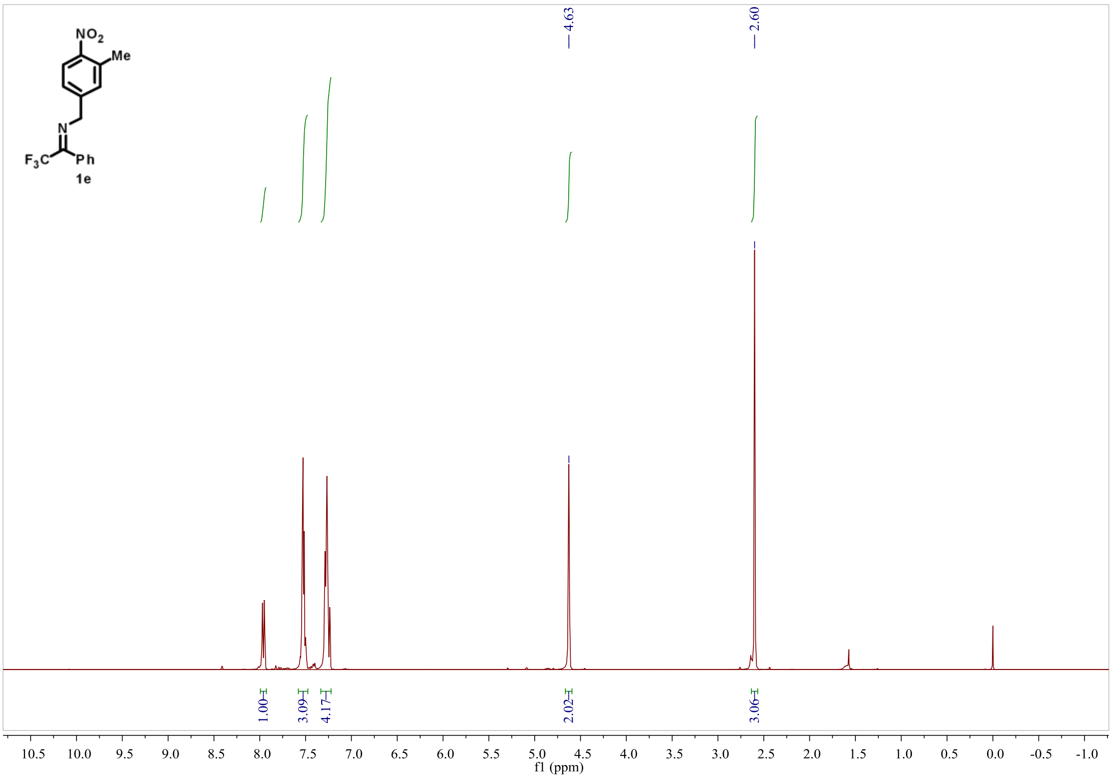

Supplementary Figure 46. ^{1}H NMR spectrum for compound **1e**

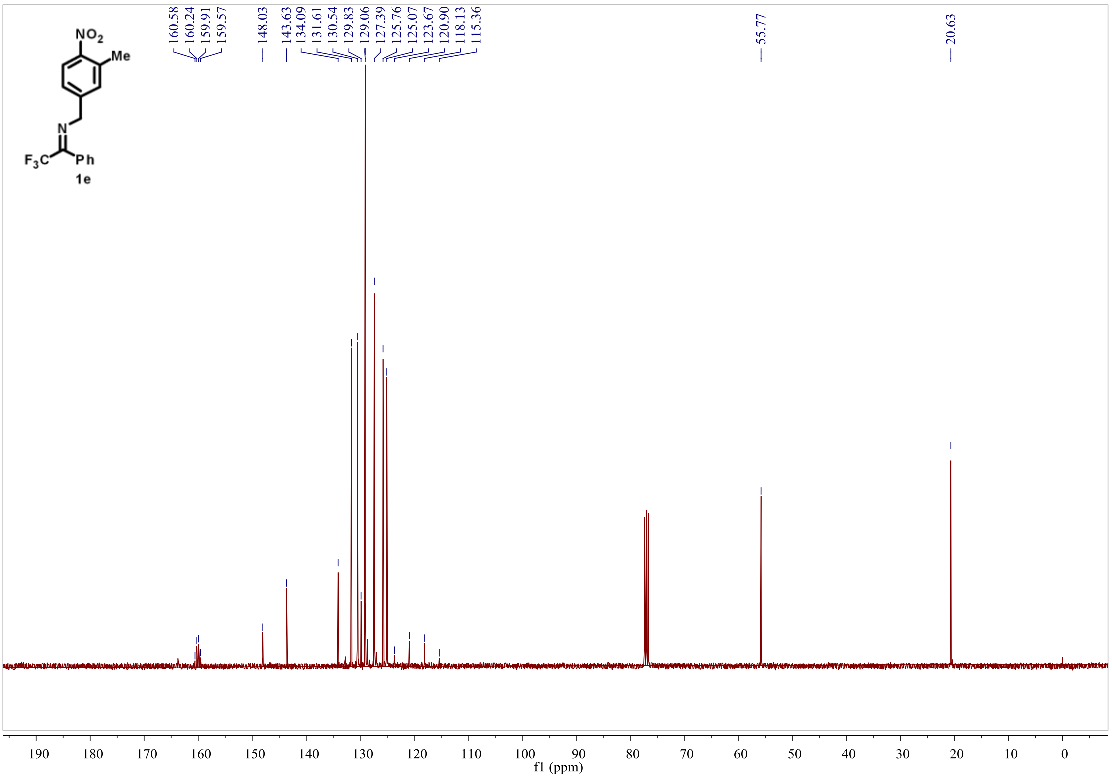

Supplementary Figure 47. ^{13}C NMR spectrum for compound **1e**

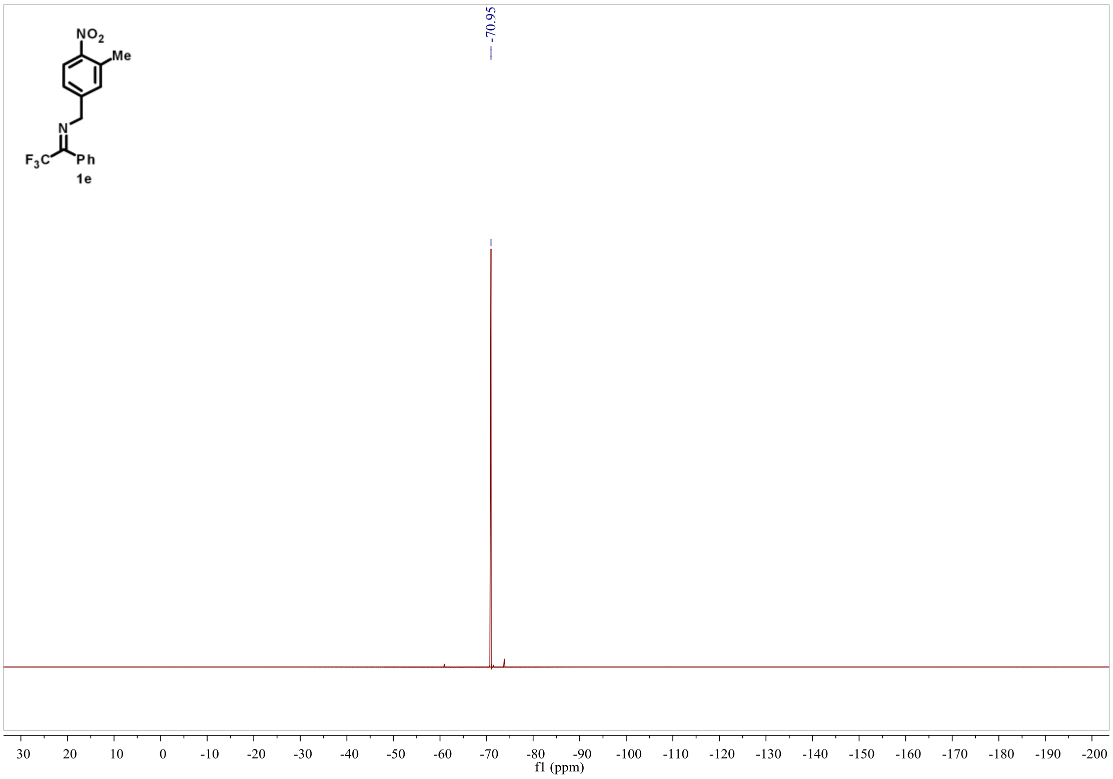

Supplementary Figure 48. ^{19}F NMR spectrum for compound **1e**

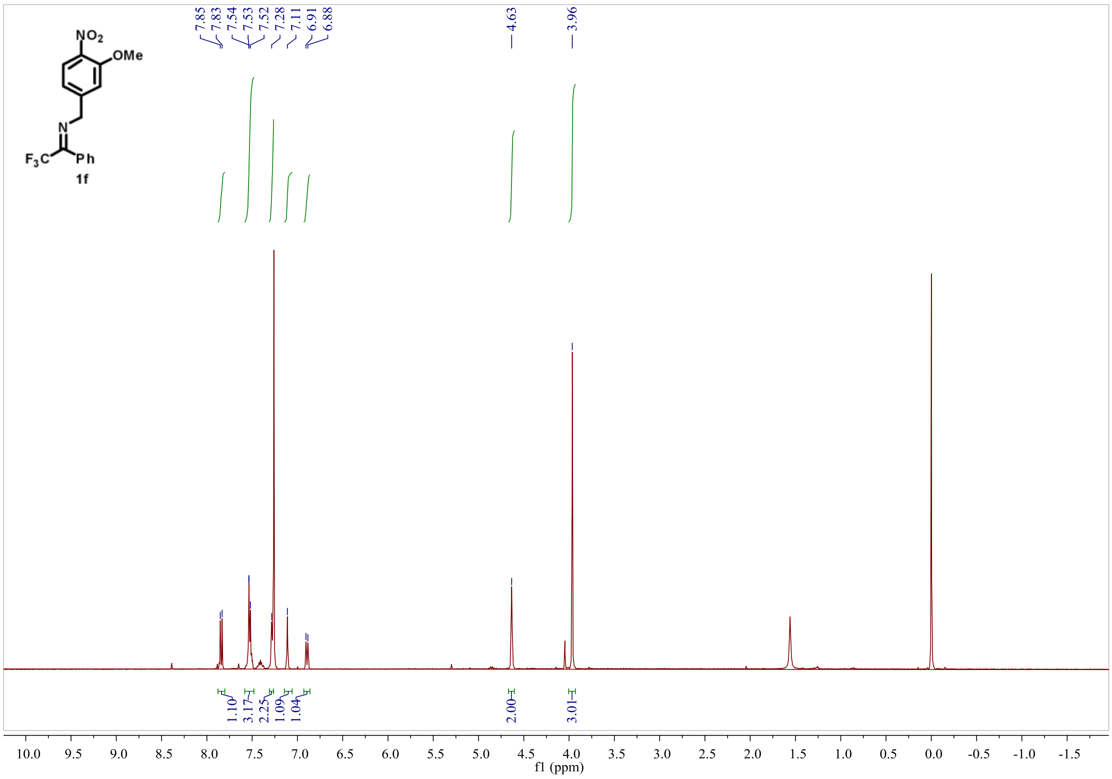

Supplementary Figure 49. ^{1}H NMR spectrum for compound **1f**

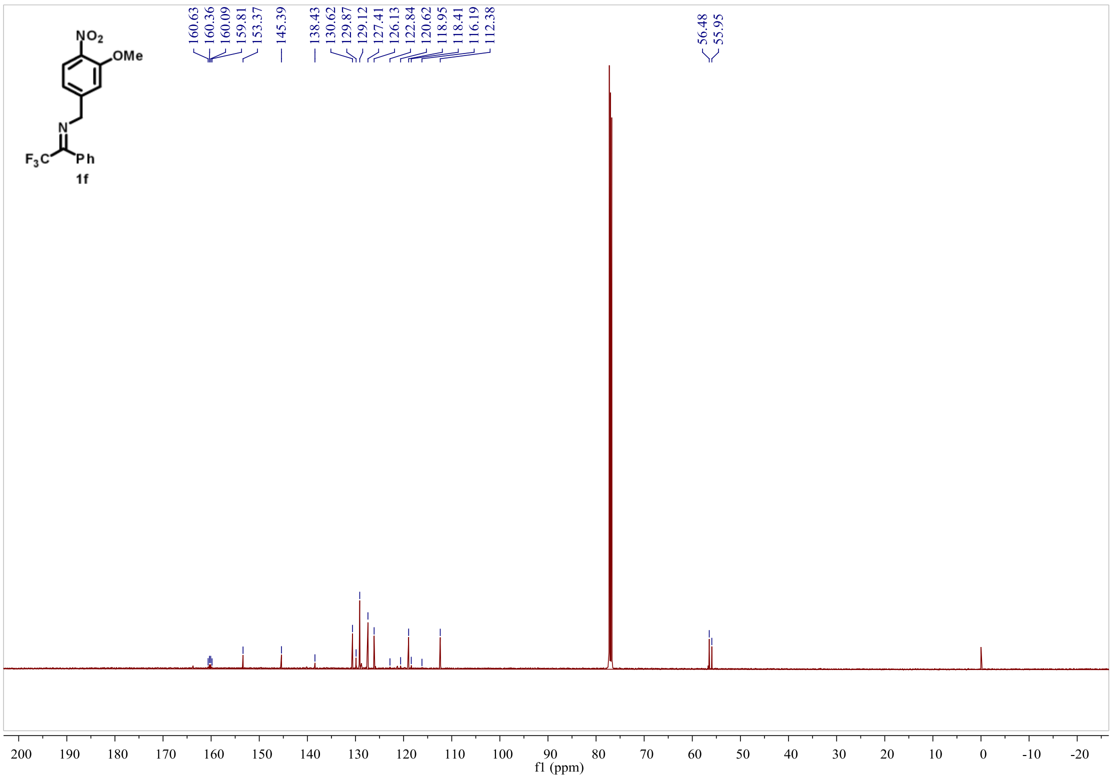

Supplementary Figure 50. ^{13}C NMR spectrum for compound **1f**

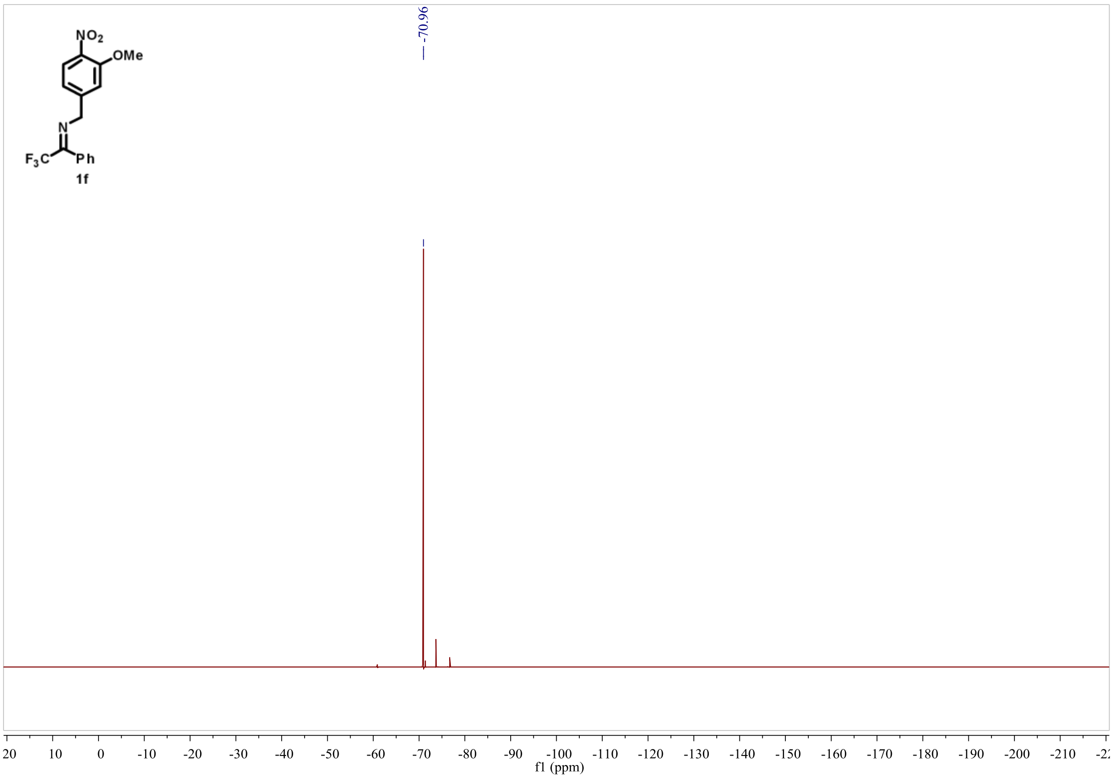

Supplementary Figure 51. ^{19}F NMR spectrum for compound **1f**

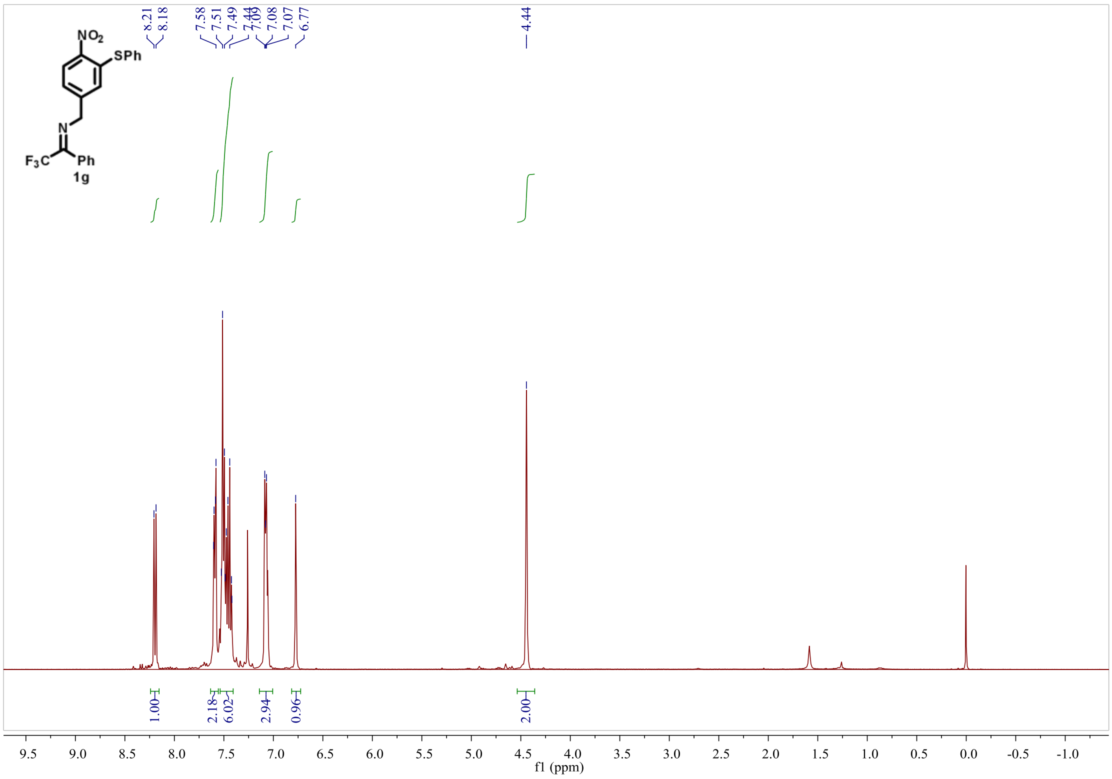

Supplementary Figure 52. ^{1}H NMR spectrum for compound **1g**

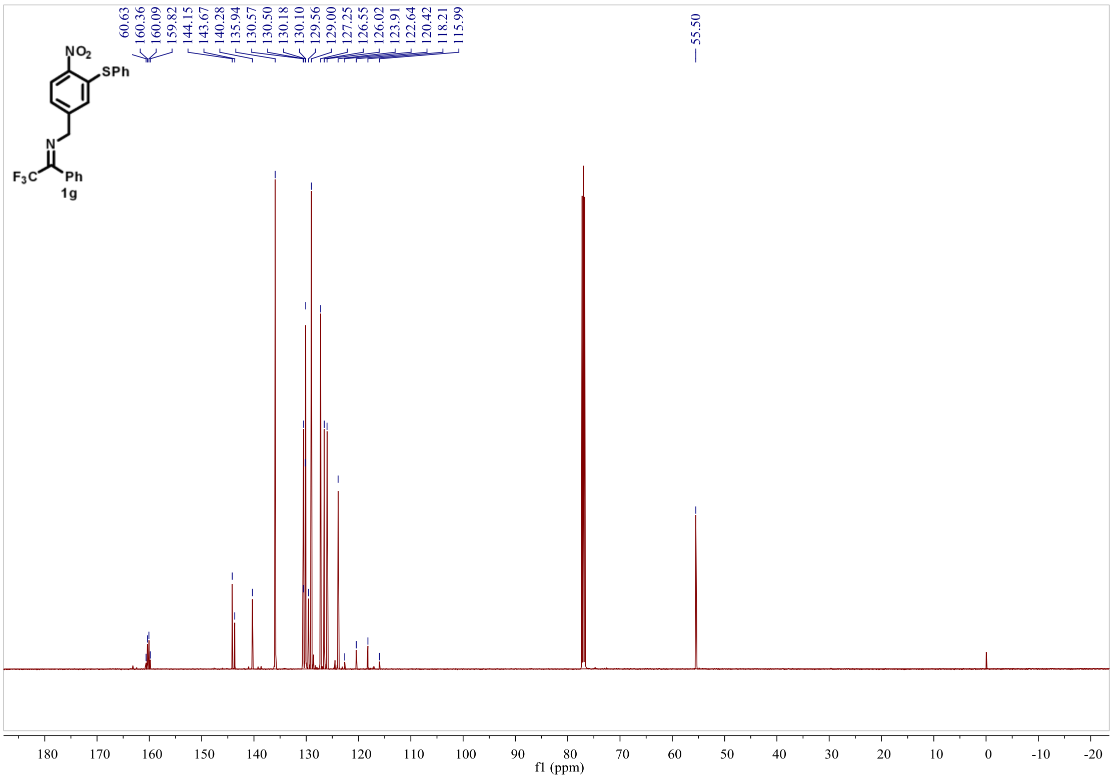

Supplementary Figure 53. ^{13}C NMR spectrum for compound **1g**

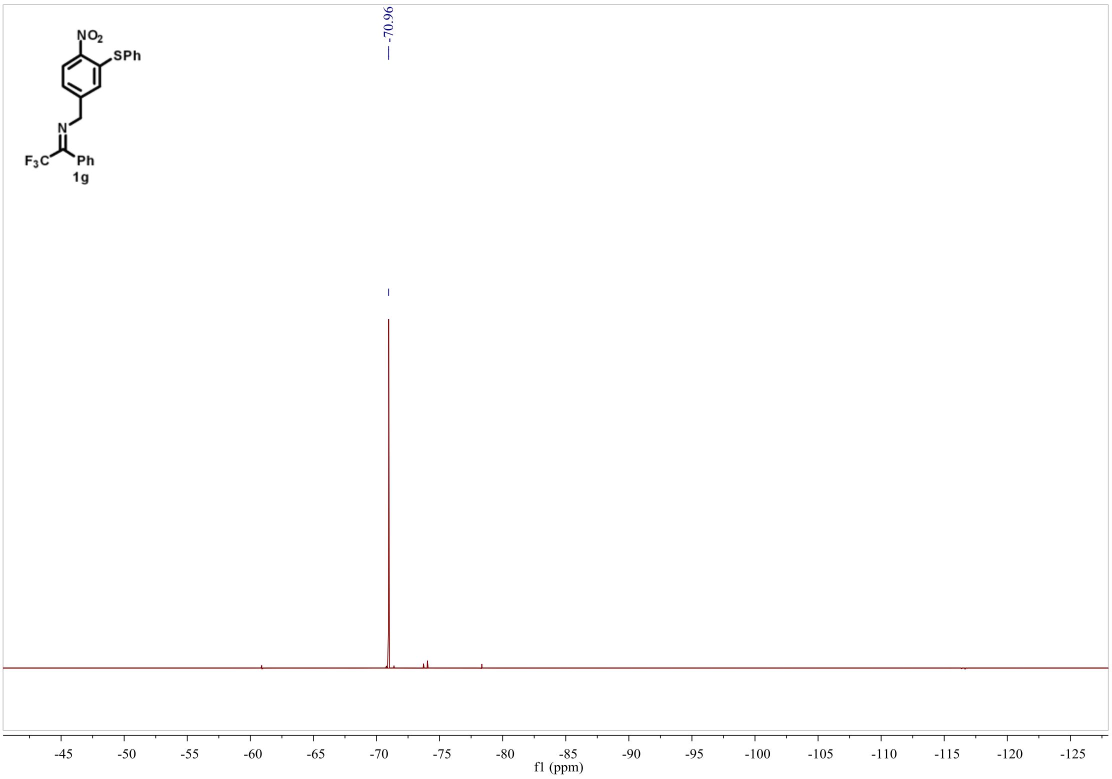

Supplementary Figure 54. ^{19}F NMR spectrum for compound **1g**

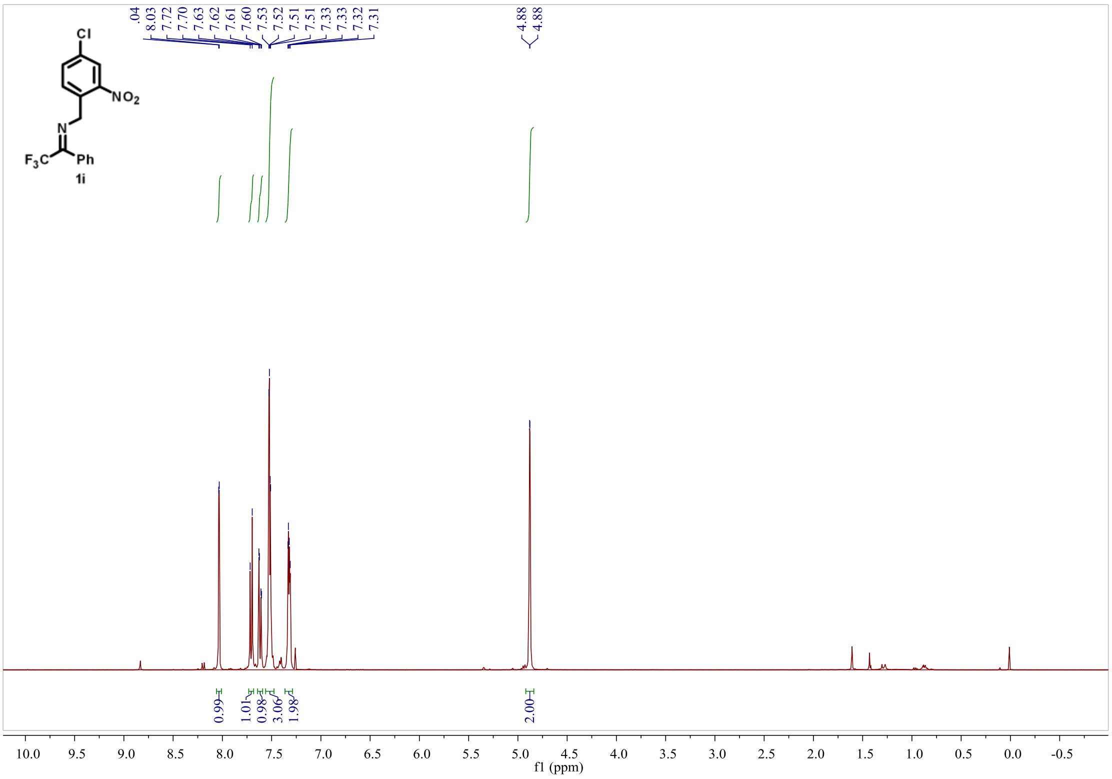

Supplementary Figure 55. ^{1}H NMR spectrum for compound **1i**

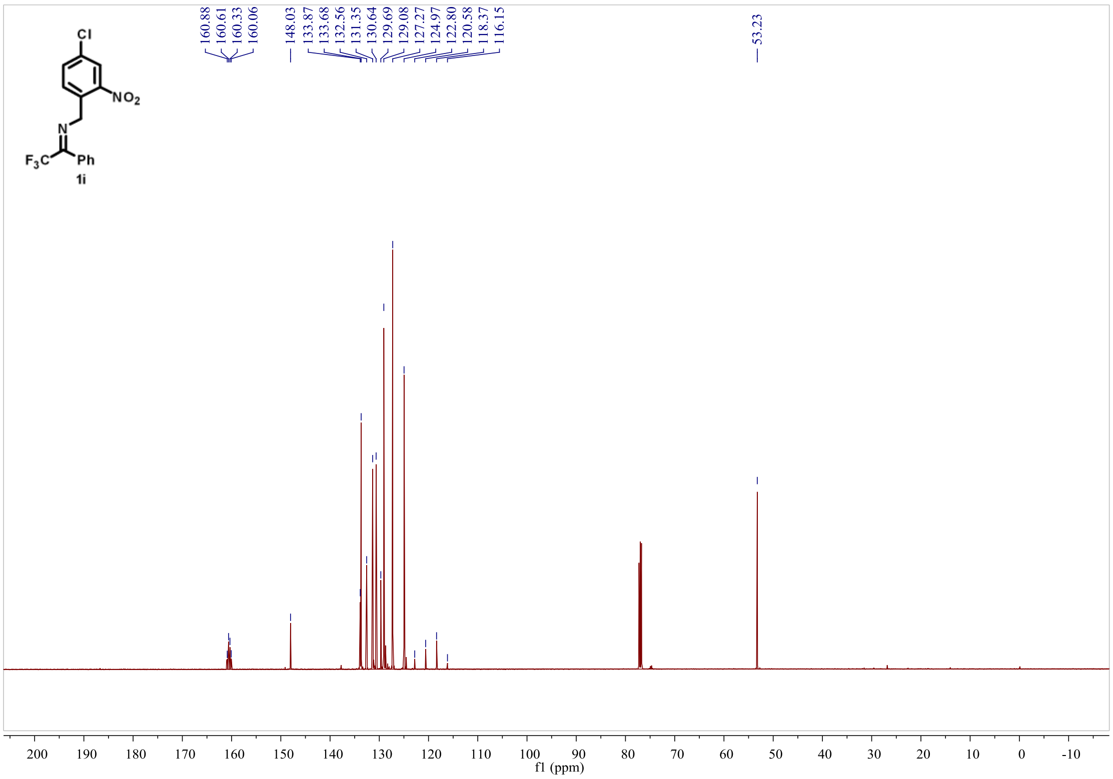

Supplementary Figure 56. ^{13}C NMR spectrum for compound **1i**

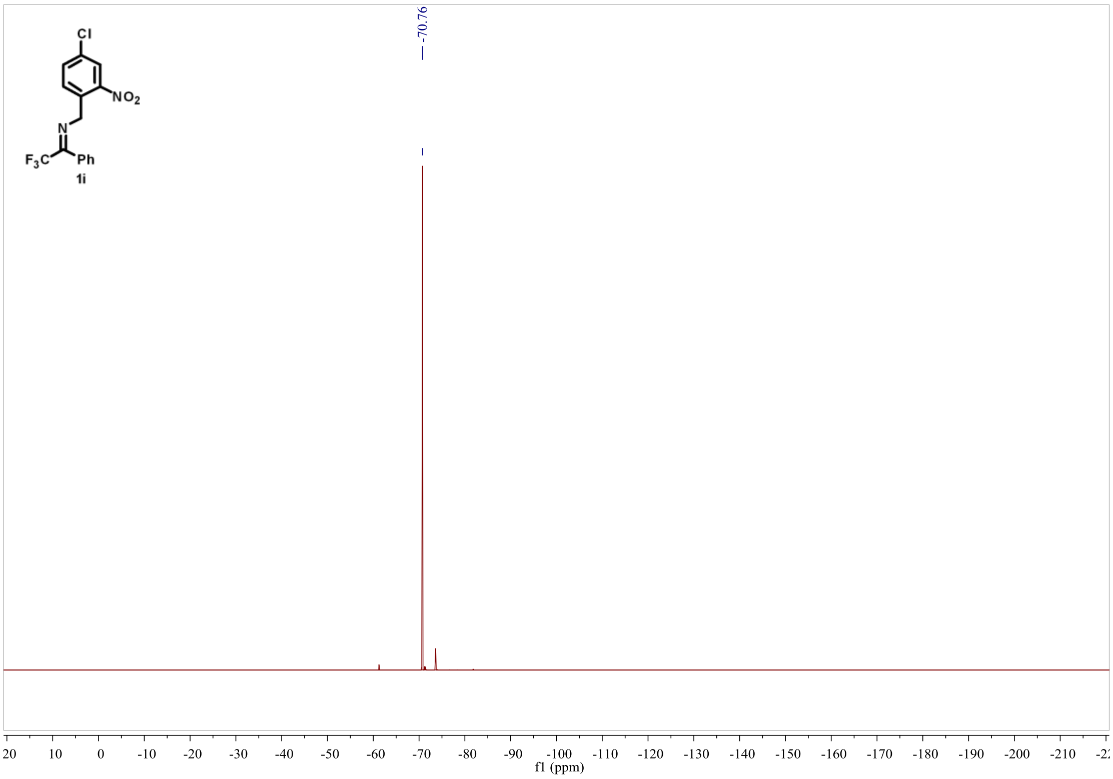

Supplementary Figure 57. ^{19}F NMR spectrum for compound **1i**

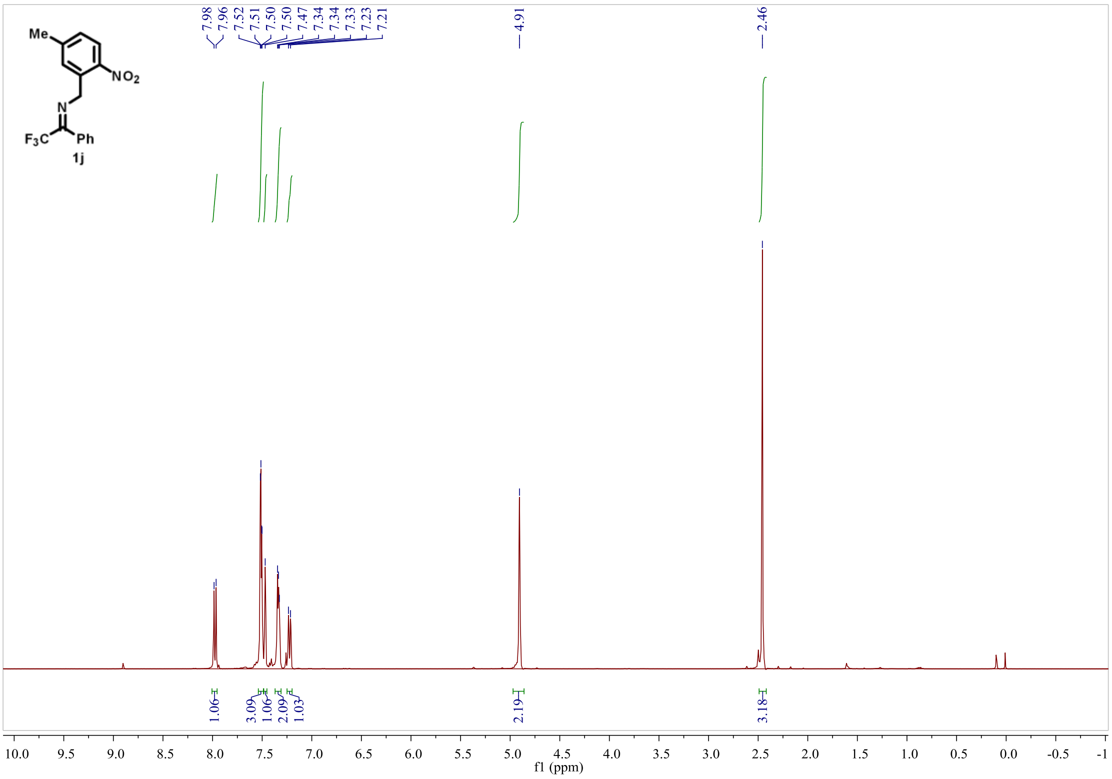

Supplementary Figure 58. ^{1}H NMR spectrum for compound **1j**

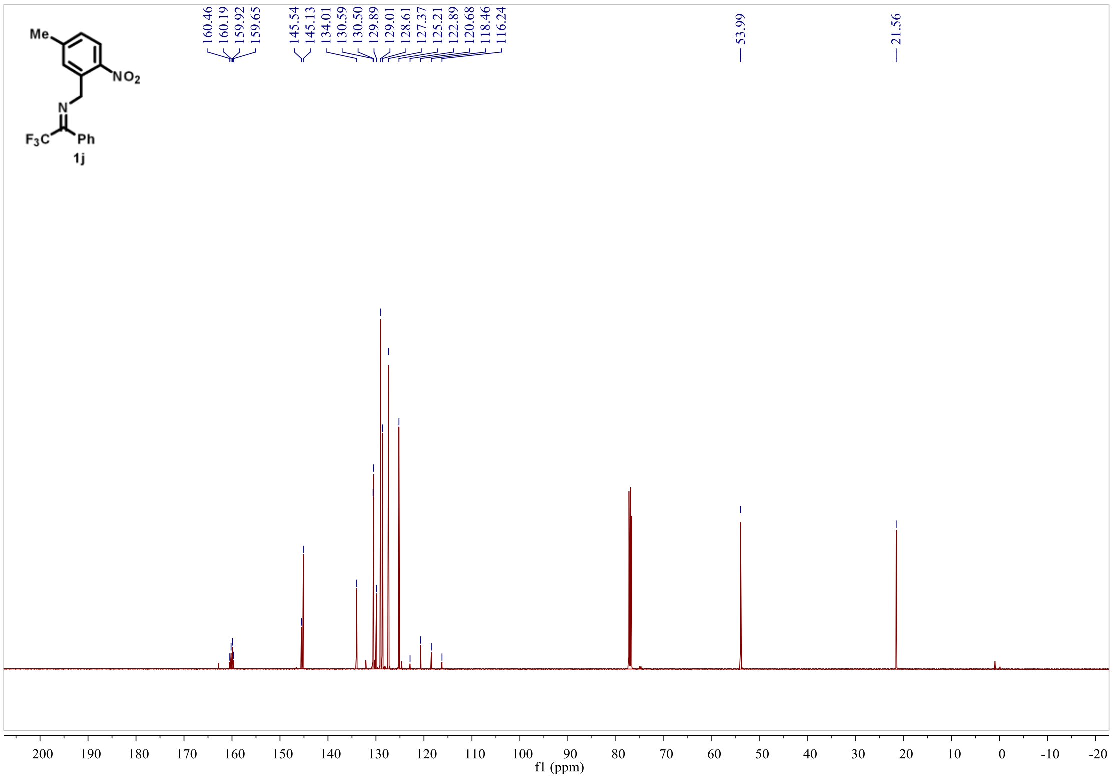

Supplementary Figure 59. ^{13}C NMR spectrum for compound **1j**

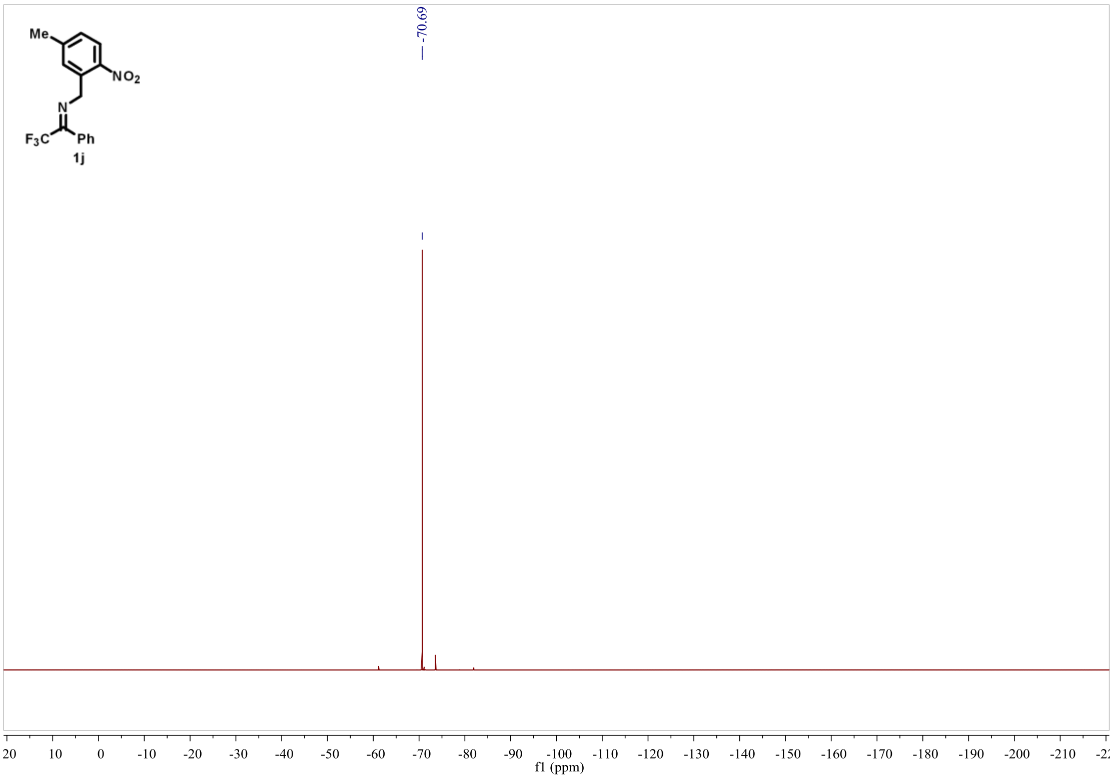

Supplementary Figure 60. ^{19}F NMR spectrum for compound **1j**

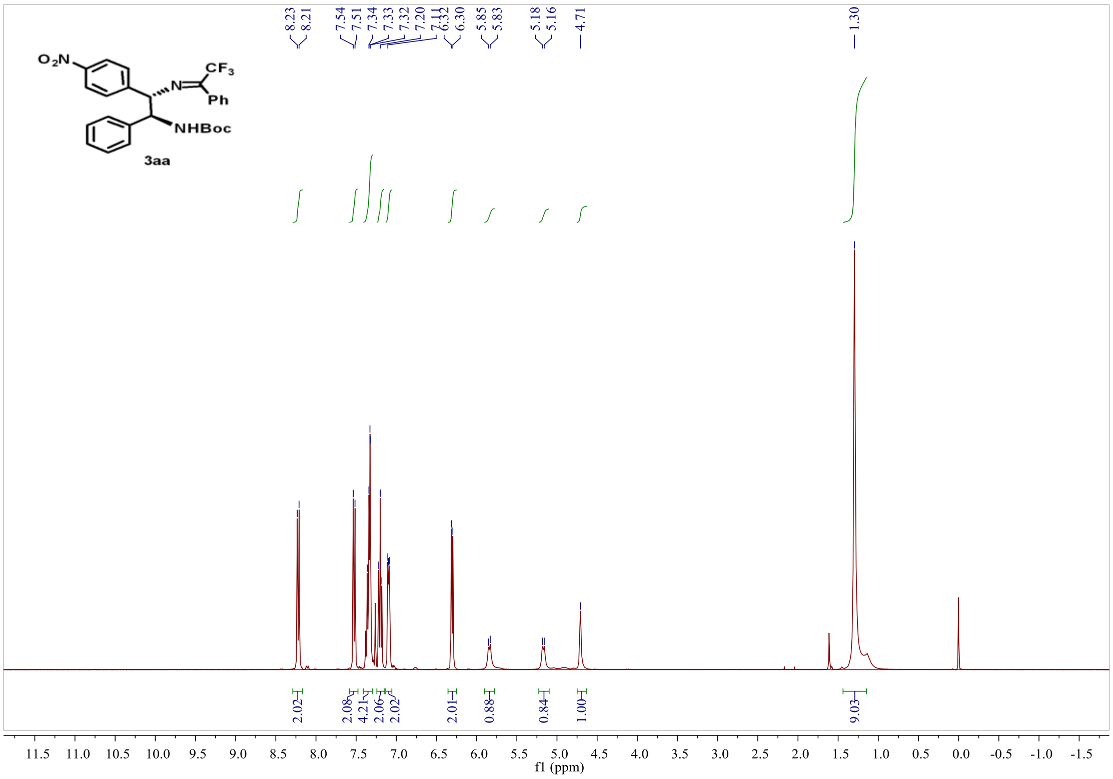

Supplementary Figure 61. ^{1}H NMR spectrum for compound **3aa**

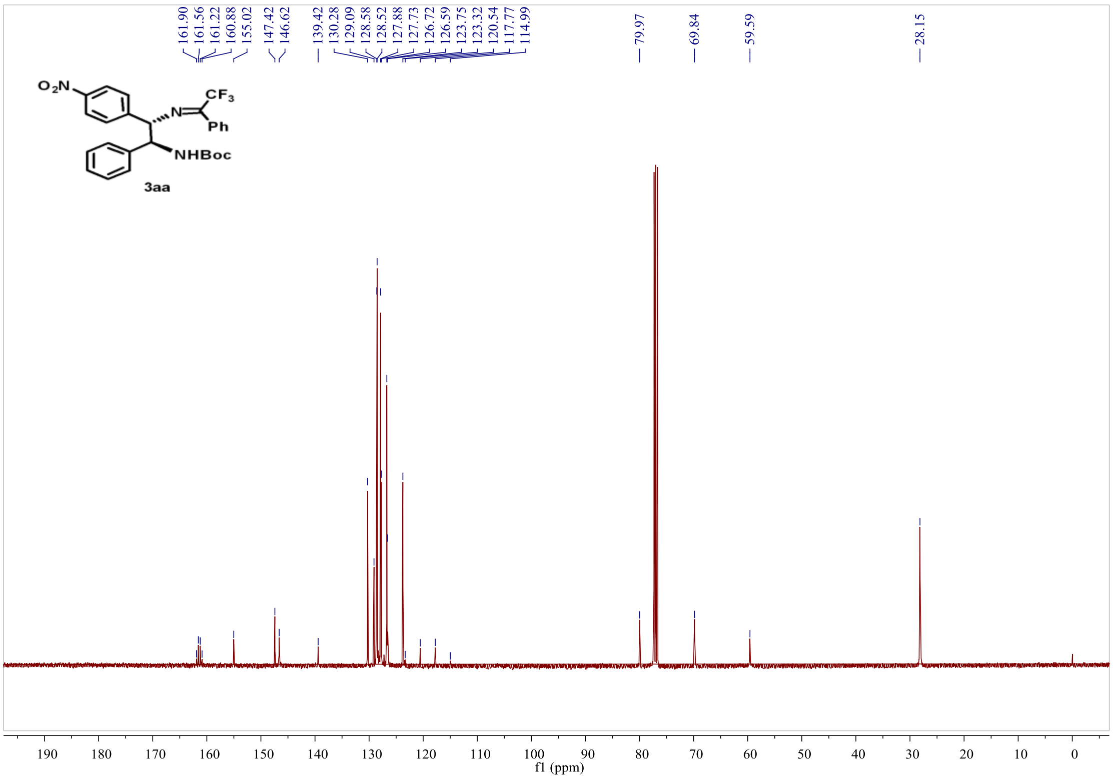

Supplementary Figure 62. ^{13}C NMR spectrum for compound **3aa**

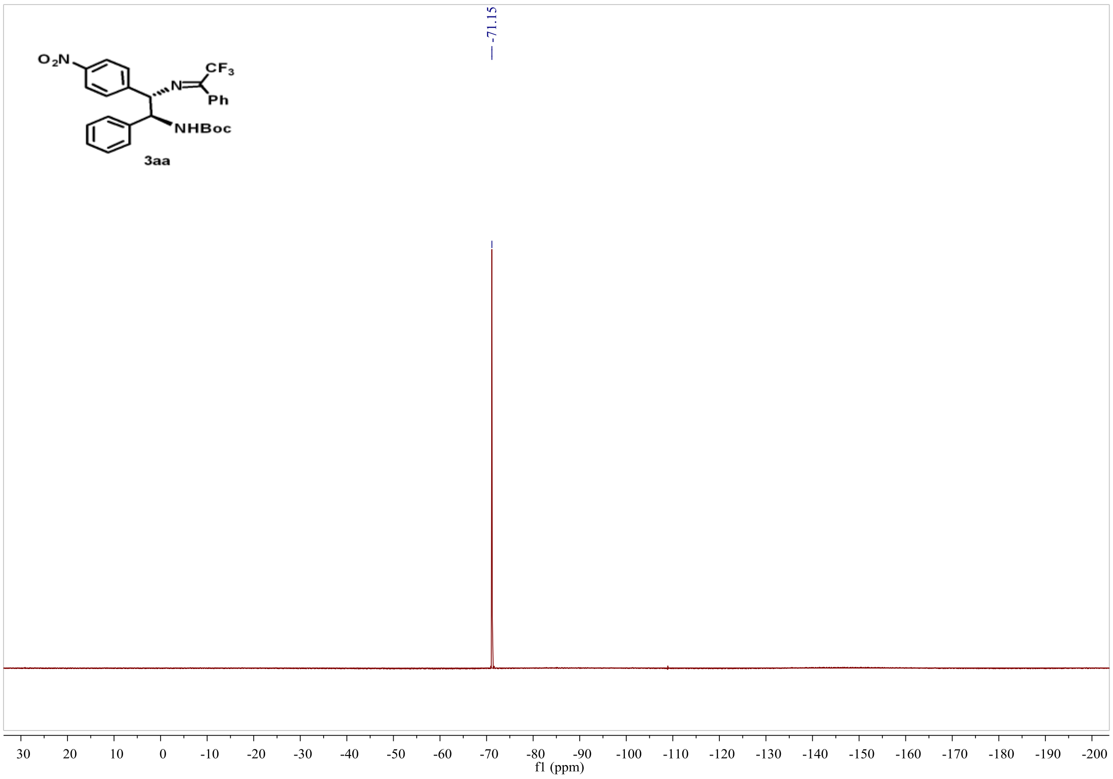

Supplementary Figure 63. ^{19}F NMR spectrum for compound **3aa**

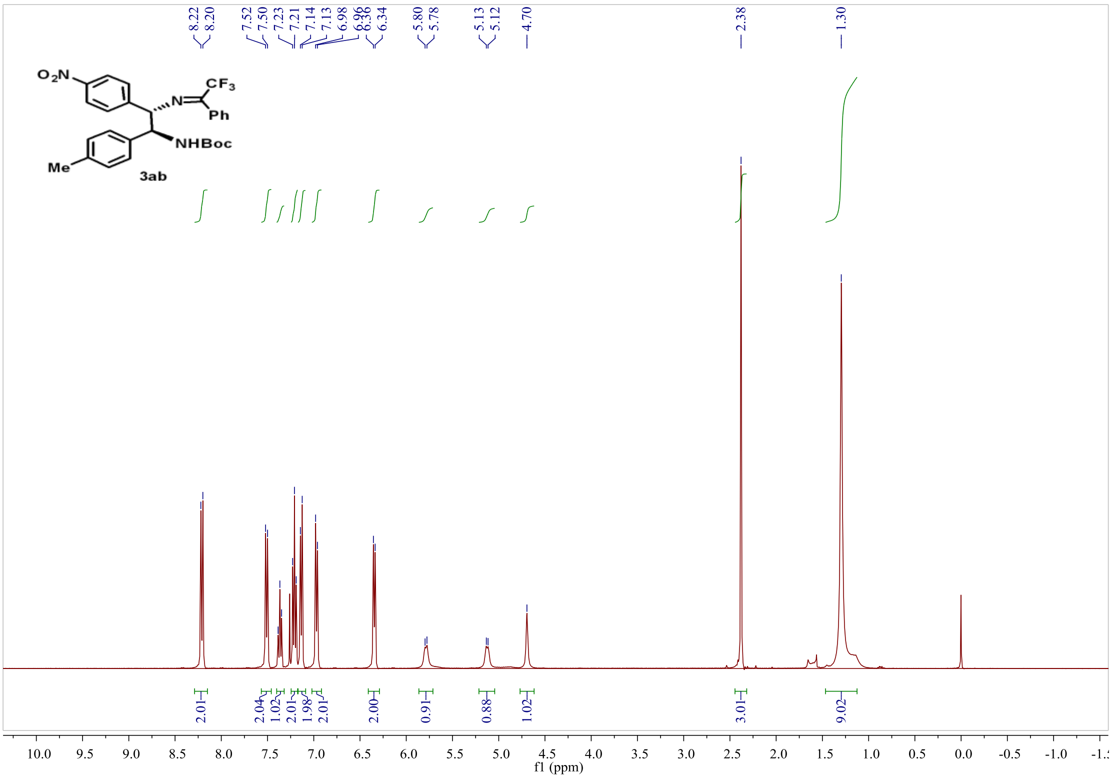

Supplementary Figure 64. ^{1}H NMR spectrum for compound **3ab**

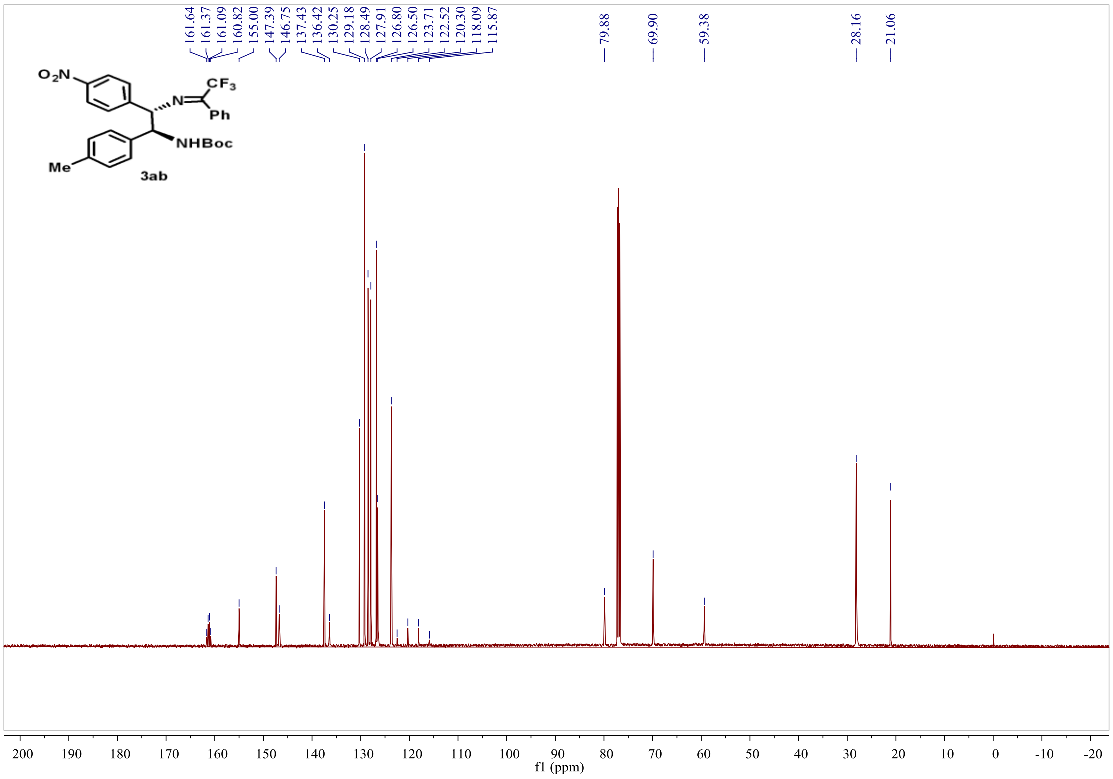

Supplementary Figure 65. ^{13}C NMR spectrum for compound **3ab**

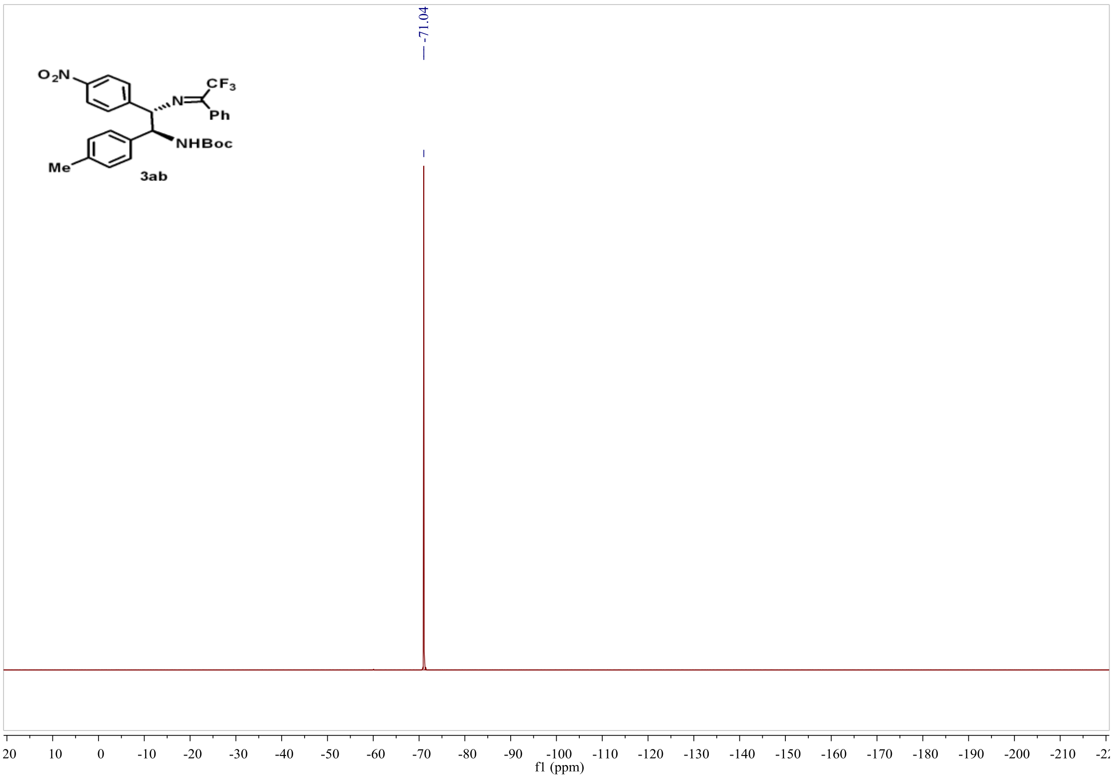

Supplementary Figure 66. ^{19}F NMR spectrum for compound **3ab**

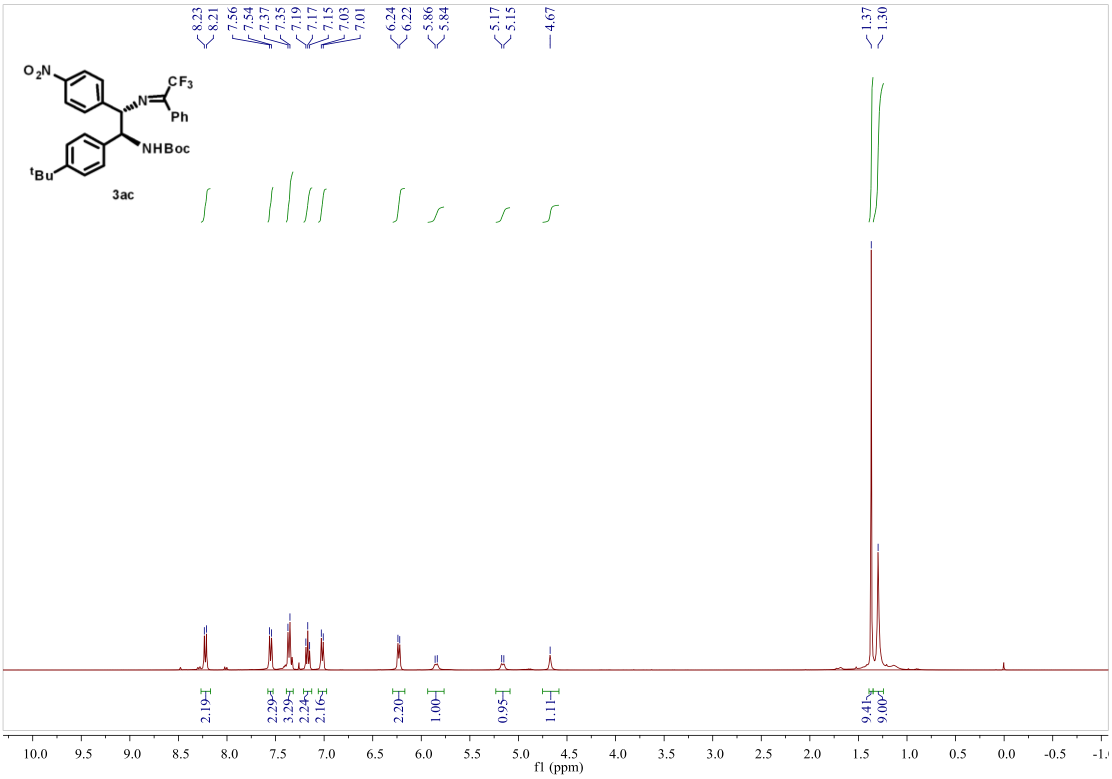

Supplementary Figure 67. ^{1}H NMR spectrum for compound **3ac**

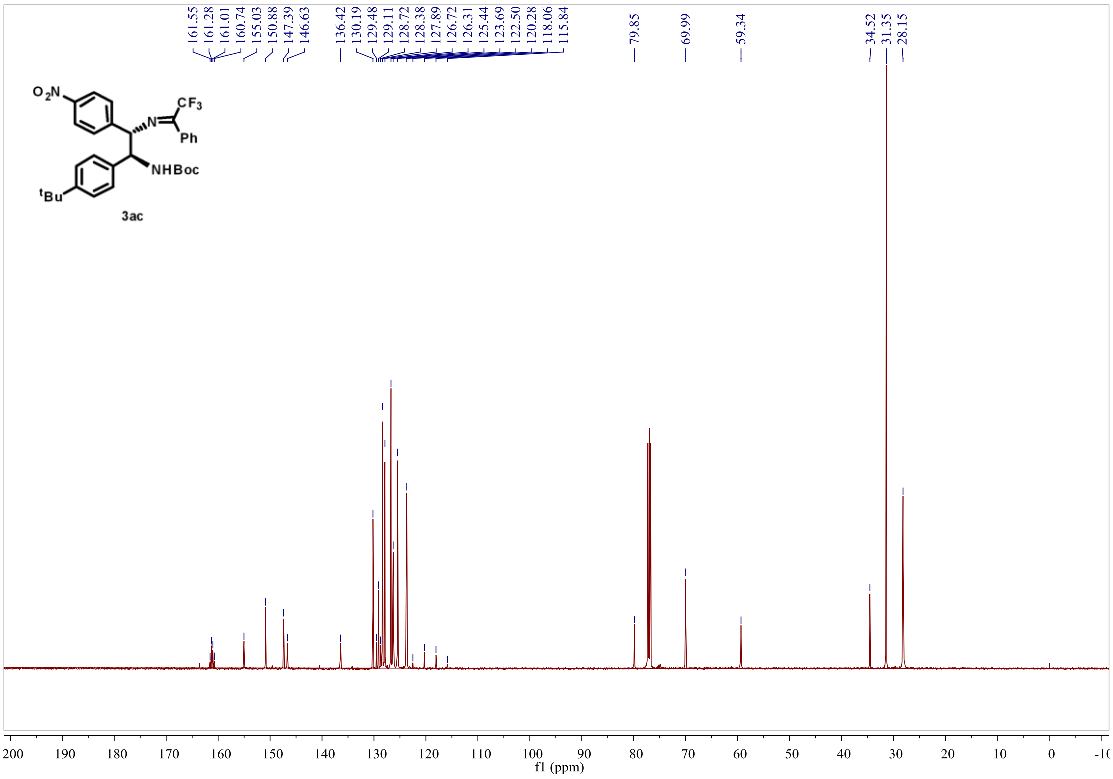

Supplementary Figure 68. ^{13}C NMR spectrum for compound **3ac**

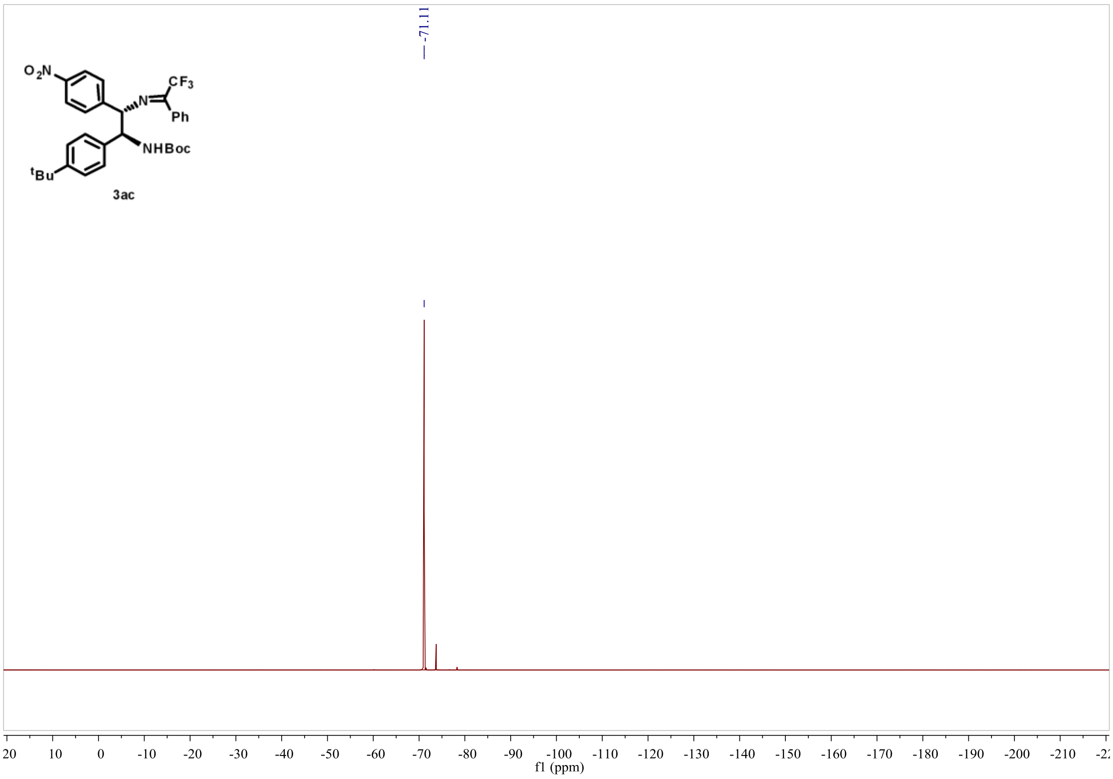

Supplementary Figure 69. ^{19}F NMR spectrum for compound **3ac**

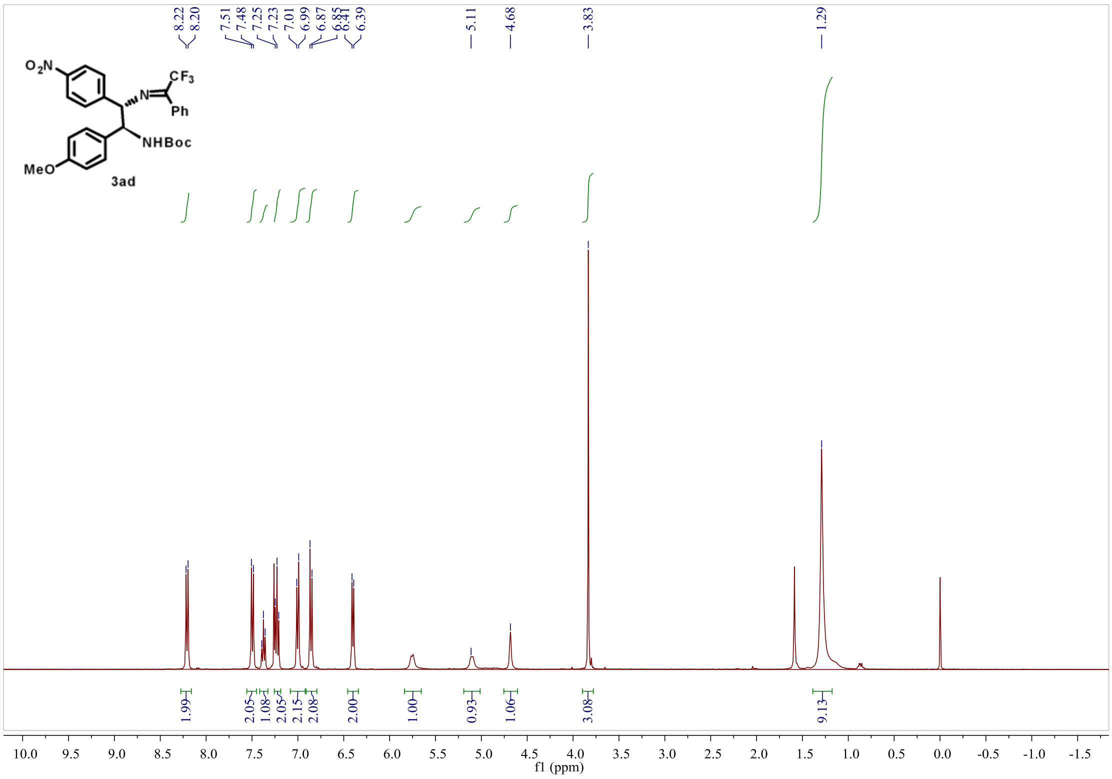

Supplementary Figure 70. ^{1}H NMR spectrum for compound **3ad**

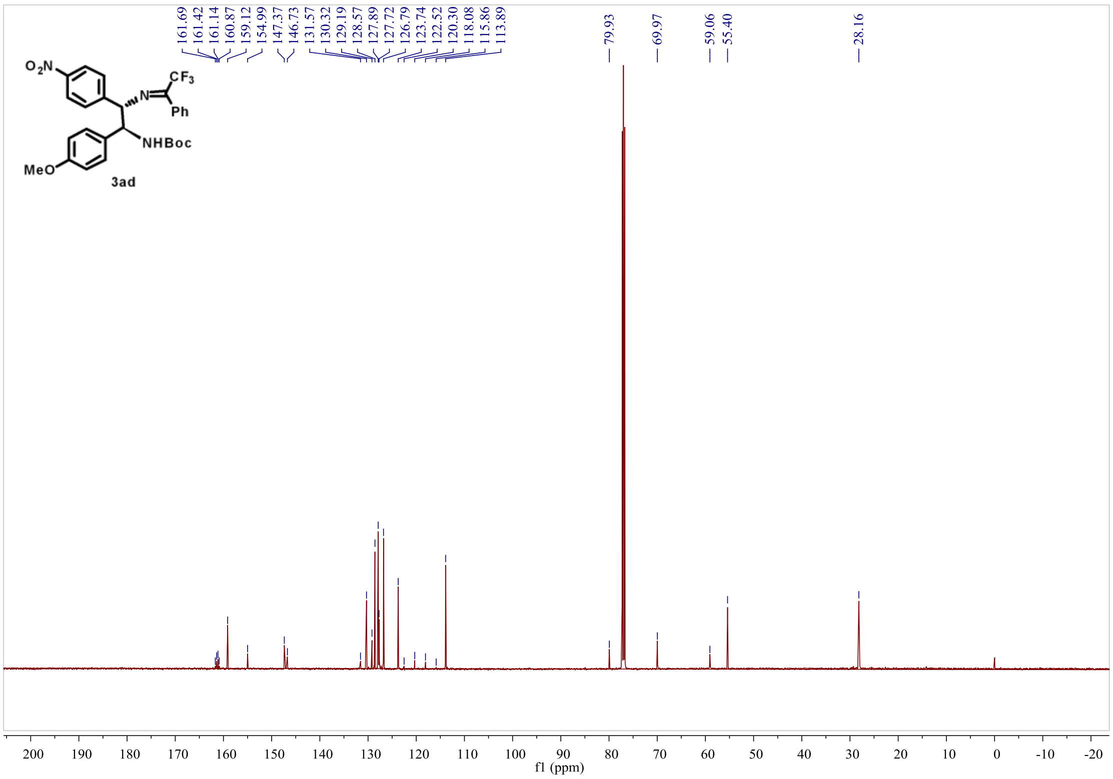

Supplementary Figure 71. ^{13}C NMR spectrum for compound **3ad**

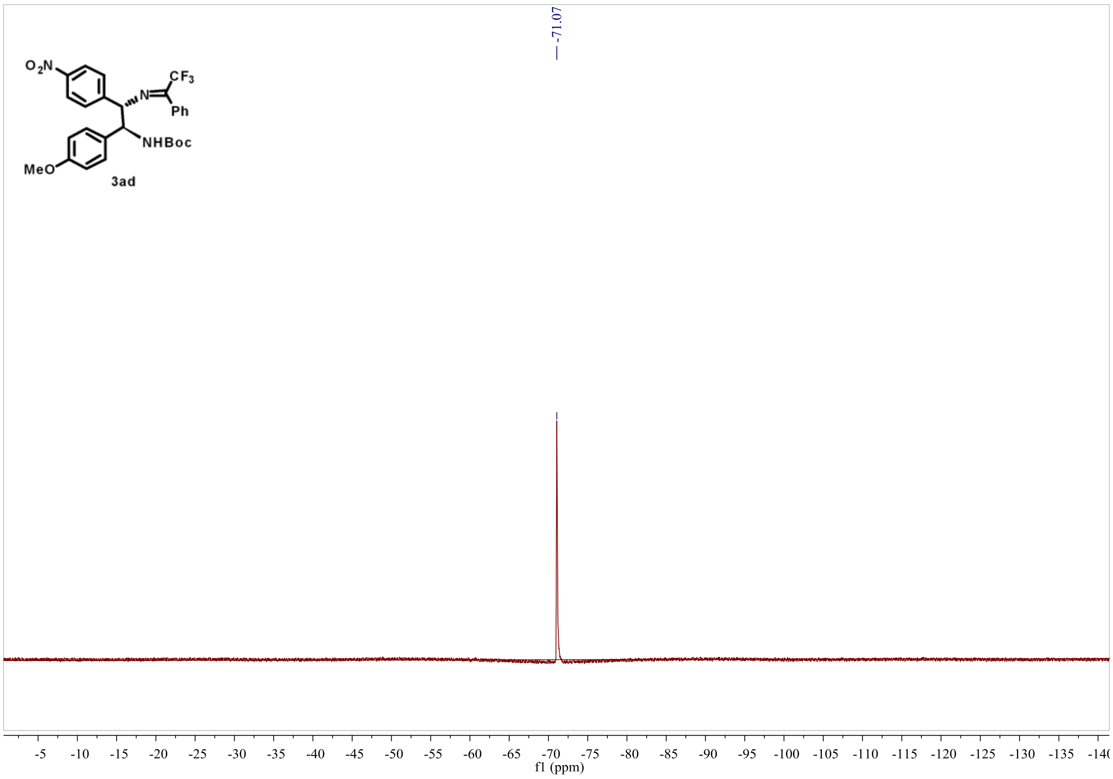

Supplementary Figure 72. ^{19}F NMR spectrum for compound **3ad**

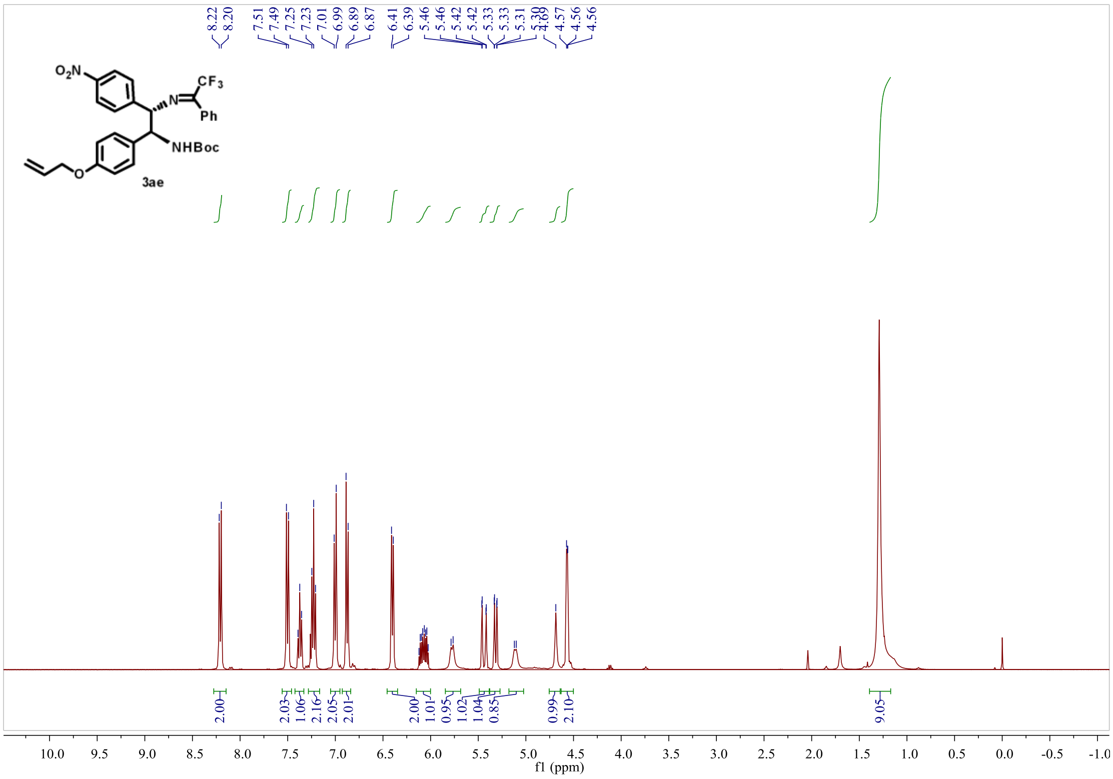

Supplementary Figure 73. ^{1}H NMR spectrum for compound **3ae**

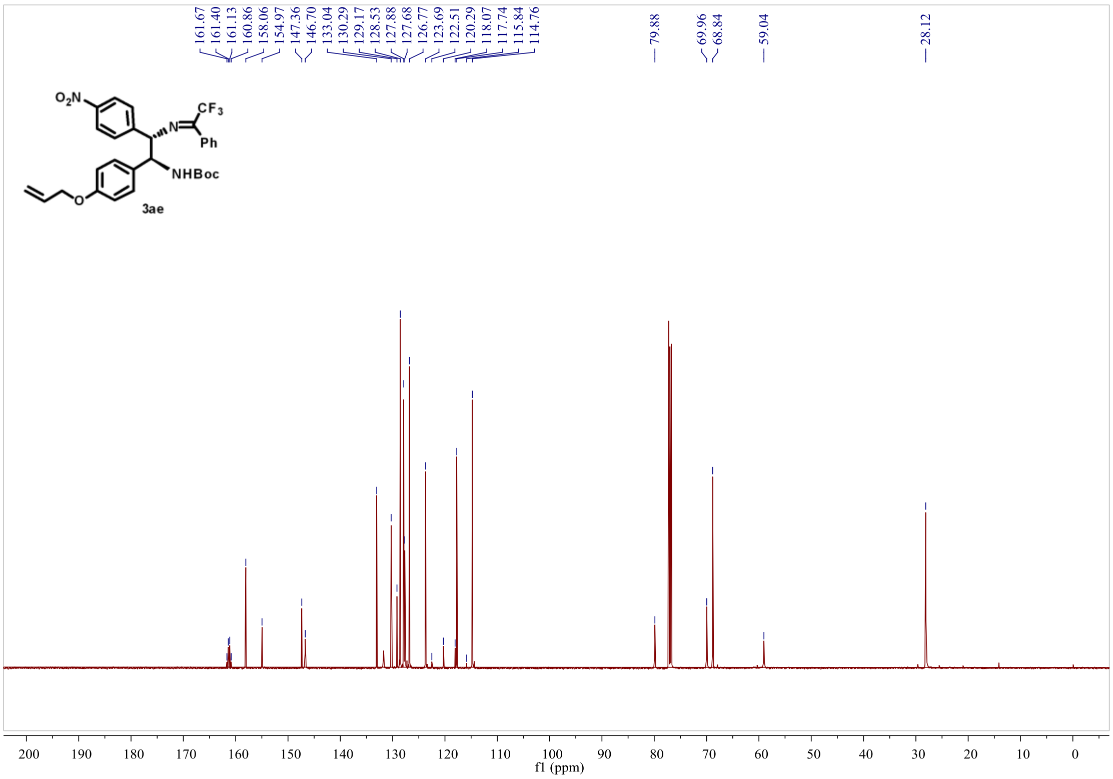

Supplementary Figure 74. ^{13}C NMR spectrum for compound **3ae**

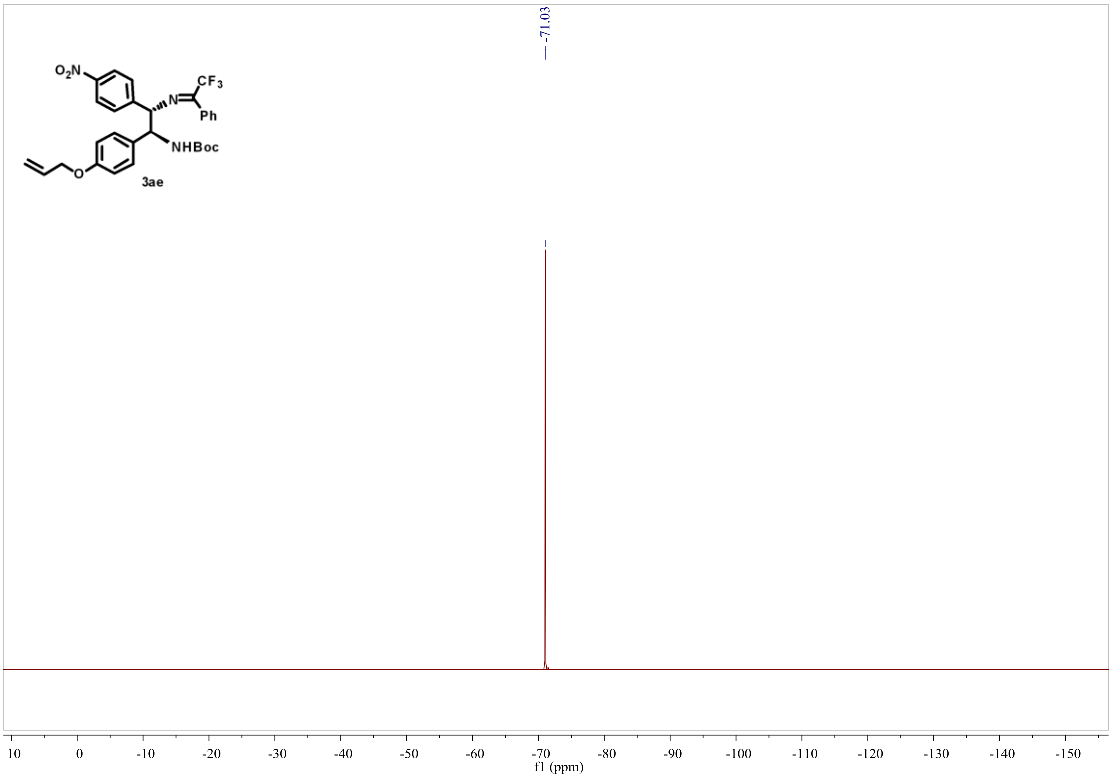

Supplementary Figure 75. ^{19}F NMR spectrum for compound **3ae**

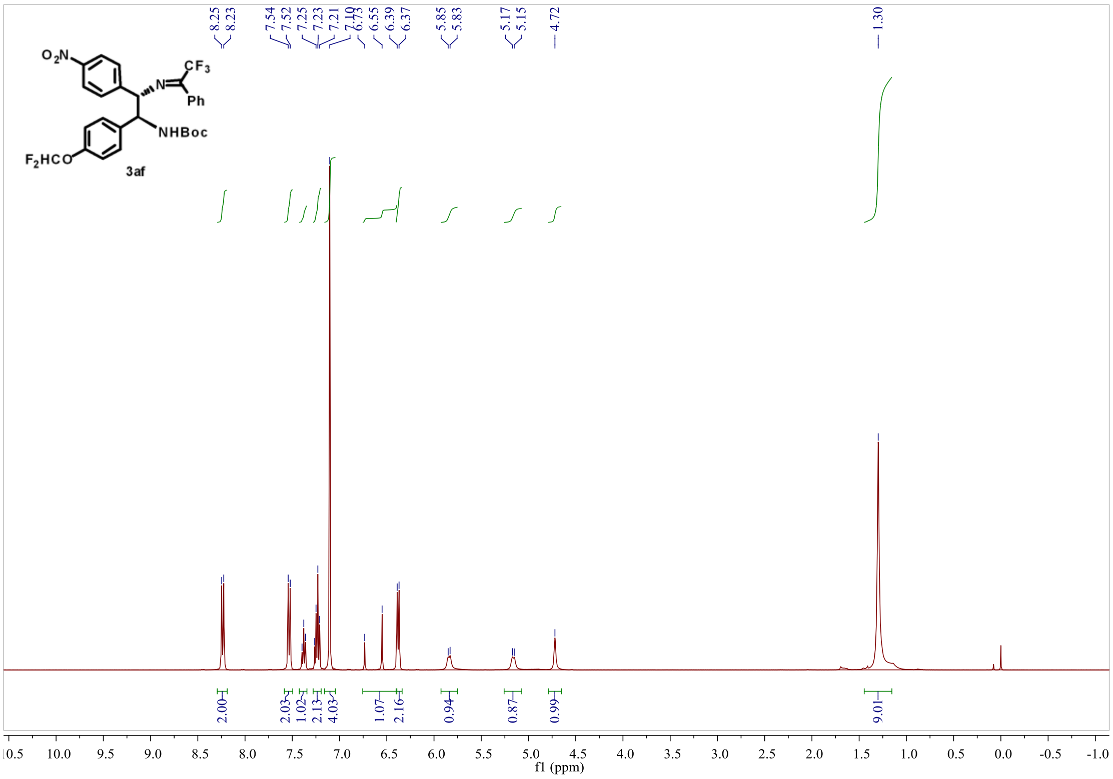

Supplementary Figure 76. ^{1}H NMR spectrum for compound **3af**

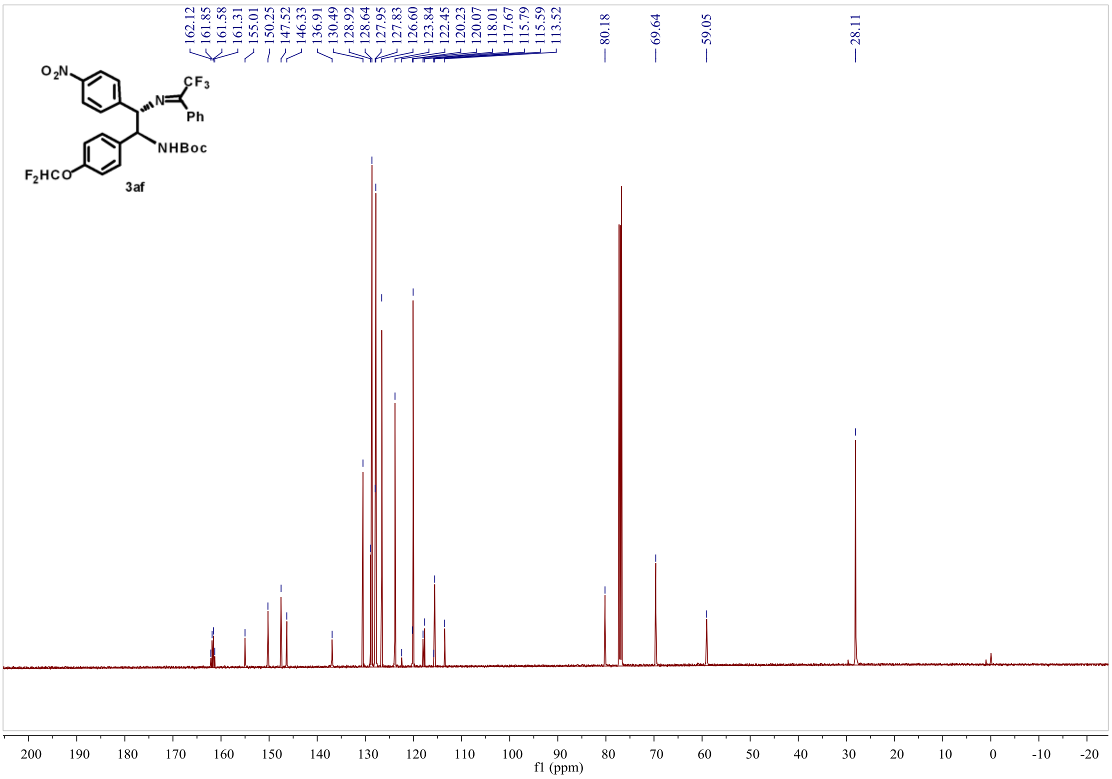

Supplementary Figure 77. ^{13}C NMR spectrum for compound **3af**

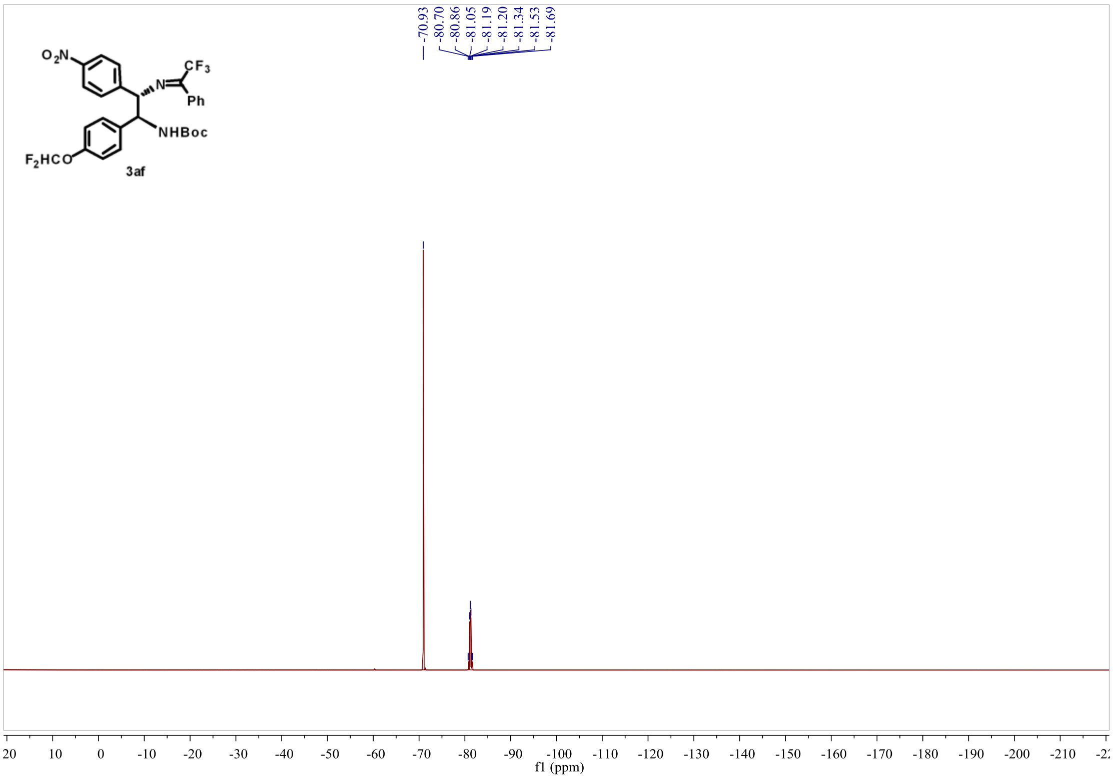

Supplementary Figure 78. ^{19}F NMR spectrum for compound **3af**

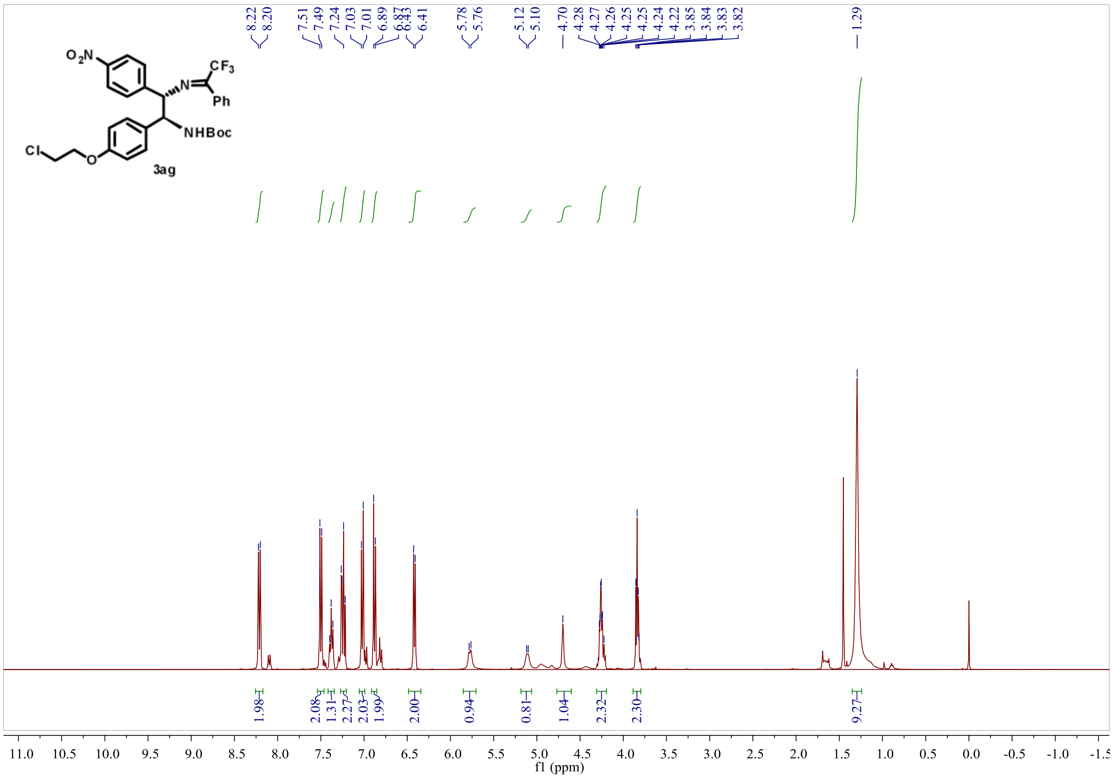

Supplementary Figure 79. ^{1}H NMR spectrum for compound **3ag**

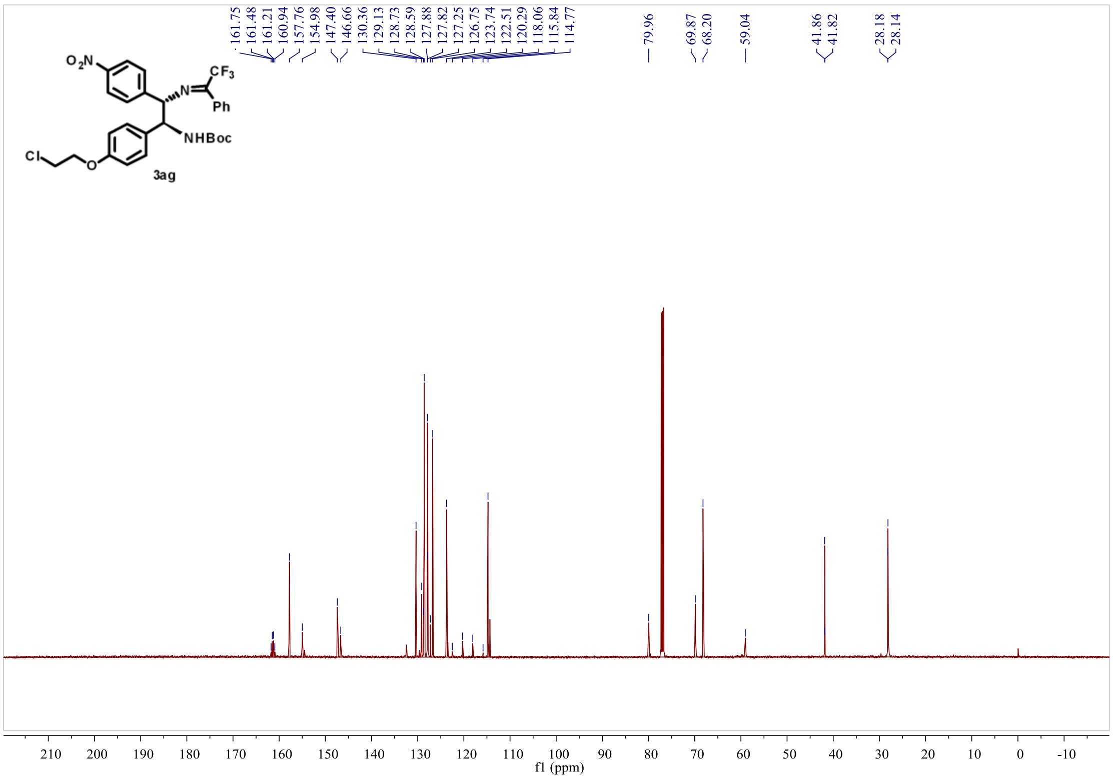

Supplementary Figure 80. ^{13}C NMR spectrum for compound **3ag**

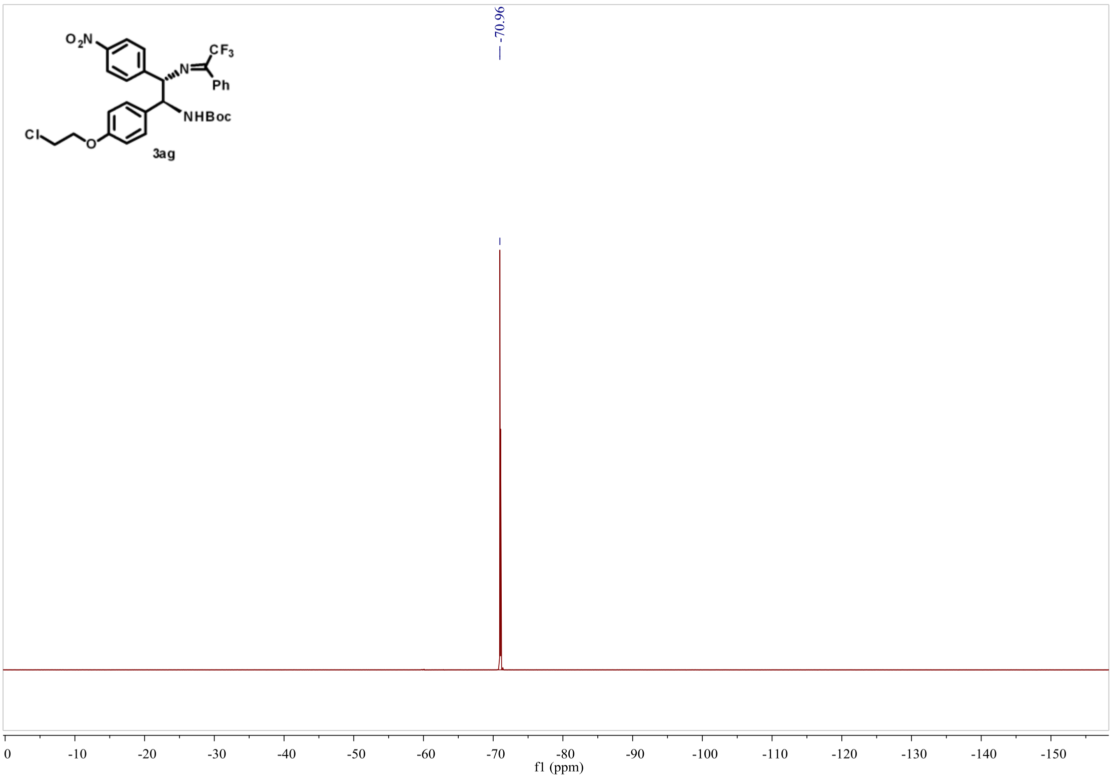

Supplementary Figure 81. ^{19}F NMR spectrum for compound **3ag**

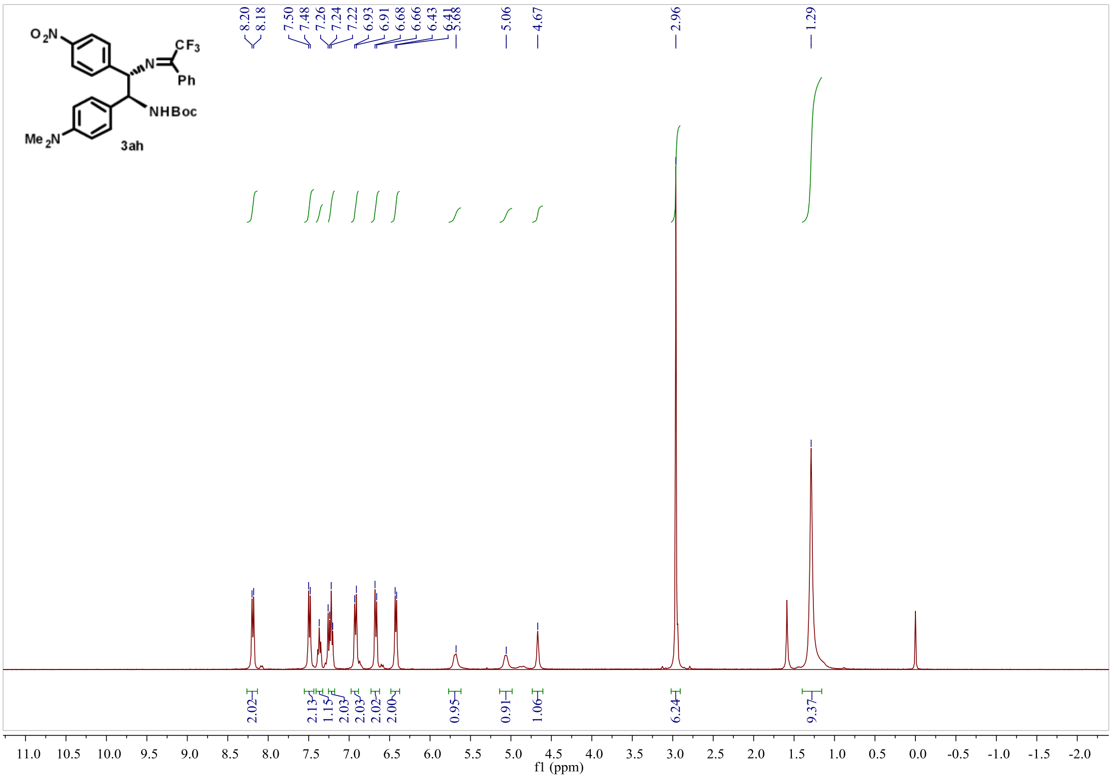

Supplementary Figure 82. ^{1}H NMR spectrum for compound **3ah**

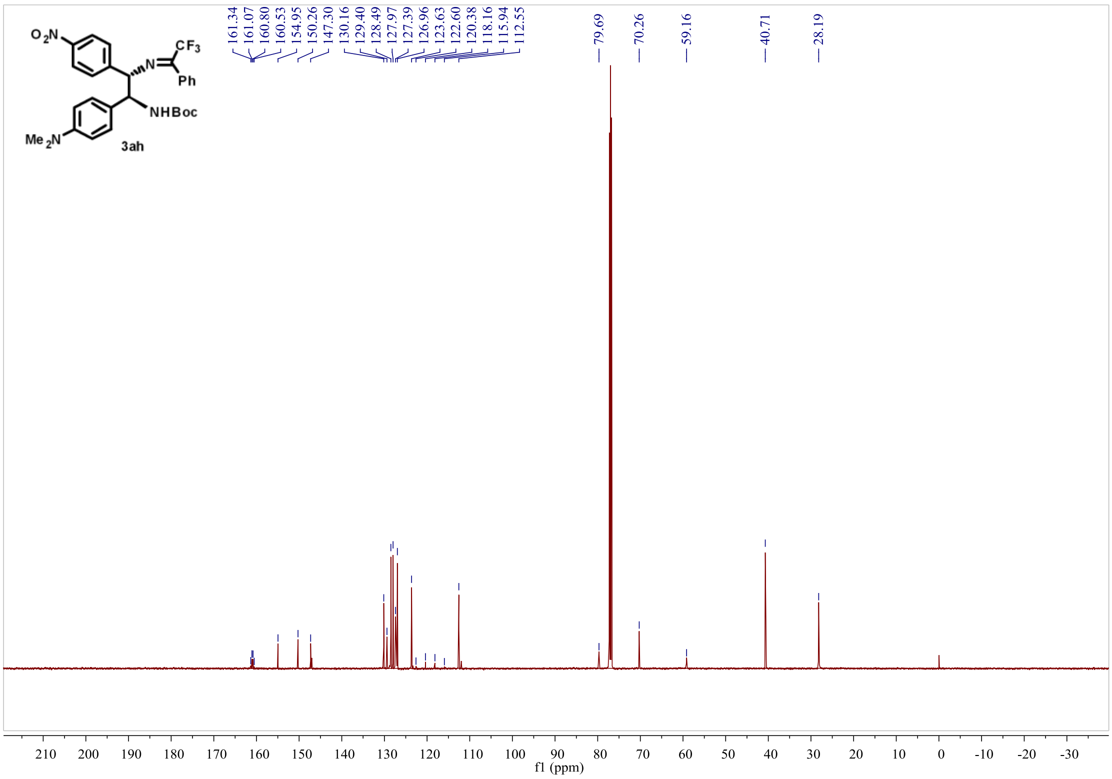

Supplementary Figure 83. ^{13}C NMR spectrum for compound **3ah**

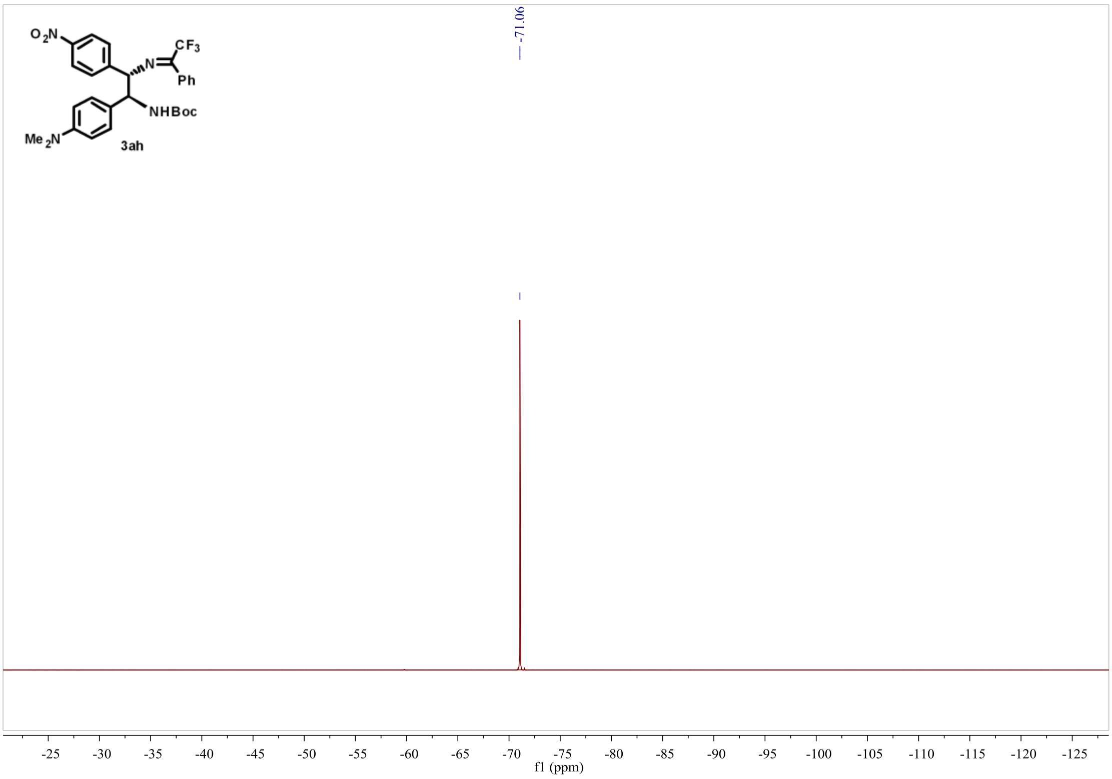

Supplementary Figure 84. ^{19}F NMR spectrum for compound **3ah**

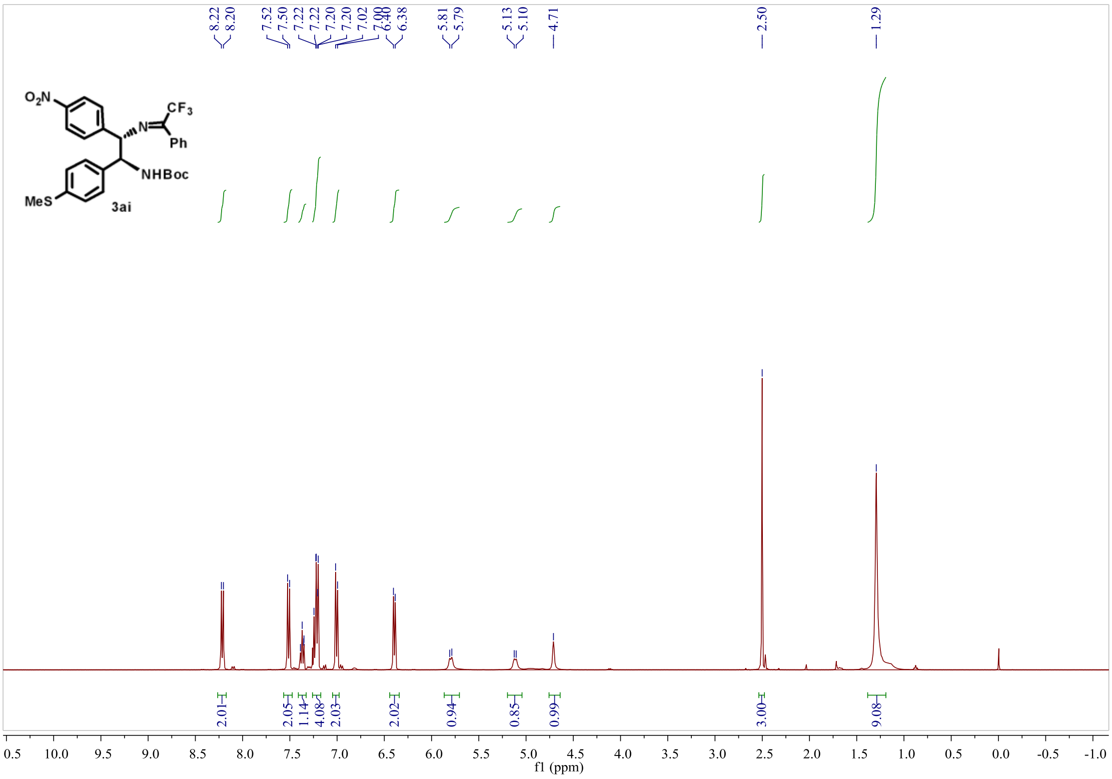

Supplementary Figure 85. ^{1}H NMR spectrum for compound **3ai**

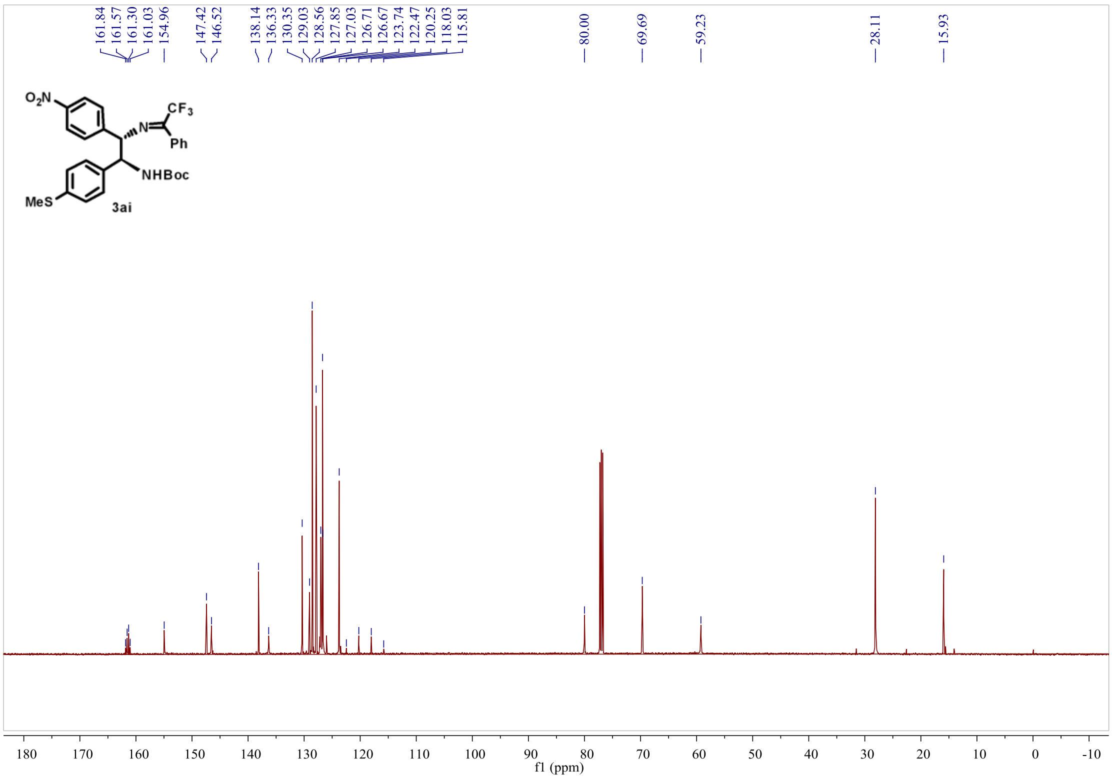

Supplementary Figure 86. ^{13}C NMR spectrum for compound **3ai**

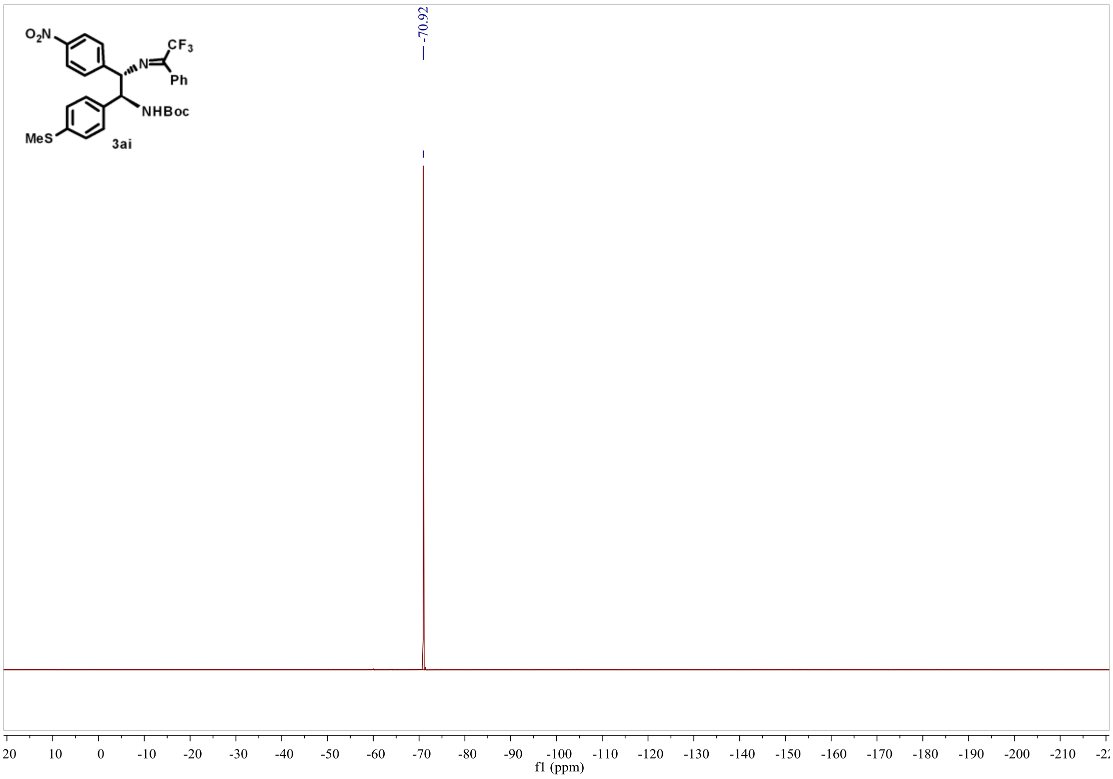

Supplementary Figure 87. ^{19}F NMR spectrum for compound **3ai**

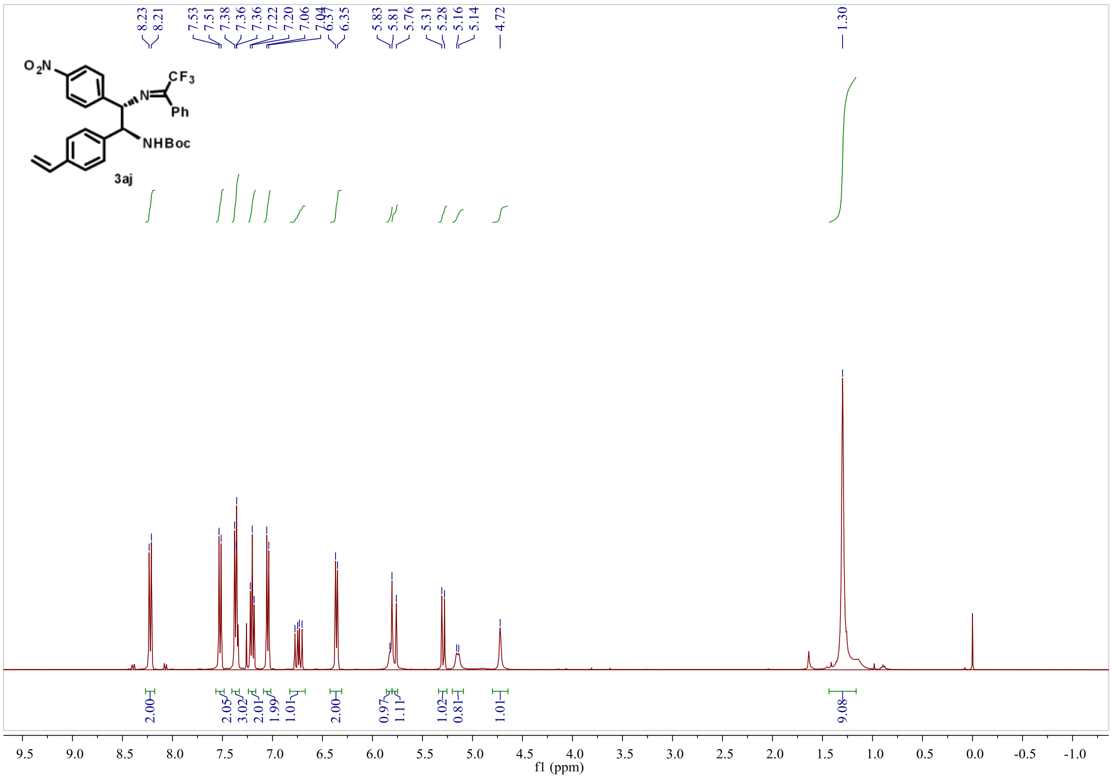

Supplementary Figure 88. ^{1}H NMR spectrum for compound **3aj**

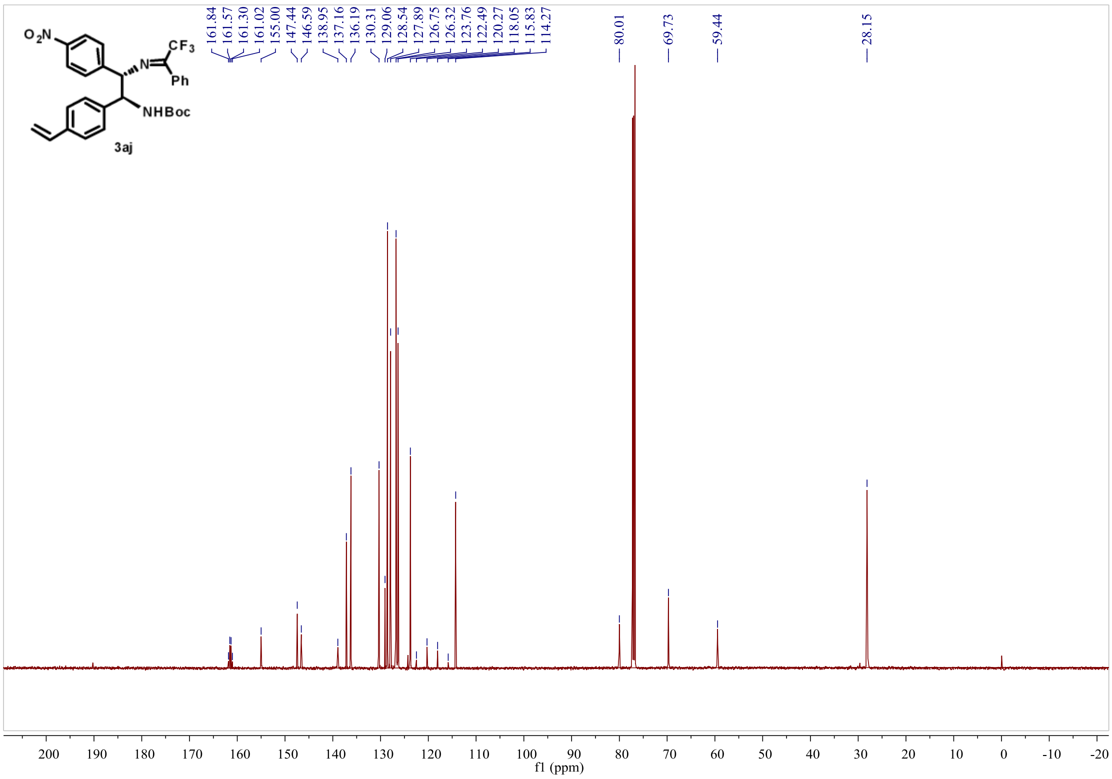

Supplementary Figure 89. ^{13}C NMR spectrum for compound **3aj**

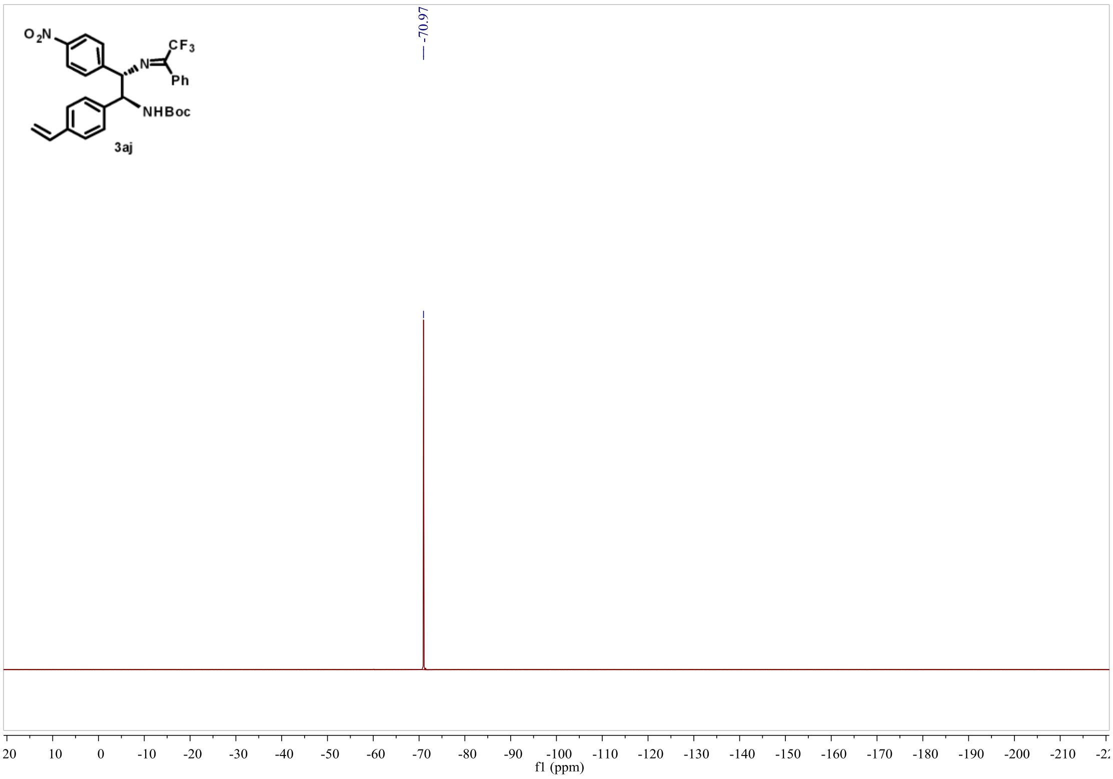

Supplementary Figure 90. ^{19}F NMR spectrum for compound **3aj**

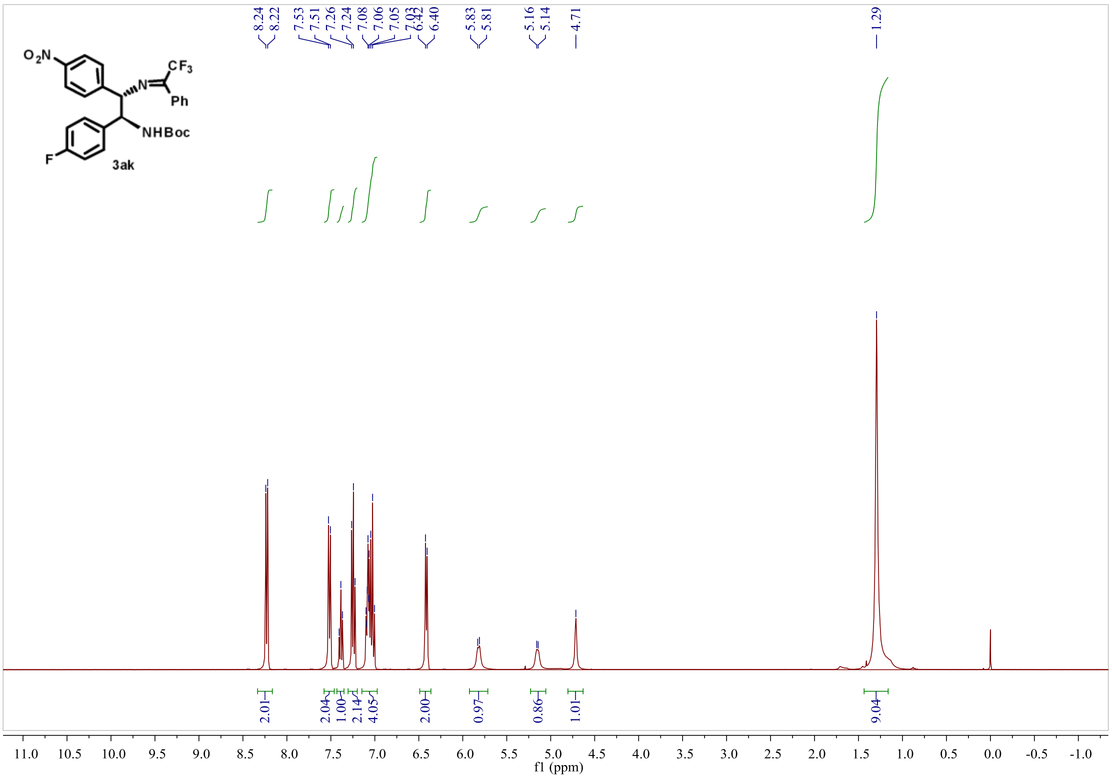

Supplementary Figure 91. ^{1}H NMR spectrum for compound **3ak**

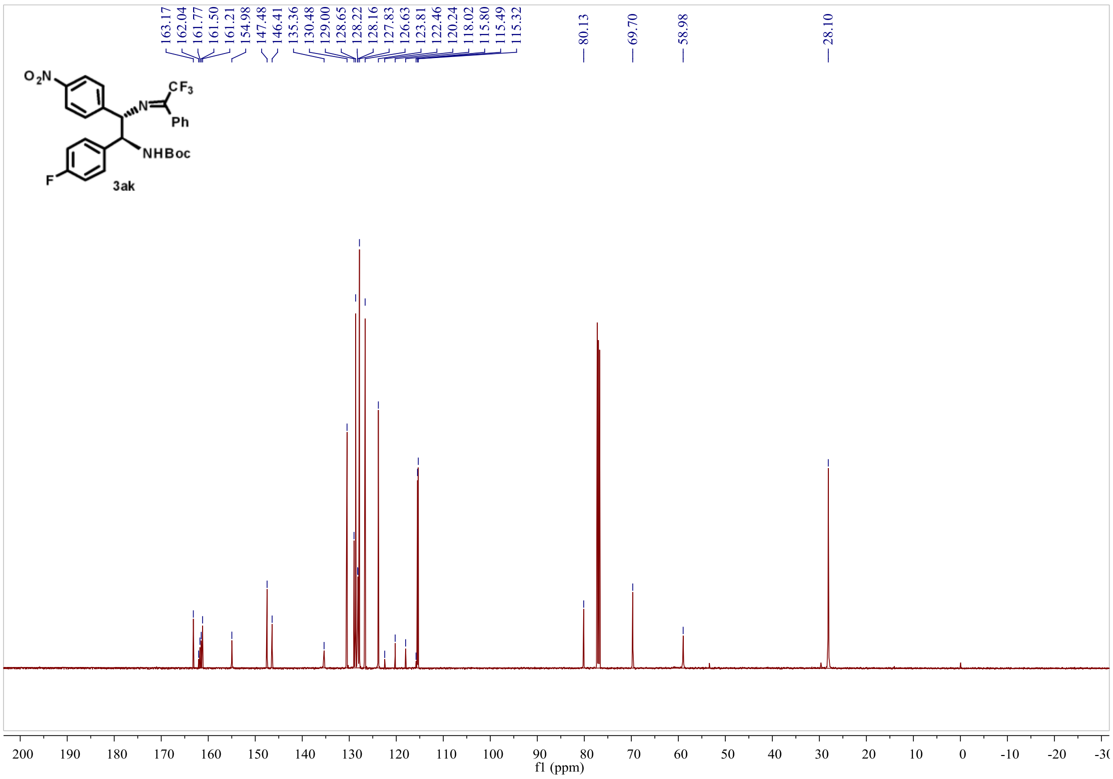

Supplementary Figure 92. ^{13}C NMR spectrum for compound **3ak**

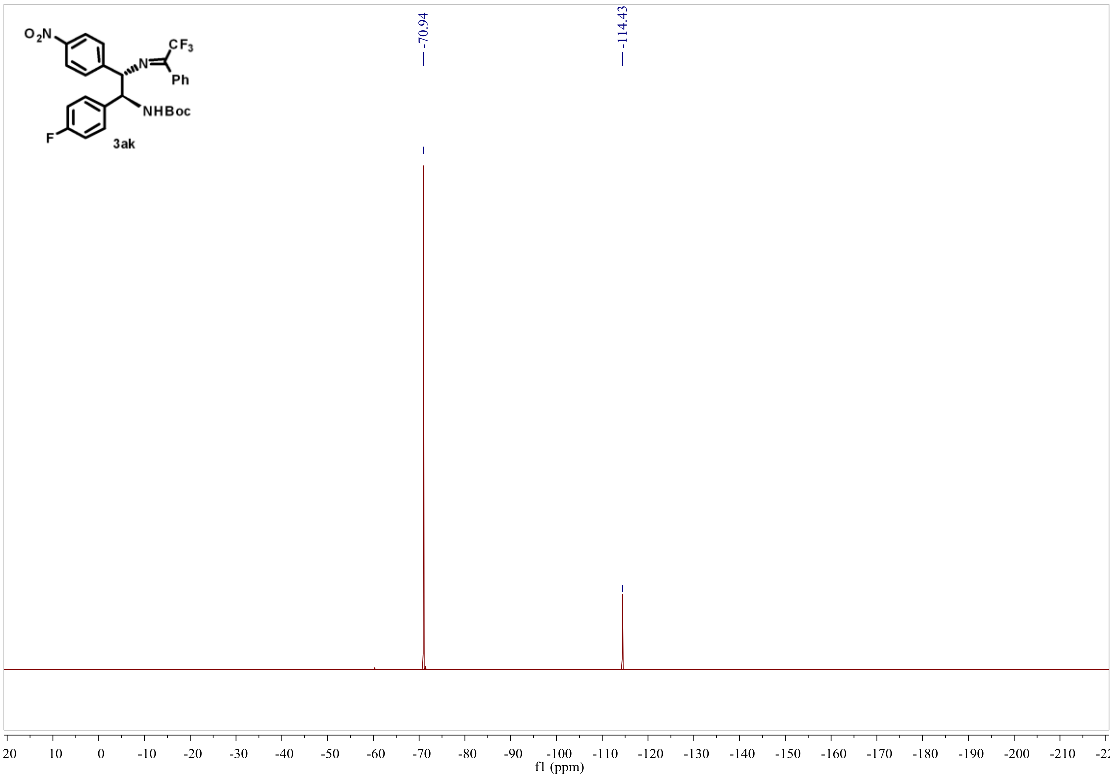

Supplementary Figure 93. ^{19}F NMR spectrum for compound **3ak**

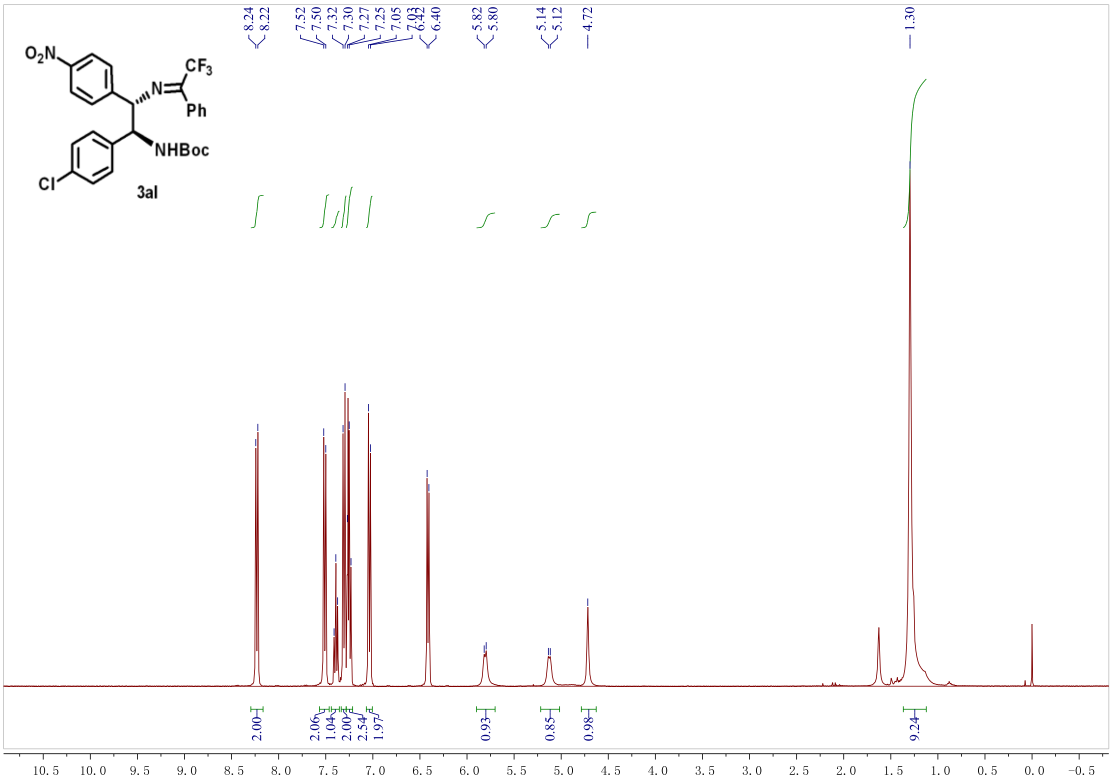

Supplementary Figure 94. ^{1}H NMR spectrum for compound **3al**

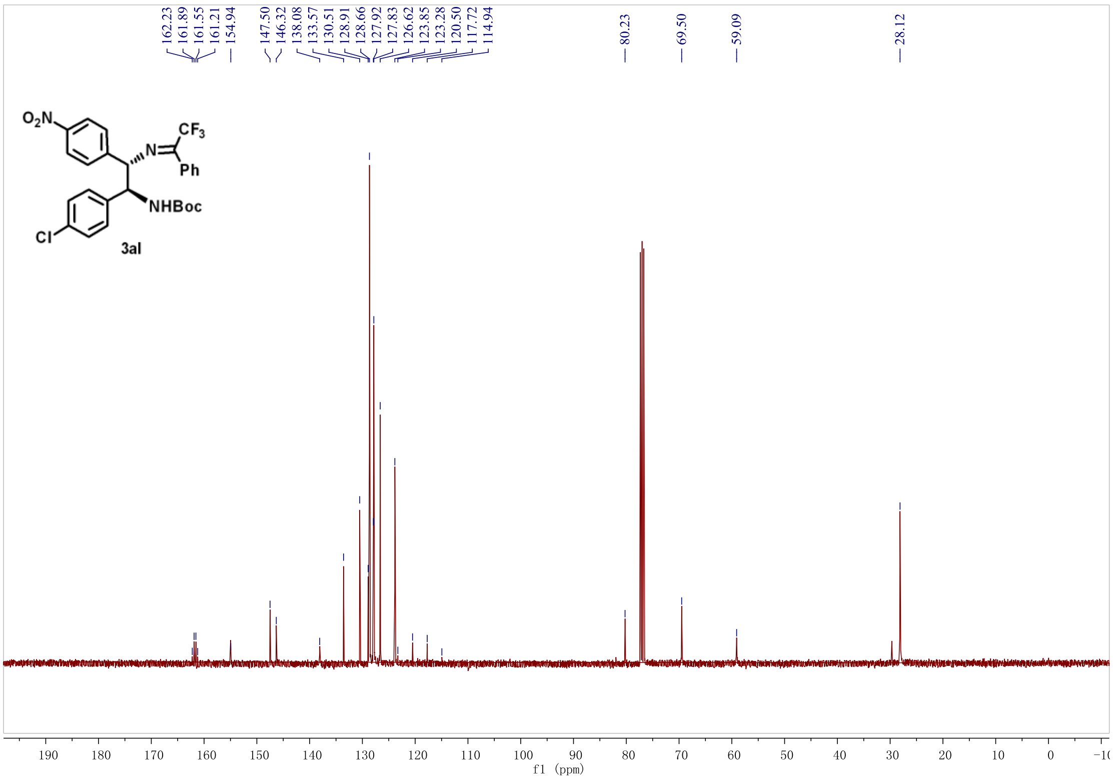

Supplementary Figure 95. ^{13}C NMR spectrum for compound **3al**

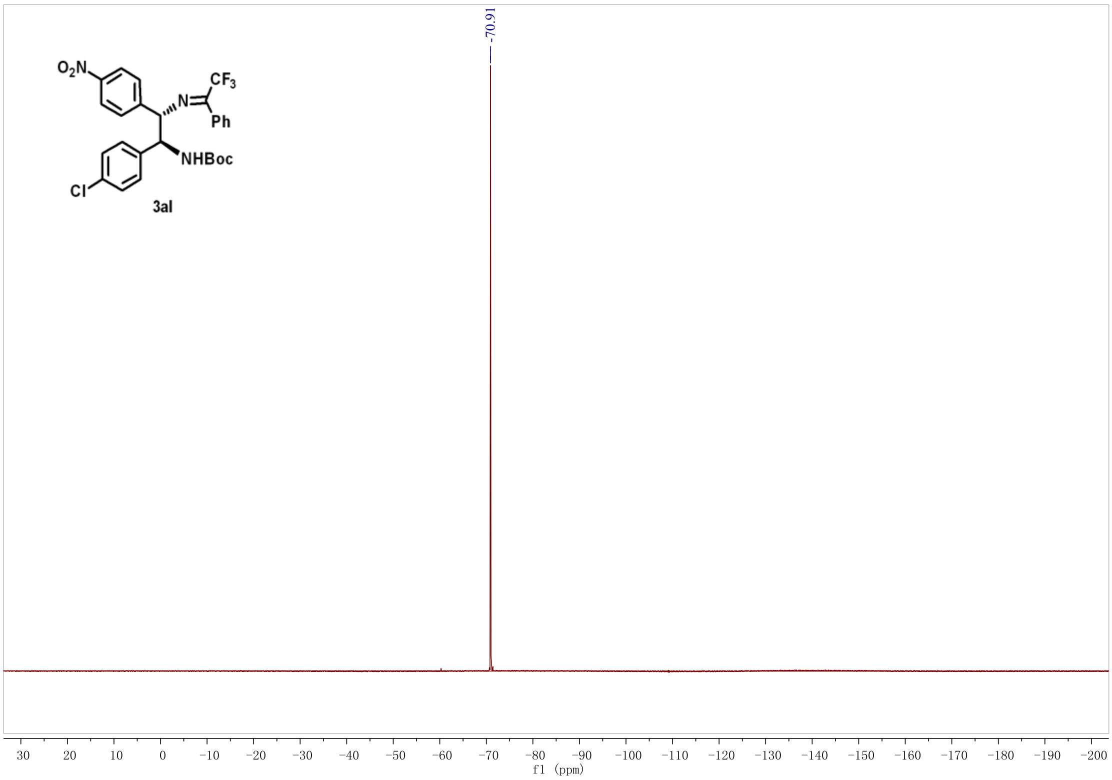

Supplementary Figure 96. ^{19}F NMR spectrum for compound **3al**

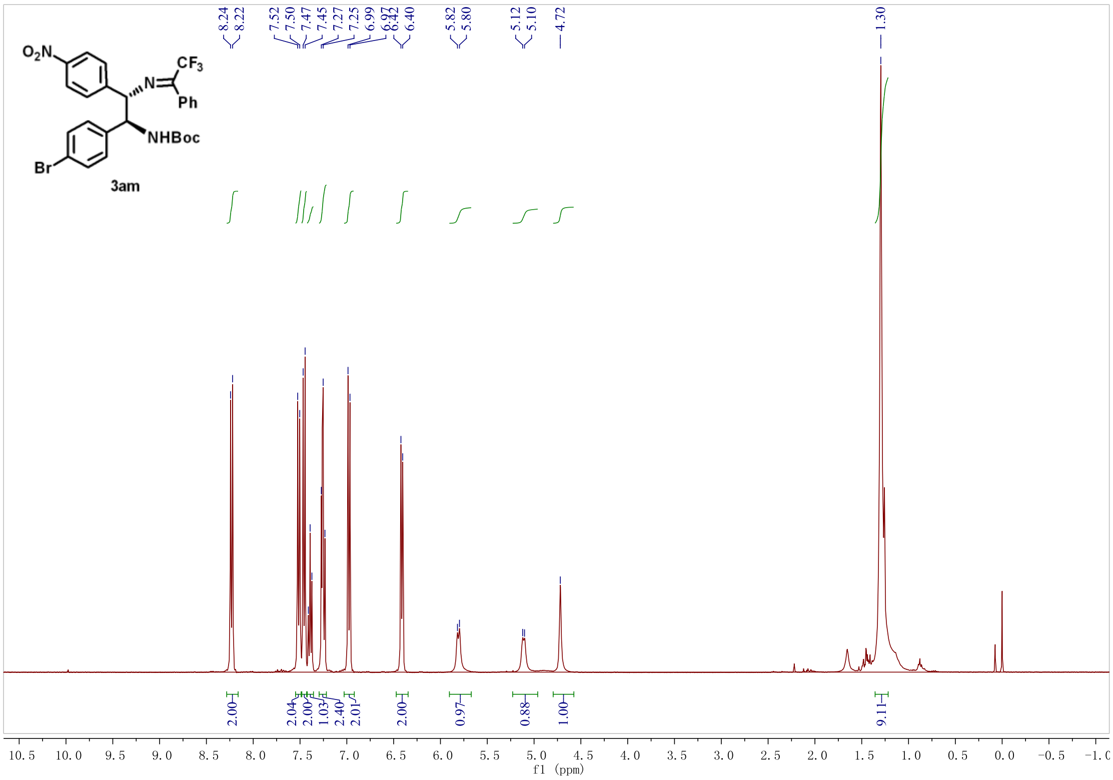

Supplementary Figure 97. ^{1}H NMR spectrum for compound **3am**

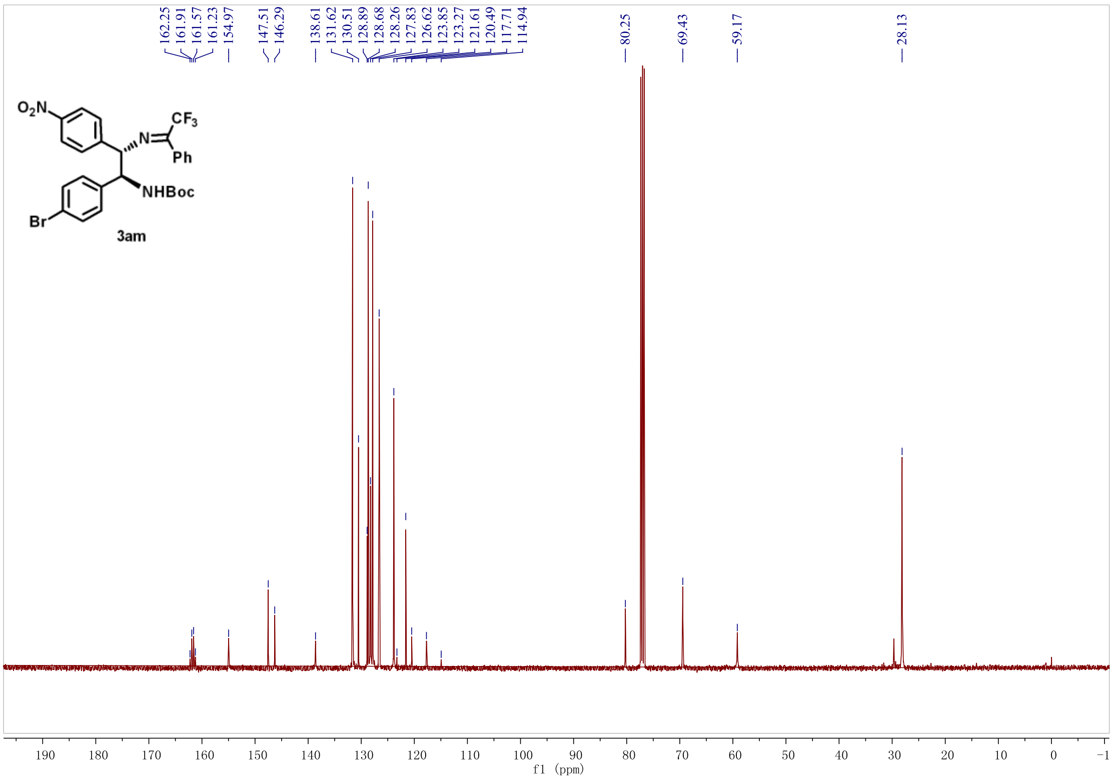

Supplementary Figure 98. ^{13}C NMR spectrum for compound **3am**

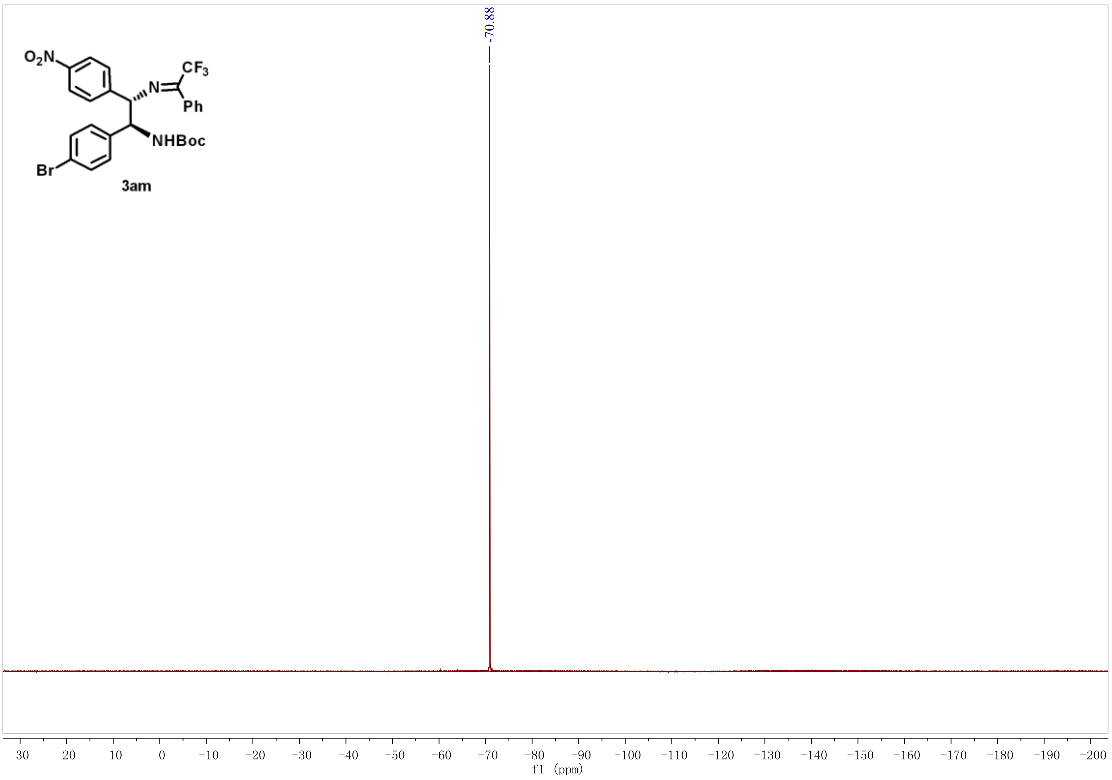

Supplementary Figure 99. ^{19}F NMR spectrum for compound **3am**

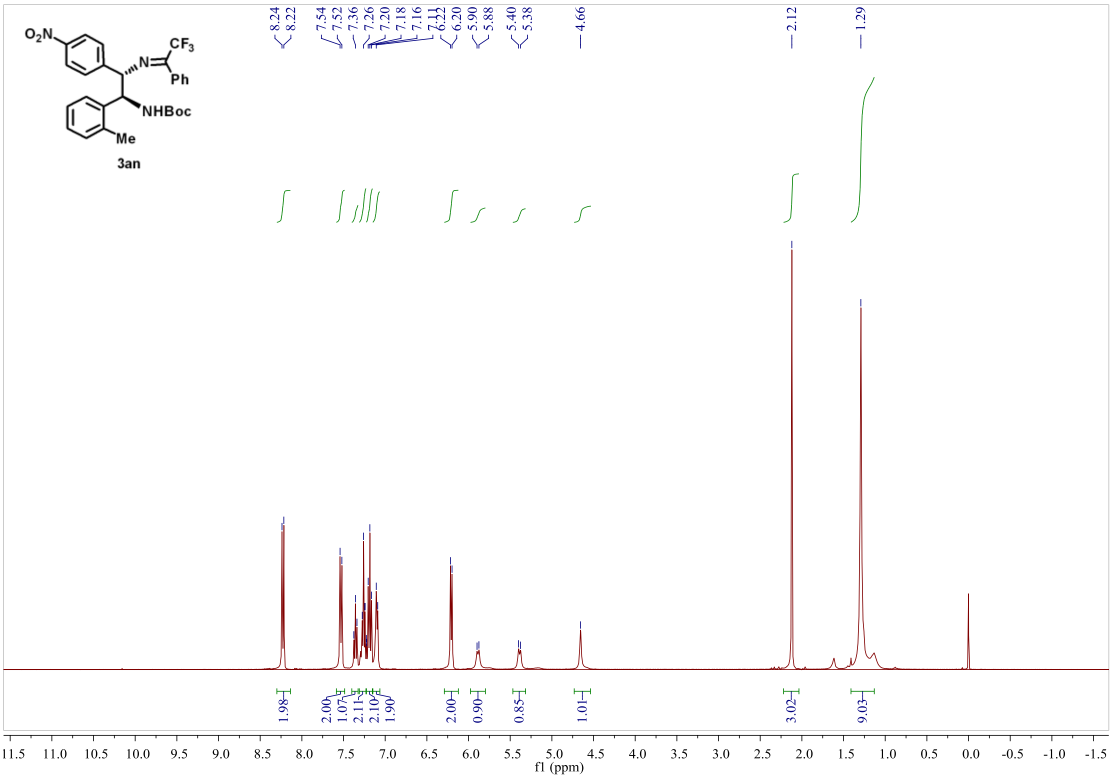

Supplementary Figure 100. ^{1}H NMR spectrum for compound **3an**

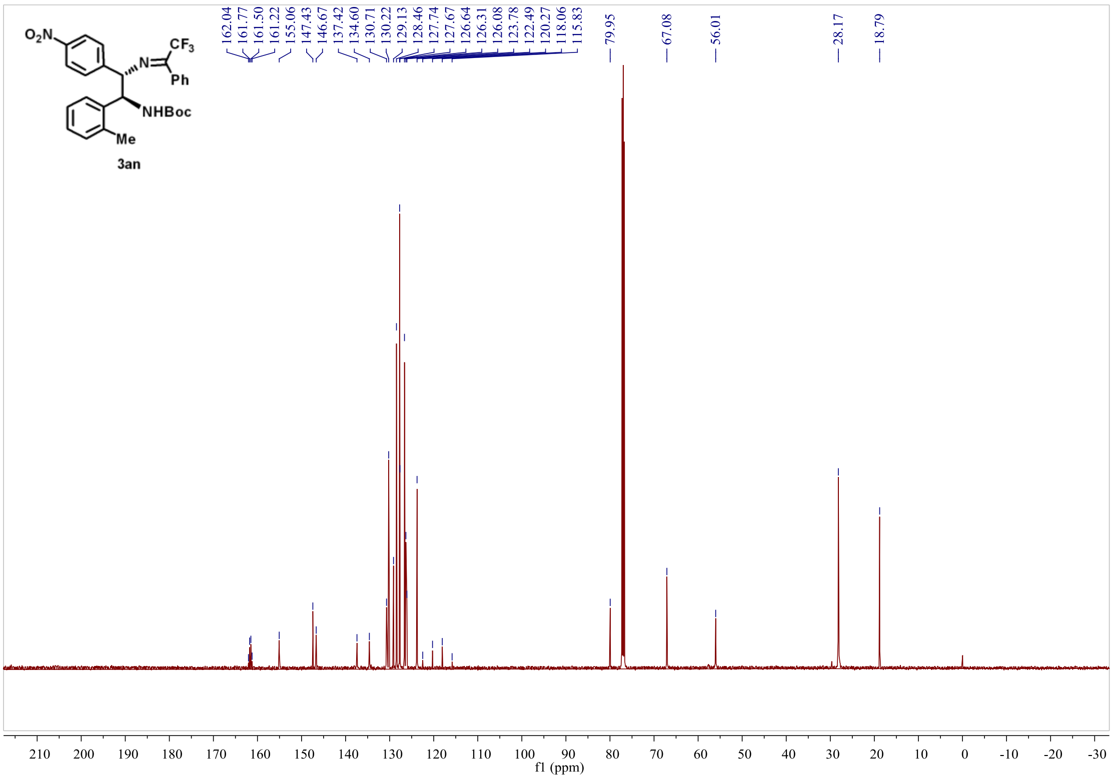

Supplementary Figure 101. ^{13}C NMR spectrum for compound **3an**

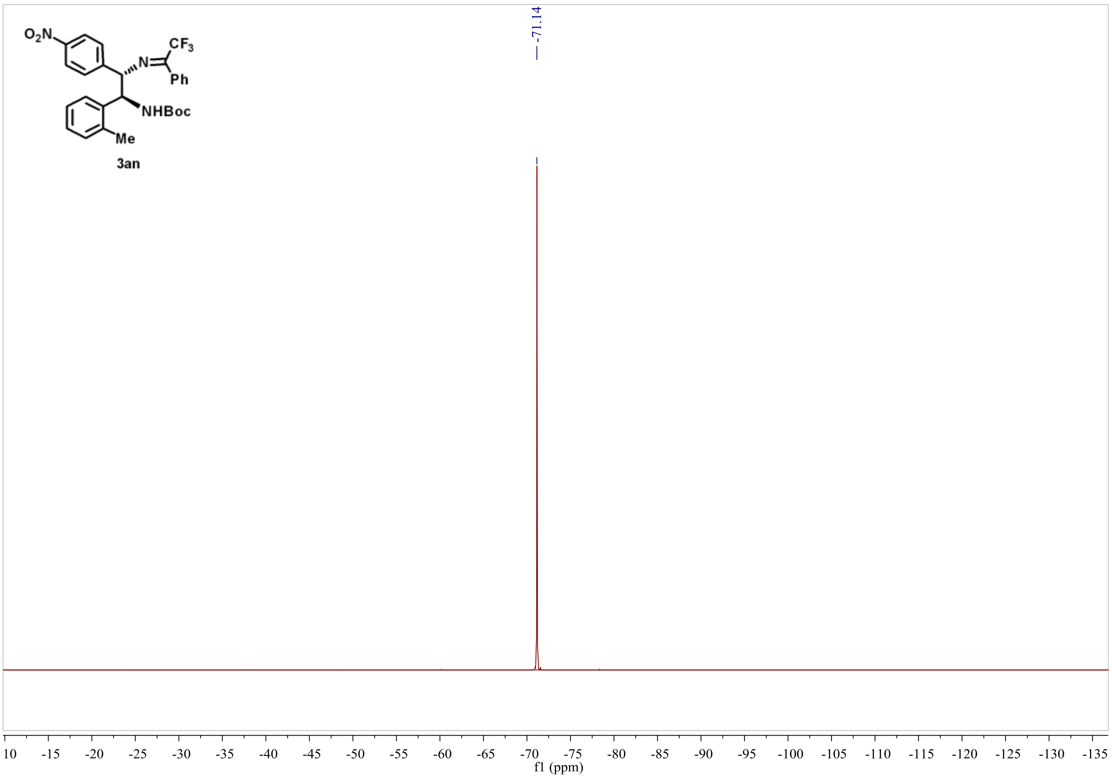

Supplementary Figure 102. ^{19}F NMR spectrum for compound **3an**

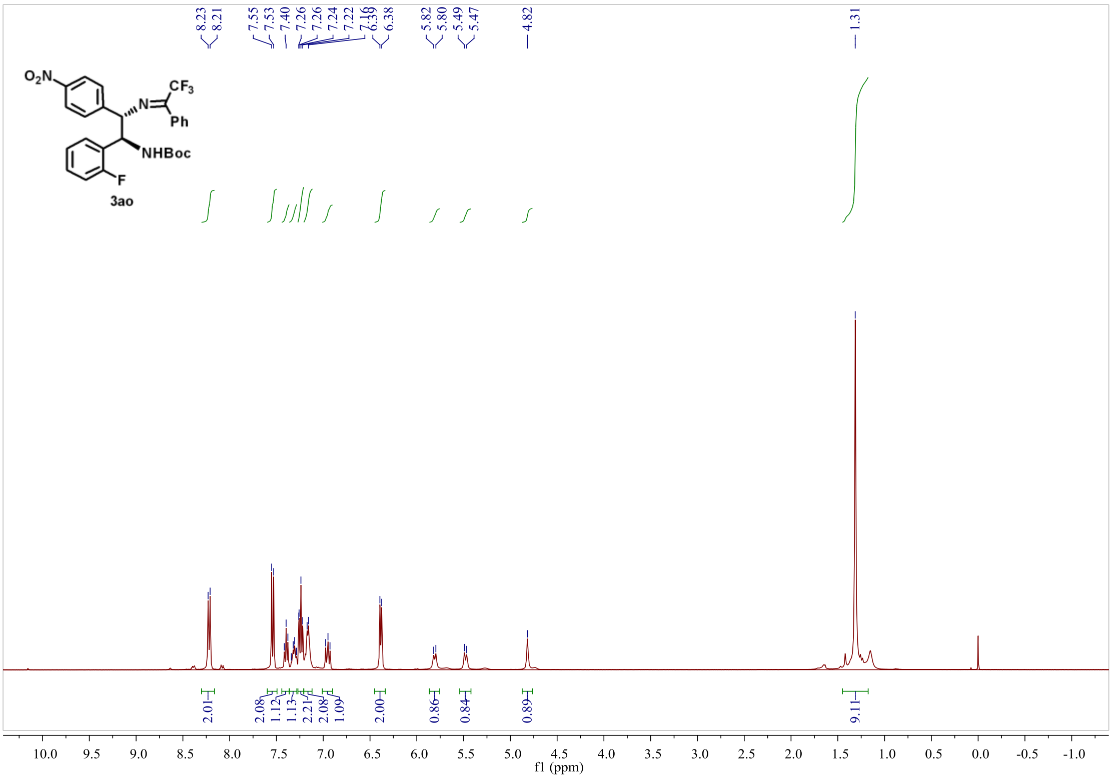

Supplementary Figure 103. ^{1}H NMR spectrum for compound **3ao**

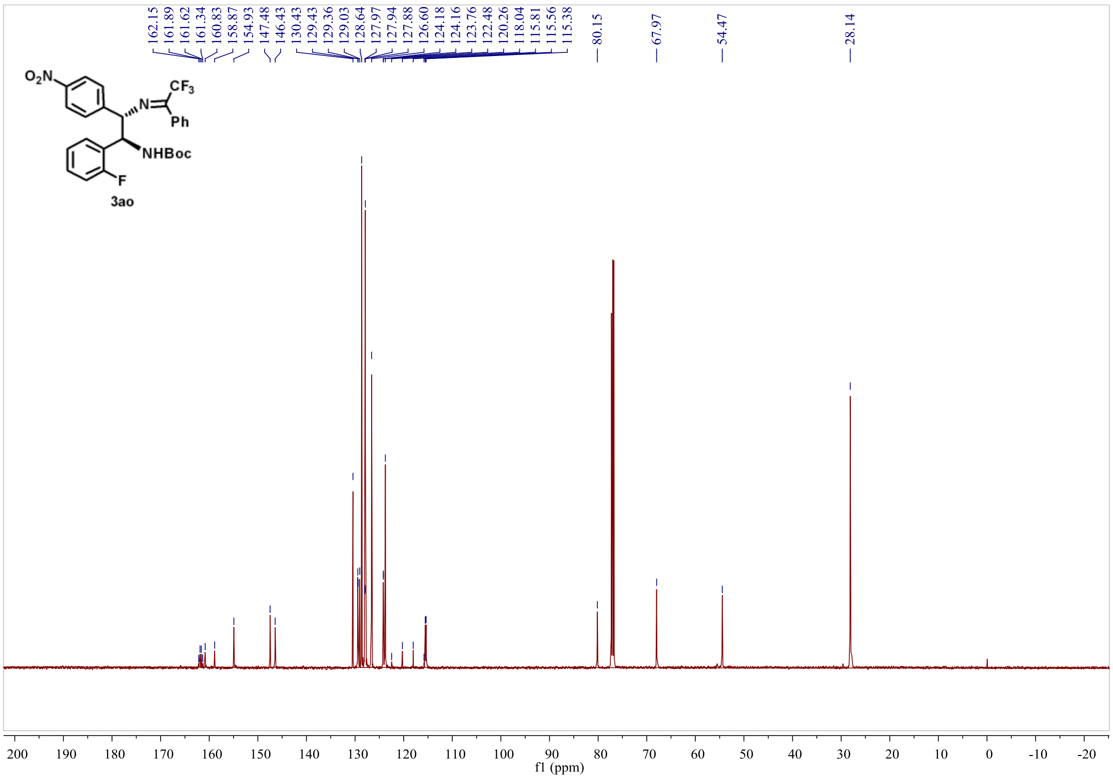

Supplementary Figure 104. ^{13}C NMR spectrum for compound **3ao**

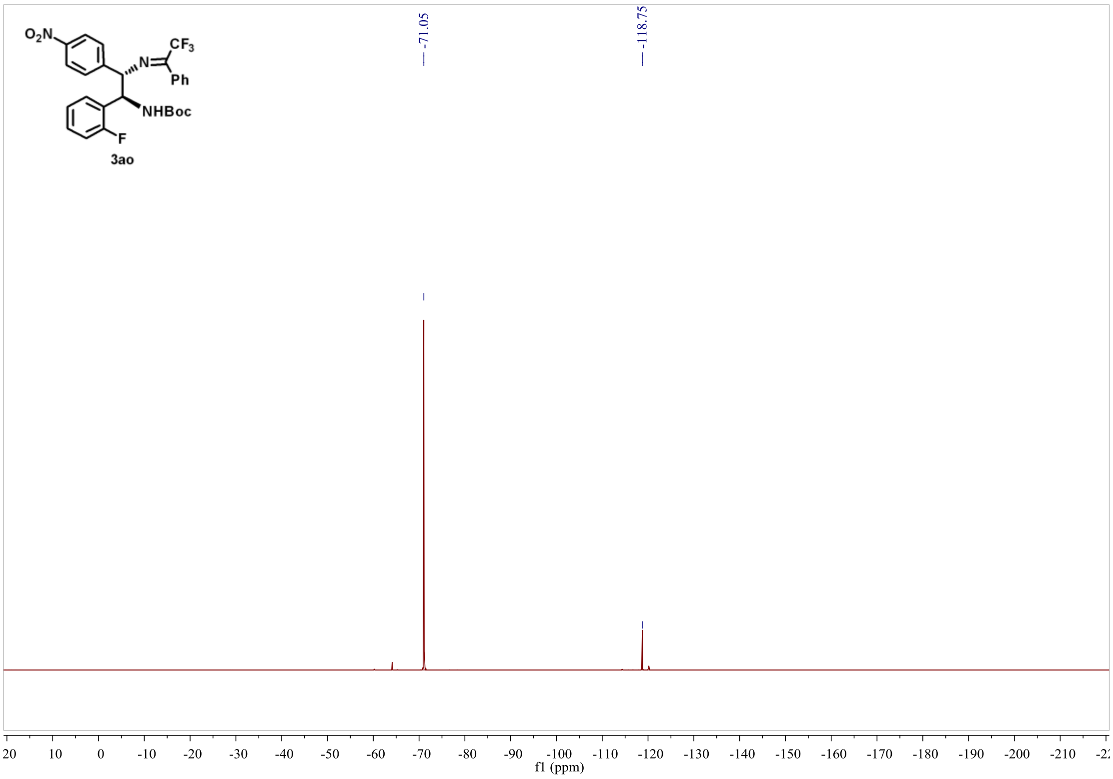

Supplementary Figure 105. ^{19}F NMR spectrum for compound **3ao**

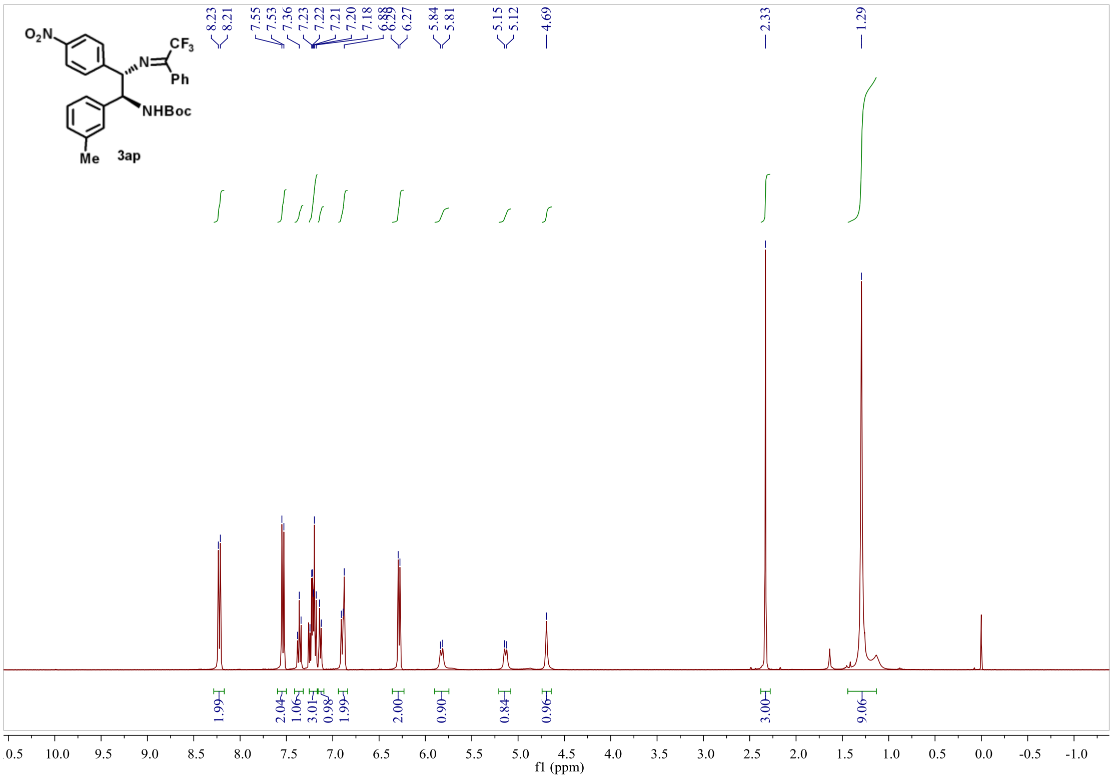

Supplementary Figure 106. ^{1}H NMR spectrum for compound **3ap**

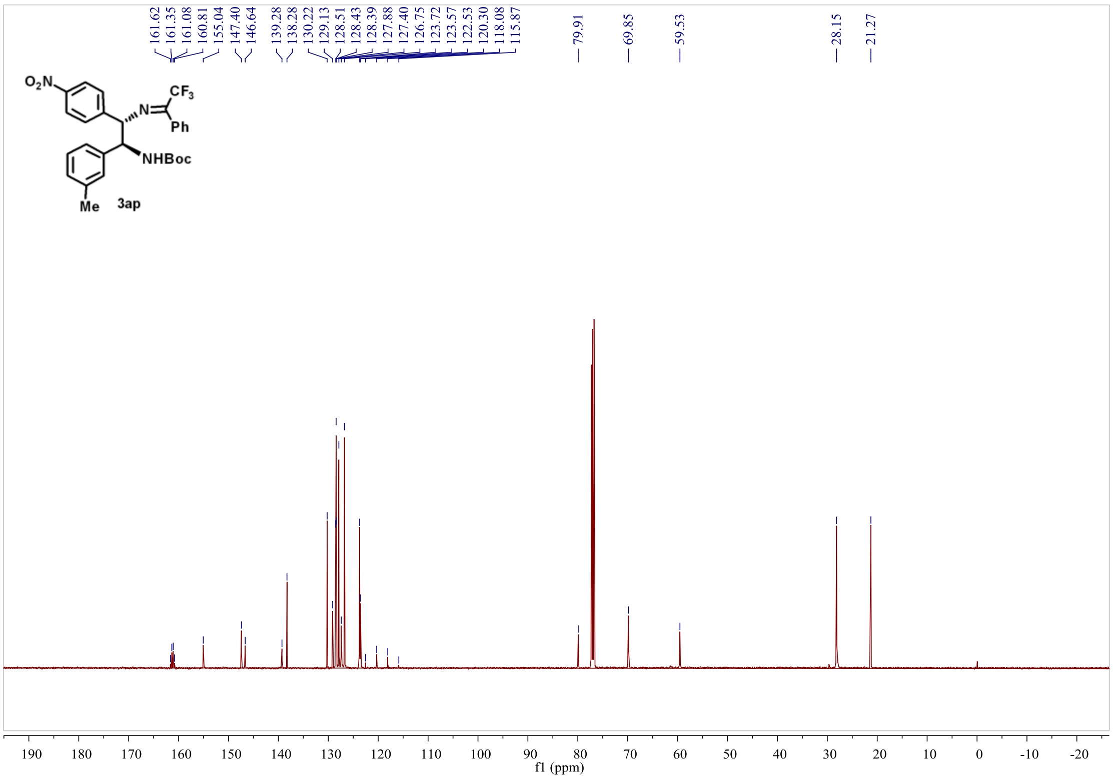

Supplementary Figure 107. ^{13}C NMR spectrum for compound **3ap**

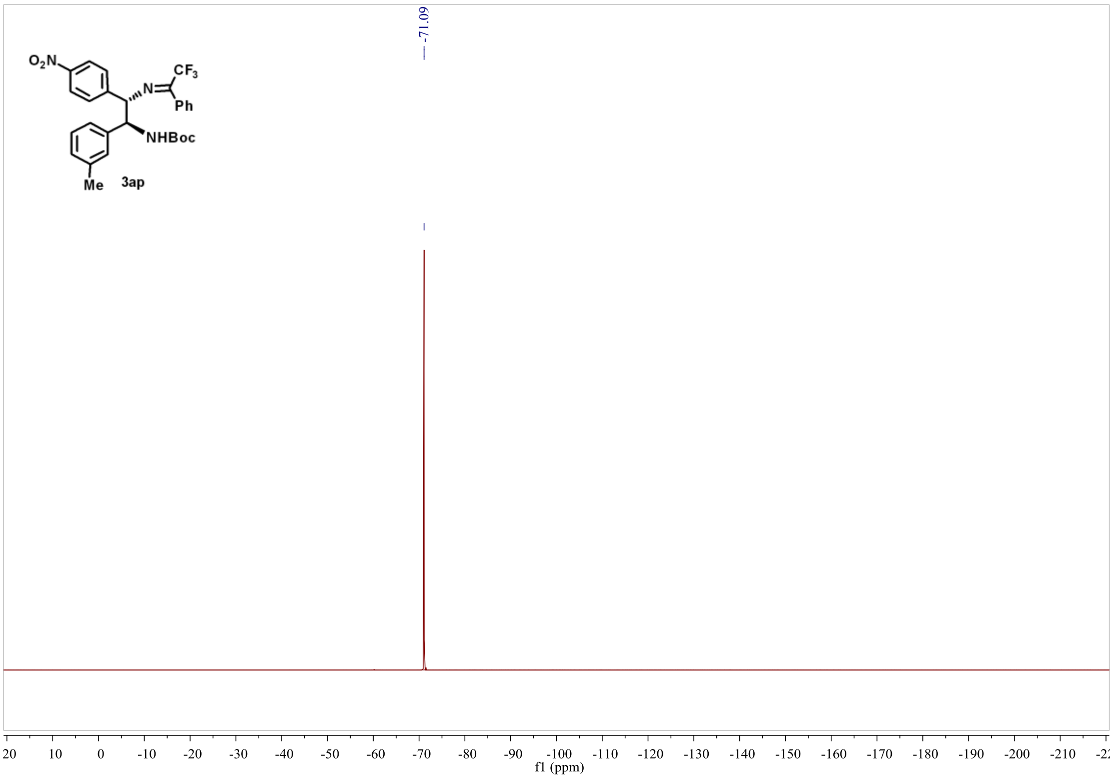

Supplementary Figure 108. ^{19}F NMR spectrum for compound **3ap**

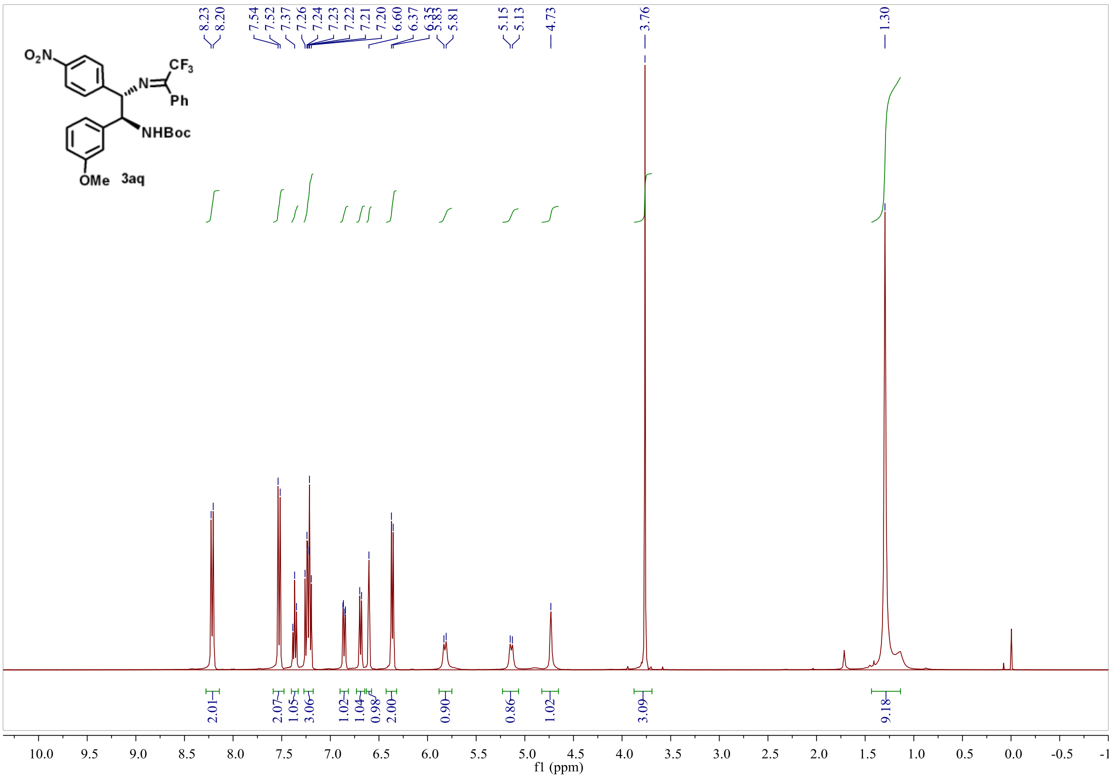

Supplementary Figure 109. ^{1}H NMR spectrum for compound **3aq**

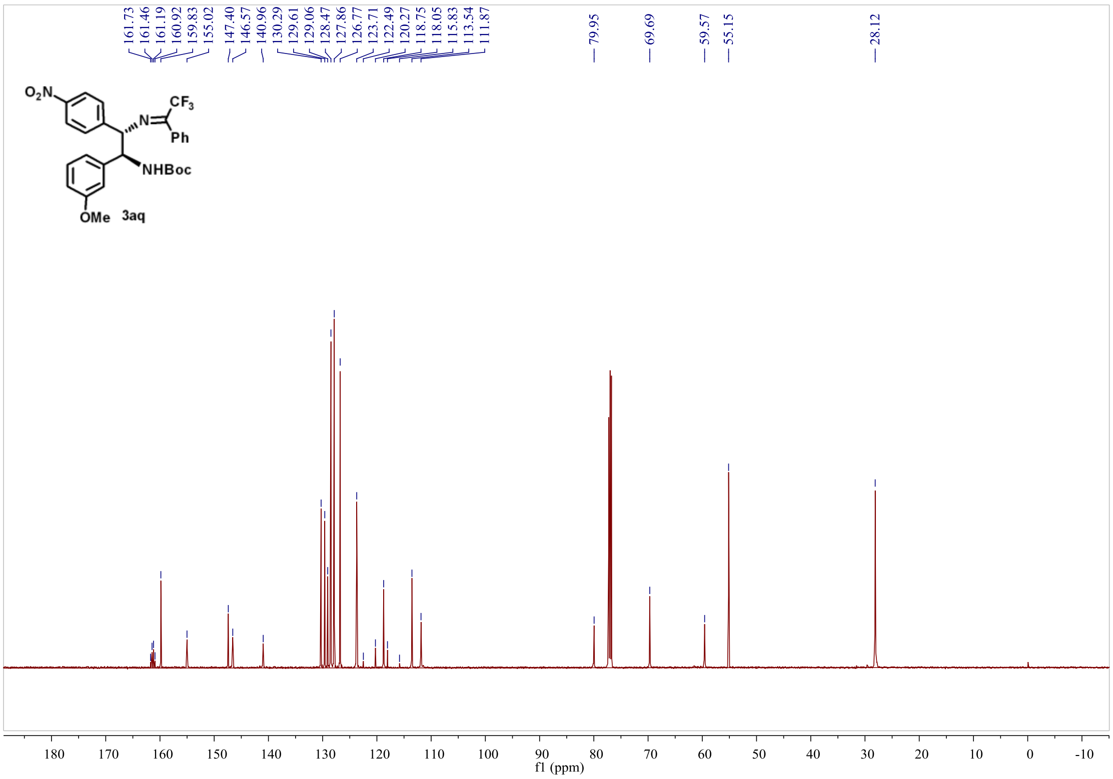

Supplementary Figure 110. ^{13}C NMR spectrum for compound **3aq**

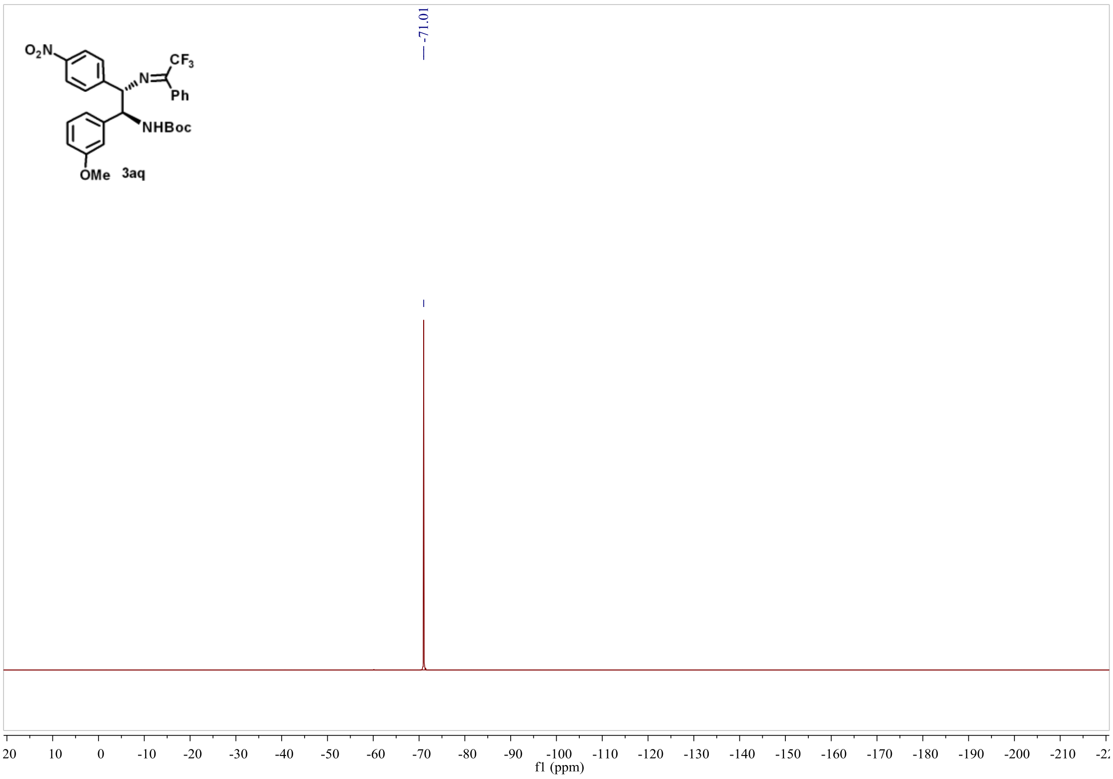

Supplementary Figure 111. ^{19}F NMR spectrum for compound **3aq**

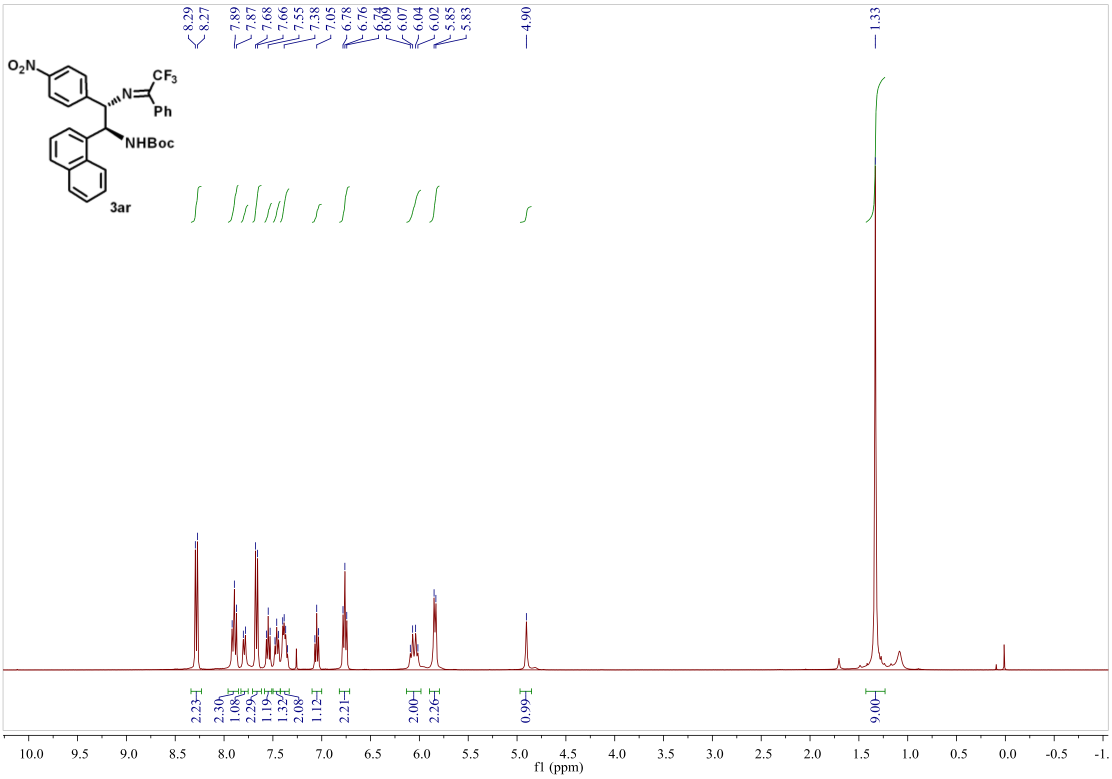

Supplementary Figure 112. ^{1}H NMR spectrum for compound **3ar**

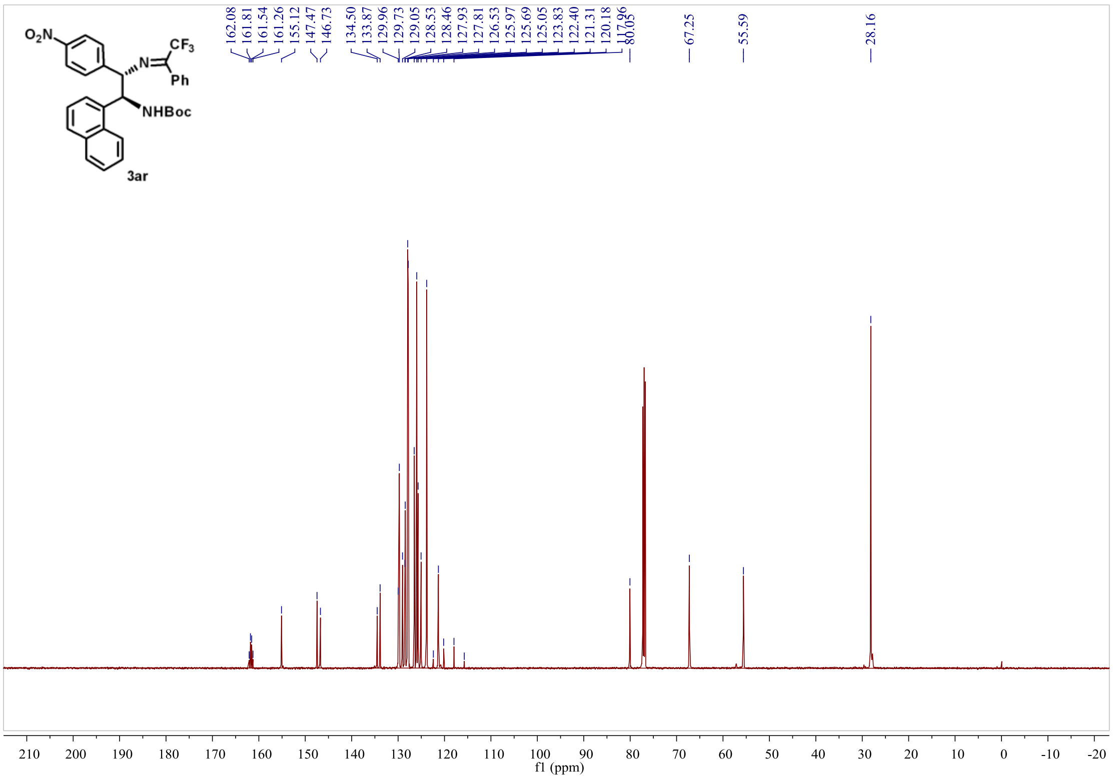

Supplementary Figure 113. ^{13}C NMR spectrum for compound **3ar**

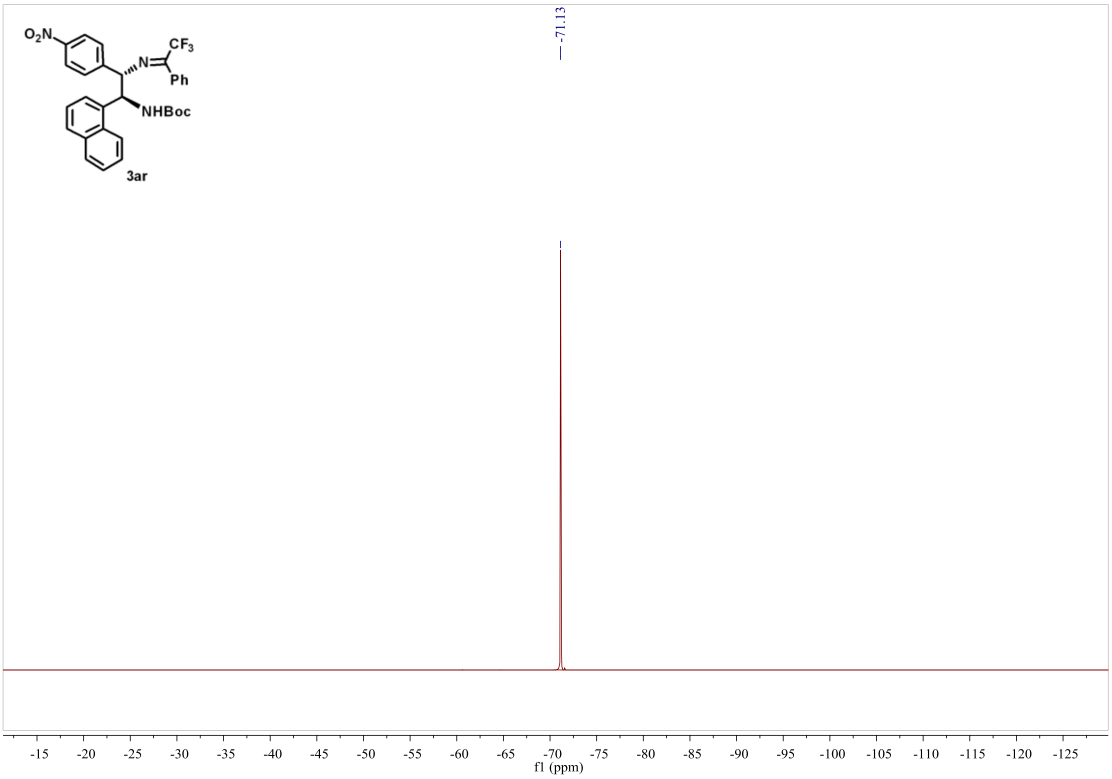

Supplementary Figure 114. ^{19}F NMR spectrum for compound **3ar**

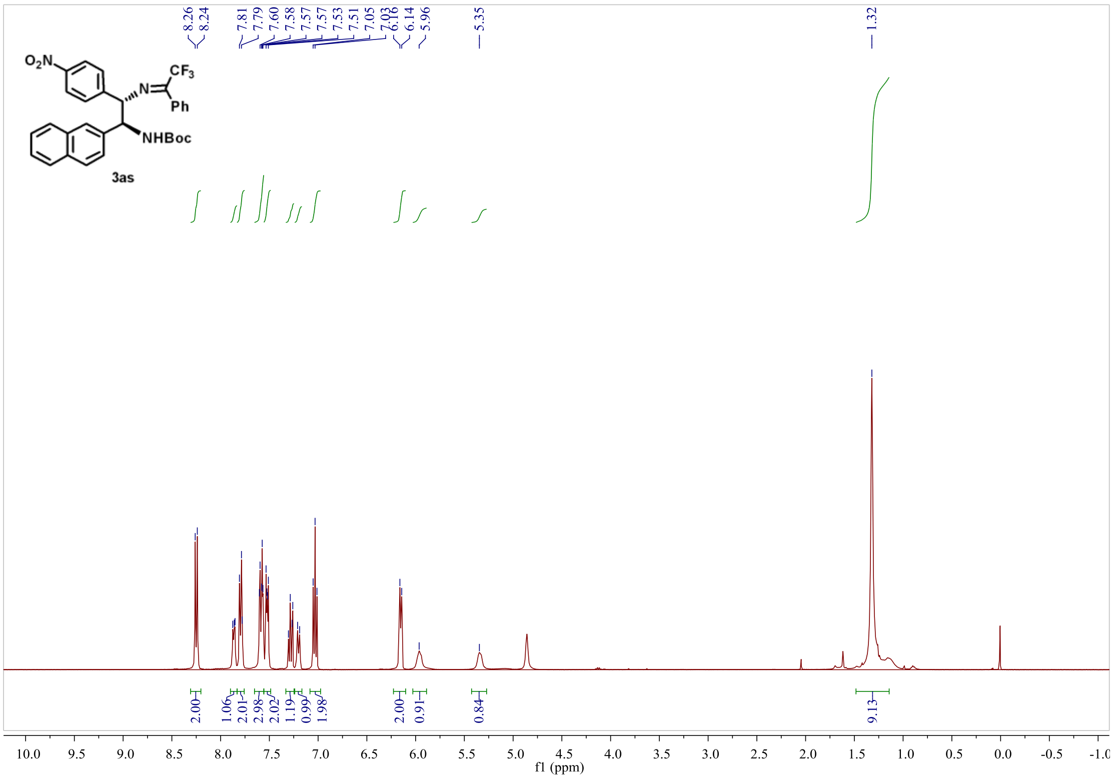

Supplementary Figure 115. ^{1}H NMR spectrum for compound **3as**

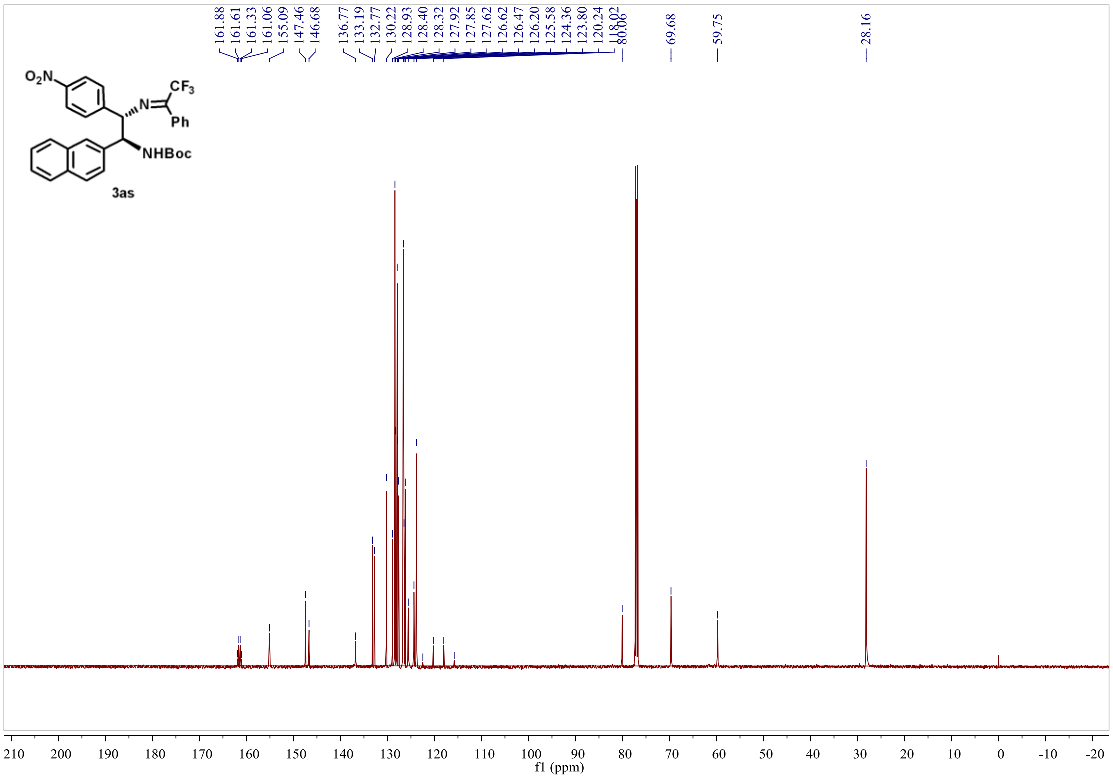

Supplementary Figure 116. ^{13}C NMR spectrum for compound **3as**

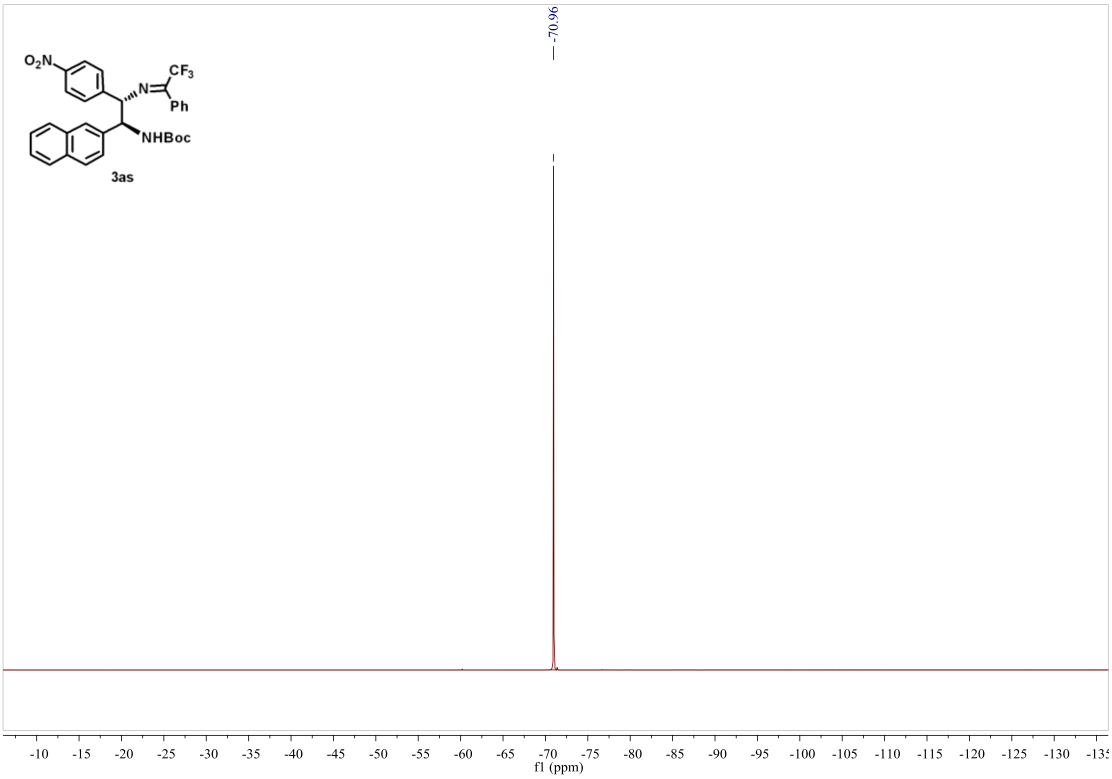

Supplementary Figure 117. ^{19}F NMR spectrum for compound **3as**

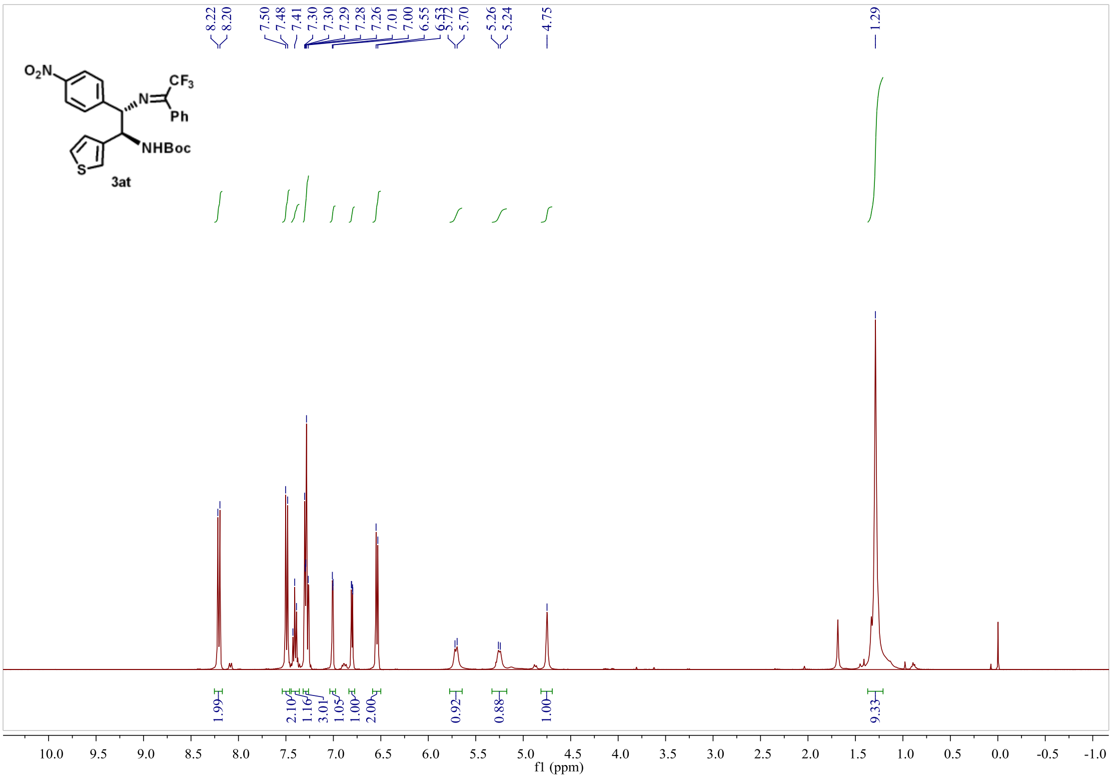

Supplementary Figure 118. ^{1}H NMR spectrum for compound **3at**

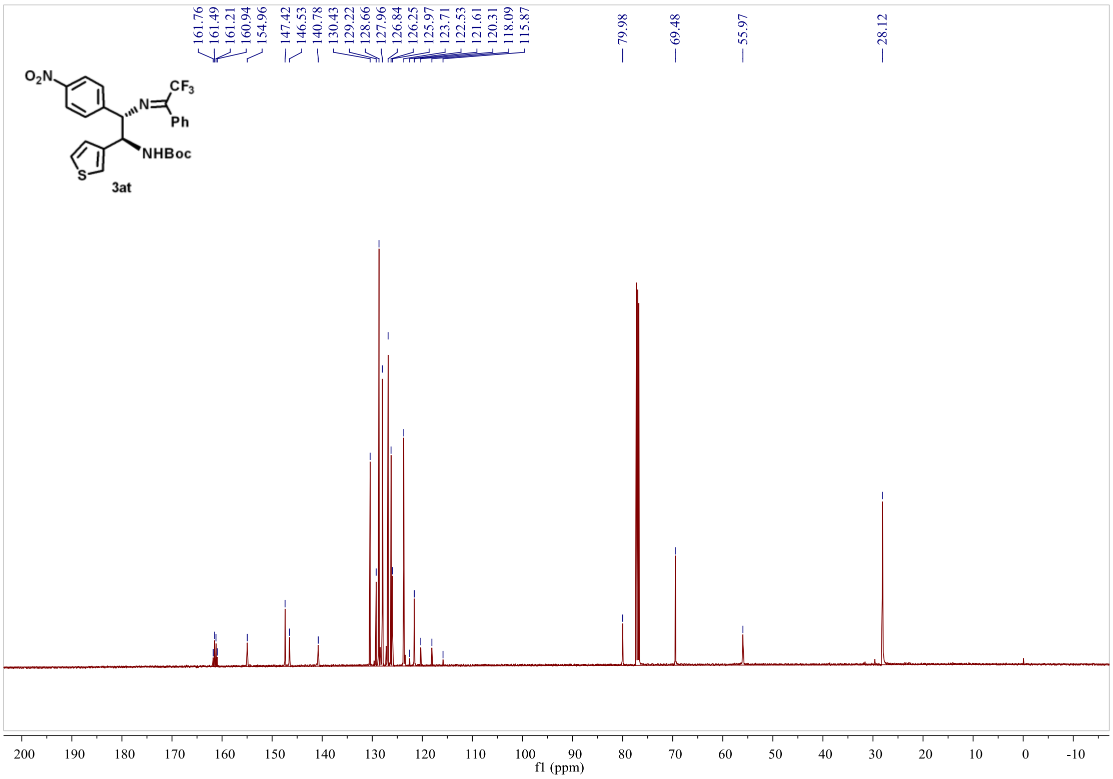

Supplementary Figure 119. ^{13}C NMR spectrum for compound **3at**

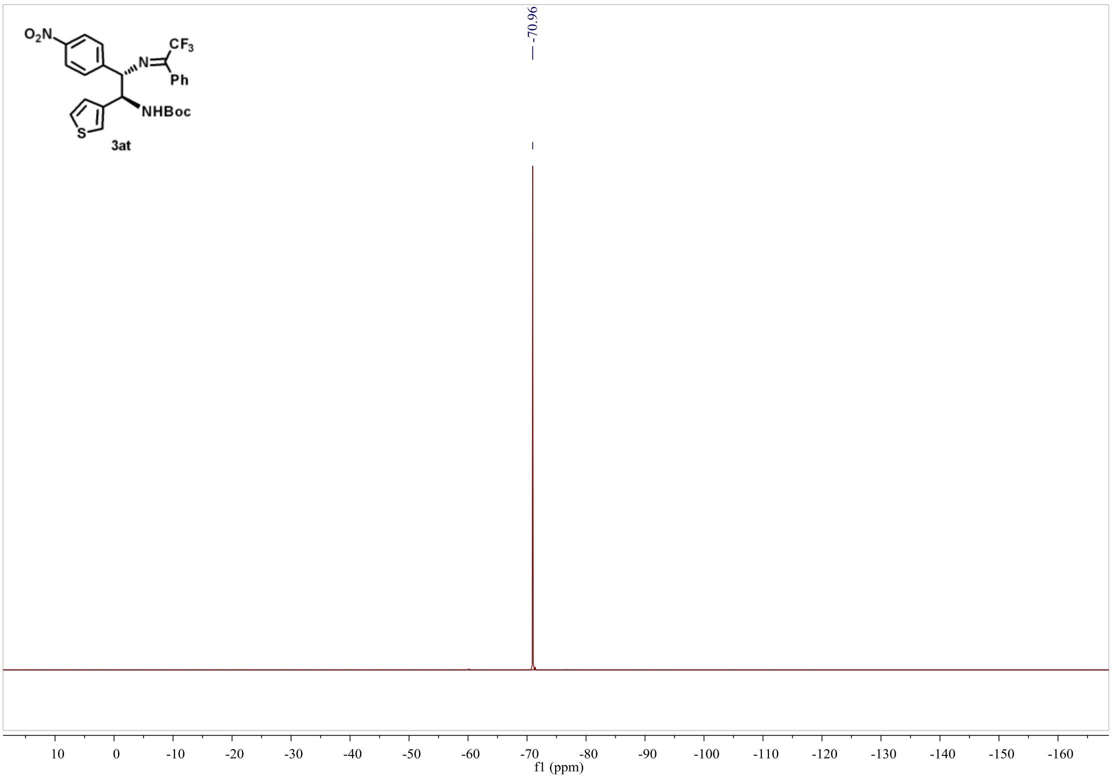

Supplementary Figure 120. ^{19}F NMR spectrum for compound **3at**

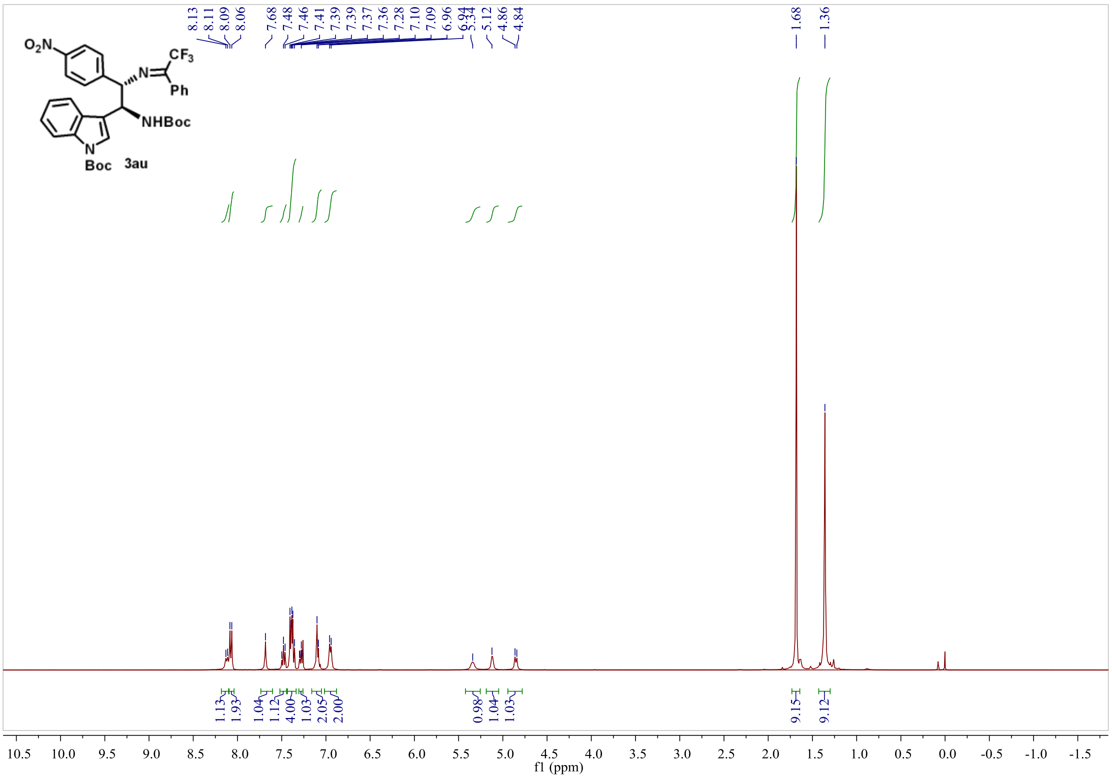

Supplementary Figure 121. ^{1}H NMR spectrum for compound **3au**

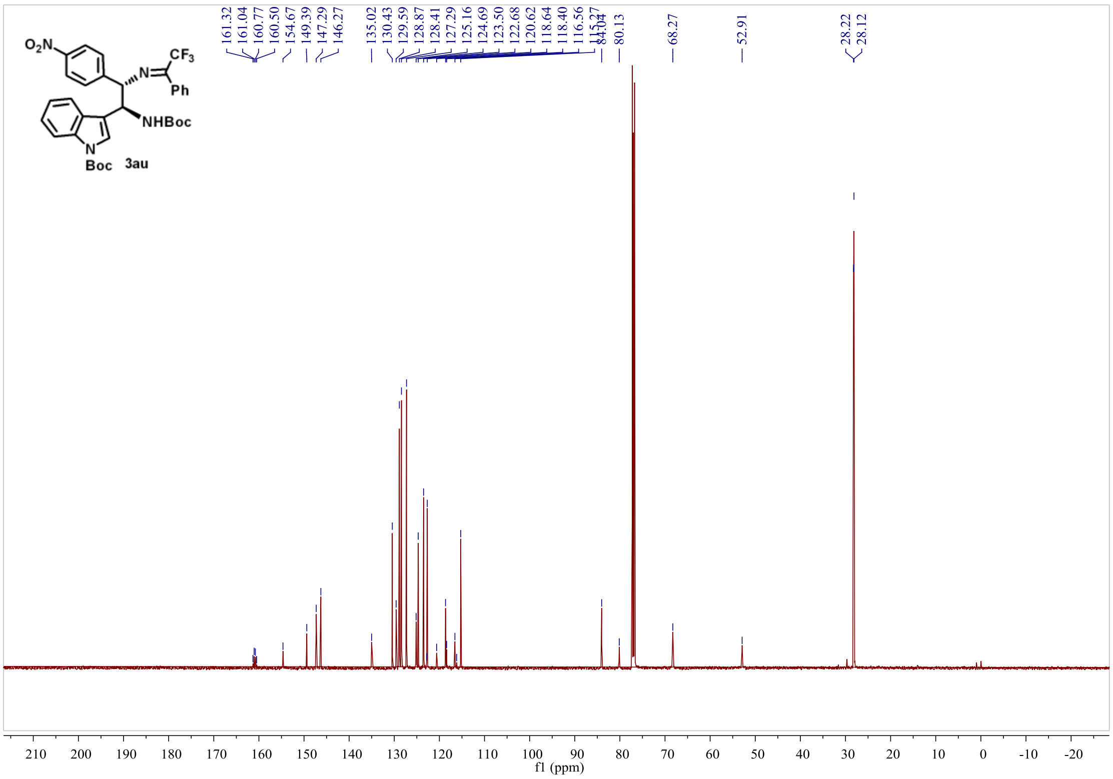

Supplementary Figure 122. ^{13}C NMR spectrum for compound **3au**

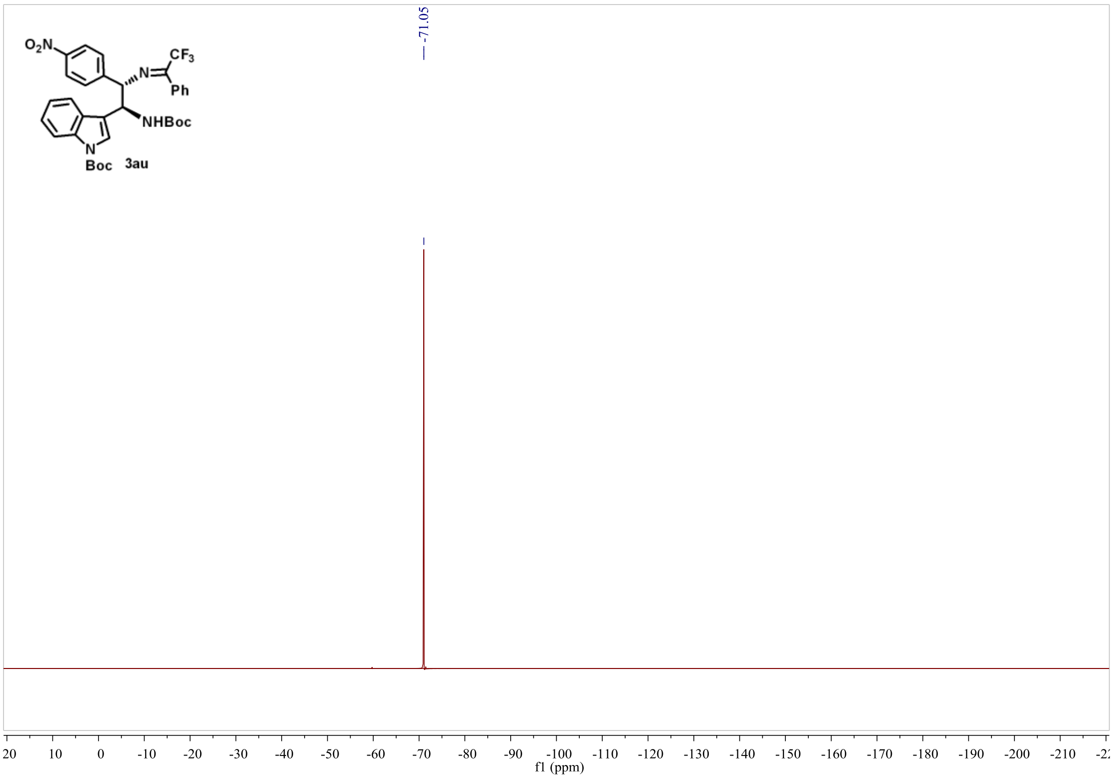

Supplementary Figure 123. ^{19}F NMR spectrum for compound **3au**

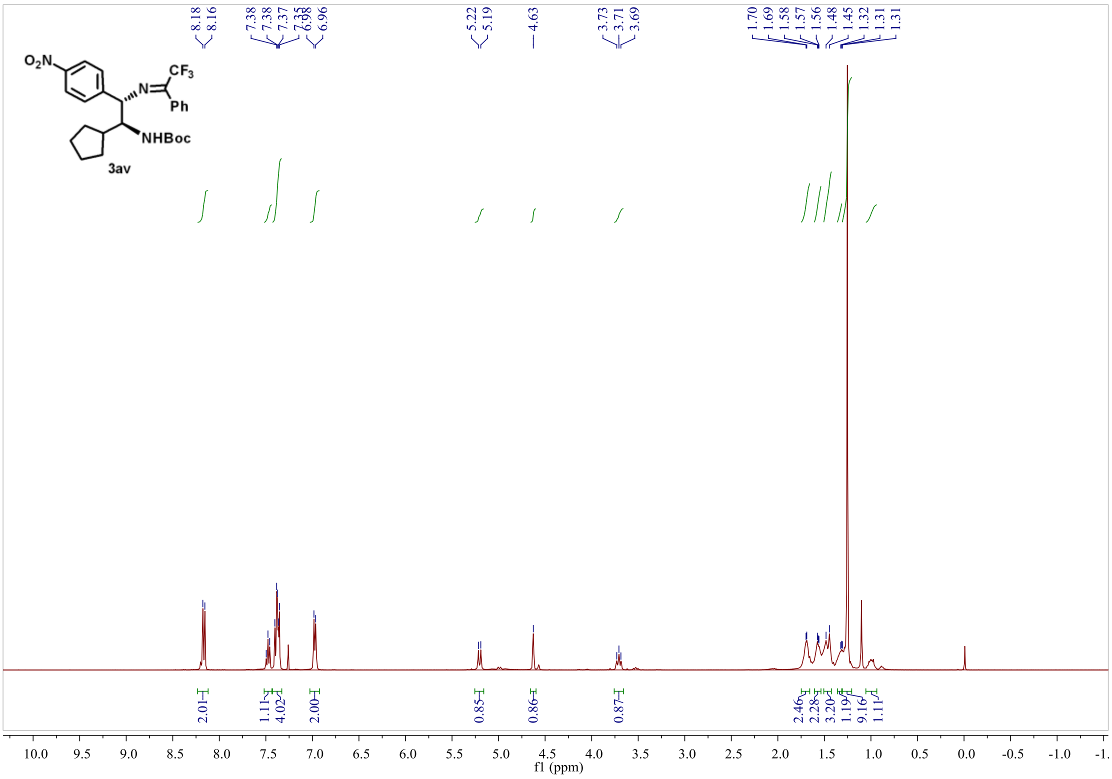

Supplementary Figure 124. ^{1}H NMR spectrum for compound **3av**

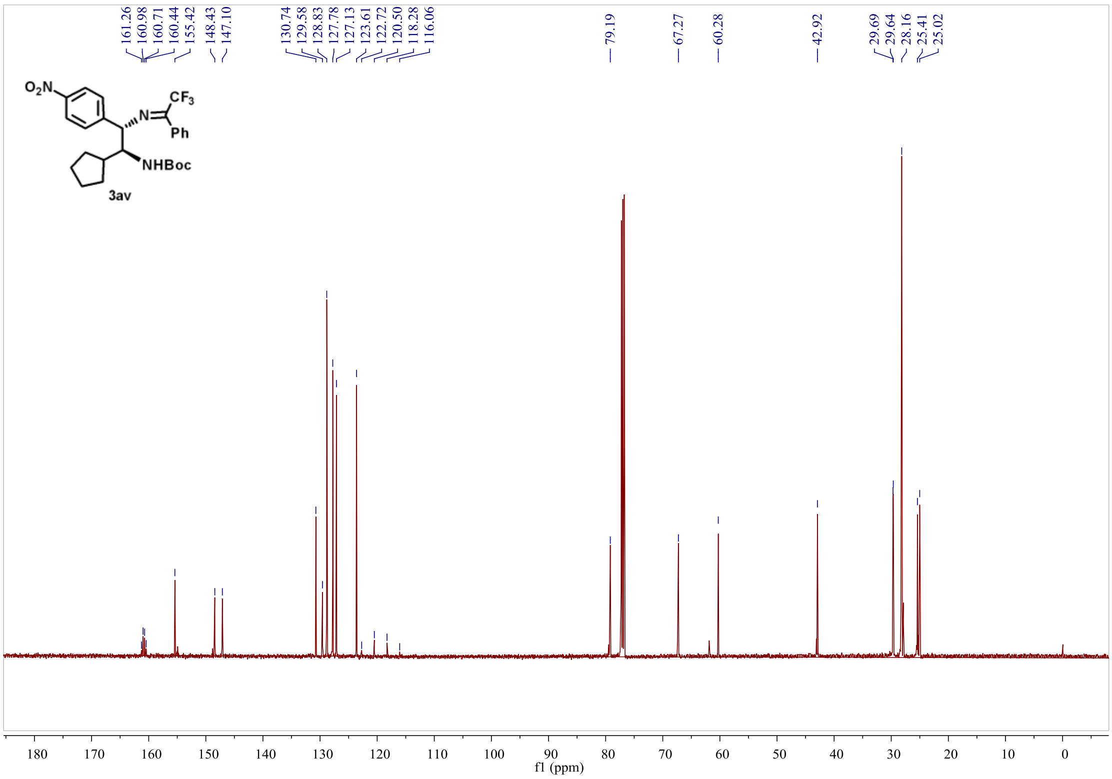

Supplementary Figure 125. ^{13}C NMR spectrum for compound **3av**

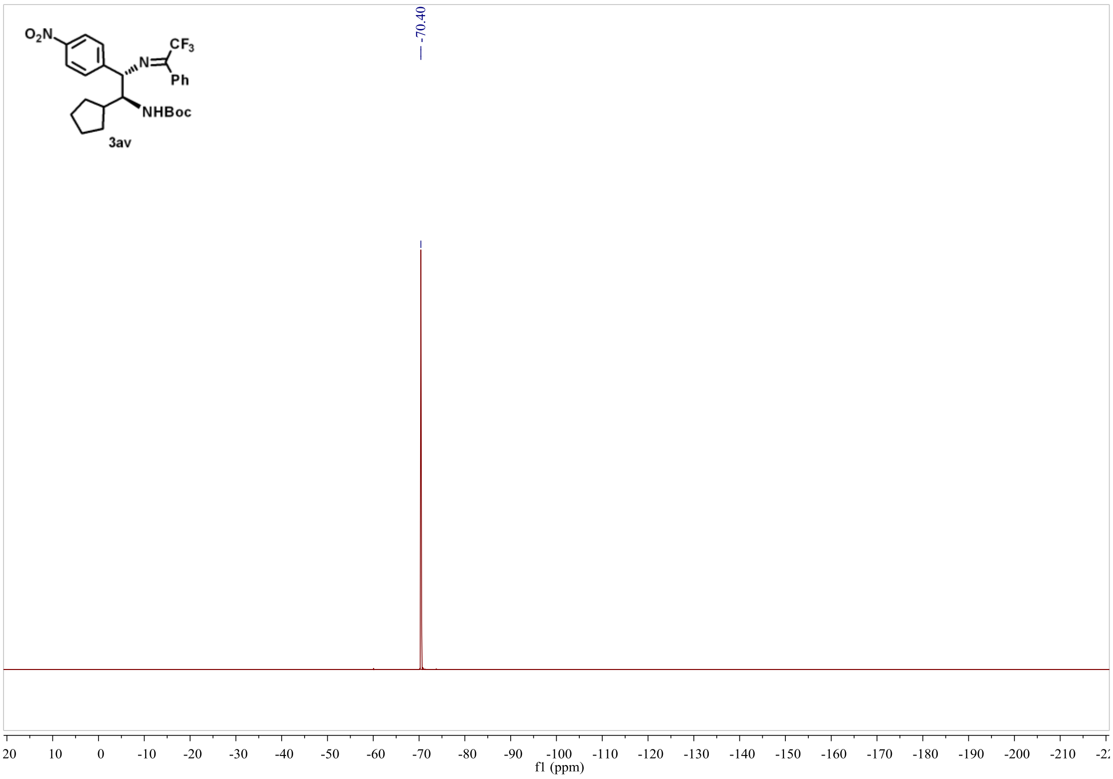

Supplementary Figure 126. ^{19}F NMR spectrum for compound **3av**

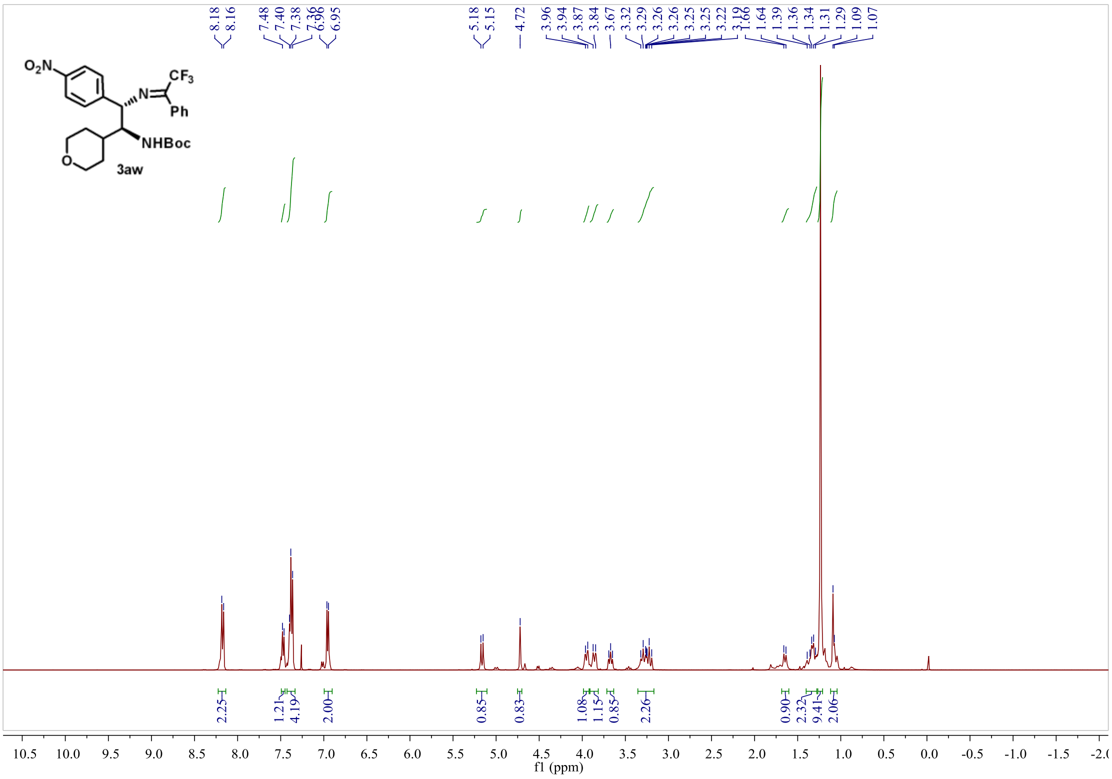

Supplementary Figure 127. ^{1}H NMR spectrum for compound **3aw**

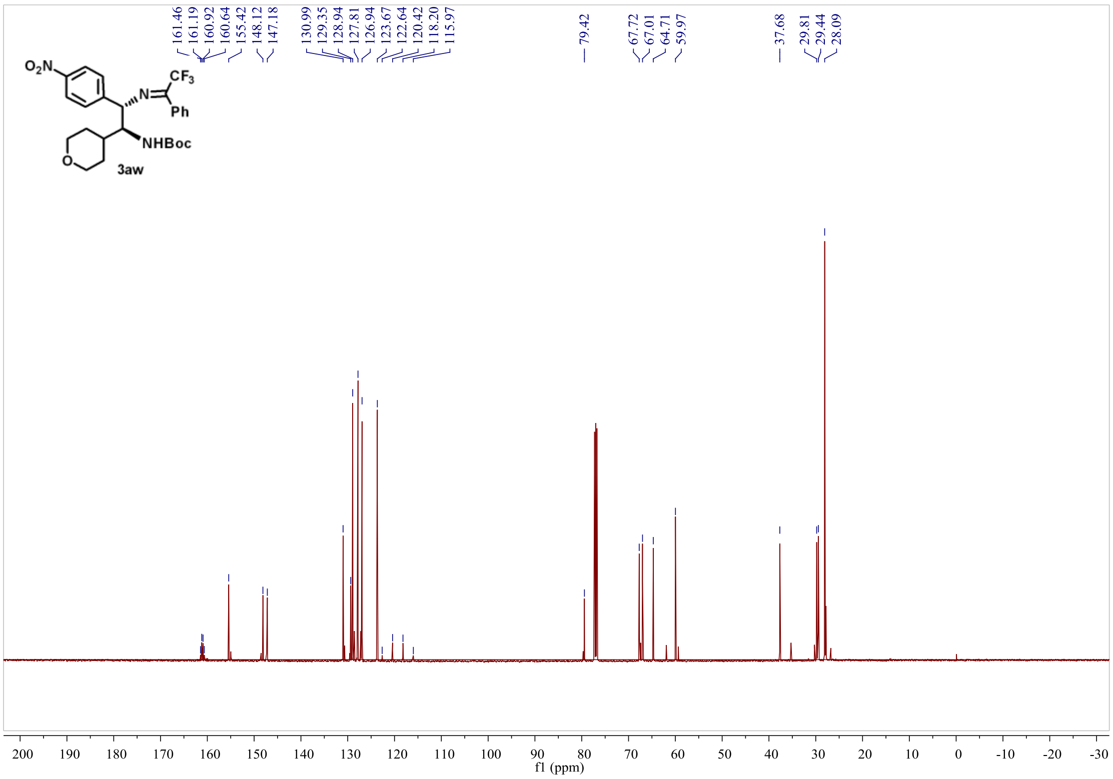

Supplementary Figure 128. ^{13}C NMR spectrum for compound **3aw**

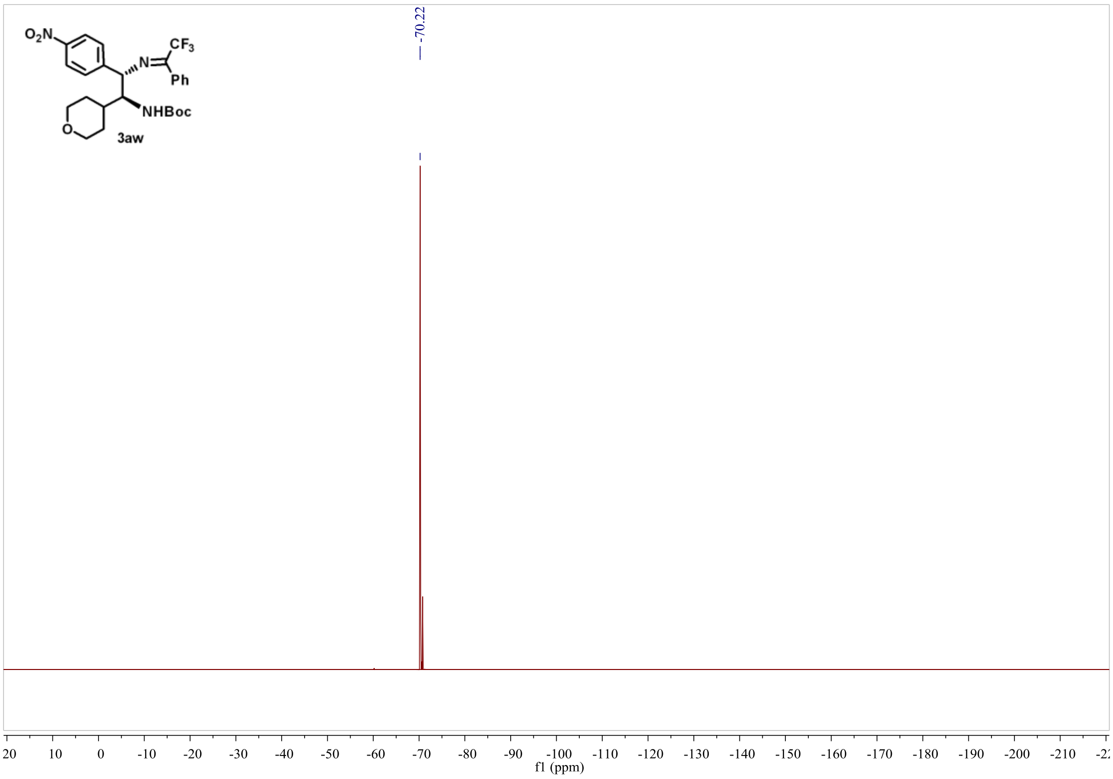

Supplementary Figure 129. ^{19}F NMR spectrum for compound **3aw**

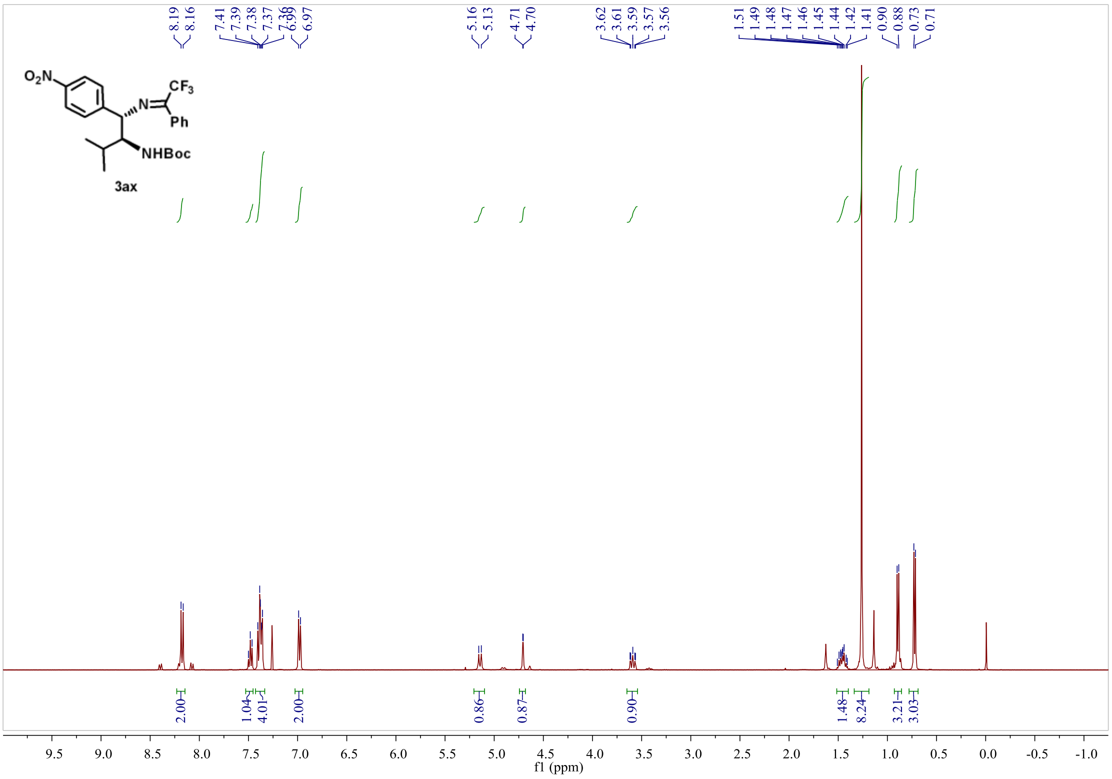

Supplementary Figure 130. ^{1}H NMR spectrum for compound **3ax**

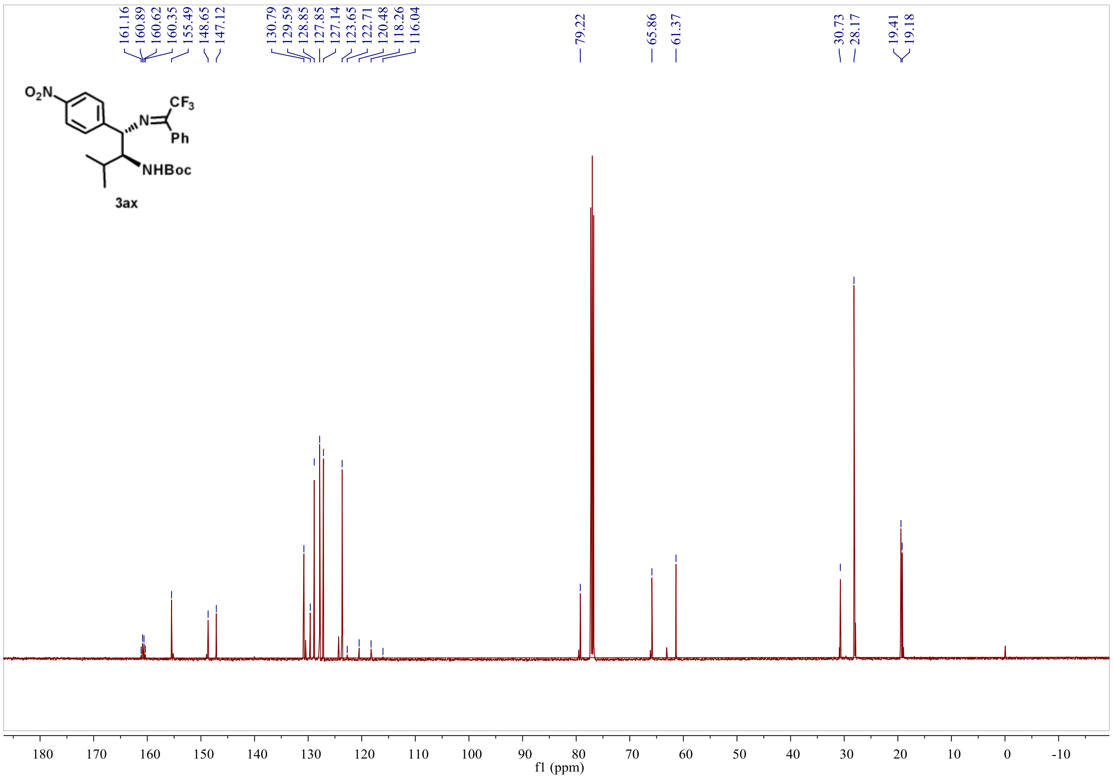

Supplementary Figure 131. ^{13}C NMR spectrum for compound **3ax**

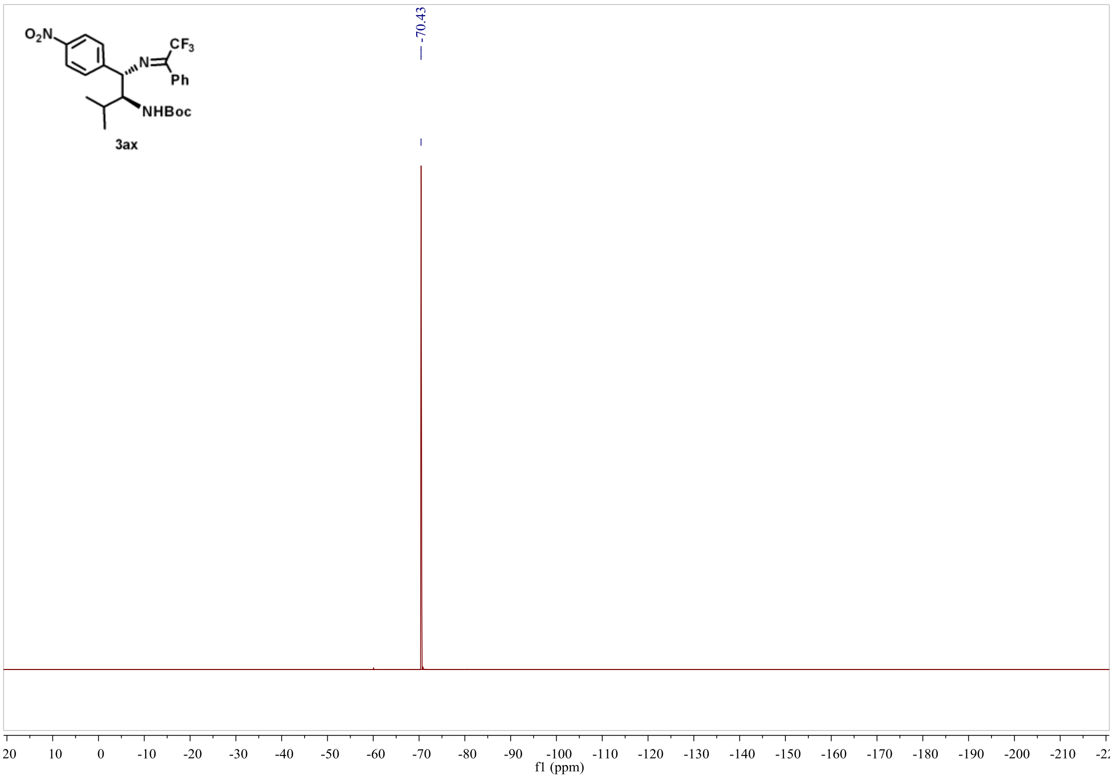

Supplementary Figure 132. ^{19}F NMR spectrum for compound **3ax**

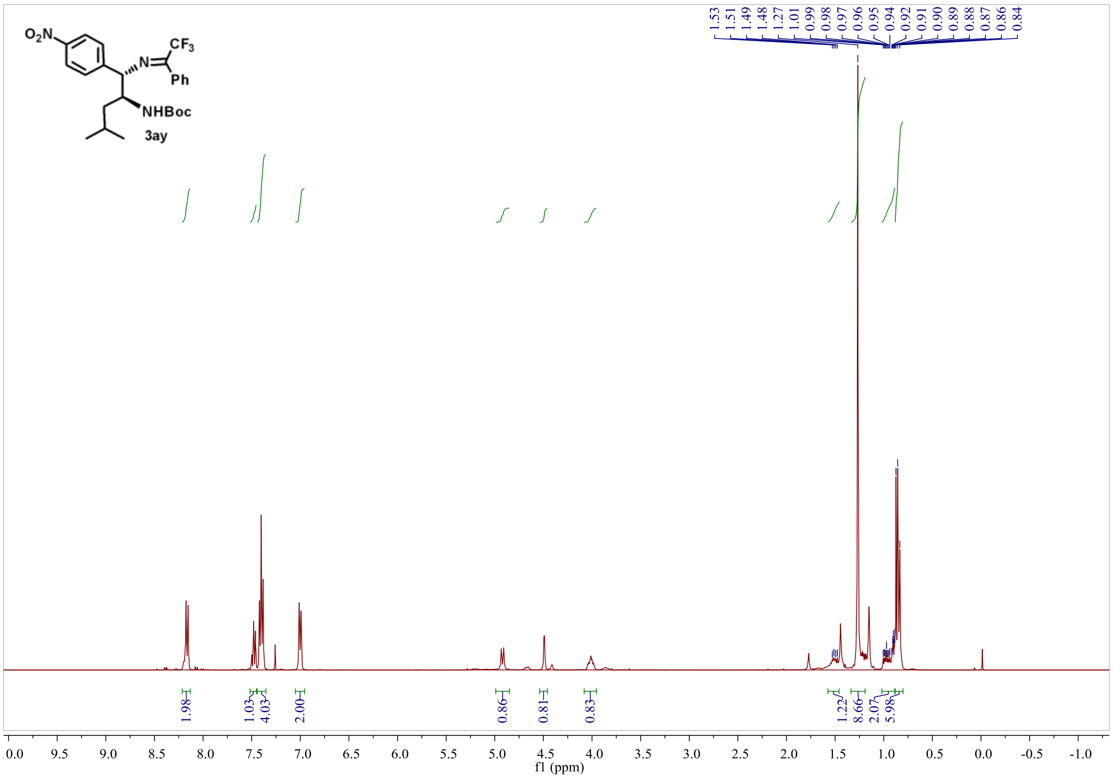

Supplementary Figure 133. ^{1}H NMR spectrum for compound **3ay**

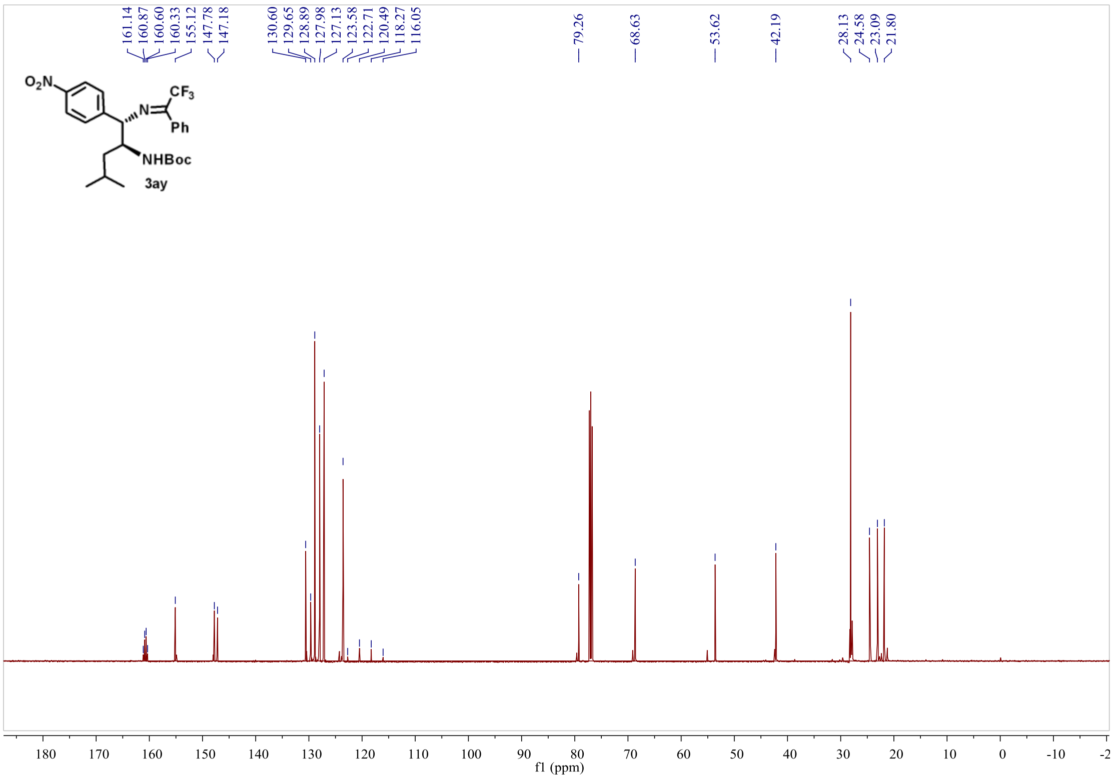

Supplementary Figure 134. ^{13}C NMR spectrum for compound **3ay**

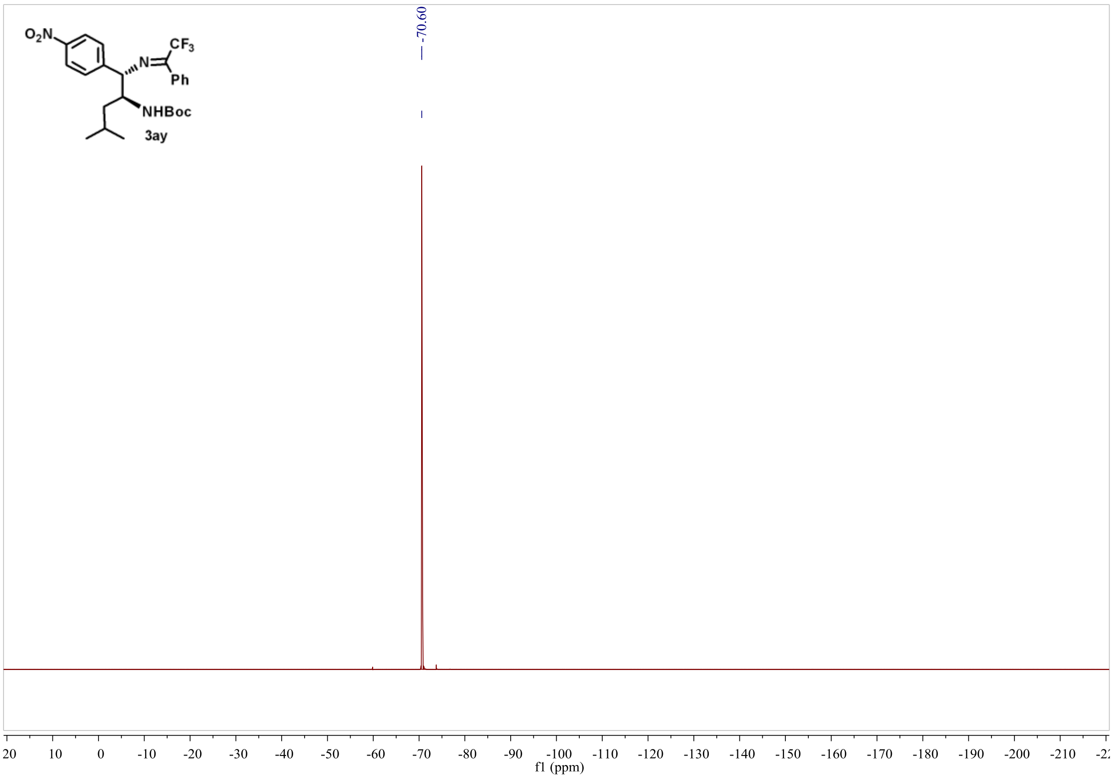

Supplementary Figure 135. ^{19}F NMR spectrum for compound **3ay**

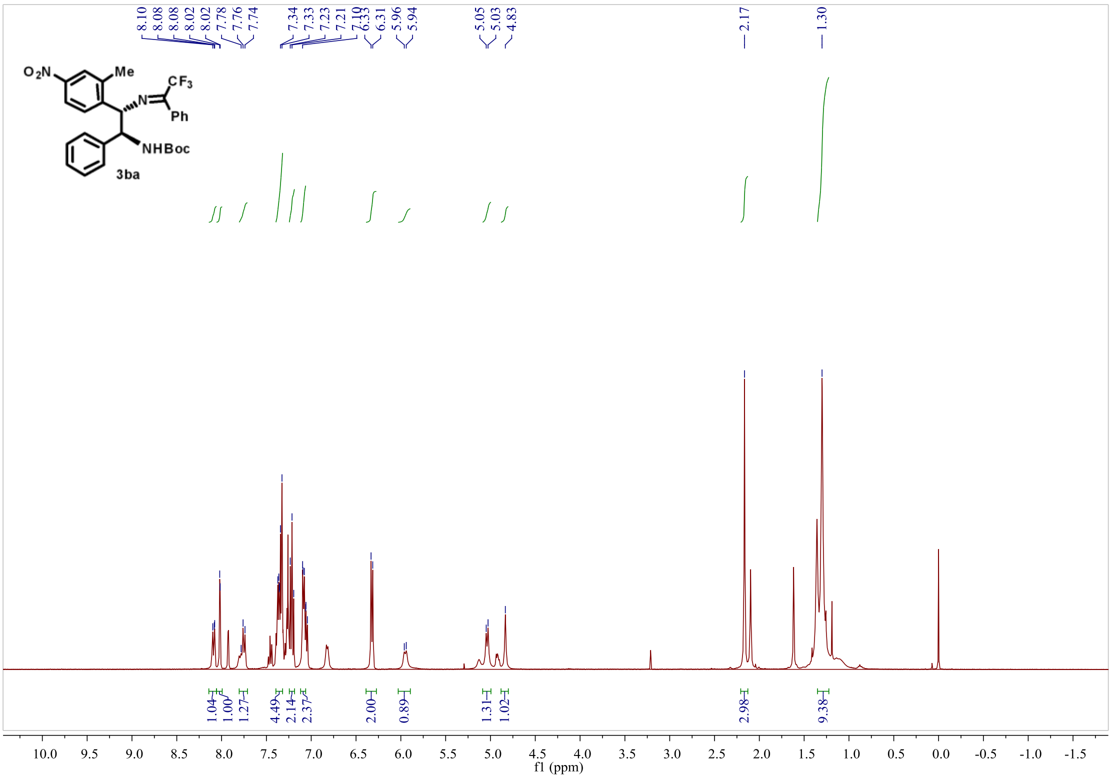

Supplementary Figure 136. ^{1}H NMR spectrum for compound **3ba**

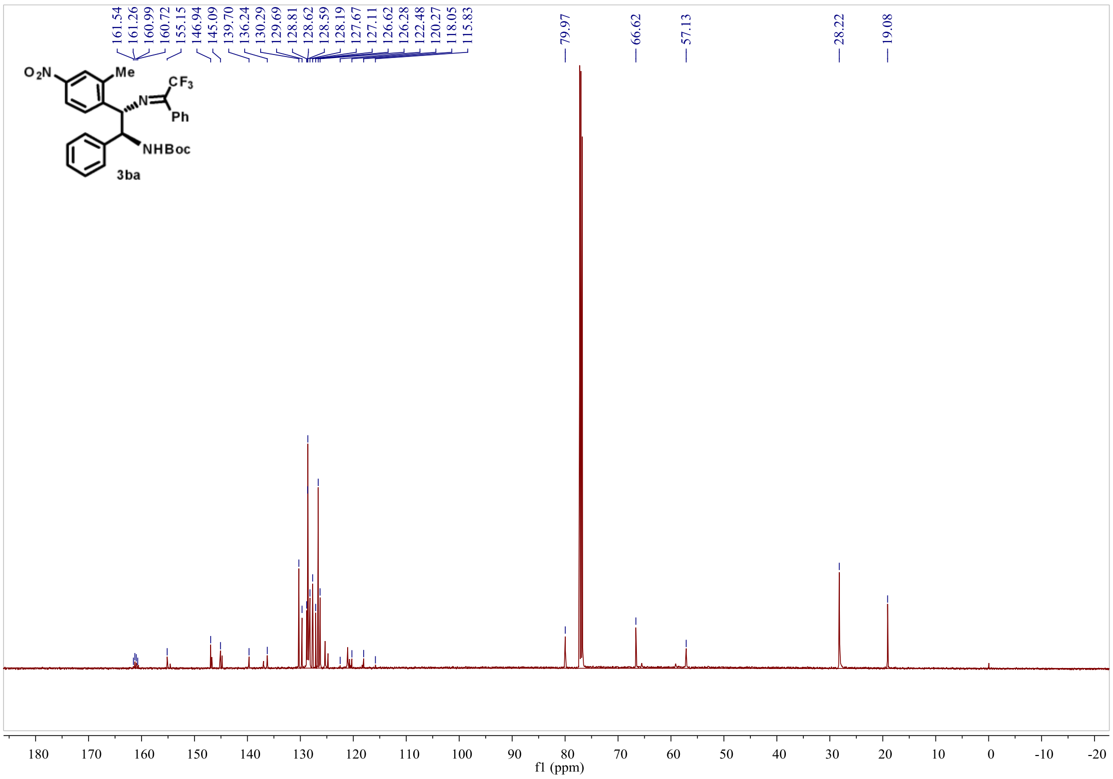

Supplementary Figure 137. ^{13}C NMR spectrum for compound **3ba**

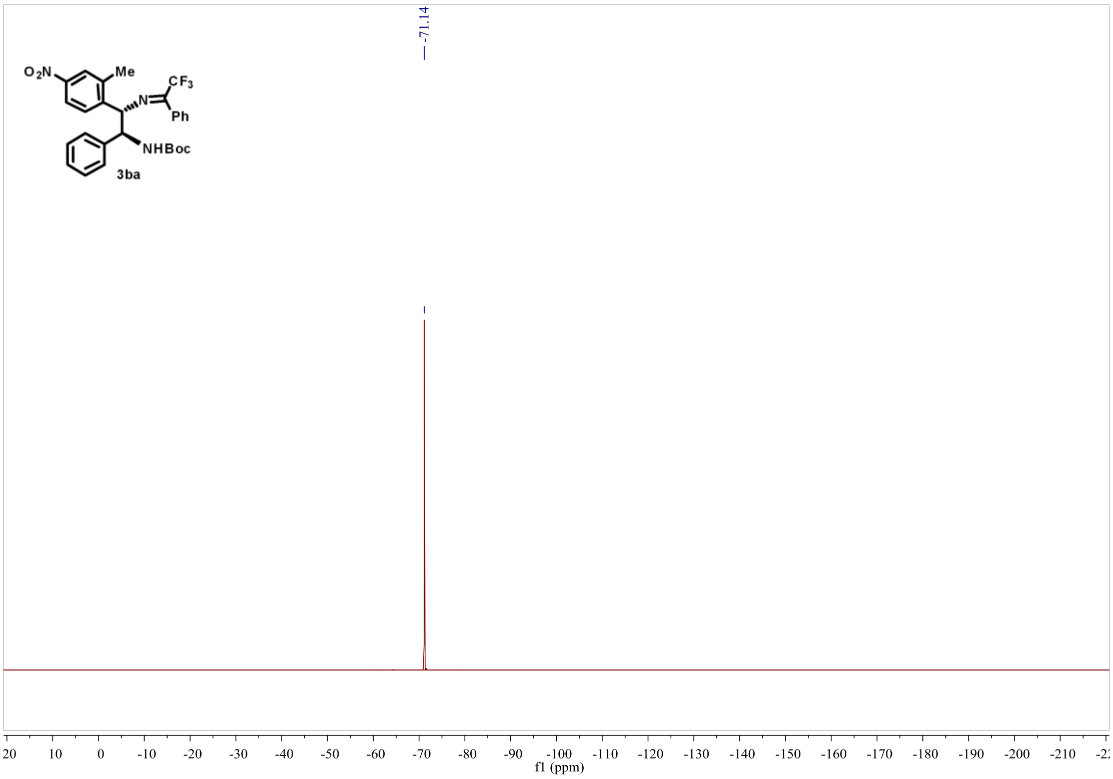

Supplementary Figure 138. ^{19}F NMR spectrum for compound **3ba**

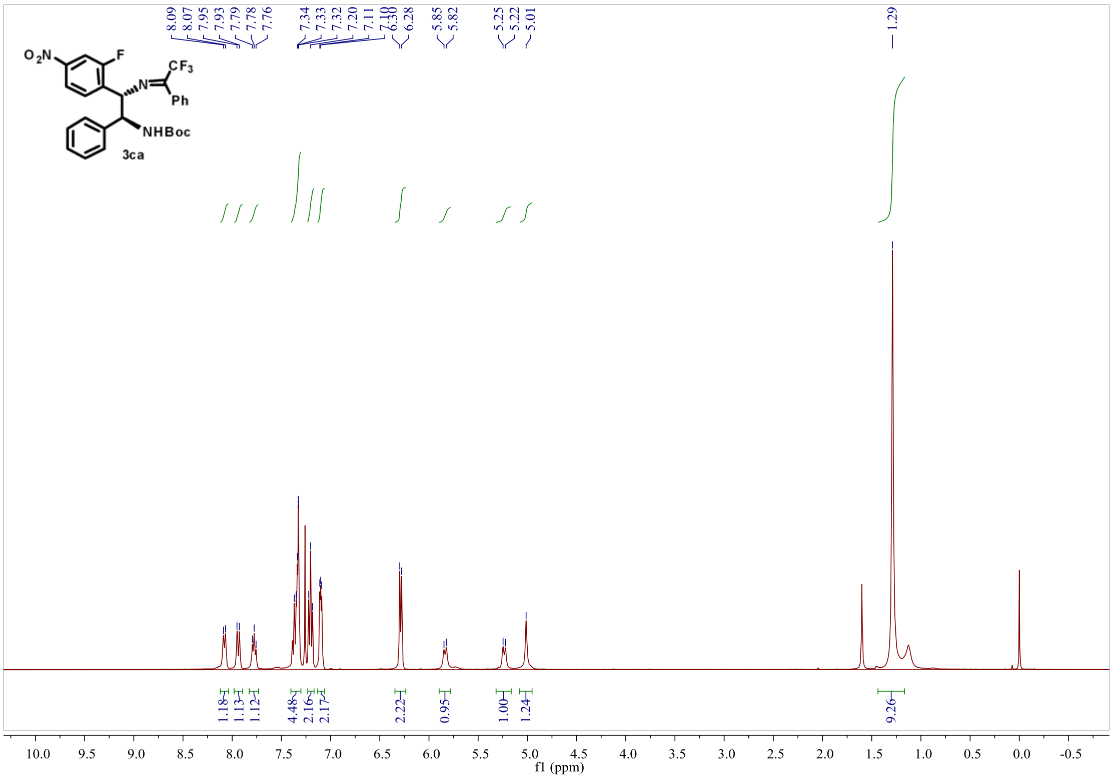

Supplementary Figure 139. ^{1}H NMR spectrum for compound **3ca**

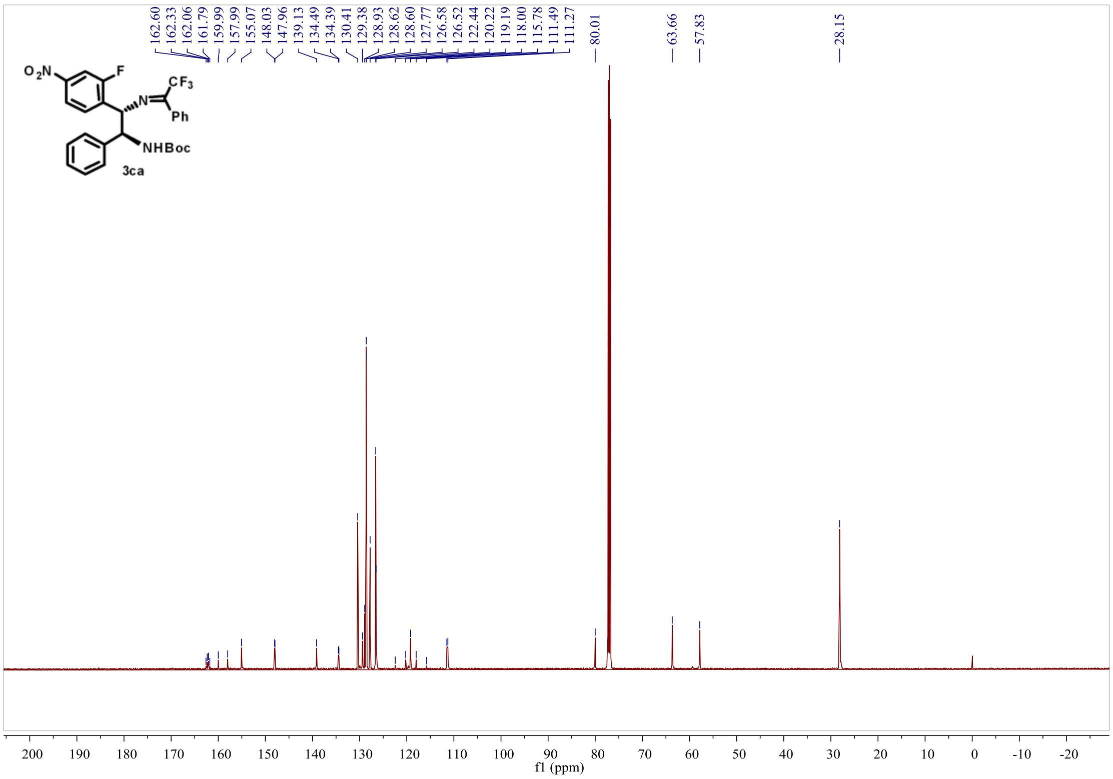

Supplementary Figure 140. ^{13}C NMR spectrum for compound **3ca**

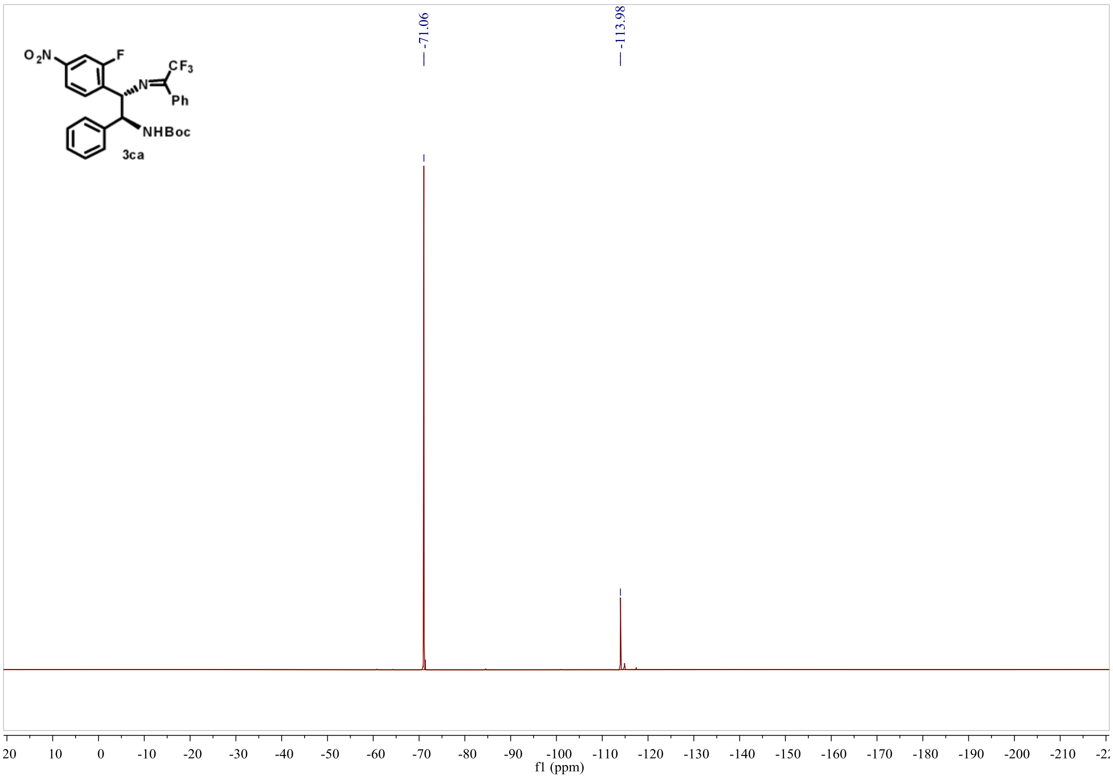

Supplementary Figure 141. ^{19}F NMR spectrum for compound **3ca**

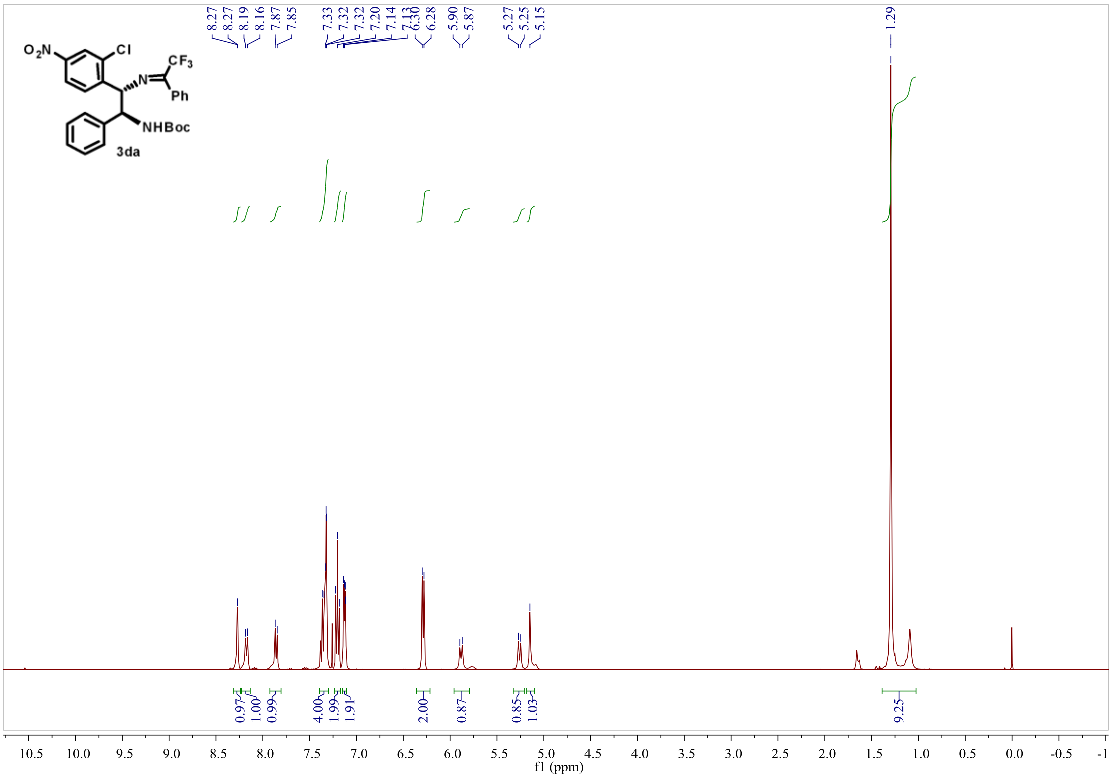

Supplementary Figure 142. ^{1}H NMR spectrum for compound **3da**

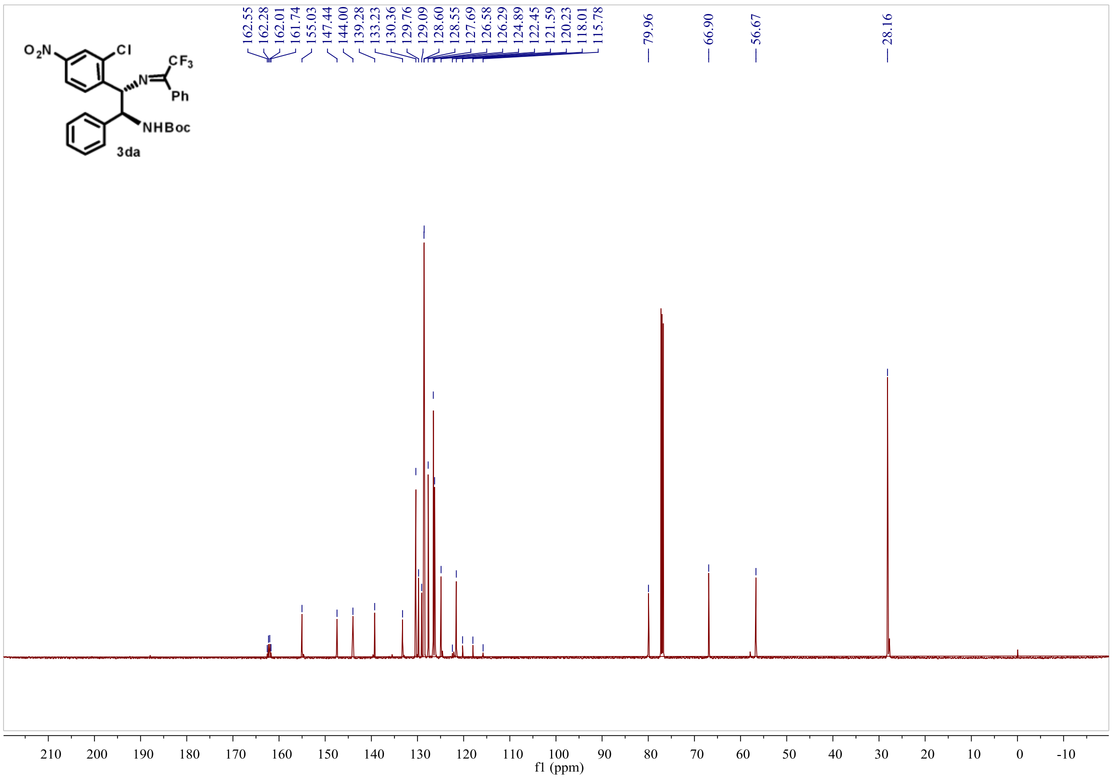

Supplementary Figure 143. ^{13}C NMR spectrum for compound **3da**

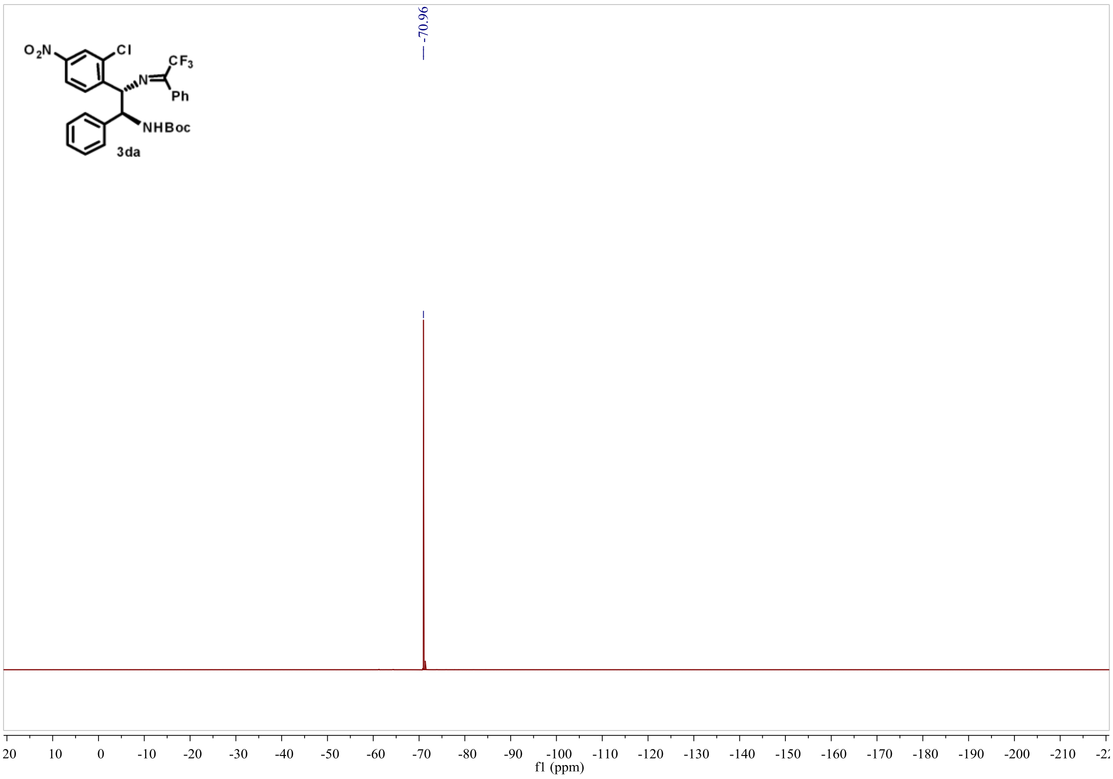

Supplementary Figure 144. ^{19}F NMR spectrum for compound **3da**

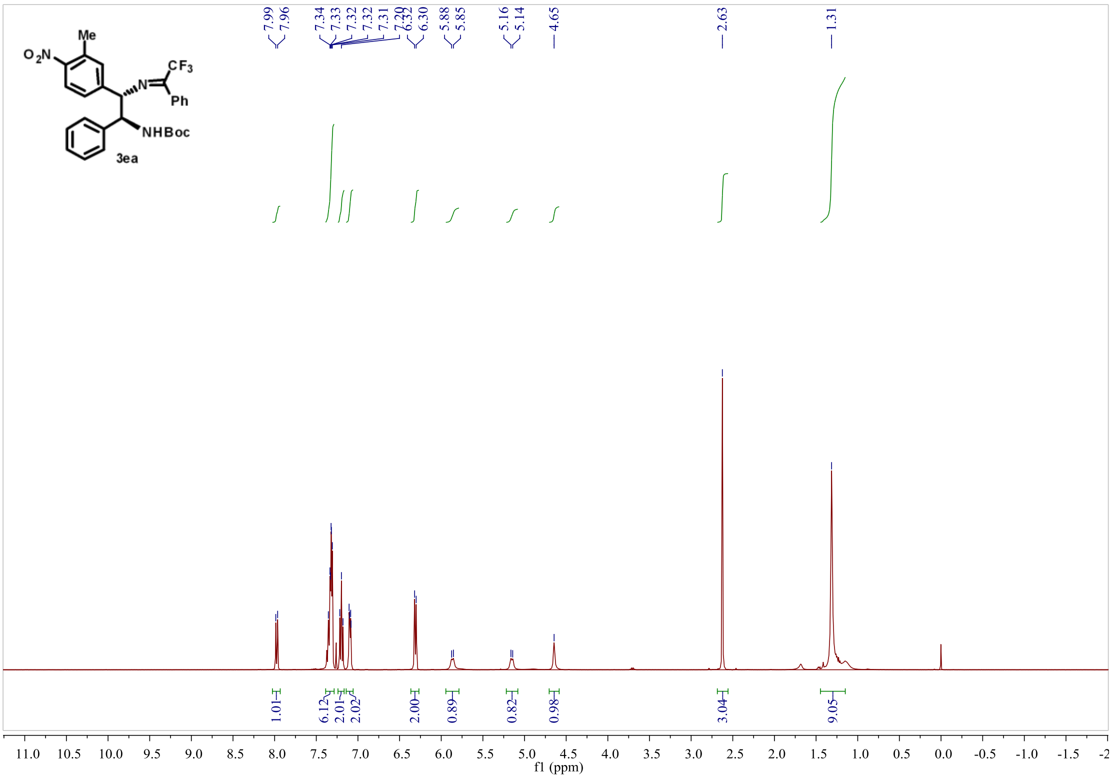

Supplementary Figure 145. ^{1}H NMR spectrum for compound **3ea**

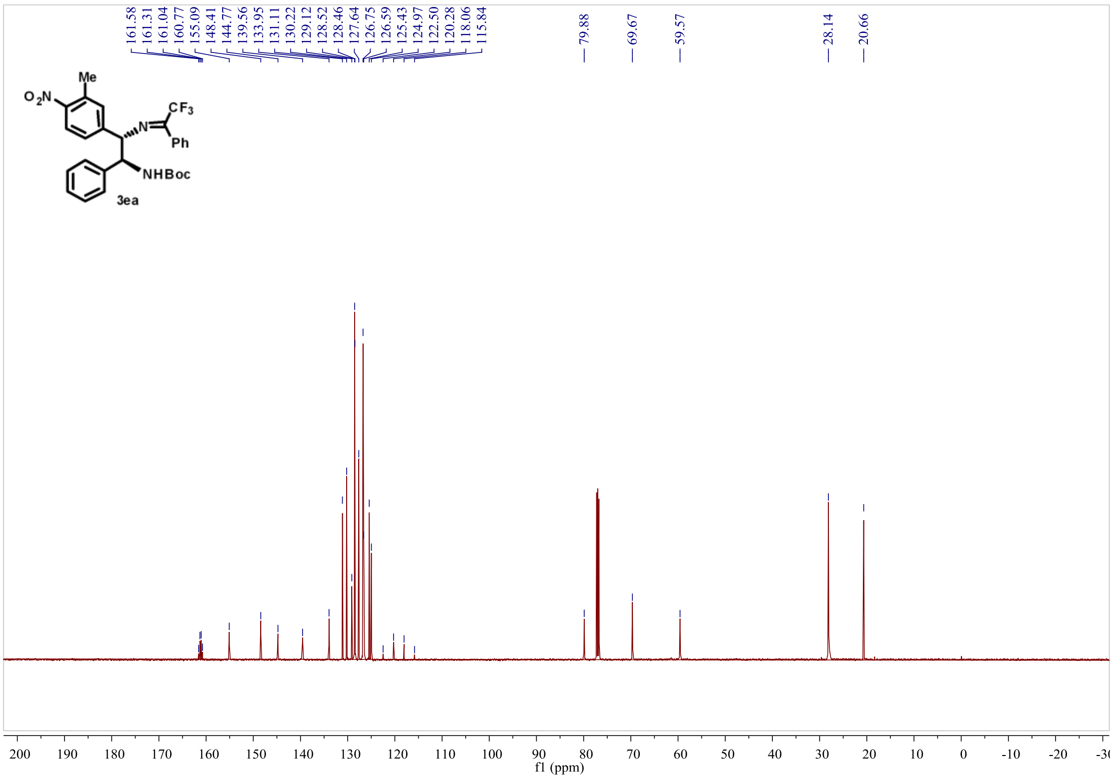

Supplementary Figure 146. ^{13}C NMR spectrum for compound **3ea**

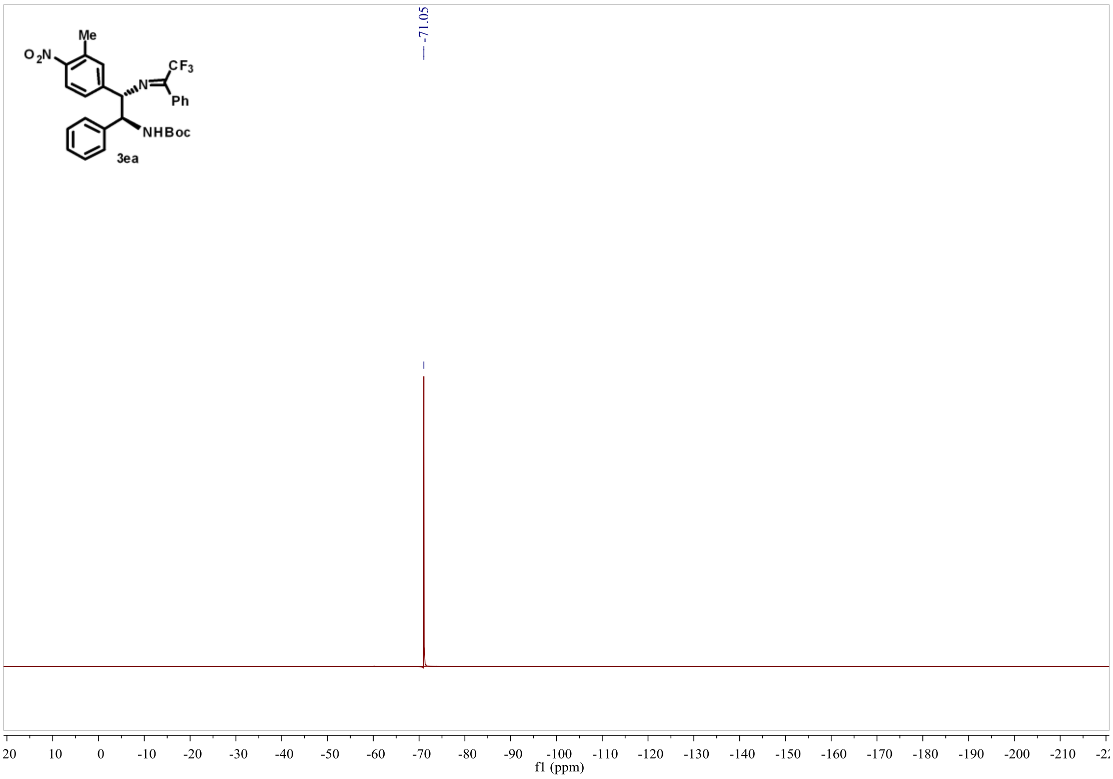

Supplementary Figure 147. ^{19}F NMR spectrum for compound **3ea**

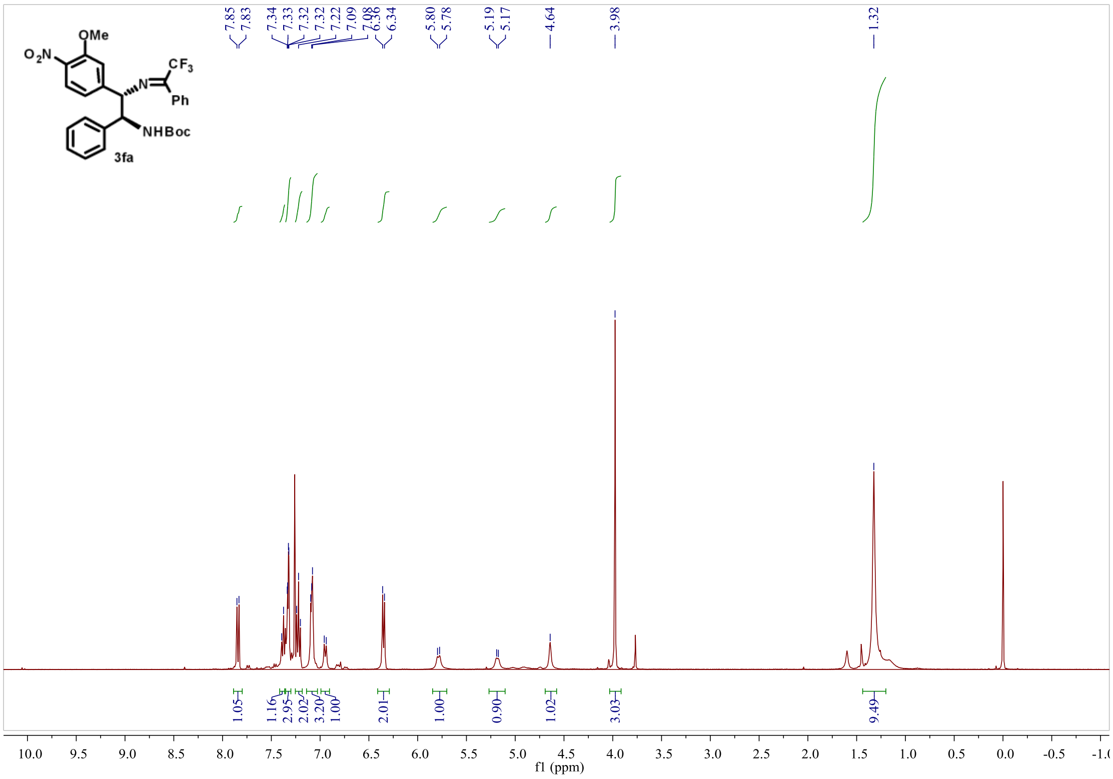

Supplementary Figure 148. ^{1}H NMR spectrum for compound **3fa**

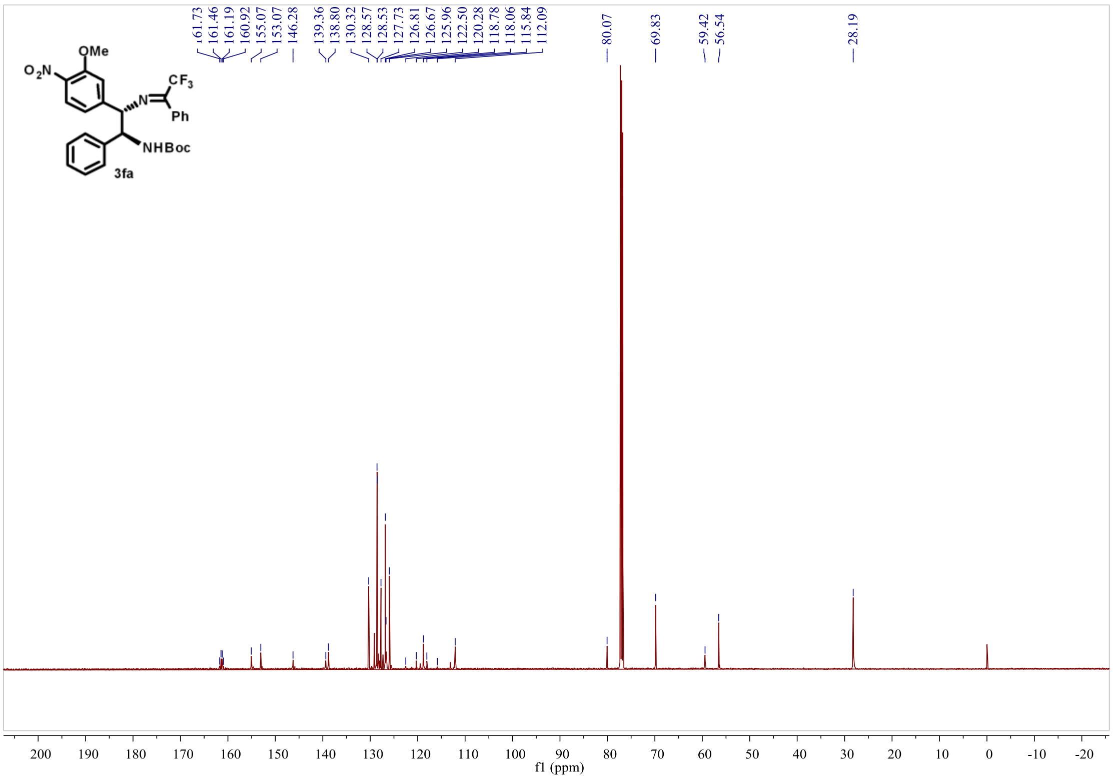

Supplementary Figure 149. ^{13}C NMR spectrum for compound **3fa**

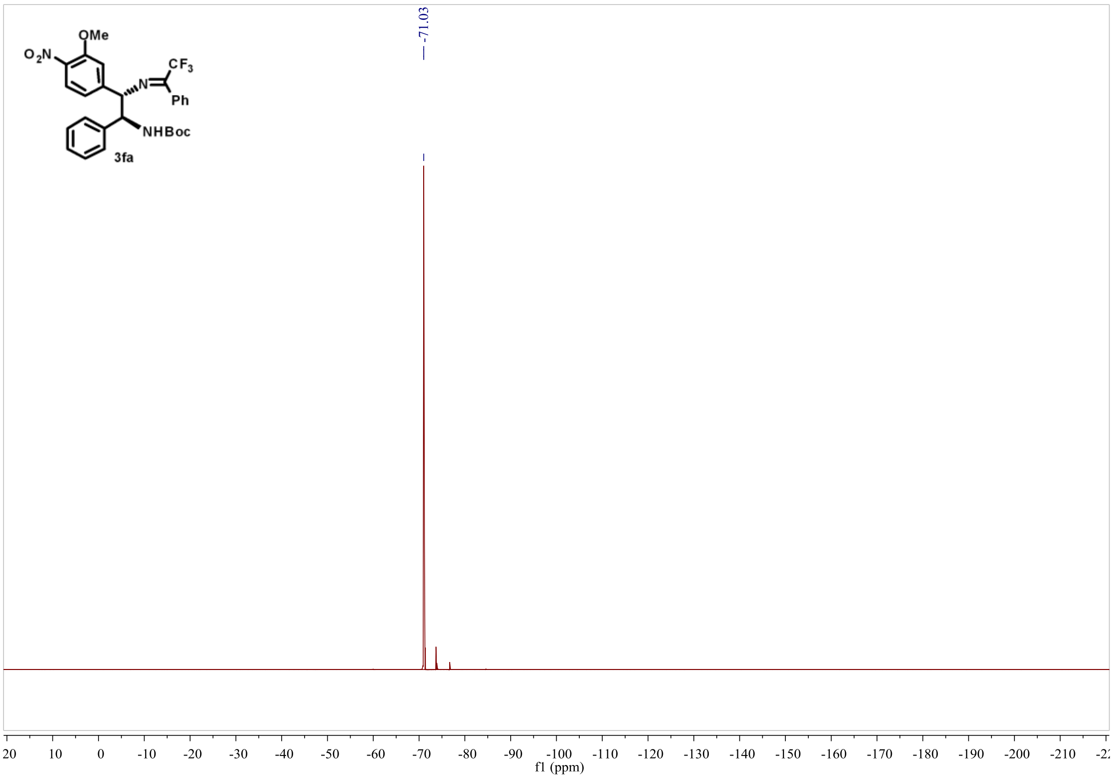

Supplementary Figure 150. ^{19}F NMR spectrum for compound **3fa**

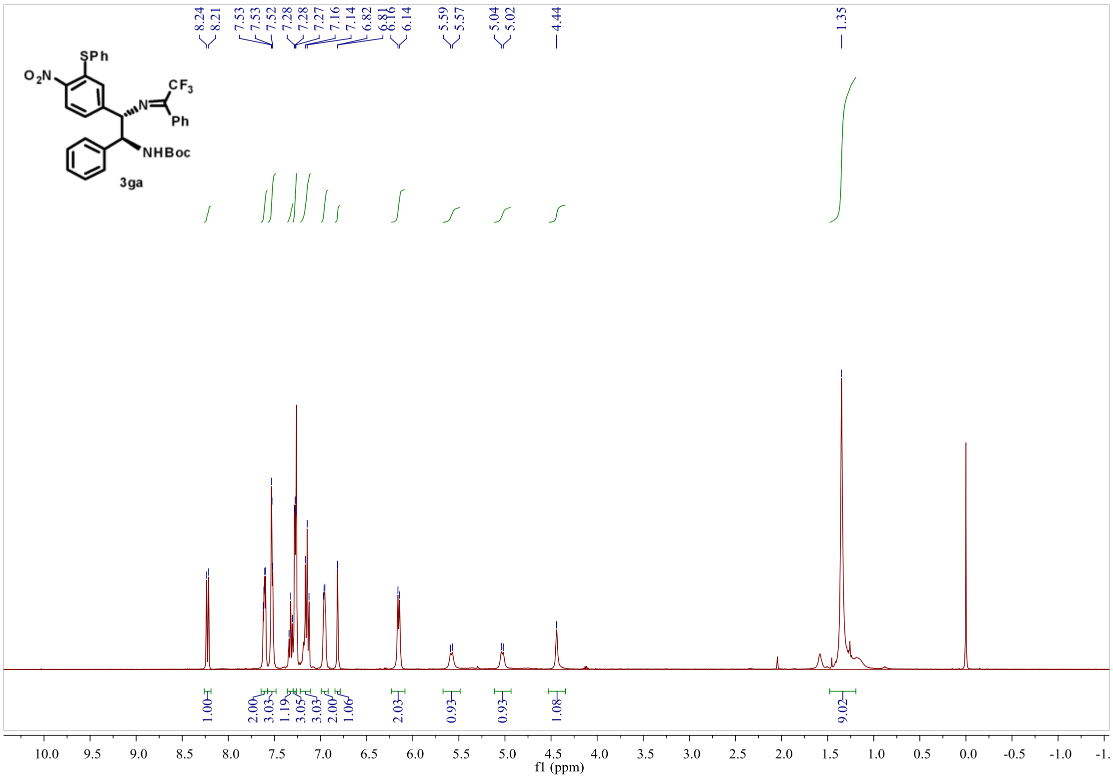

Supplementary Figure 151. ^{1}H NMR spectrum for compound **3ga**

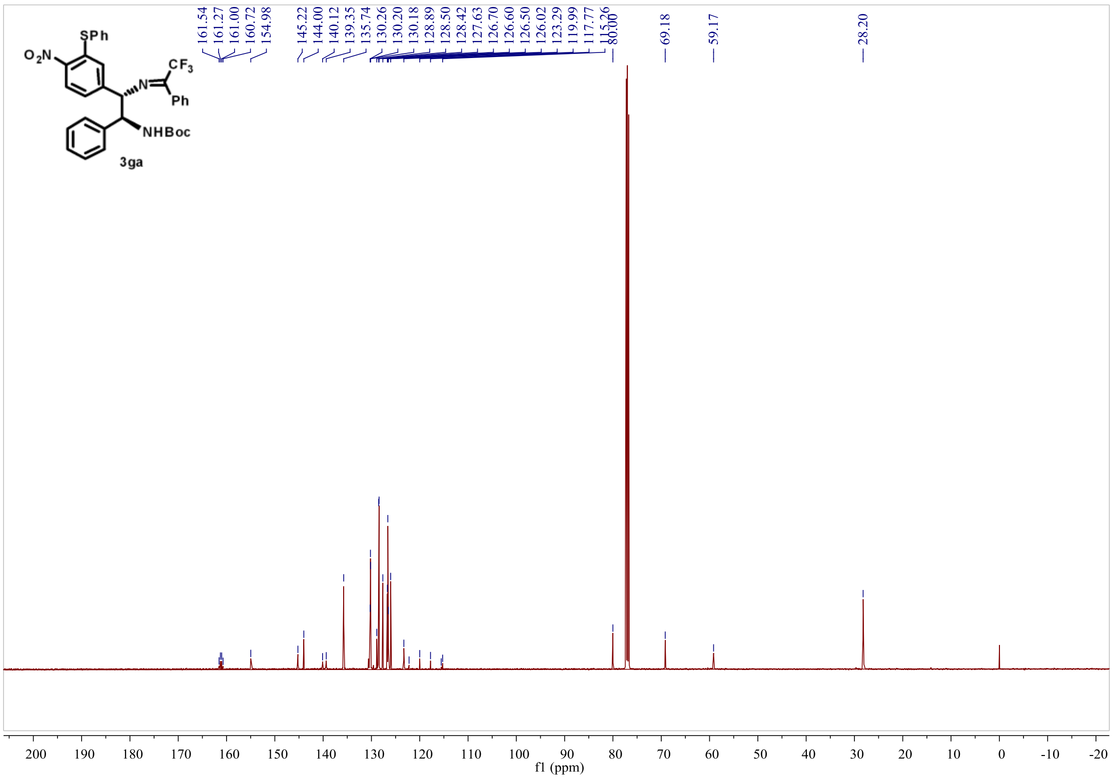

Supplementary Figure 152. ^{13}C NMR spectrum for compound **3ga**

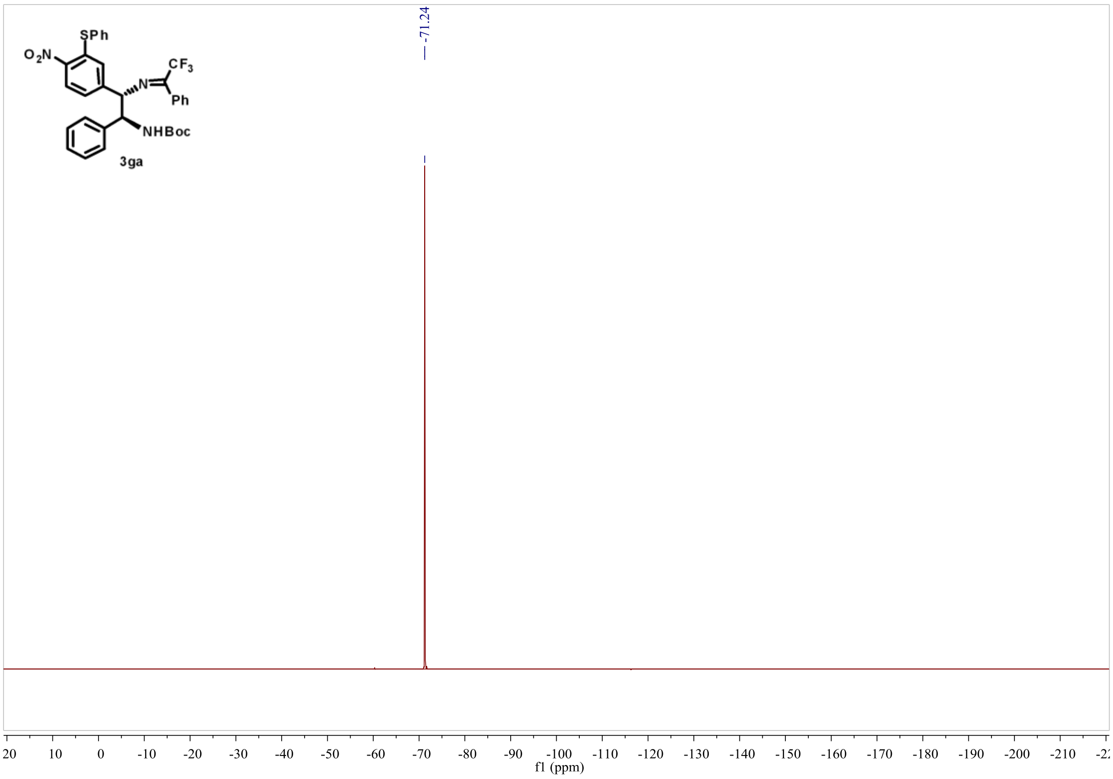

Supplementary Figure 153. ^{19}F NMR spectrum for compound **3ga**

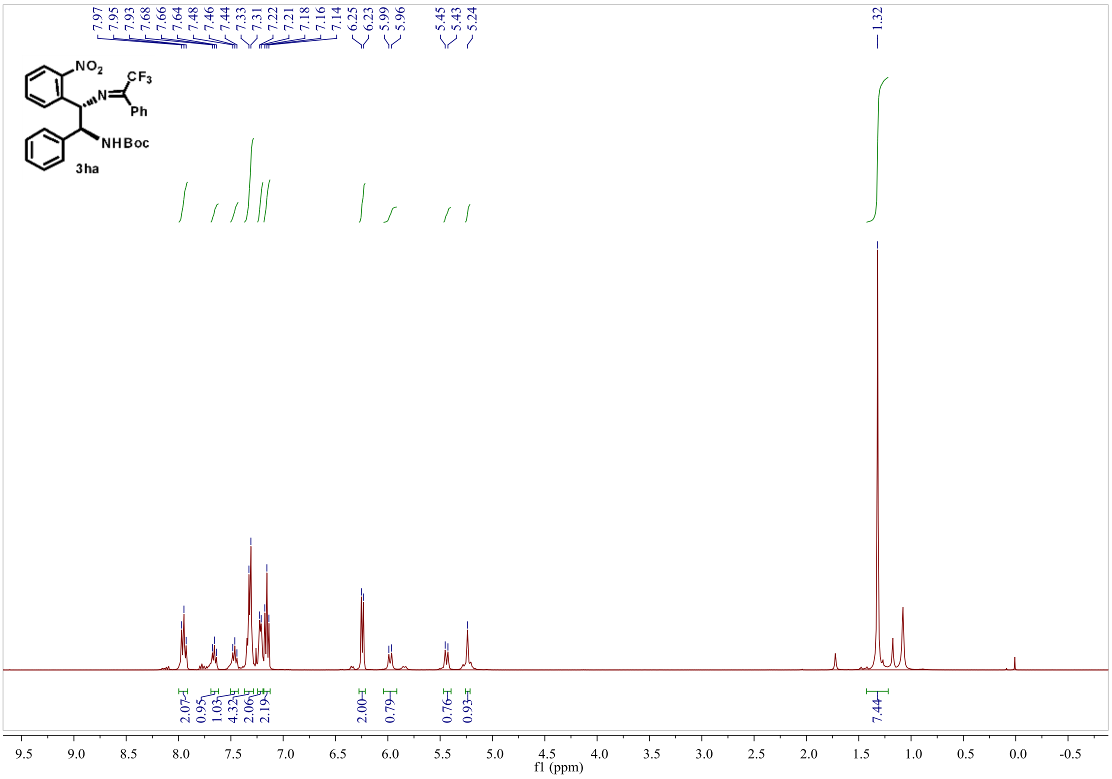

Supplementary Figure 154. ^{1}H NMR spectrum for compound **3ha**

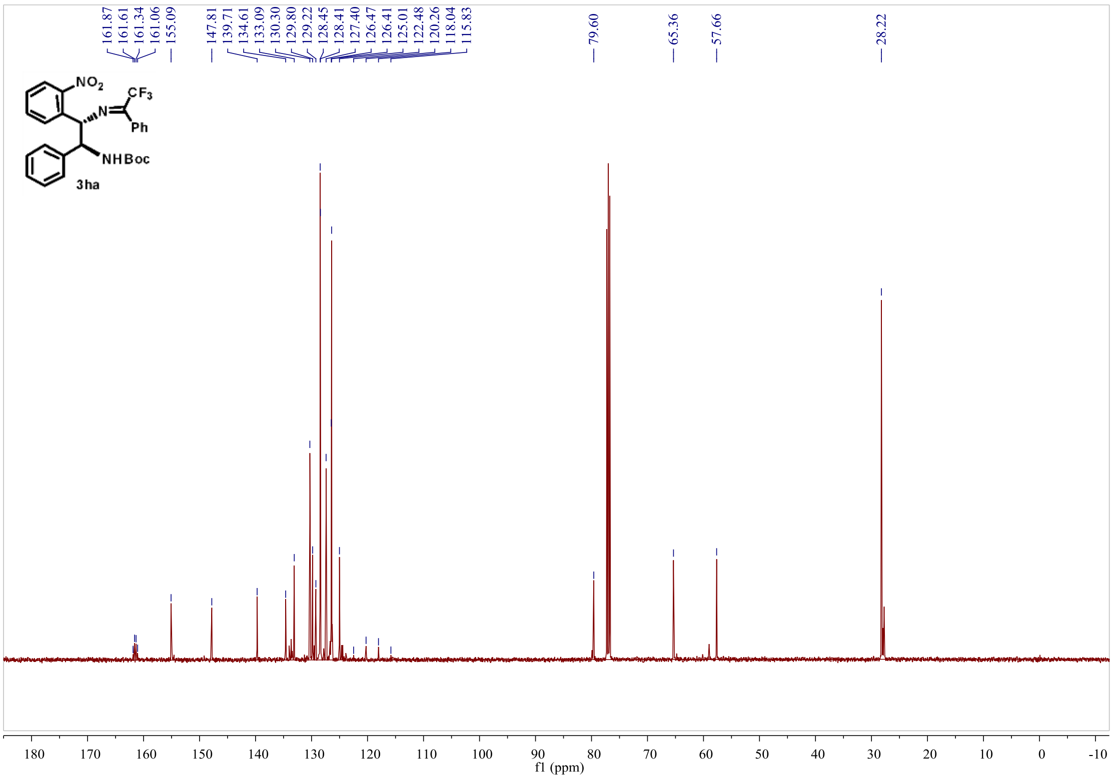

Supplementary Figure 155. ^{13}C NMR spectrum for compound **3ha**

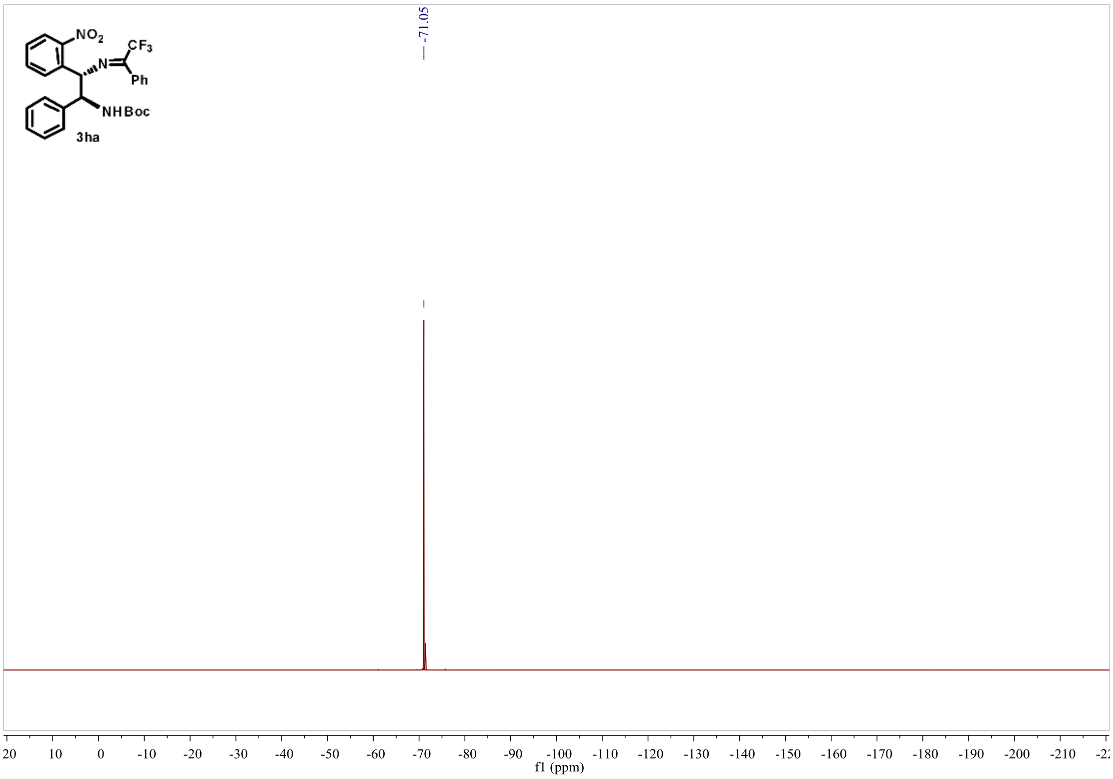

Supplementary Figure 156. ^{19}F NMR spectrum for compound **3ha**

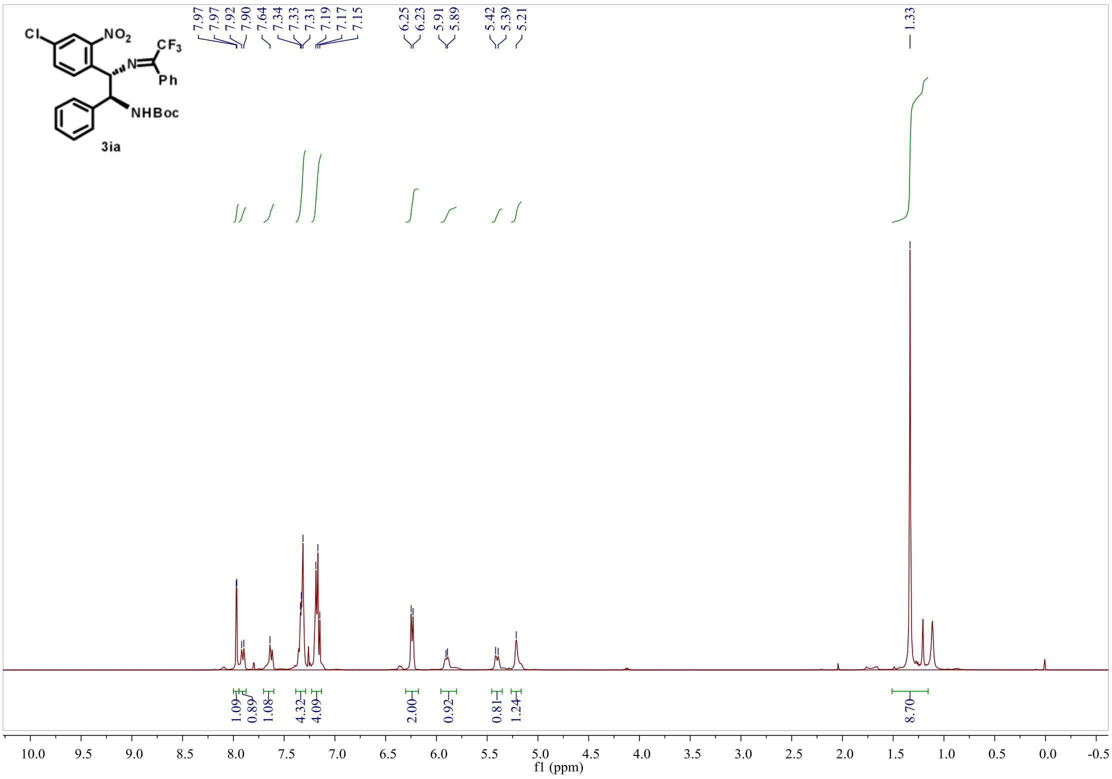

Supplementary Figure 157. ^{1}H NMR spectrum for compound **3ia**

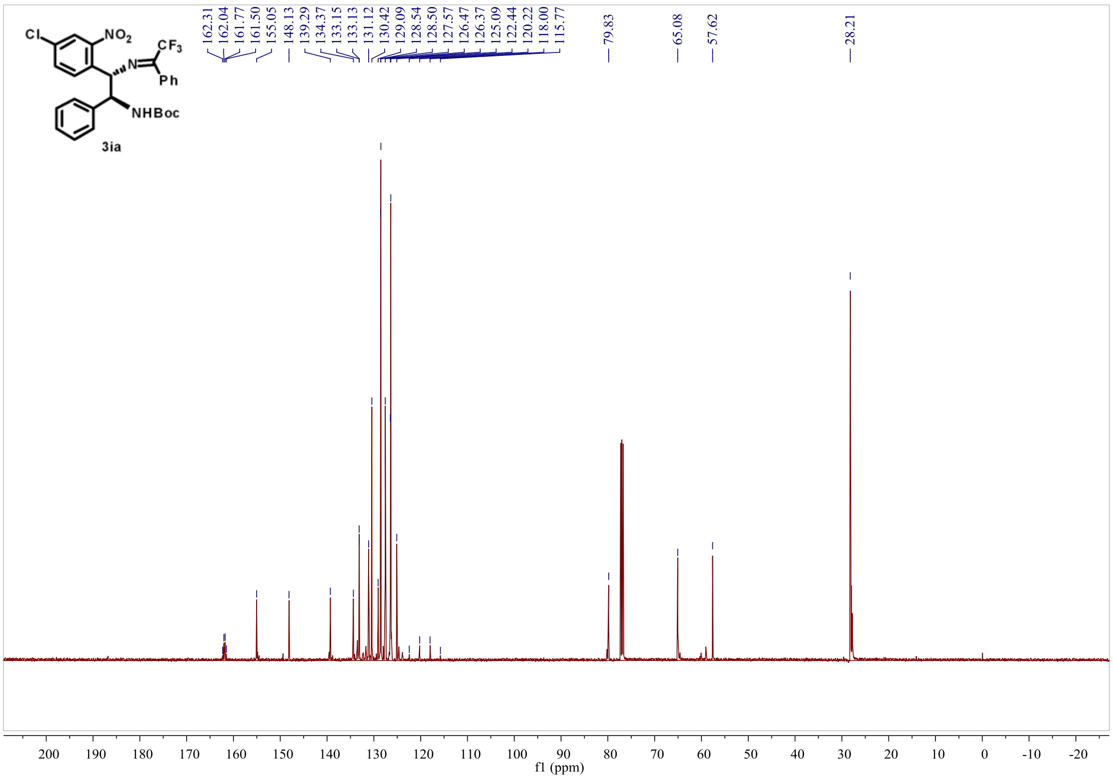

Supplementary Figure 158. ^{13}C NMR spectrum for compound **3ia**

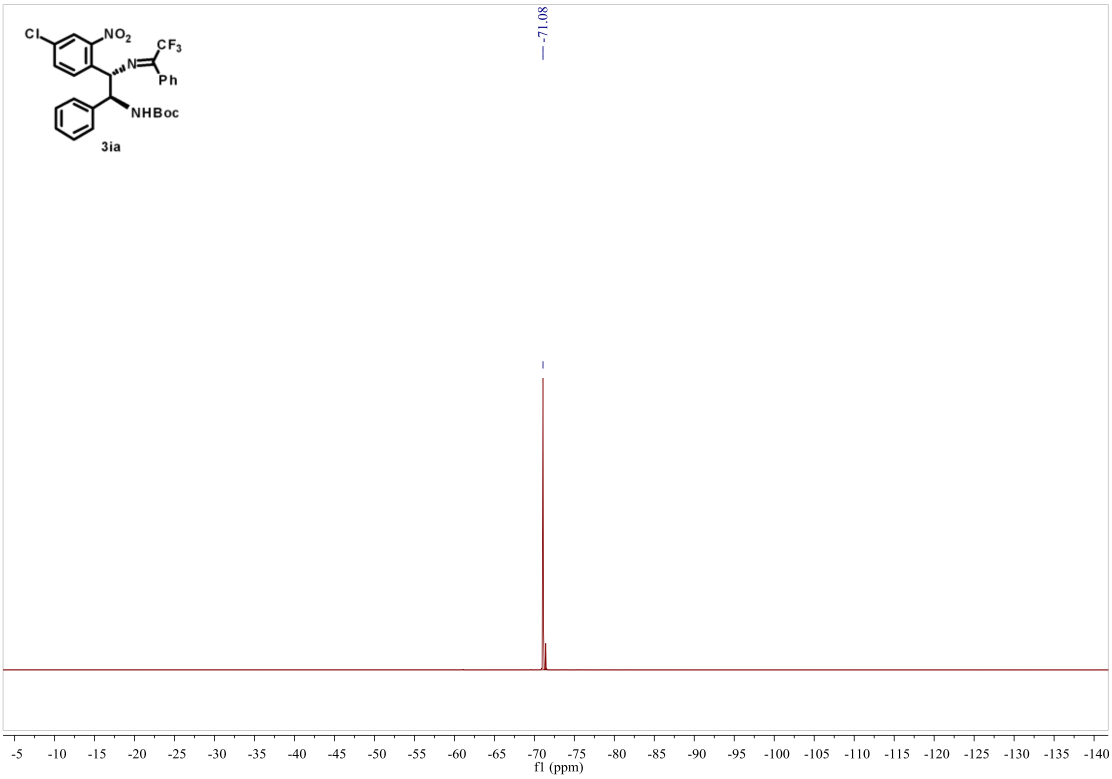

Supplementary Figure 159. ^{19}F NMR spectrum for compound **3ia**

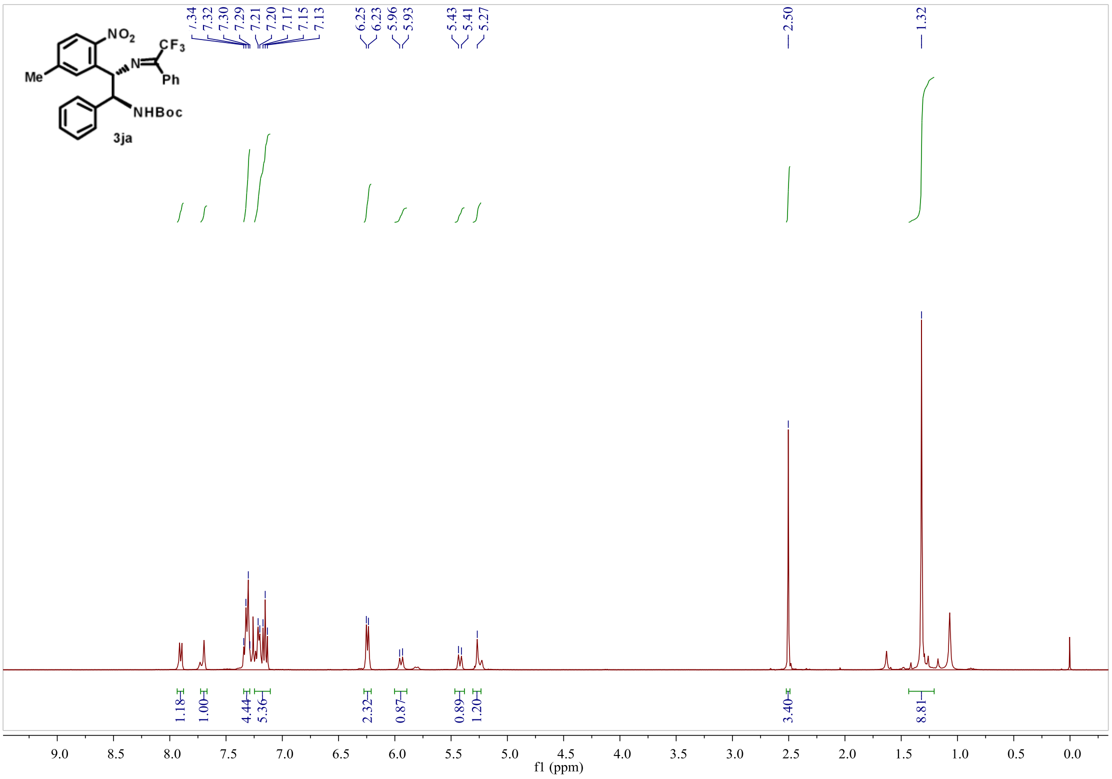

Supplementary Figure 160. ^{1}H NMR spectrum for compound **3ja**

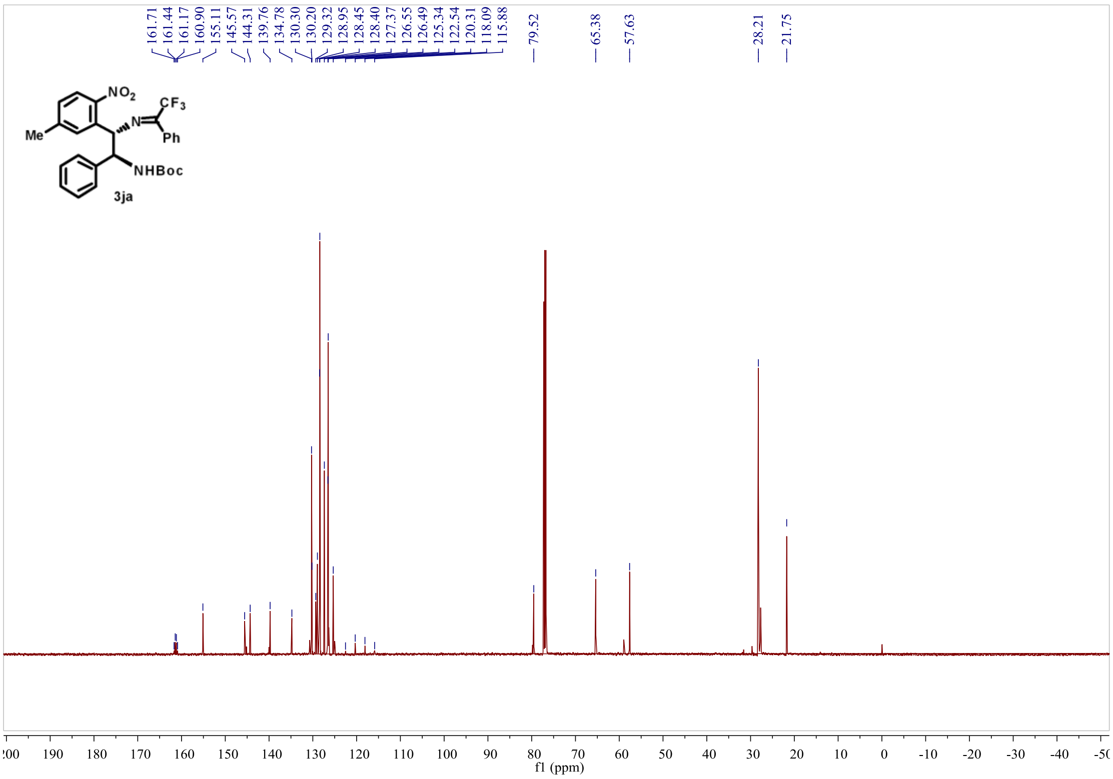

Supplementary Figure 161. ^{13}C NMR spectrum for compound **3ja**

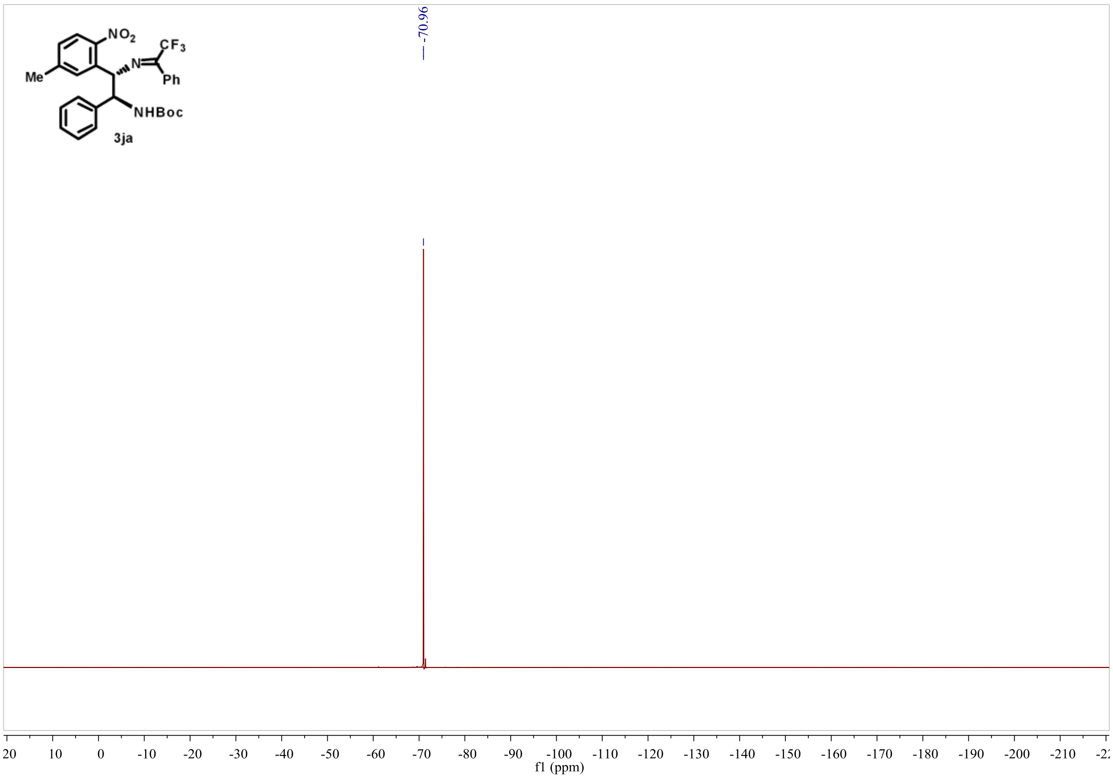

Supplementary Figure 162. ^{19}F NMR spectrum for compound **3ja**

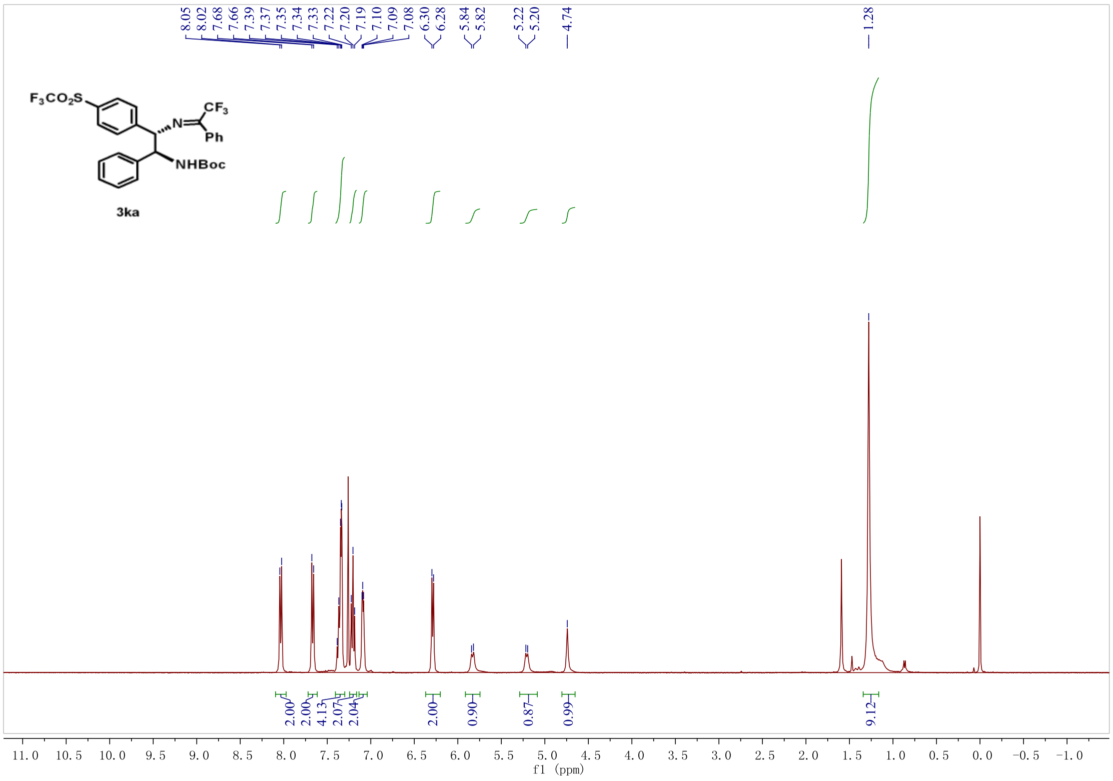

Supplementary Figure 163. ^{1}H NMR spectrum for compound **3ka**

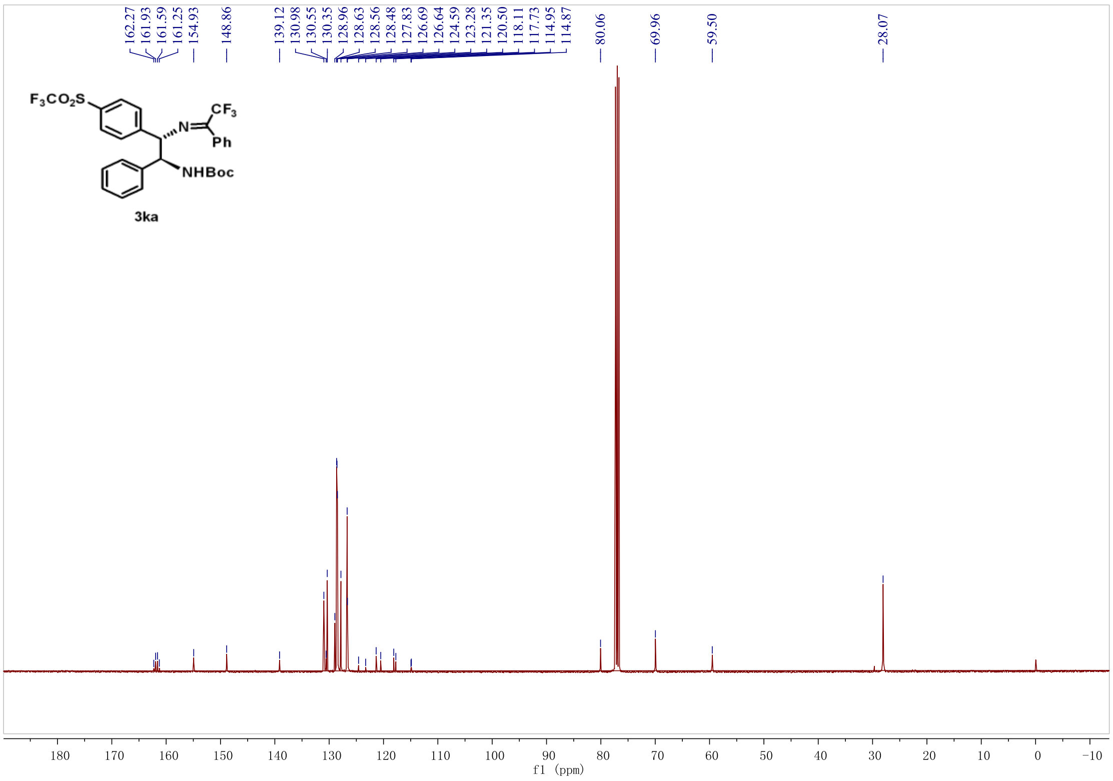

Supplementary Figure 164. ^{13}C NMR spectrum for compound **3ka**

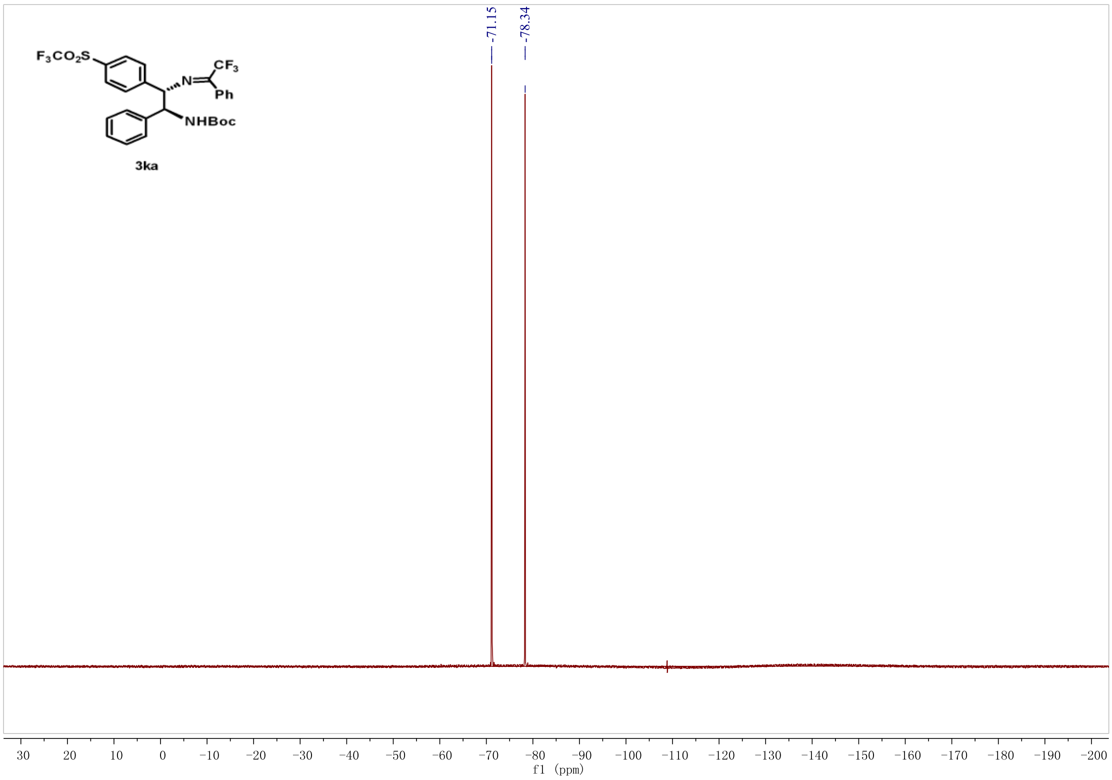

Supplementary Figure 165. ^{19}F NMR spectrum for compound **3ka**

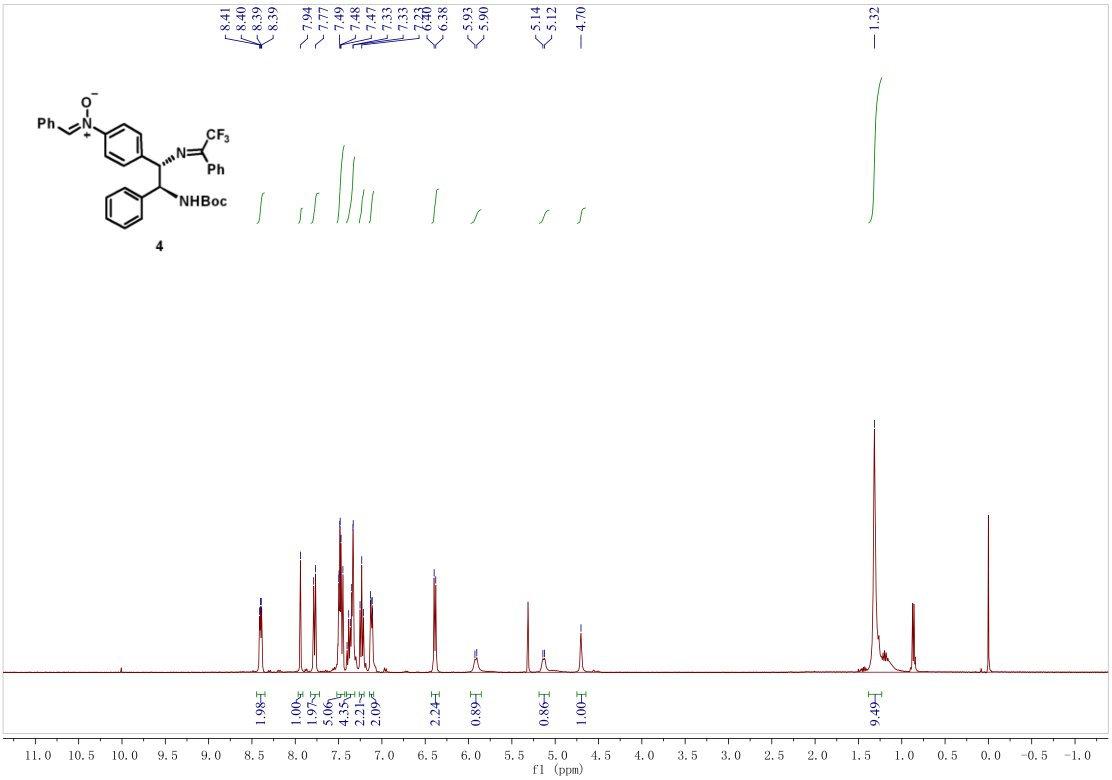

Supplementary Figure 166. ^{1}H NMR spectrum for compound **4**

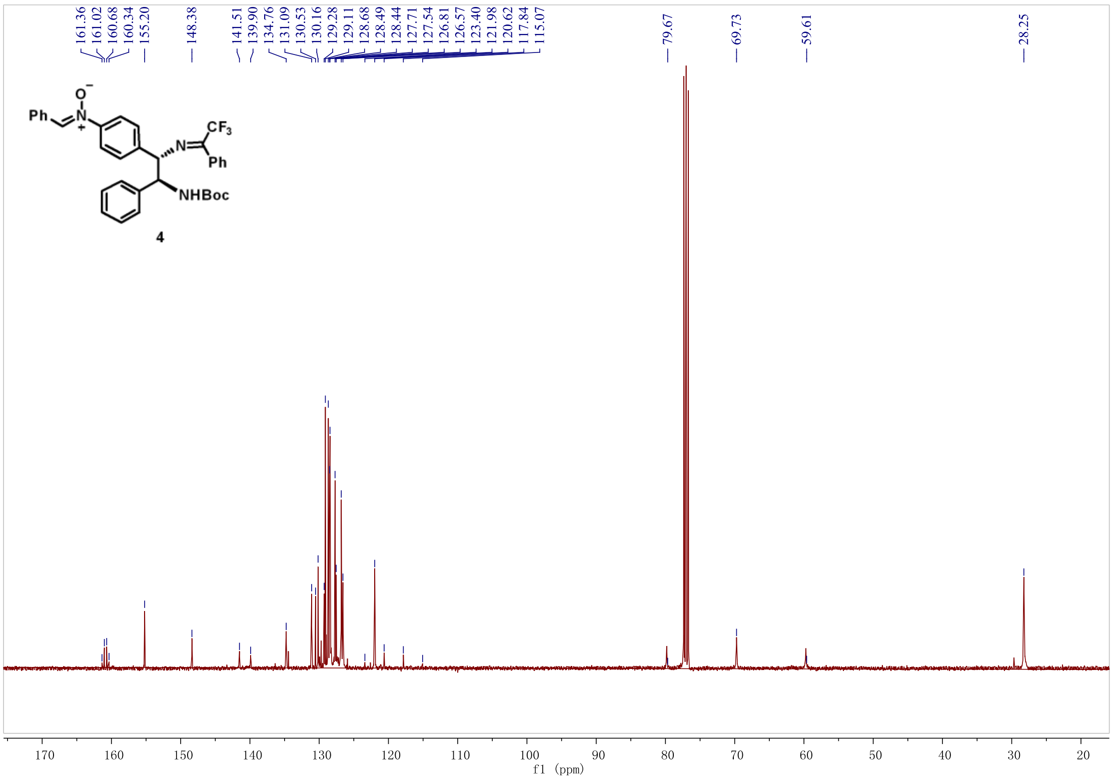

Supplementary Figure 167. ^{13}C NMR spectrum for compound **4**

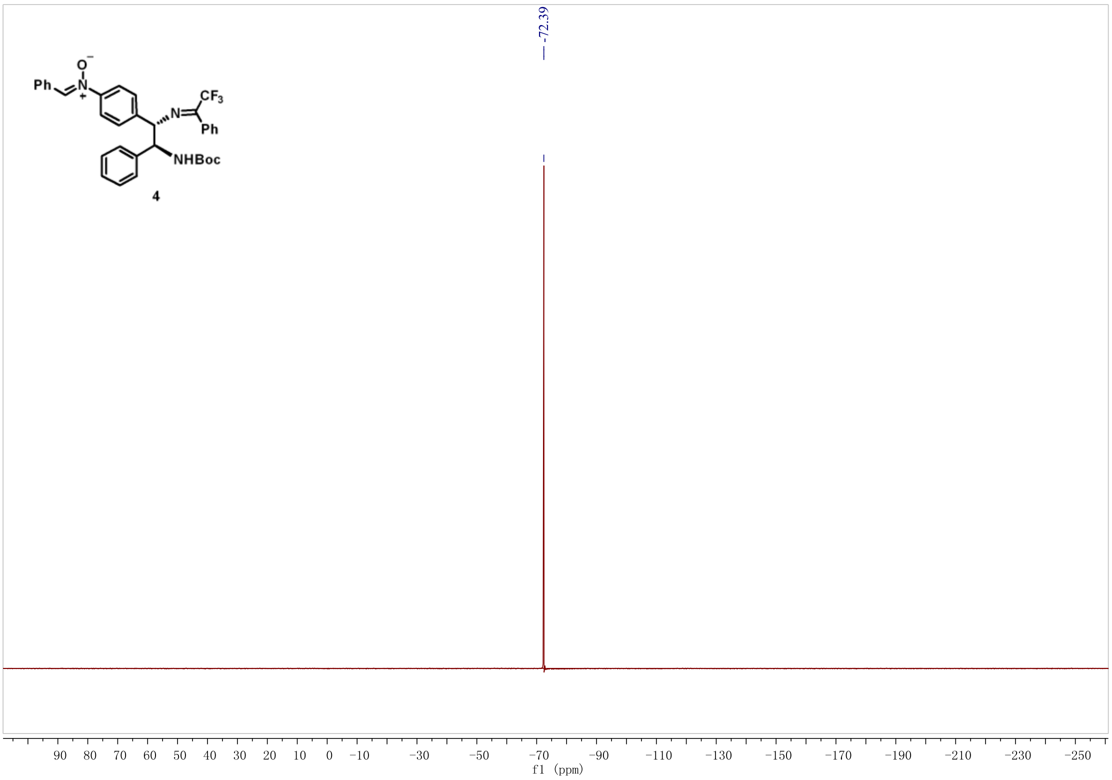

Supplementary Figure 168. ^{19}F NMR spectrum for compound **4**

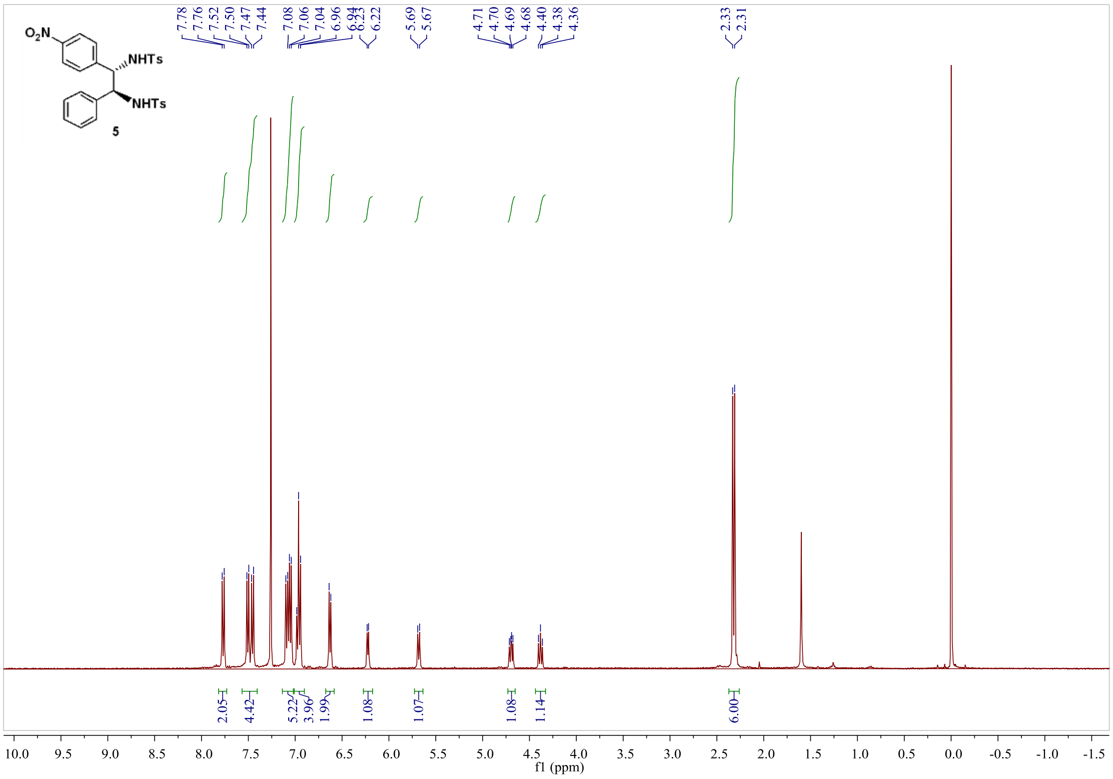

Supplementary Figure 169. ^{1}H NMR spectrum for compound **5**

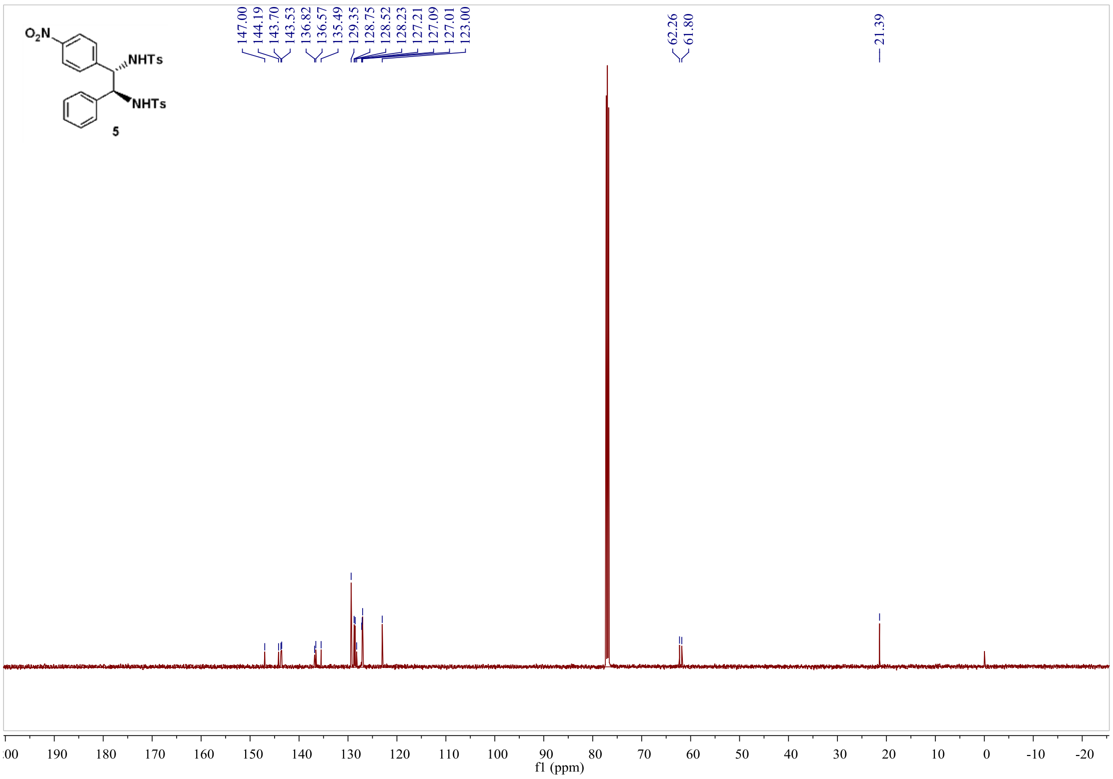

Supplementary Figure 170. ^{13}C NMR spectrum for compound **5**

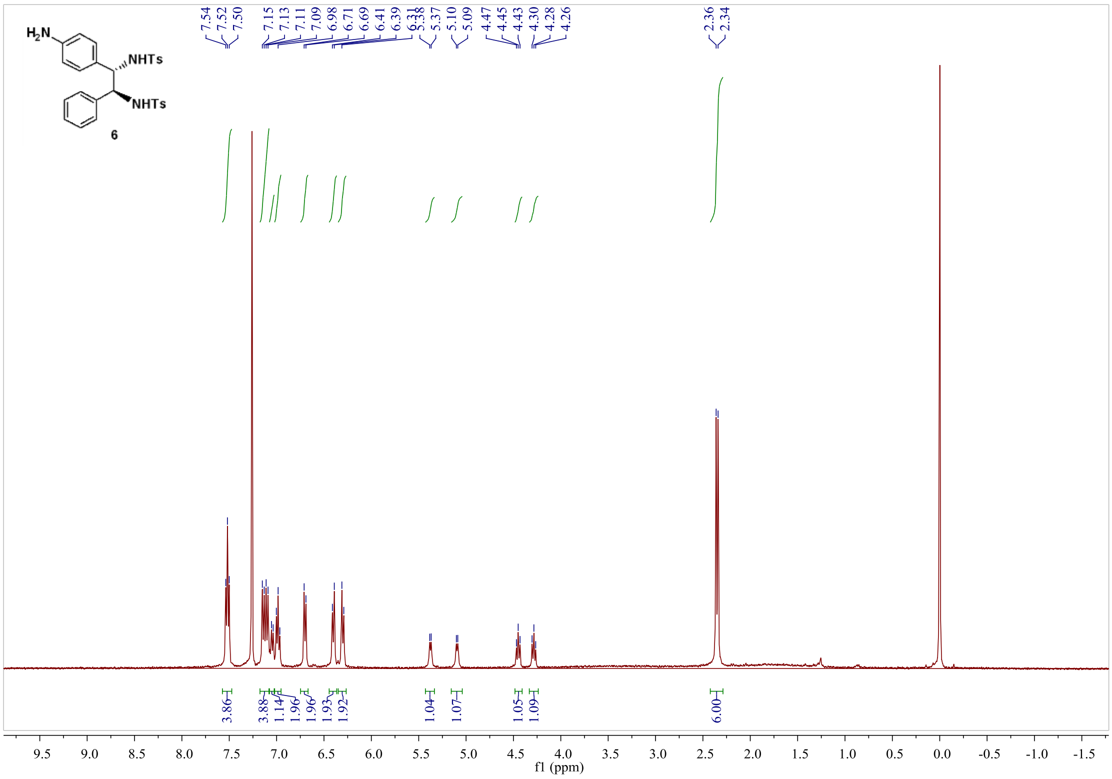

Supplementary Figure 171. ^{1}H NMR spectrum for compound **6**

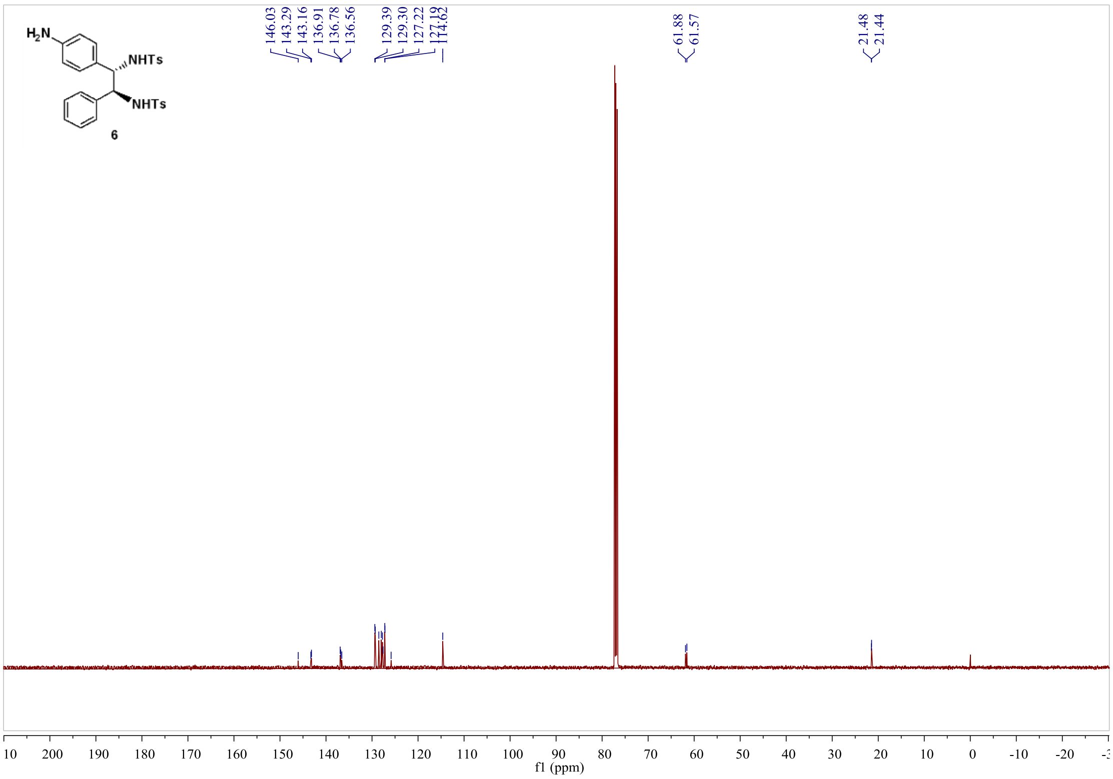

Supplementary Figure 172. ^{13}C NMR spectrum for compound **6**

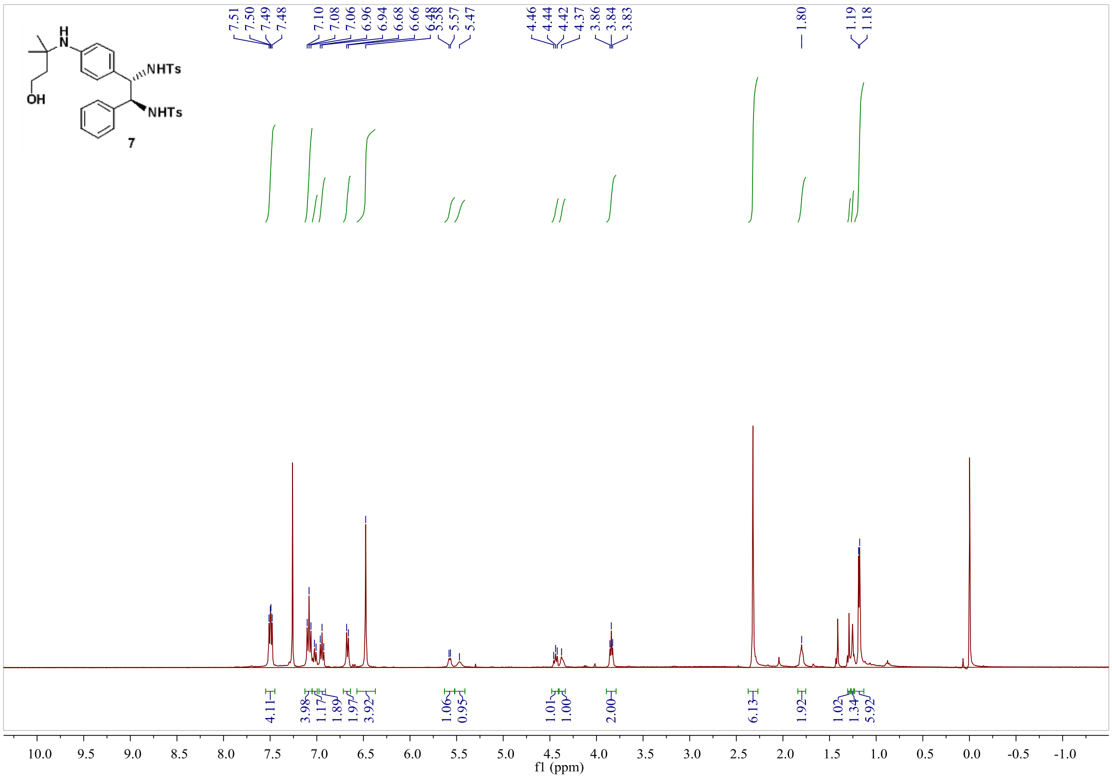

Supplementary Figure 173. ^{1}H NMR spectrum for compound **7**

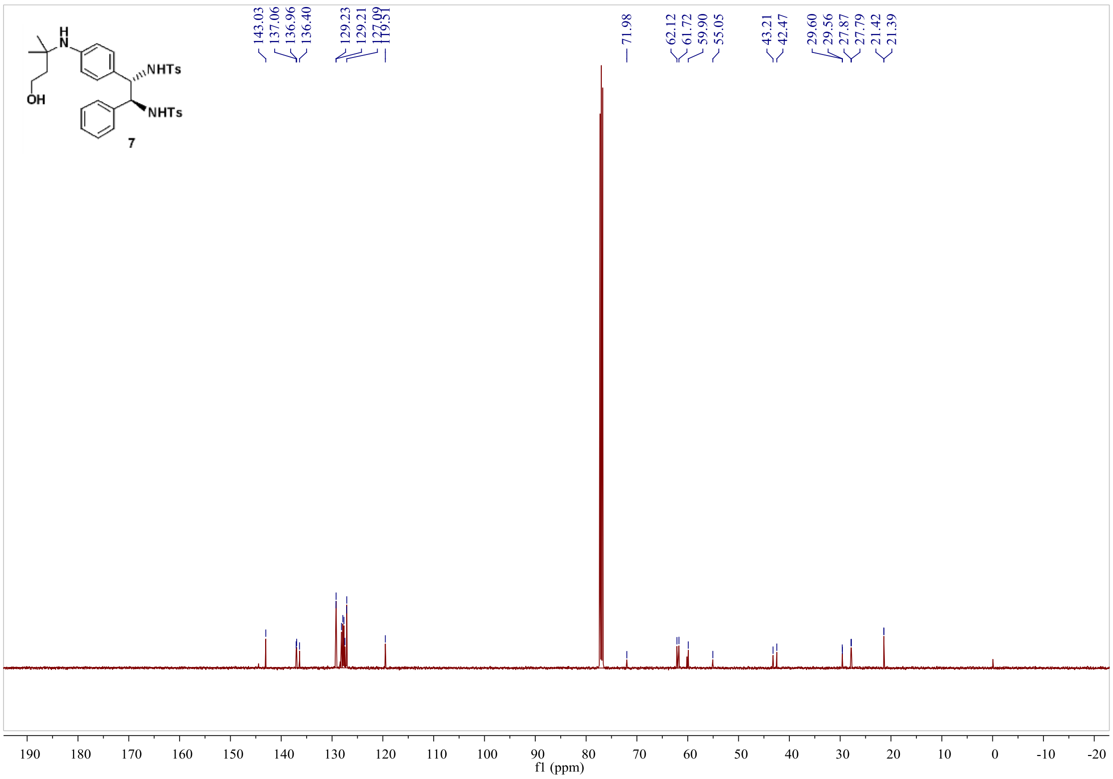

Supplementary Figure 174. ^{13}C NMR spectrum for compound **7**

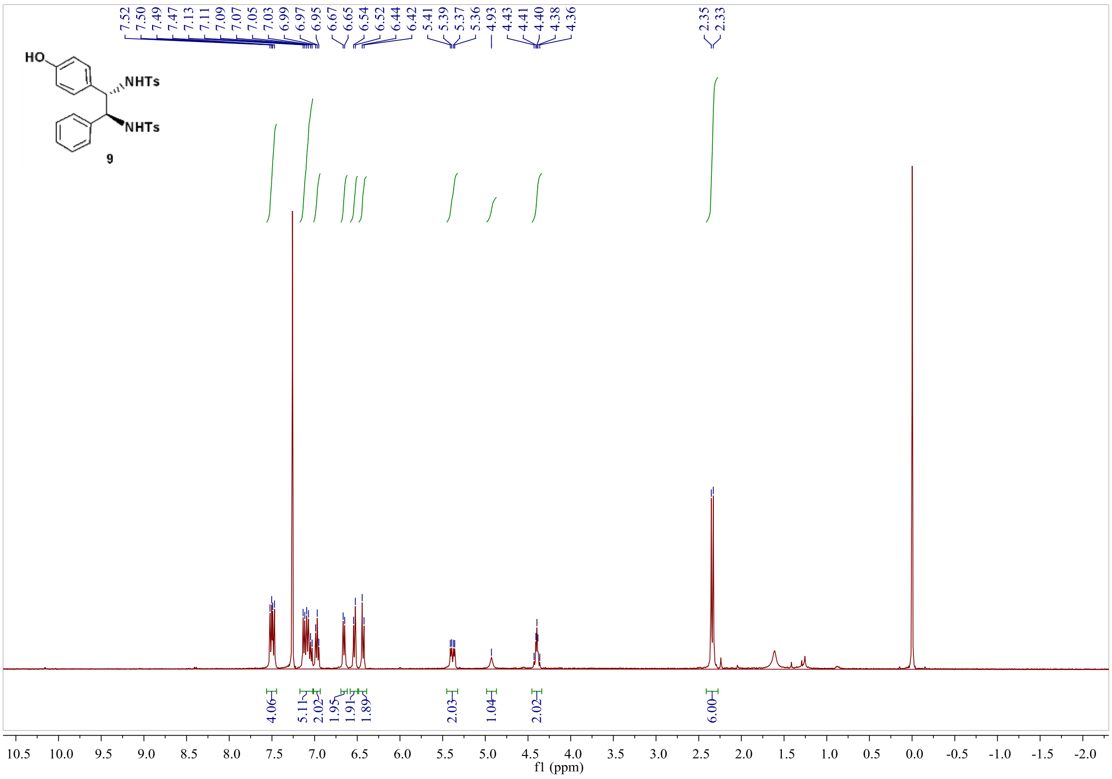

Supplementary Figure 175. ^{1}H NMR spectrum for compound **9**

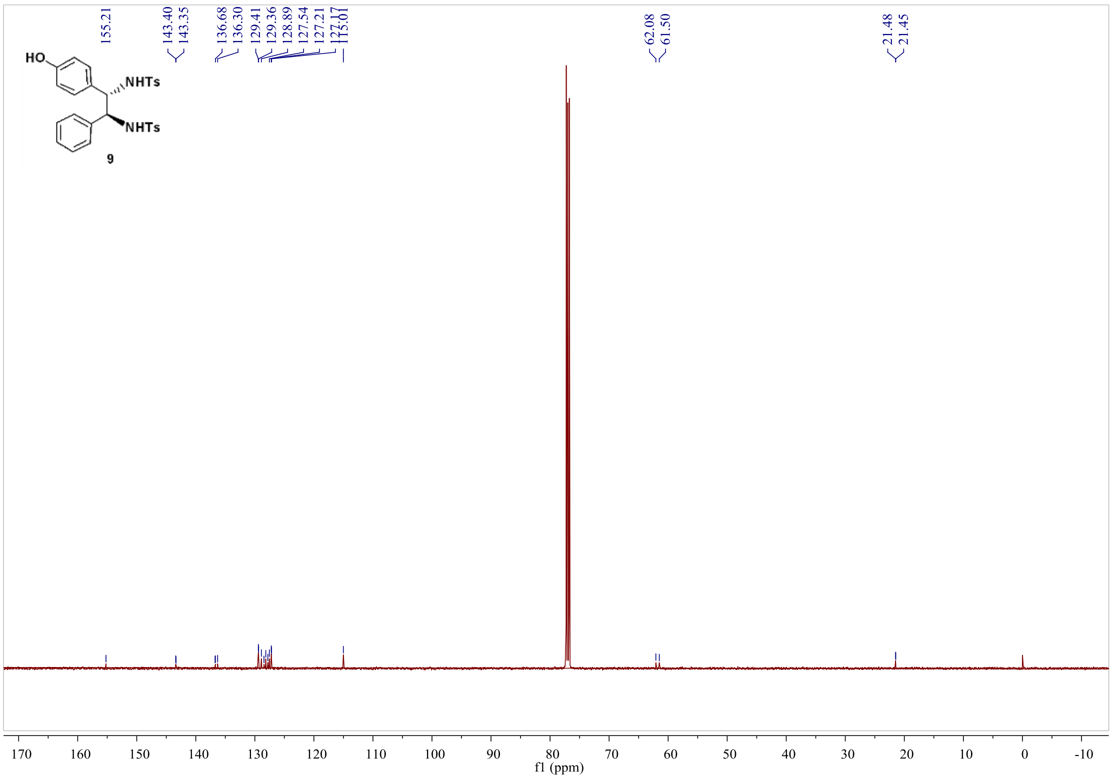

Supplementary Figure 176. ^{13}C NMR spectrum for compound **9**

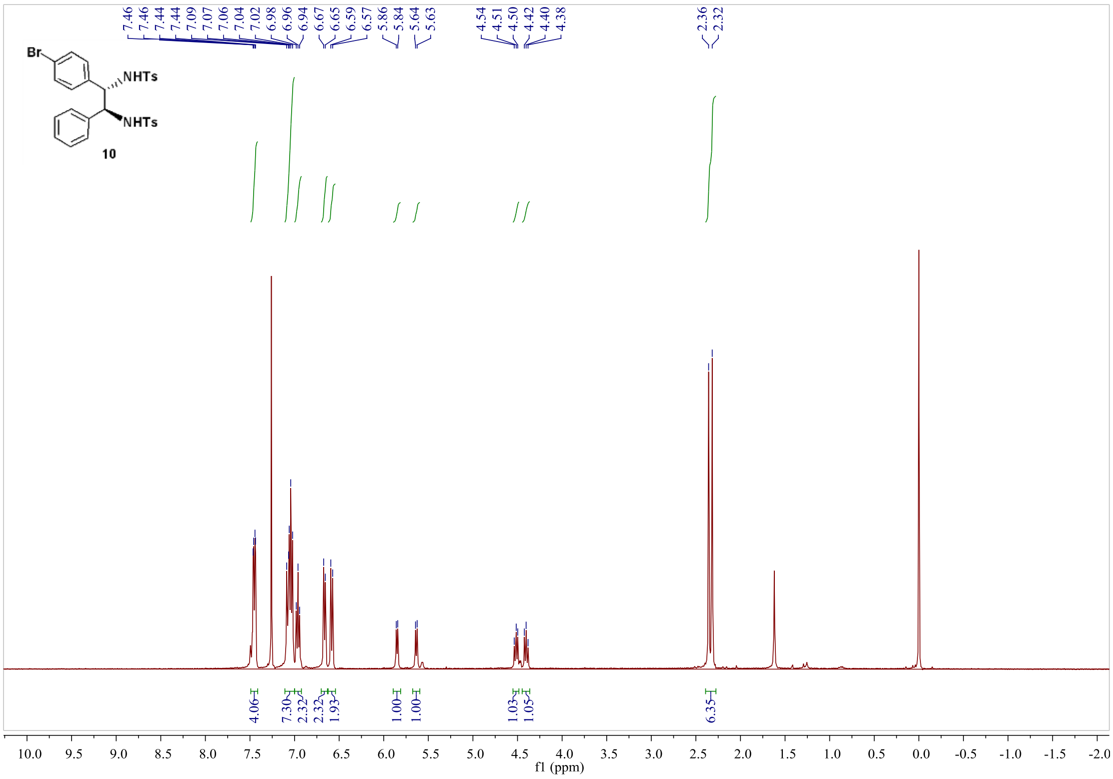

Supplementary Figure 177. ^{1}H NMR spectrum for compound **10**

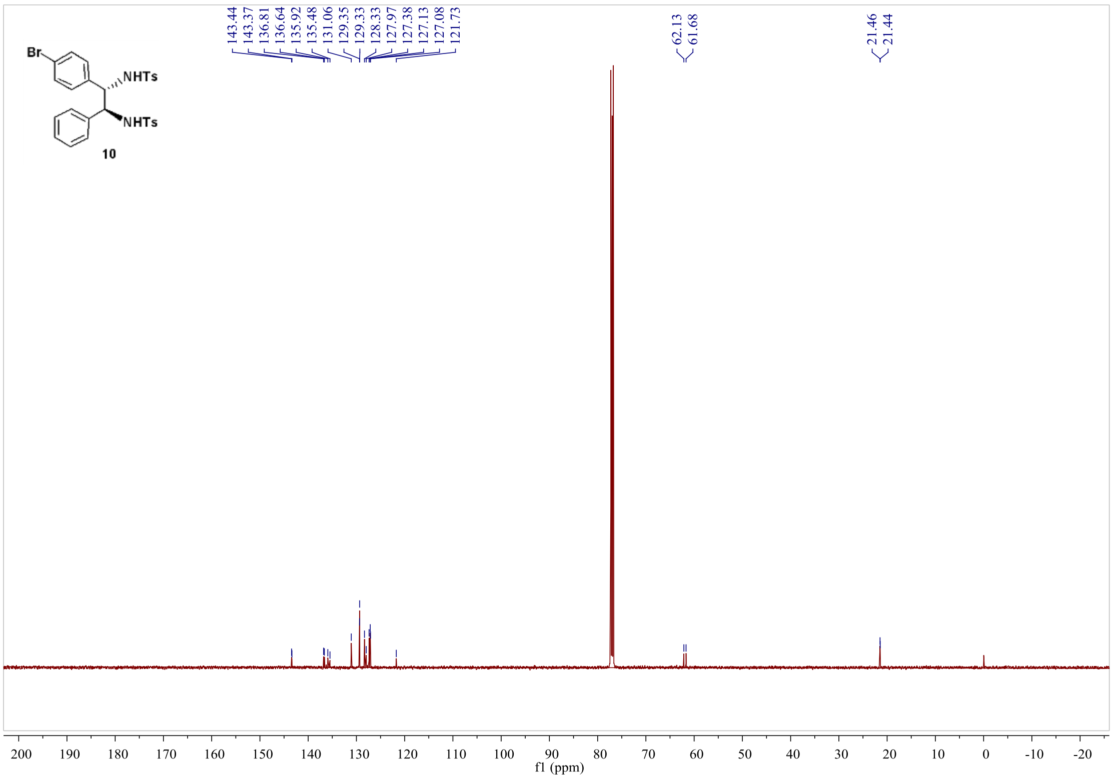

Supplementary Figure 178. ^{13}C NMR spectrum for compound **10**

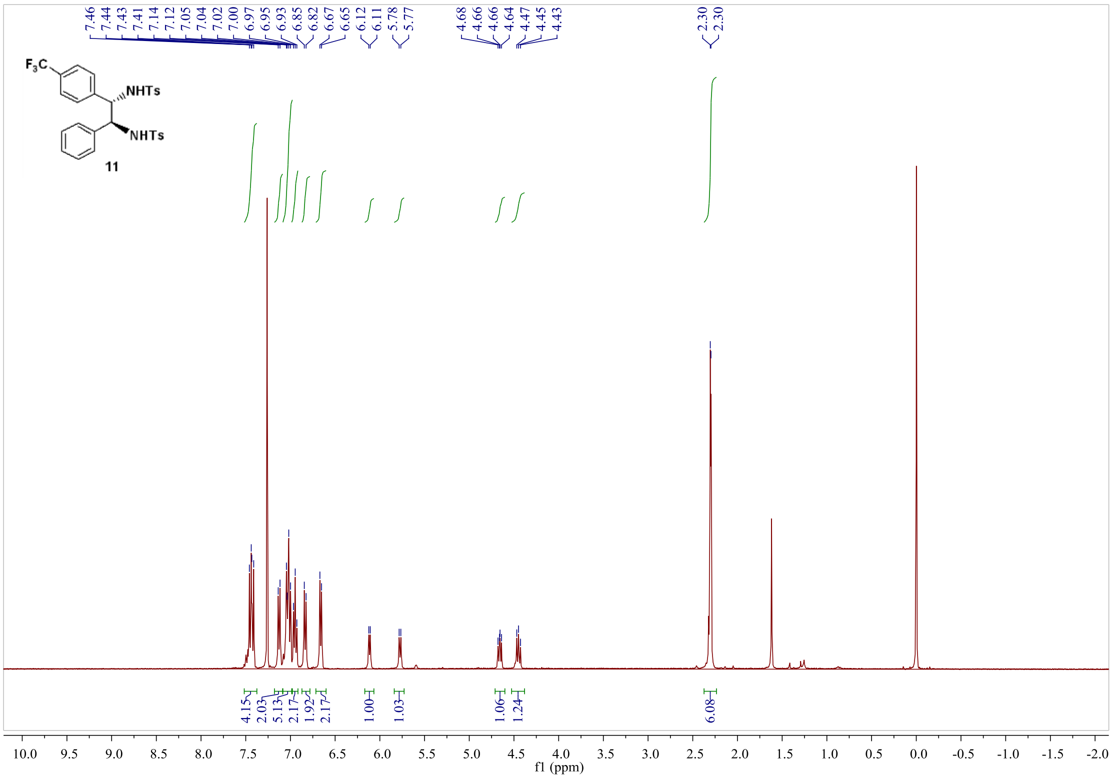

Supplementary Figure 179. ^{1}H NMR spectrum for compound **11**

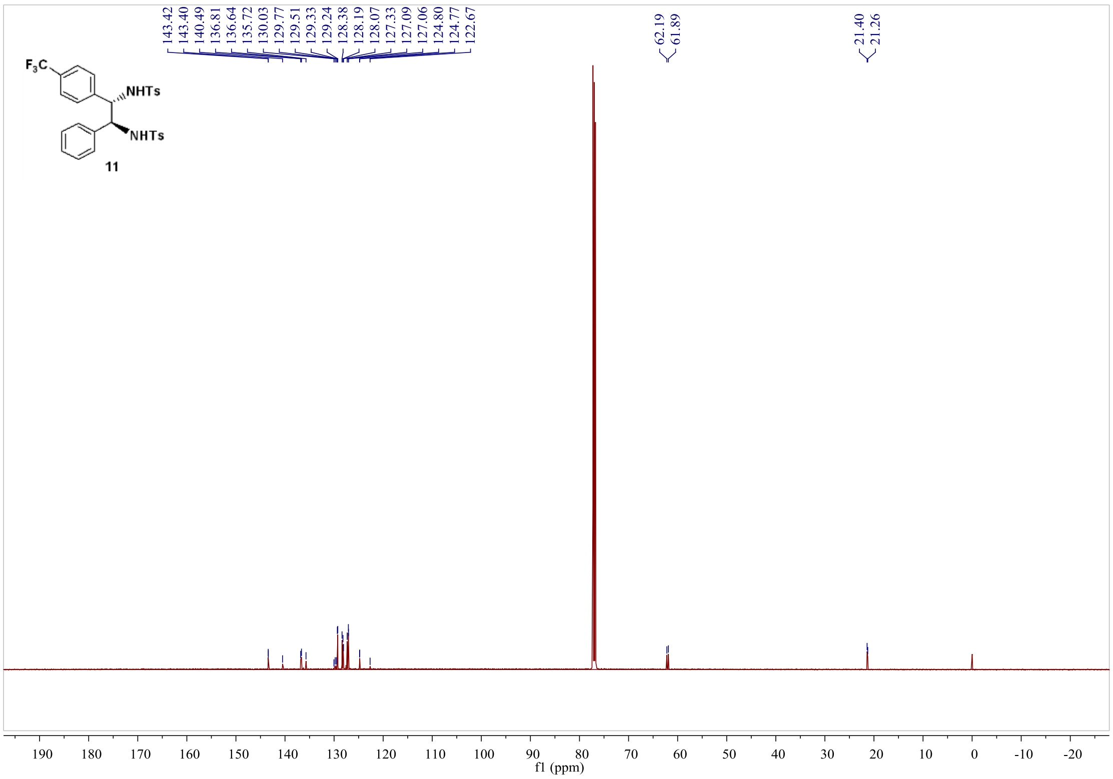

Supplementary Figure 180. ^{13}C NMR spectrum for compound **11**

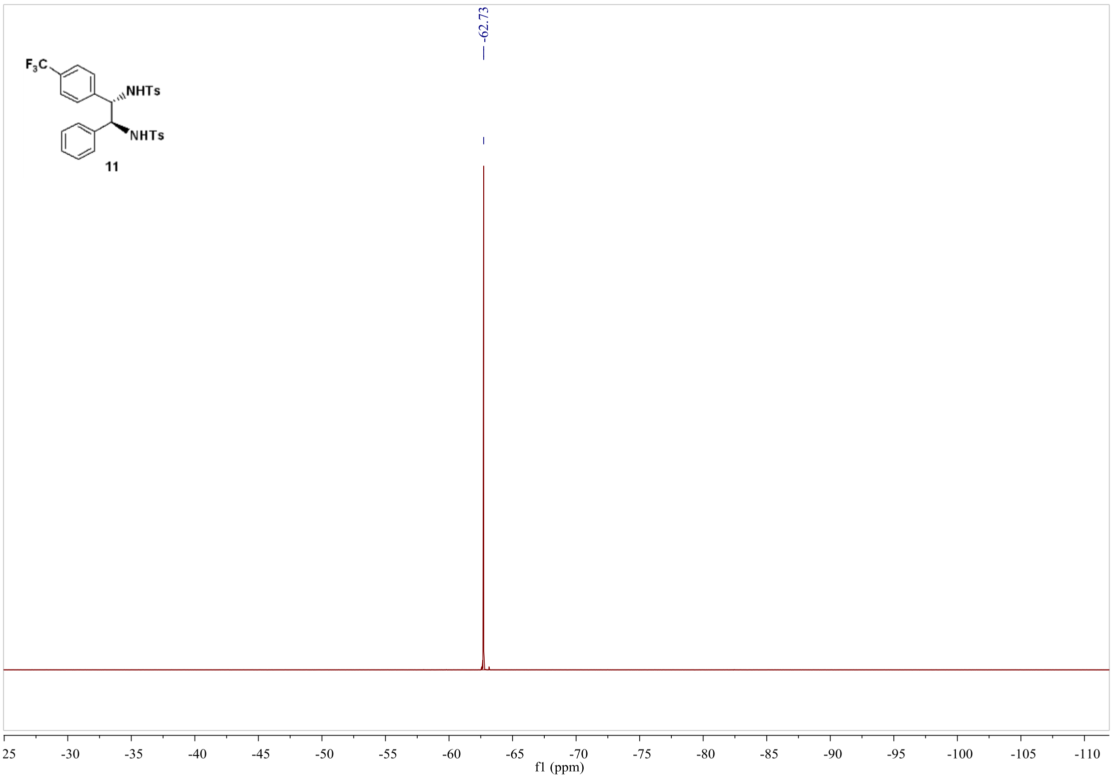

Supplementary Figure 181. ^{19}F NMR spectrum for compound **11**

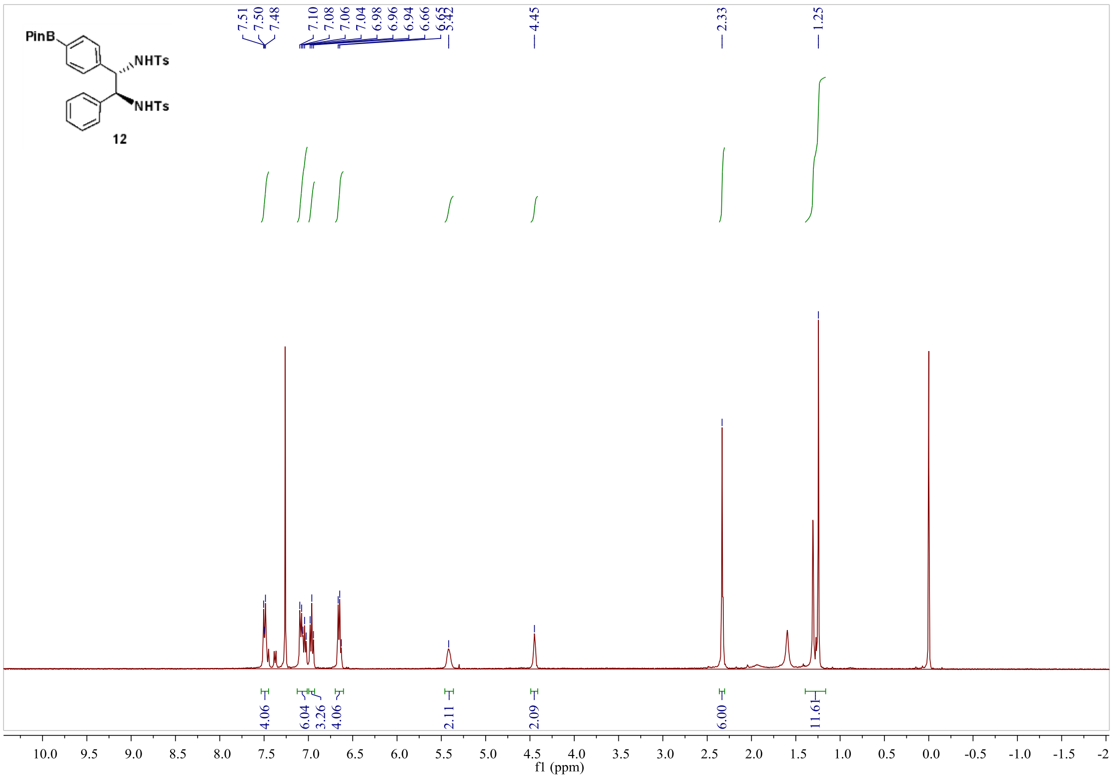

Supplementary Figure 182. ^{1}H NMR spectrum for compound **12**

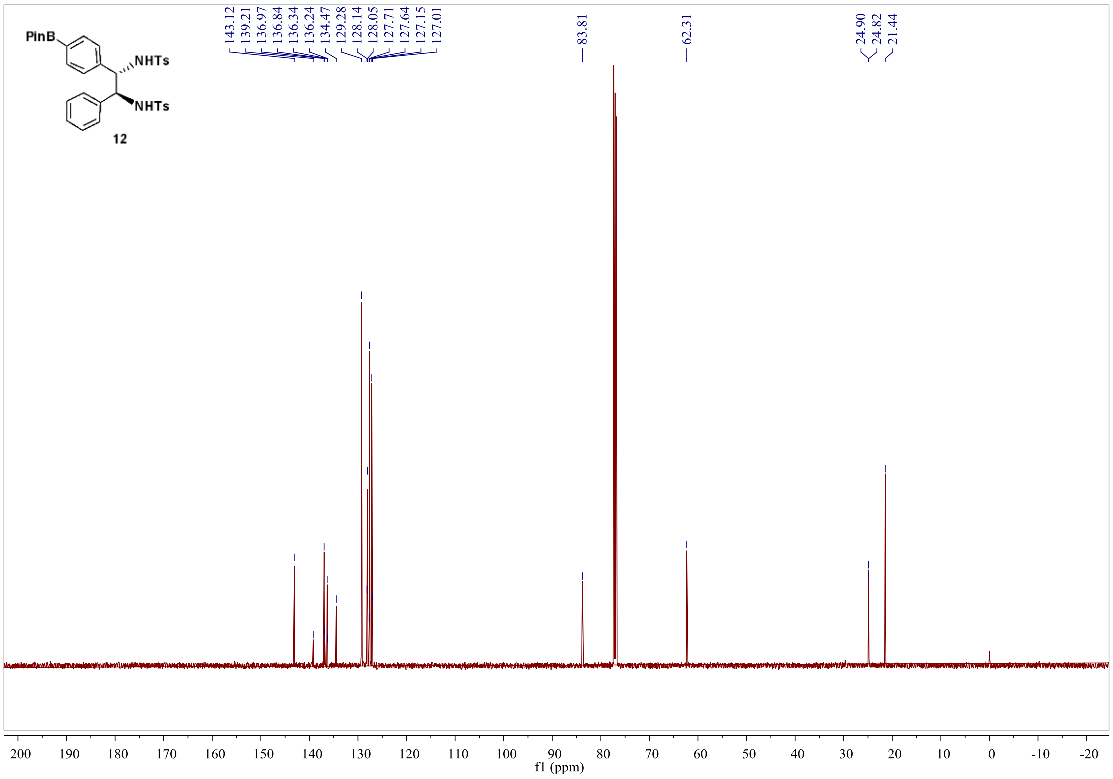

Supplementary Figure 183. ^{13}C NMR spectrum for compound **12**

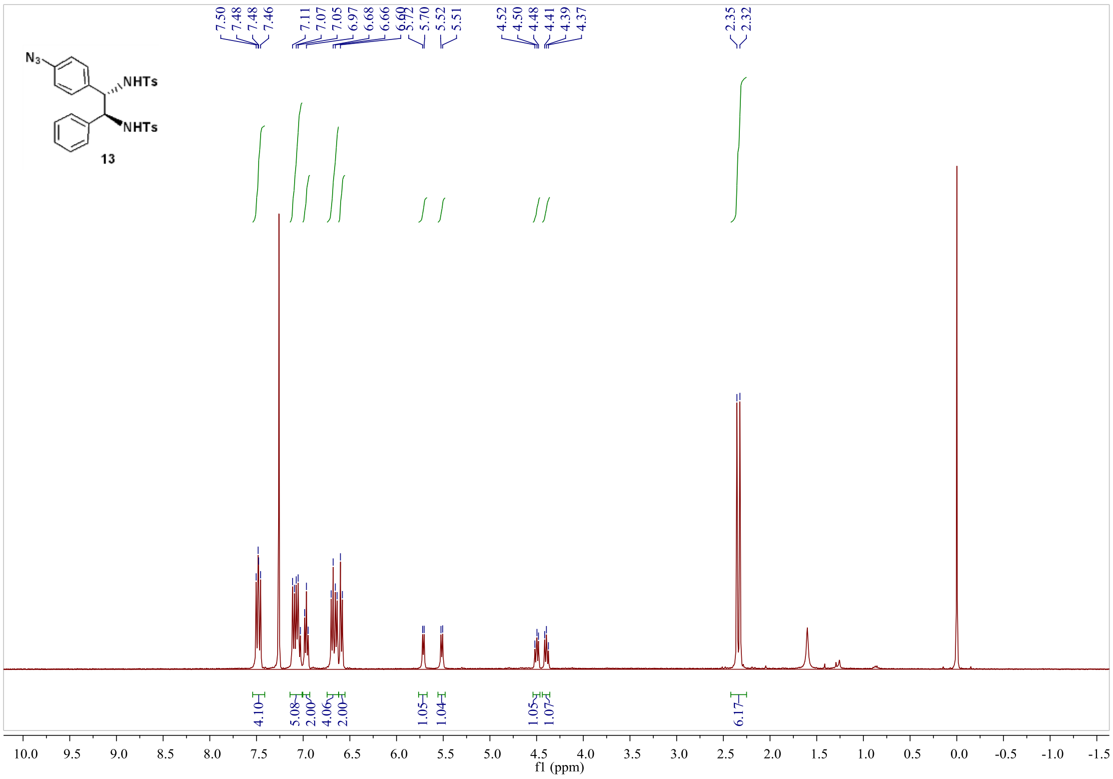

Supplementary Figure 184. ^{1}H NMR spectrum for compound **13**

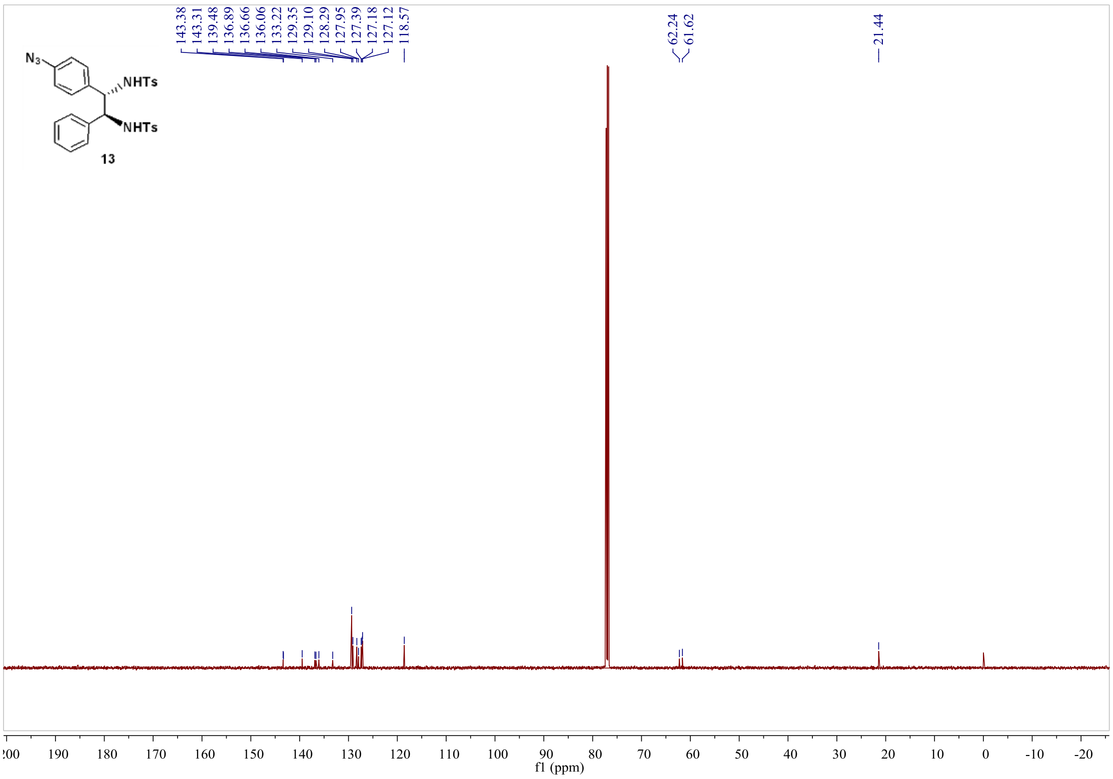

Supplementary Figure 185. ^{13}C NMR spectrum for compound **13**

Supplementary References

[1] Zhou, X., Wu, Y., Deng, L. Cinchonium Betaines as Efficient Catalysts for Asymmetric Proton Transfer Catalysis: The Development of a Practical Enantioselective Isomerization of Trifluoromethyl Imines. *J. Am. Chem. Soc.* **2016**, *138*, 12297.

[2] Matias, A. C., Biazolla, G., Cerchiaro, G., Keppler, A. F. α-Aryl-*N*-aryl nitrones: Synthesis and screening of a new scaffold for cellular protection against an oxidative toxic stimulus. *Bioorg. Med. Chem.* **2016**, *24*, 232.

[3] Gui, J., Pan, C-M., Jin, Y., Qin, T., Lo, J. C., Lee, B. J., Spergel, S. H., Mertzman, M. E., Pitts, W. J., Cruz, T. E. L., Schmidt, Darvatkar, M. A., N., Natarajan, S. R., Baran, P. S. Practical olefin hydroamination with nitroarenes. *Science* **2015**, *348*, 886.

[4] Hilgraf, R., Pfaltz, A. Chiral Bis(*N*-sulfonylamino)phosphine- and TADDOL-Phosphite-Oxazoline Ligands: Synthesis and Application in Asymmetric Catalysis. *Adv. Synth. Catal.* **2005**, *347*, 61.

[5] Wang, X., Xu, Y., Mo, F., Ji, G., Qiu, D., Feng, J., Ye, Y., Zhang, S., Zhang, Y., Wang, J. Silver-Mediated Trifluoromethylation of Aryldiazonium Salts: Conversion of Amino Group into Trifluoromethyl Group. *J. Am. Chem. Soc.* **2013**, *135*, 10330.

[6] Qiu, D., Jin, L., Zheng, Z., Meng, H., Mo, F., Wang, X., Zhang, Y., Wang, J. Synthesis of Pinacol Arylboronates from Aromatic Amines: A Metal-Free Transformation. *J. Org. Chem.* **2013**, *78*, 1923.

[7] Meng, G., Guo, T., Ma, T., Zhang, J., Shen, Y., Sharpless, K. B., Dong, J. Modular click chemistry libraries for functional screens using a diazotizing reagent. *Nature* **2019**, *574*, 86.
